# Supplementary figures and images for: A novel single alpha-helix DNA-binding domain in CAF-1 promotes gene silencing and DNA damage survival through tetrasome-length DNA selectivity and spacer function (part 1 of 2)
Source: eLife. 2023 Jul 11;12:e83538. doi: 10.7554/eLife.83538 (PMC10335832; doi:10.7554/eLife.83538)

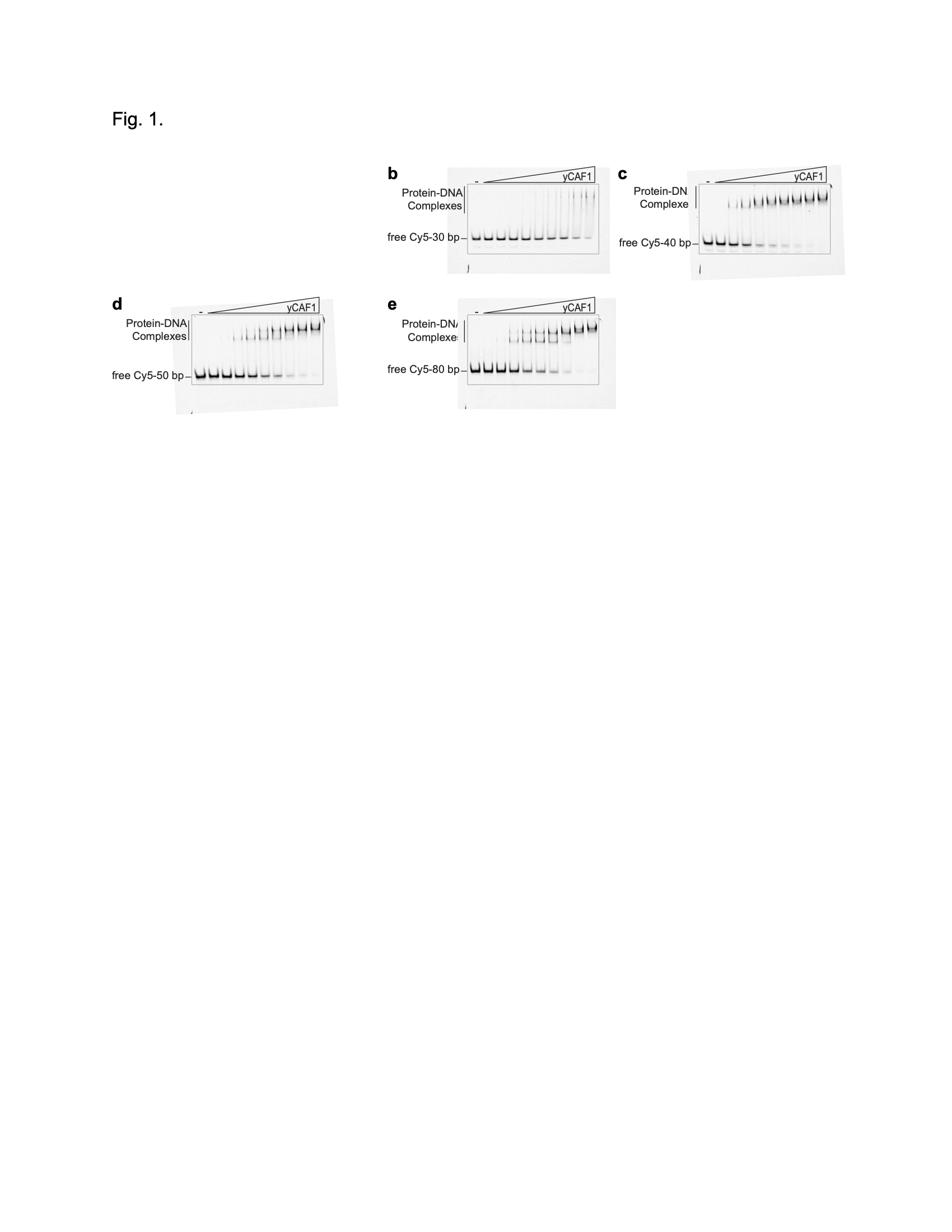

Supplement: Figure 1—source data 1. — Electrophoretic mobility shift assay (EMSA) images (panels b–e) and data analyses (panel j). [file elife-83538-fig1-data1.zip › Figure 1 - Source data 1/Figure 1 - Source data 1_Gels Labeled.png]

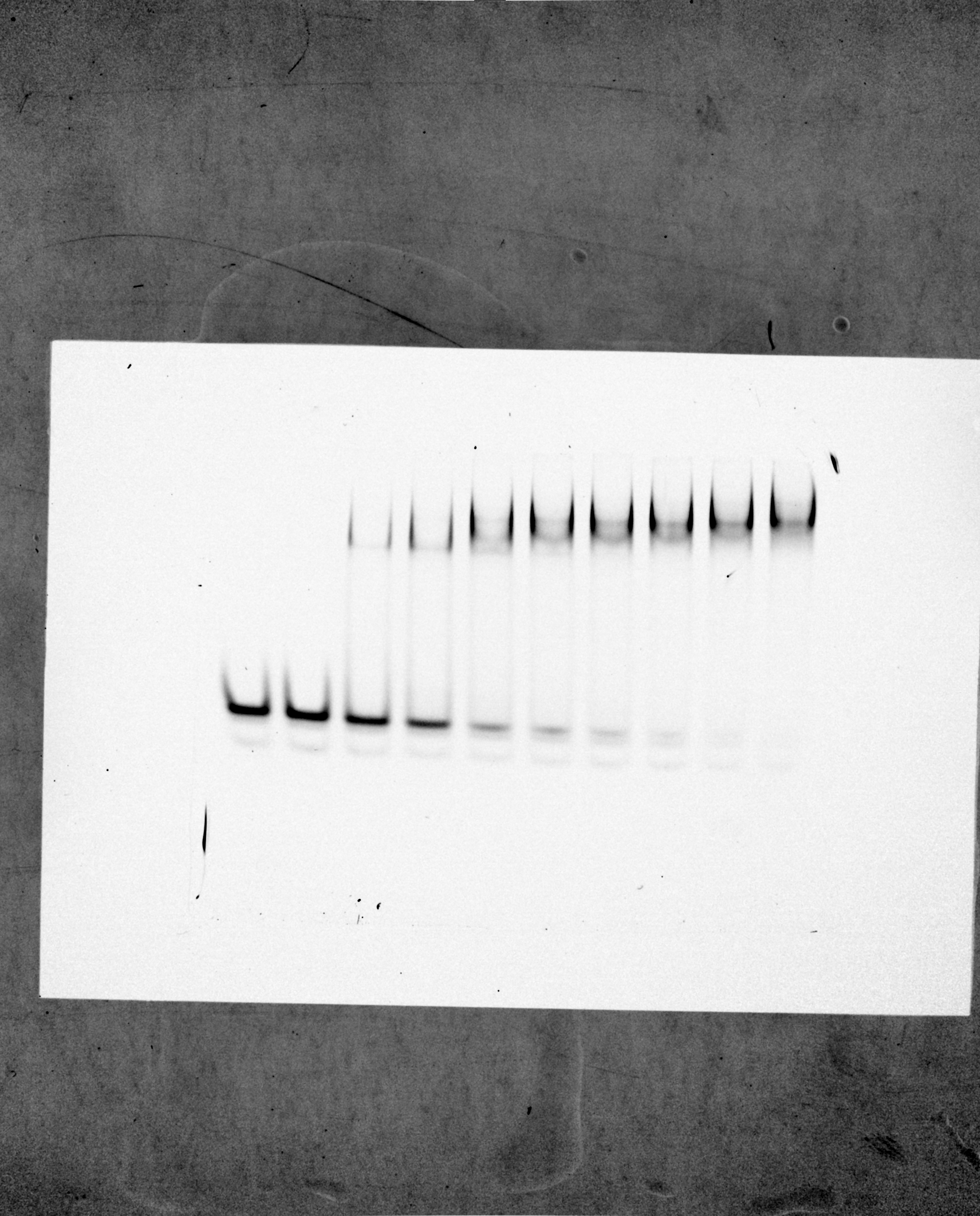

Supplement: Figure 1—source data 1. — Electrophoretic mobility shift assay (EMSA) images (panels b–e) and data analyses (panel j). [file elife-83538-fig1-data1.zip › Figure 1 - Source data 1/c/211004 Cy5 40 bp EMSA with yCAF1 WT_PUB_600.tif]

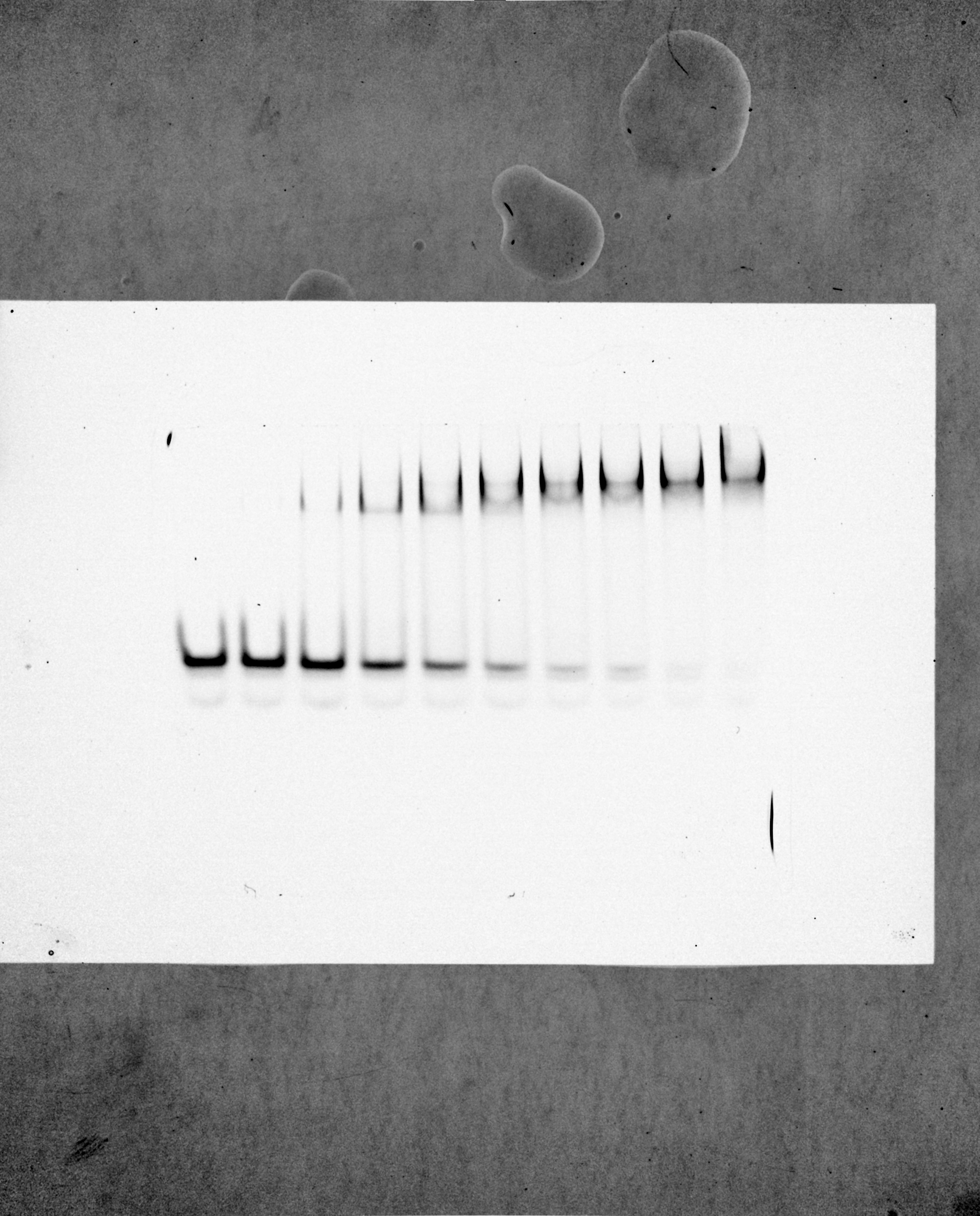

Supplement: Figure 1—source data 1. — Electrophoretic mobility shift assay (EMSA) images (panels b–e) and data analyses (panel j). [file elife-83538-fig1-data1.zip › Figure 1 - Source data 1/c/210921 Cy5 40bp EMSA with yCAF1 WT_PUB_600.tif]

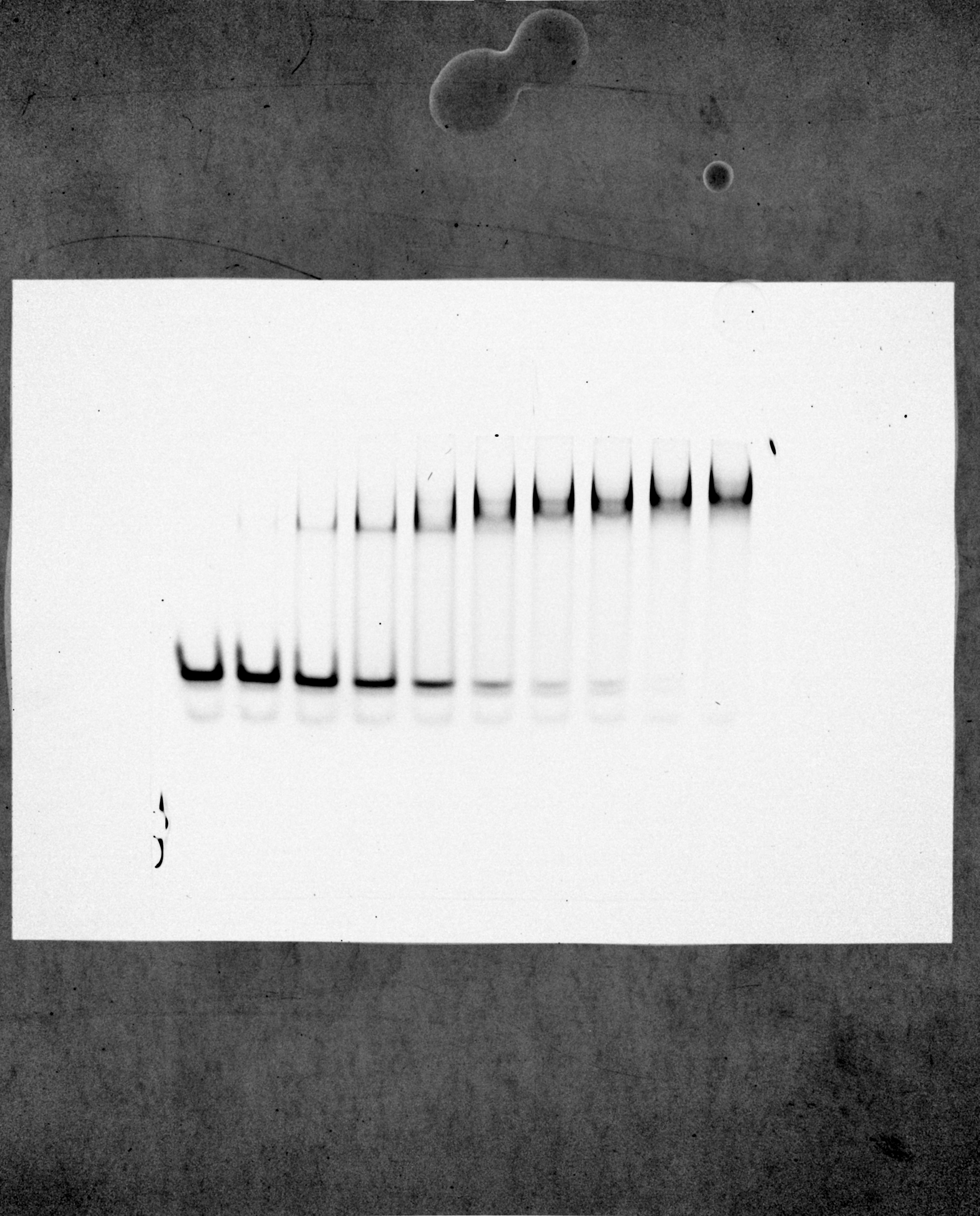

Supplement: Figure 1—source data 1. — Electrophoretic mobility shift assay (EMSA) images (panels b–e) and data analyses (panel j). [file elife-83538-fig1-data1.zip › Figure 1 - Source data 1/c/211001 Cy5 40bp EMSA with yCAF1 WT_PUB_600.tif]

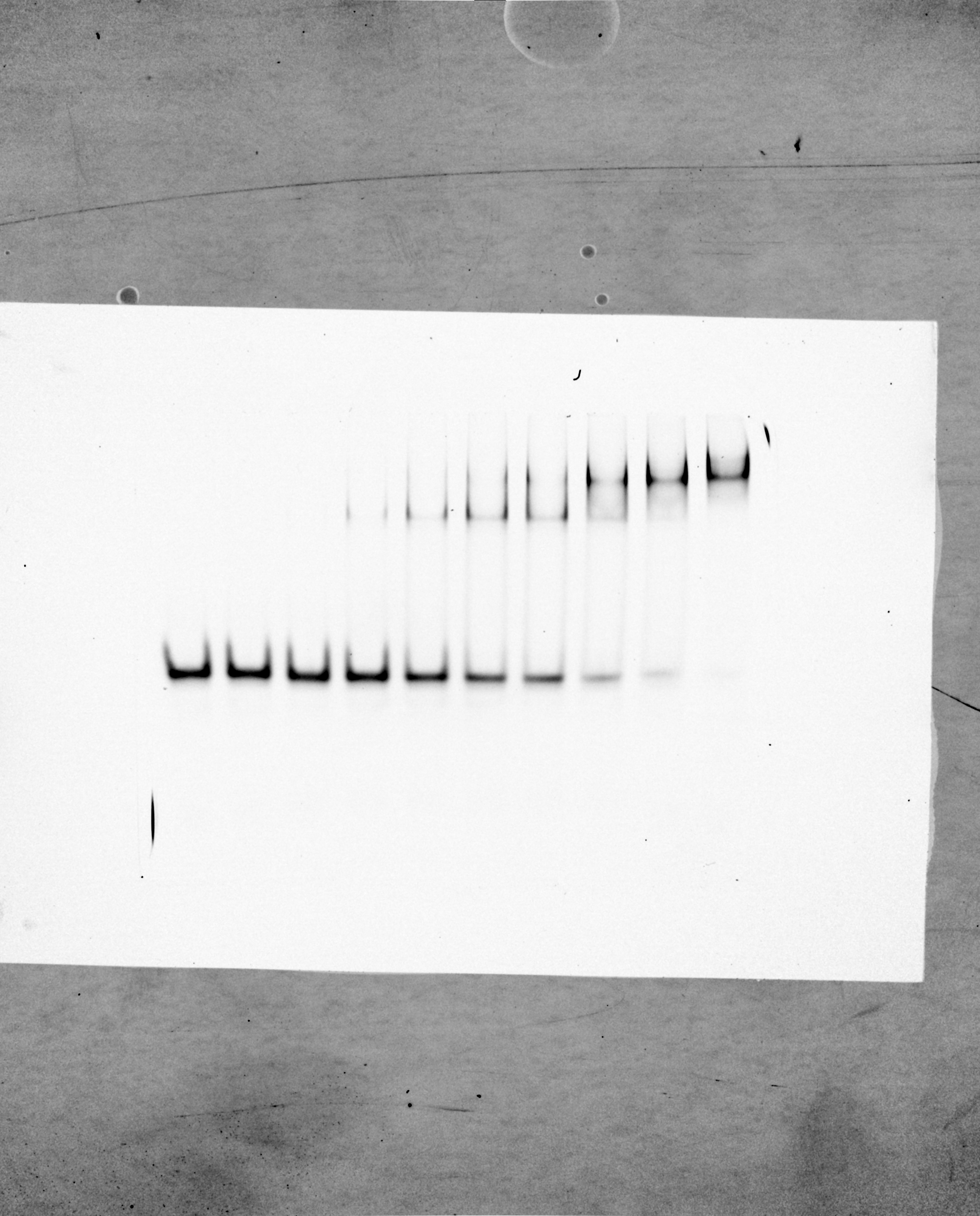

Supplement: Figure 1—source data 1. — Electrophoretic mobility shift assay (EMSA) images (panels b–e) and data analyses (panel j). [file elife-83538-fig1-data1.zip › Figure 1 - Source data 1/d/210921 Cy5 50bp EMSA with yCAF1 WT_PUB_600.tif]

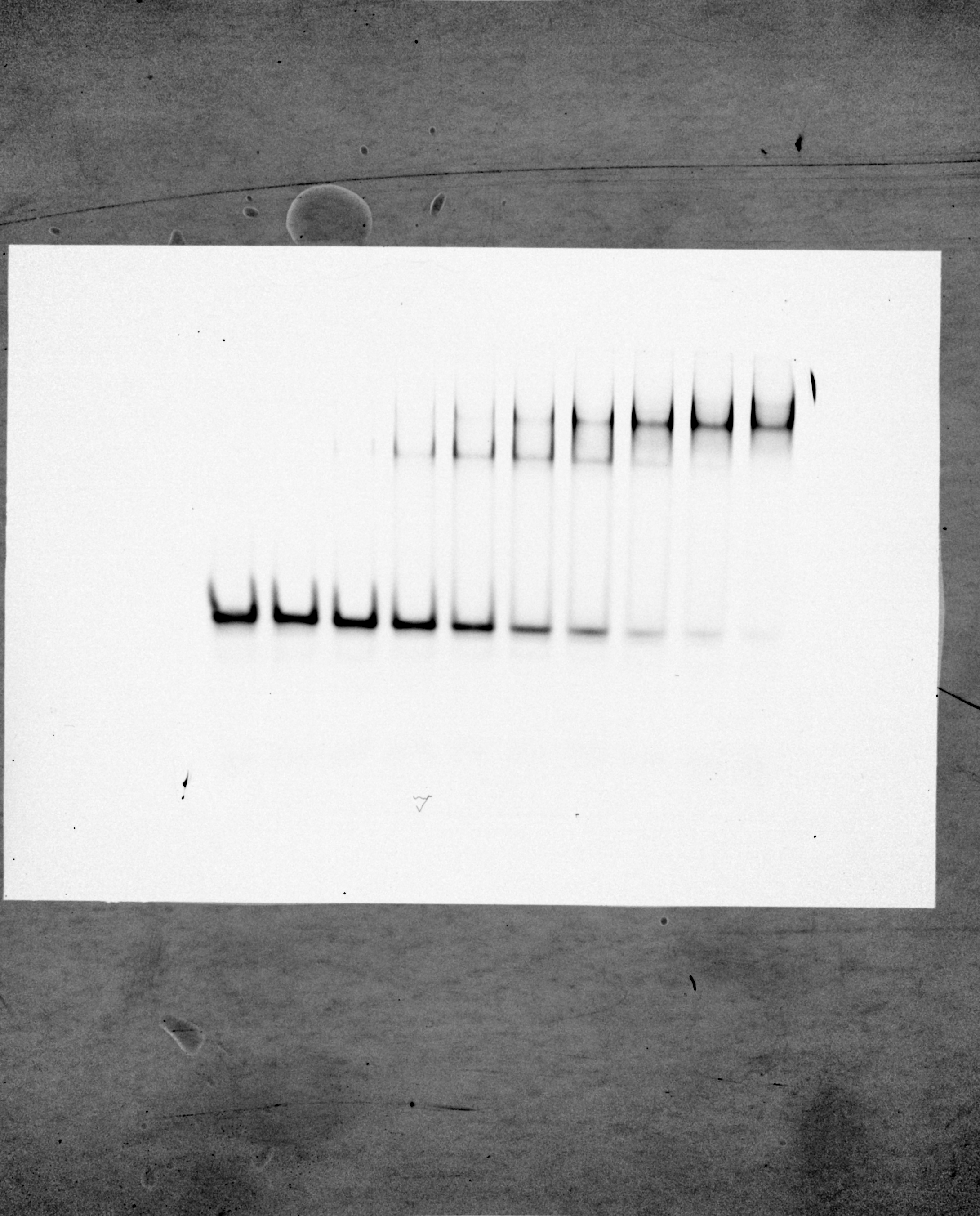

Supplement: Figure 1—source data 1. — Electrophoretic mobility shift assay (EMSA) images (panels b–e) and data analyses (panel j). [file elife-83538-fig1-data1.zip › Figure 1 - Source data 1/d/211004 Cy5 50 bp EMSA with yCAF1 WT_PUB_600.tif]

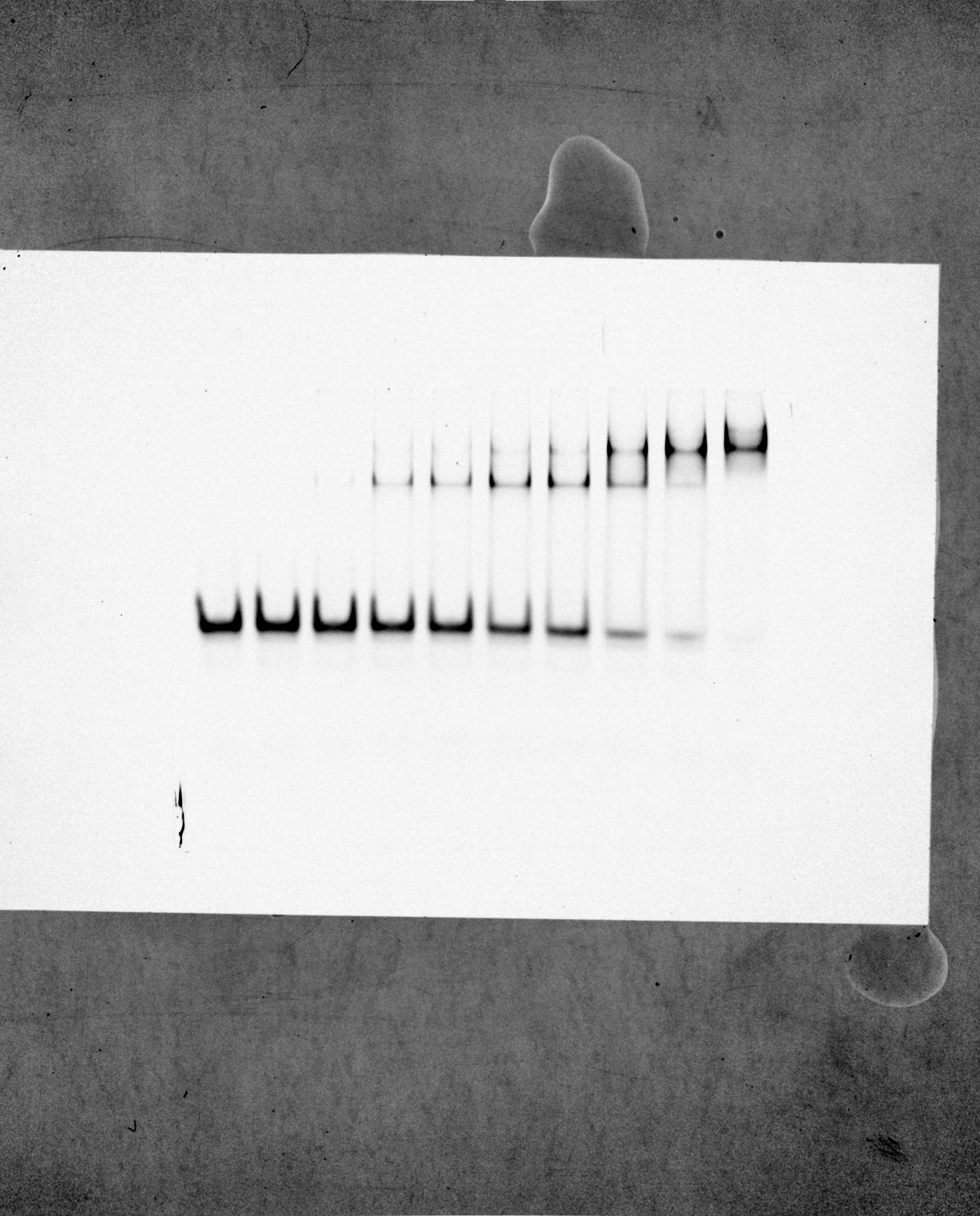

Supplement: Figure 1—source data 1. — Electrophoretic mobility shift assay (EMSA) images (panels b–e) and data analyses (panel j). [file elife-83538-fig1-data1.zip › Figure 1 - Source data 1/d/211001 Cy5 50bp EMSA with yCAF1 WT_PUB_600.tif]

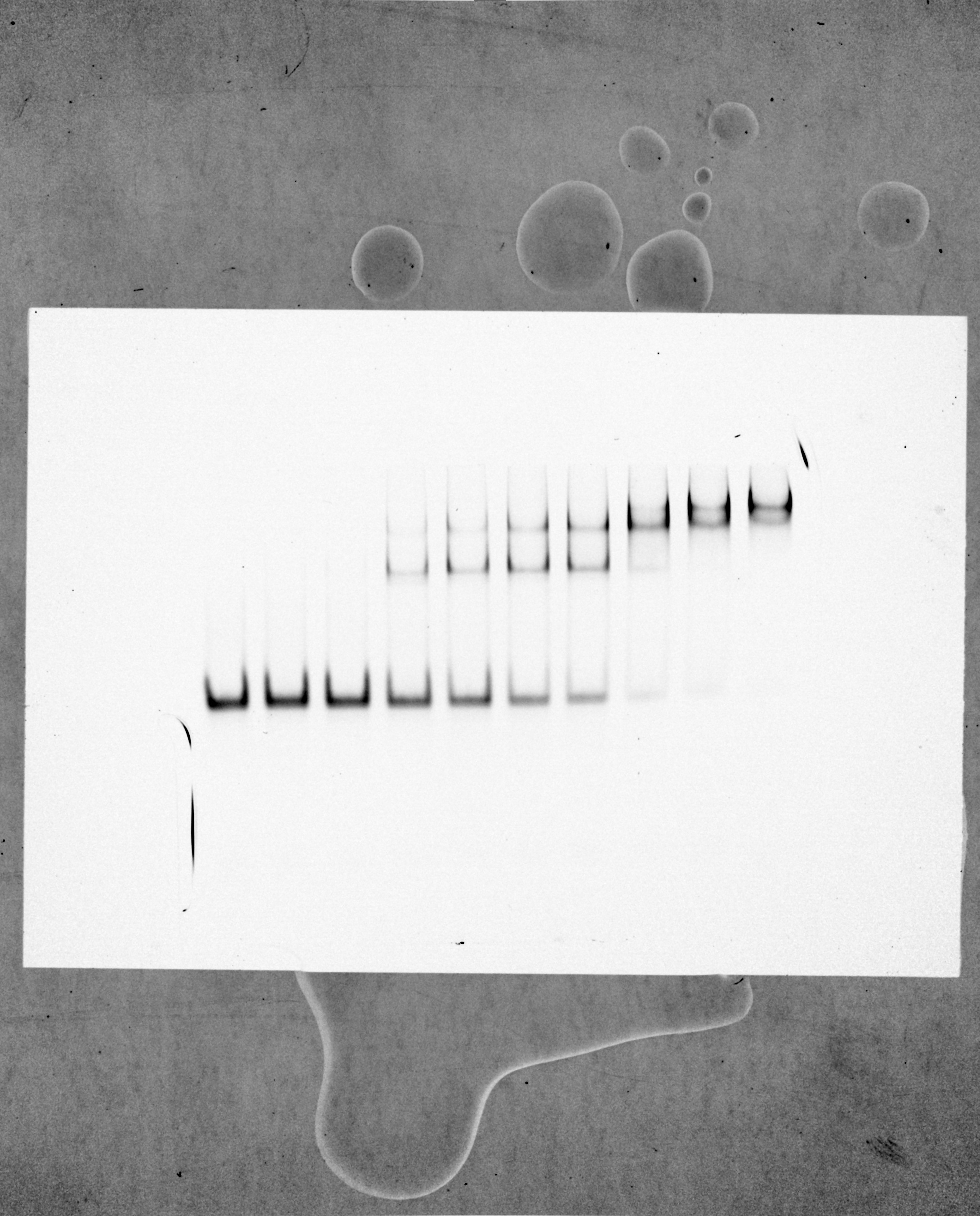

Supplement: Figure 1—source data 1. — Electrophoretic mobility shift assay (EMSA) images (panels b–e) and data analyses (panel j). [file elife-83538-fig1-data1.zip › Figure 1 - Source data 1/e/210921 Cy5 80bp EMSA with yCAF1 WT_PUB_600.tif]

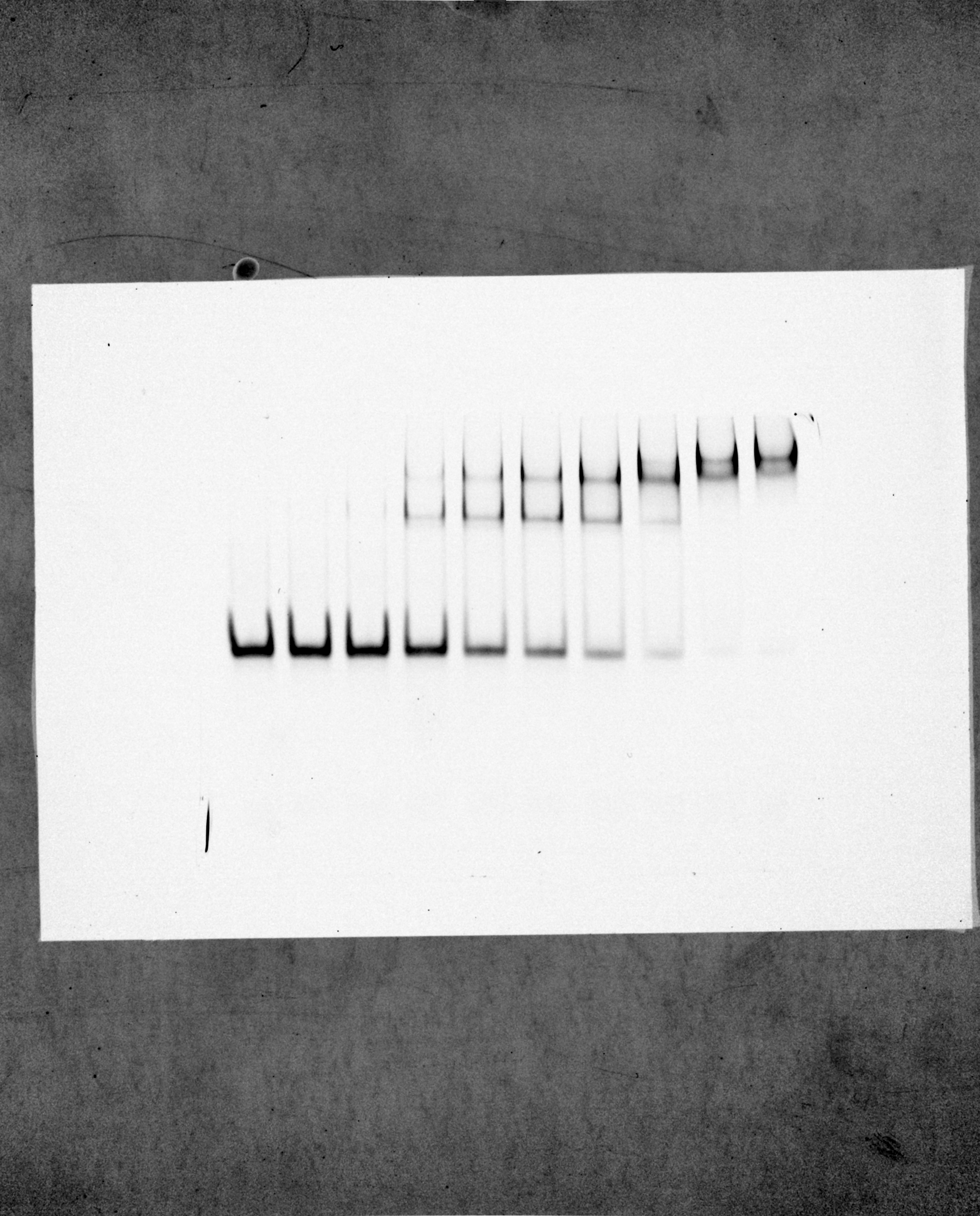

Supplement: Figure 1—source data 1. — Electrophoretic mobility shift assay (EMSA) images (panels b–e) and data analyses (panel j). [file elife-83538-fig1-data1.zip › Figure 1 - Source data 1/e/211004 Cy5 80 bp EMSA with yCAF1 WT_PUB_600.tif]

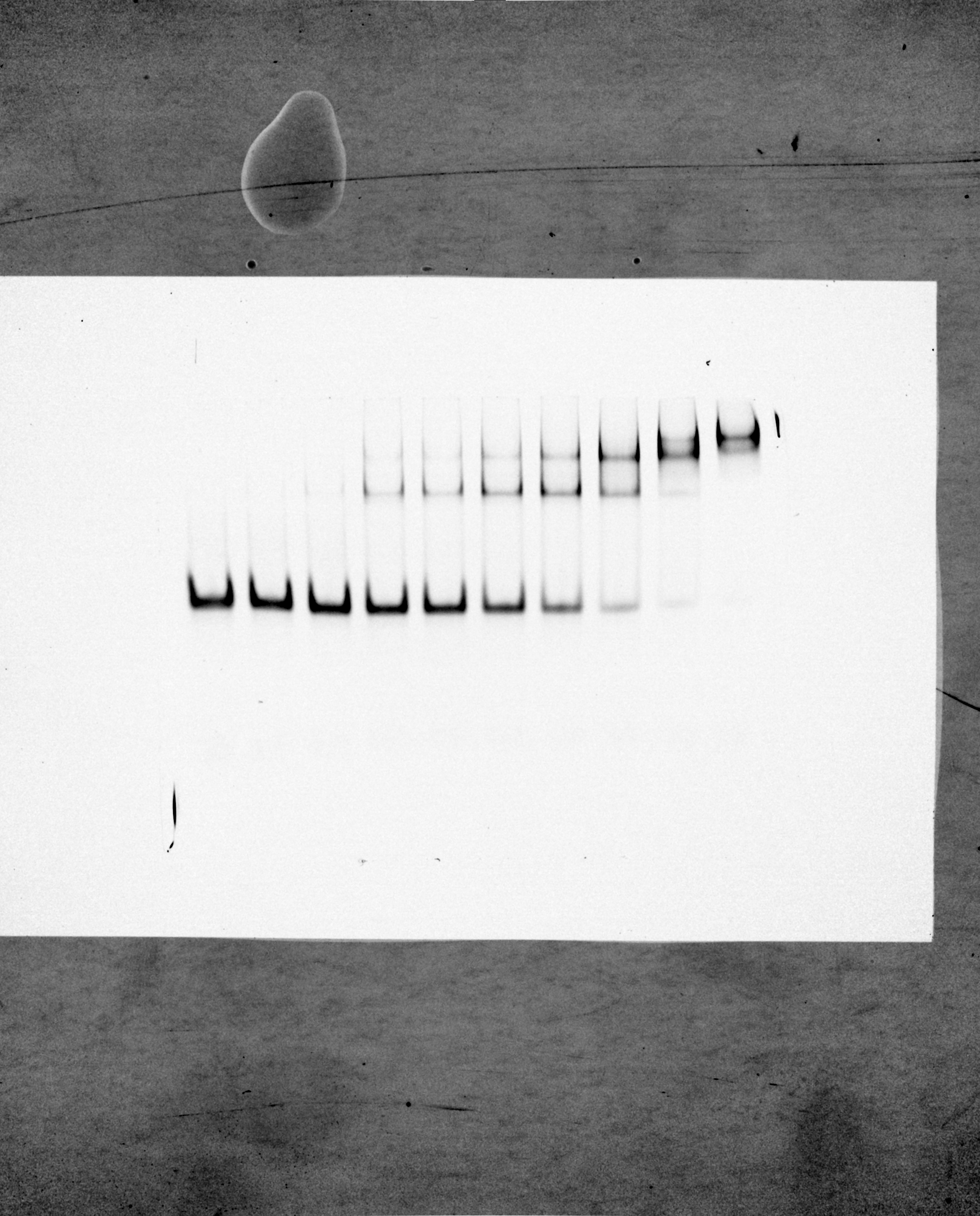

Supplement: Figure 1—source data 1. — Electrophoretic mobility shift assay (EMSA) images (panels b–e) and data analyses (panel j). [file elife-83538-fig1-data1.zip › Figure 1 - Source data 1/e/211001 Cy5 80bp EMSA with yCAF1 WT_PUB_600.tif]

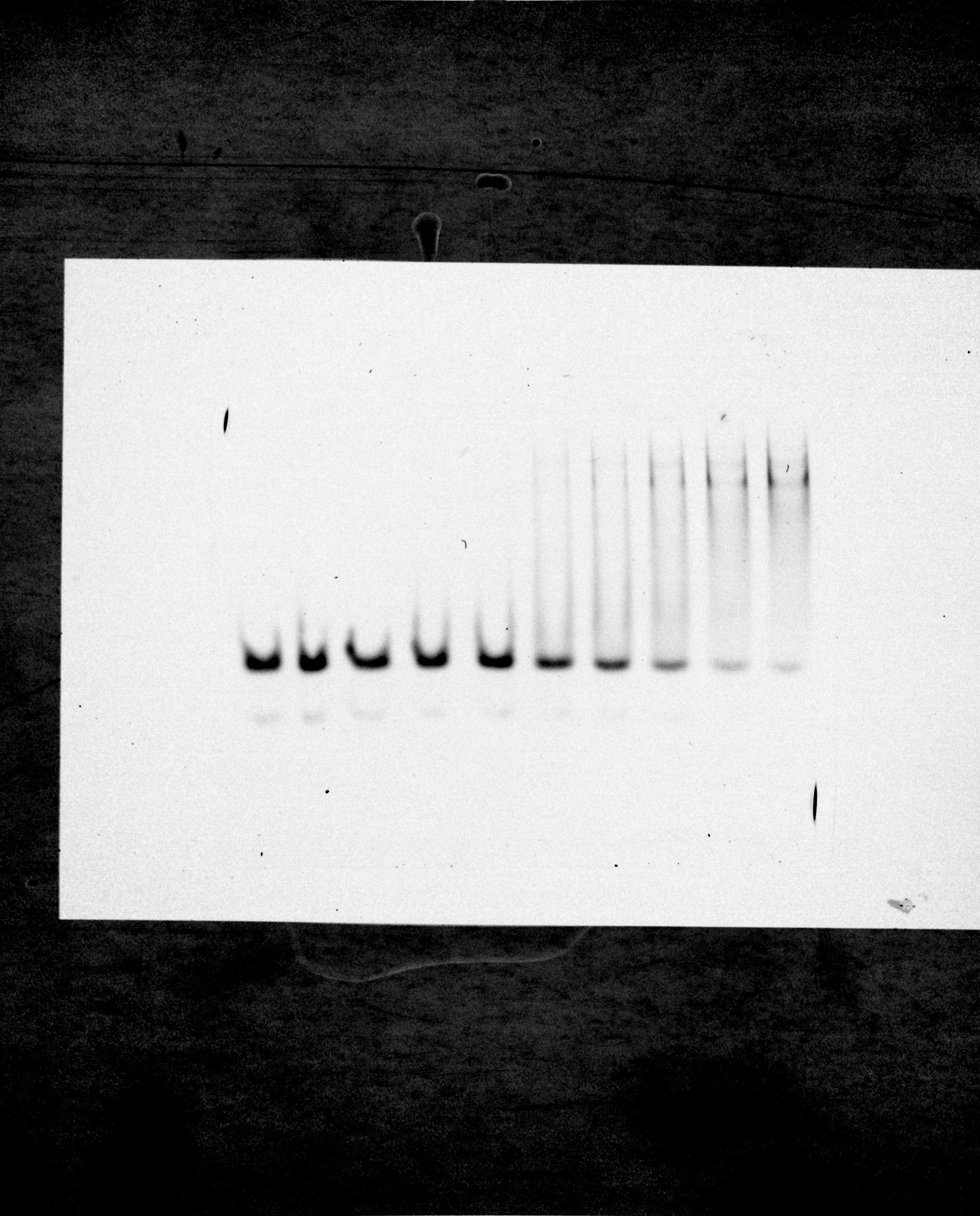

Supplement: Figure 1—source data 1. — Electrophoretic mobility shift assay (EMSA) images (panels b–e) and data analyses (panel j). [file elife-83538-fig1-data1.zip › Figure 1 - Source data 1/b/211001 Cy5 30bp EMSA with yCAF1 WT_PUB_600.tif]

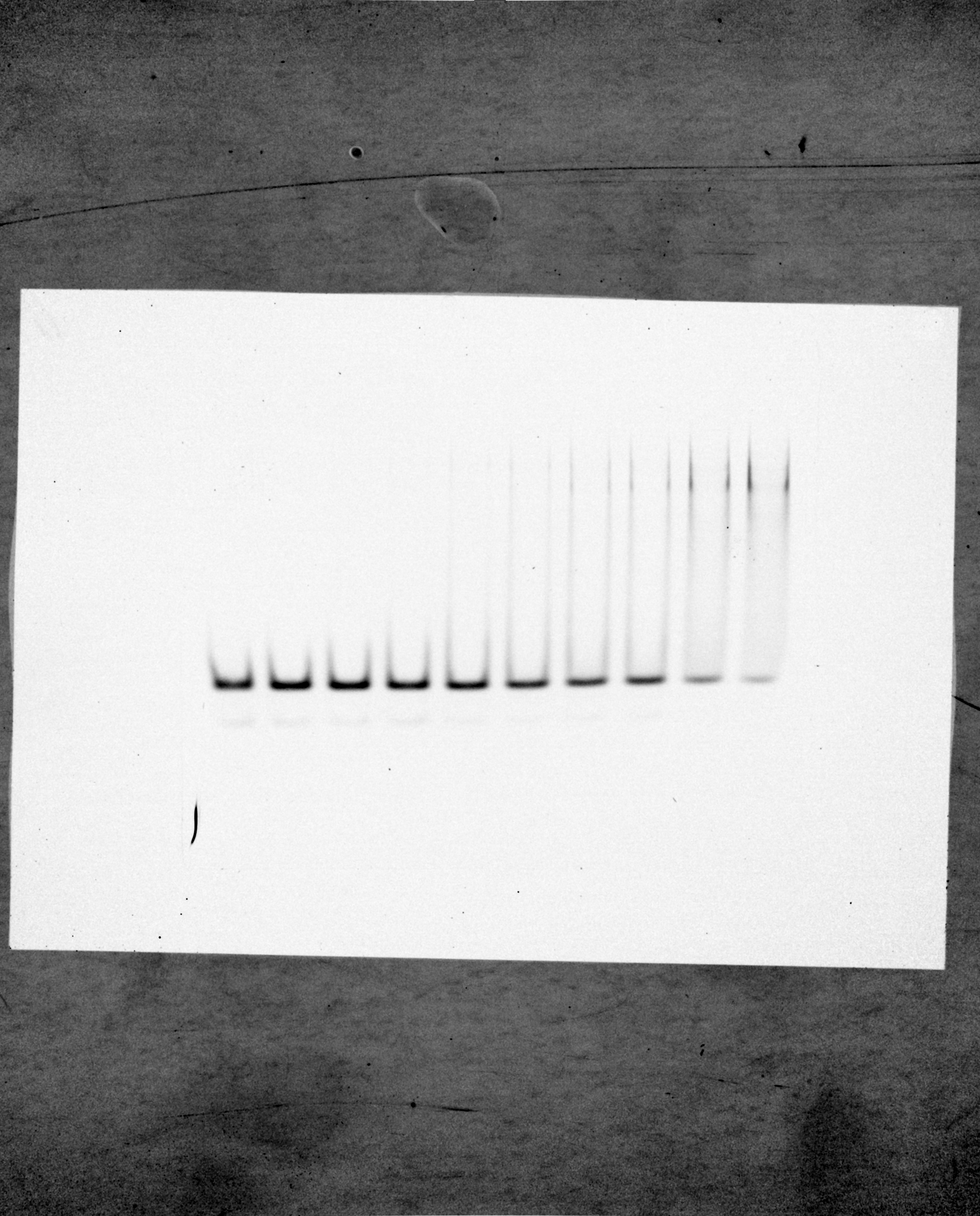

Supplement: Figure 1—source data 1. — Electrophoretic mobility shift assay (EMSA) images (panels b–e) and data analyses (panel j). [file elife-83538-fig1-data1.zip › Figure 1 - Source data 1/b/211004 Cy5 30 bp EMSA with yCAF1 WT_PUB_600.tif]

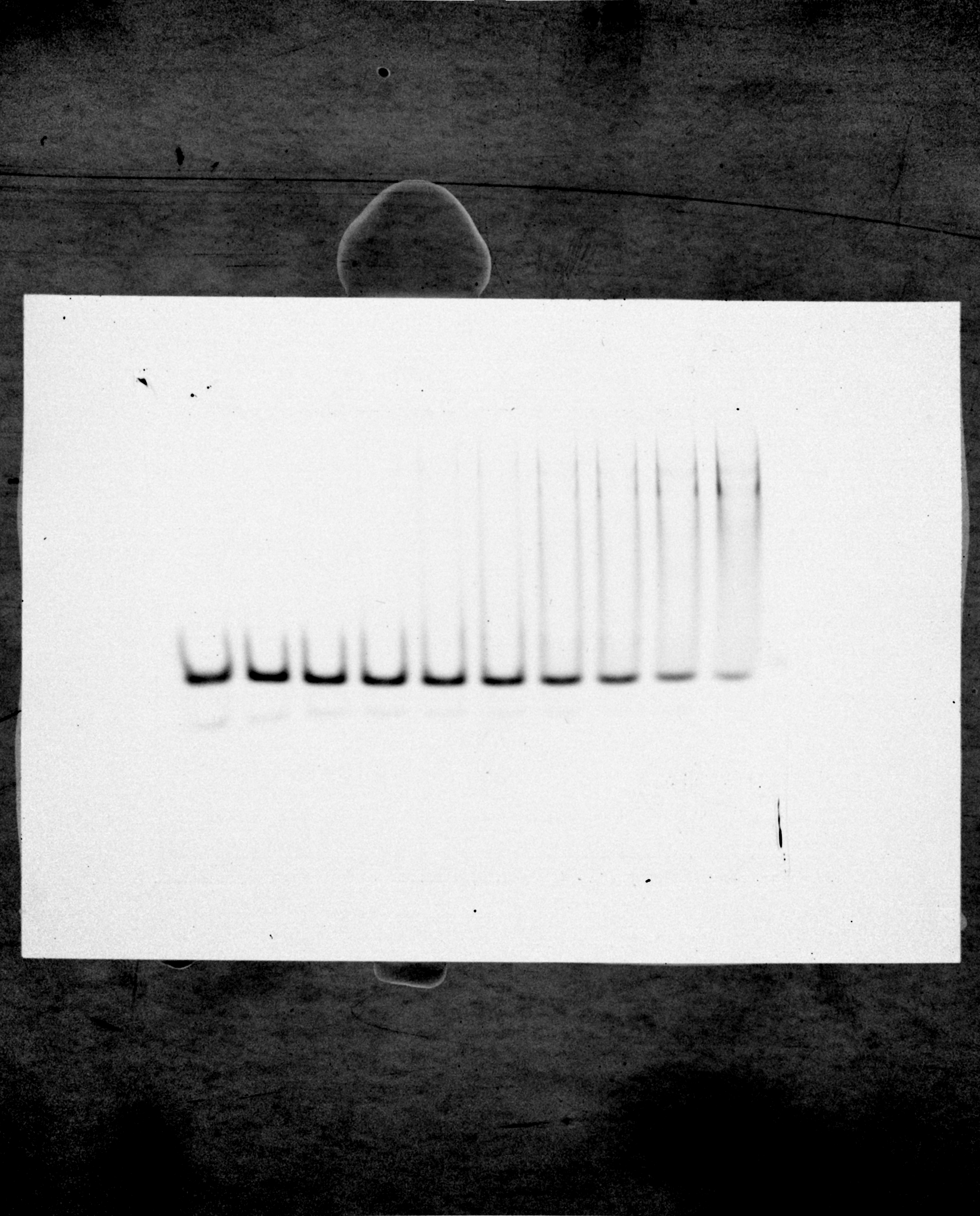

Supplement: Figure 1—source data 1. — Electrophoretic mobility shift assay (EMSA) images (panels b–e) and data analyses (panel j). [file elife-83538-fig1-data1.zip › Figure 1 - Source data 1/b/210921 Cy5 30bp EMSA with yCAF1 WT_PUB_600.tif]

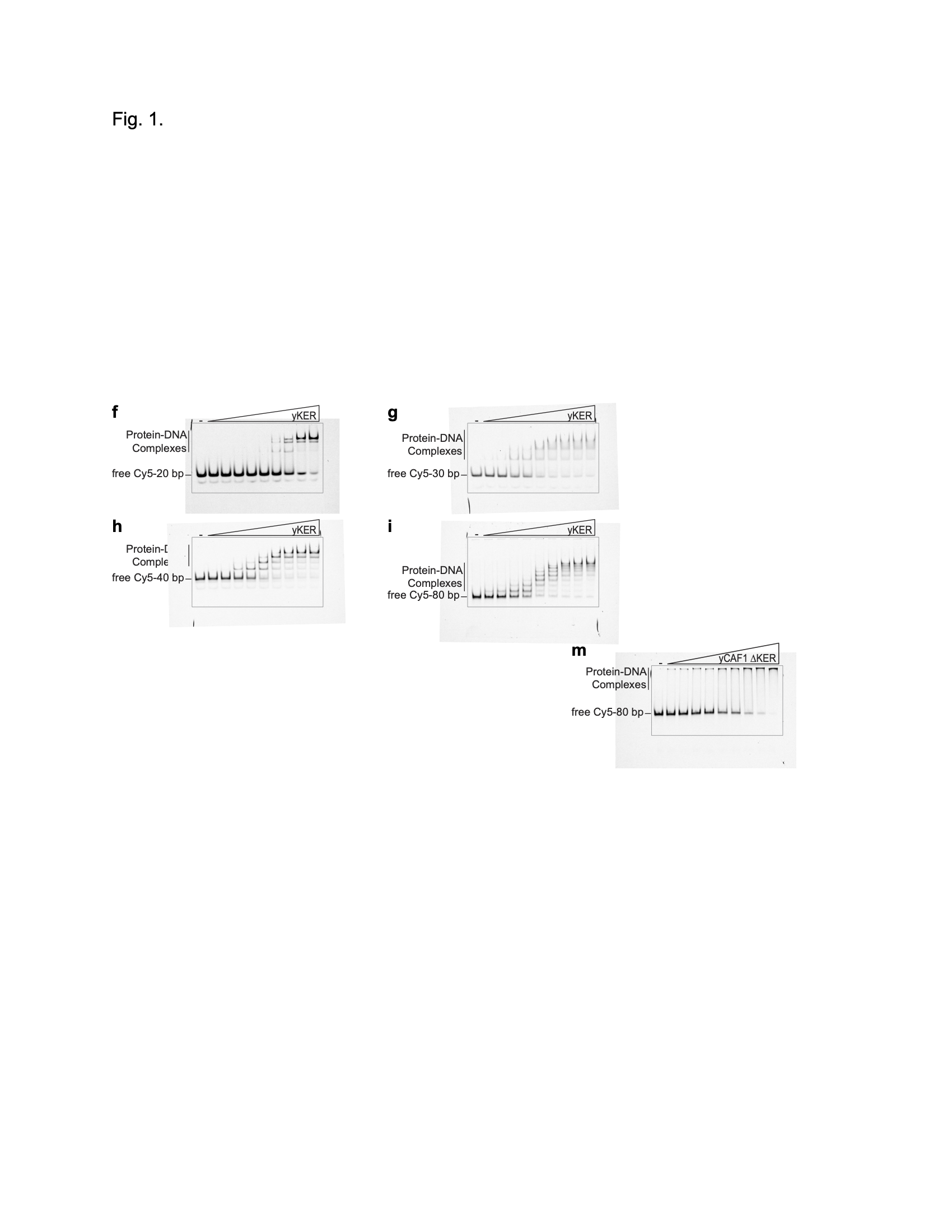

Supplement: Figure 1—source data 2. — Electrophoretic mobility shift assay (EMSA) images (panels f–i and m), data analyses (panels k and l), and flow cytometry data (panel o). [file elife-83538-fig1-data2.zip › Figure 1 - Source data 2/Figure 1 - Source data 2_Gels Labeled.png]

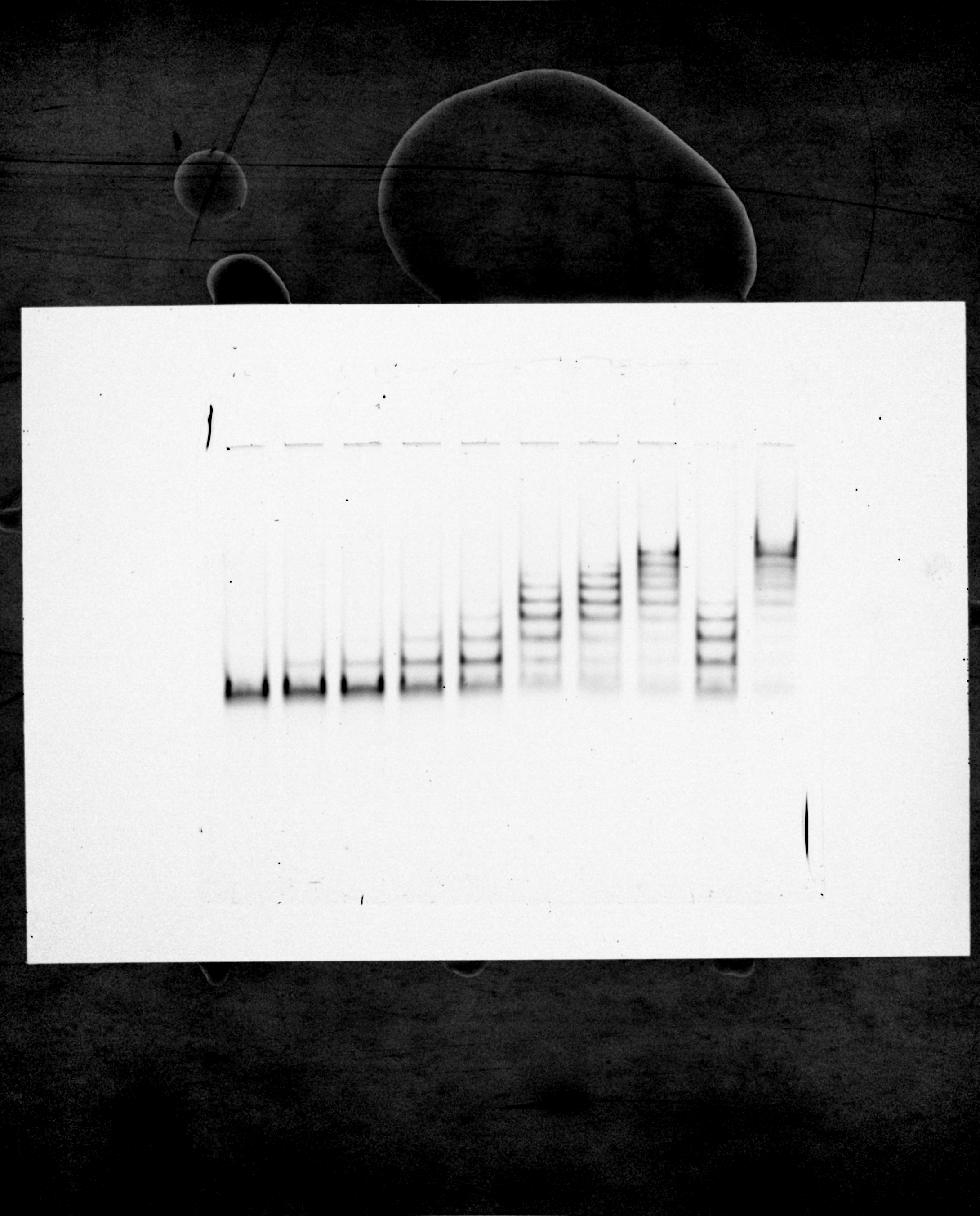

Supplement: Figure 1—source data 2. — Electrophoretic mobility shift assay (EMSA) images (panels f–i and m), data analyses (panels k and l), and flow cytometry data (panel o). [file elife-83538-fig1-data2.zip › Figure 1 - Source data 2/i/220324 Cy5 80bp EMSA with yKER 136to225Y_n3_PUB_600.tif]

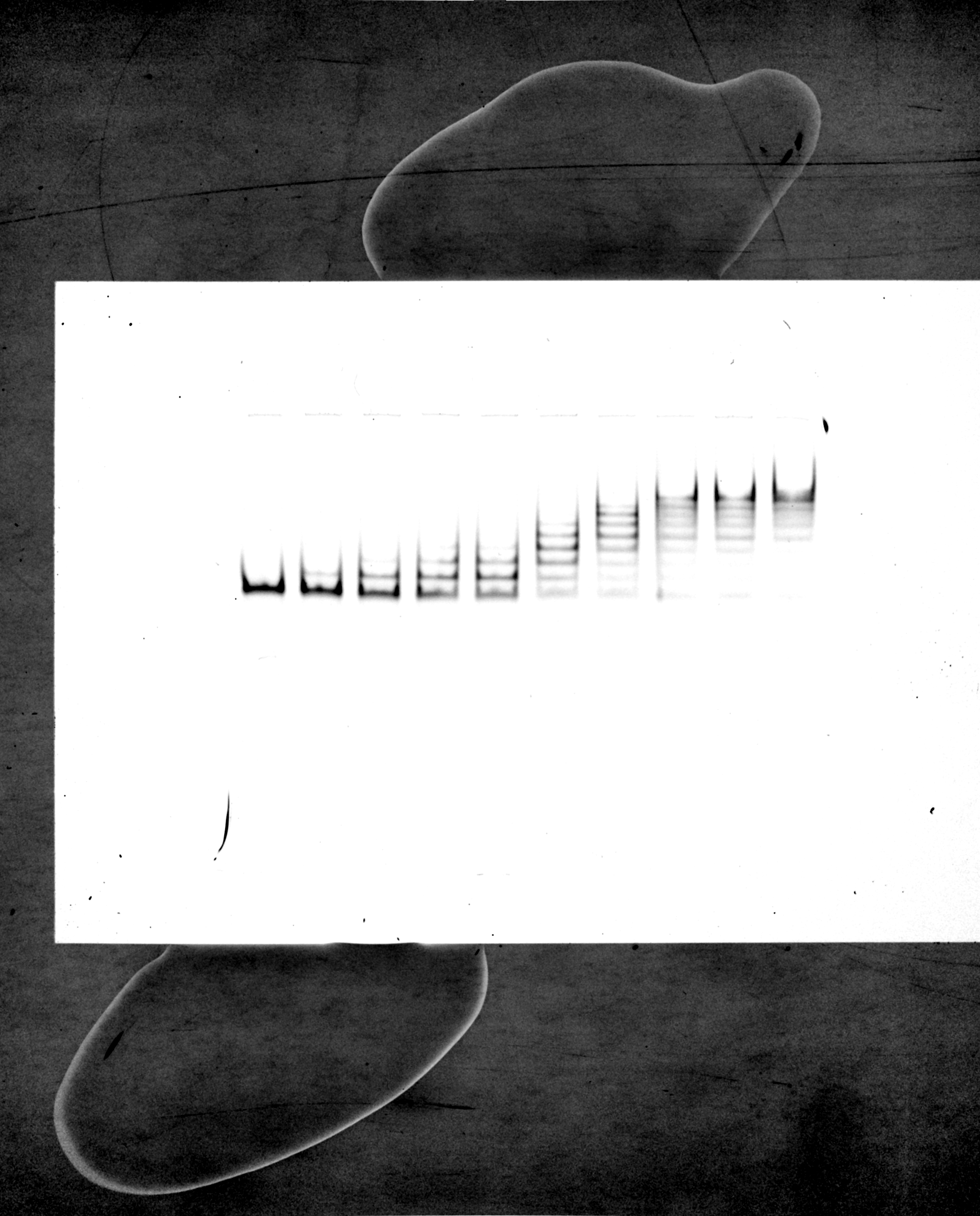

Supplement: Figure 1—source data 2. — Electrophoretic mobility shift assay (EMSA) images (panels f–i and m), data analyses (panels k and l), and flow cytometry data (panel o). [file elife-83538-fig1-data2.zip › Figure 1 - Source data 2/i/220324 Cy5 80bp EMSA with yKER 136to225Y_n1_PUB_600.tif]

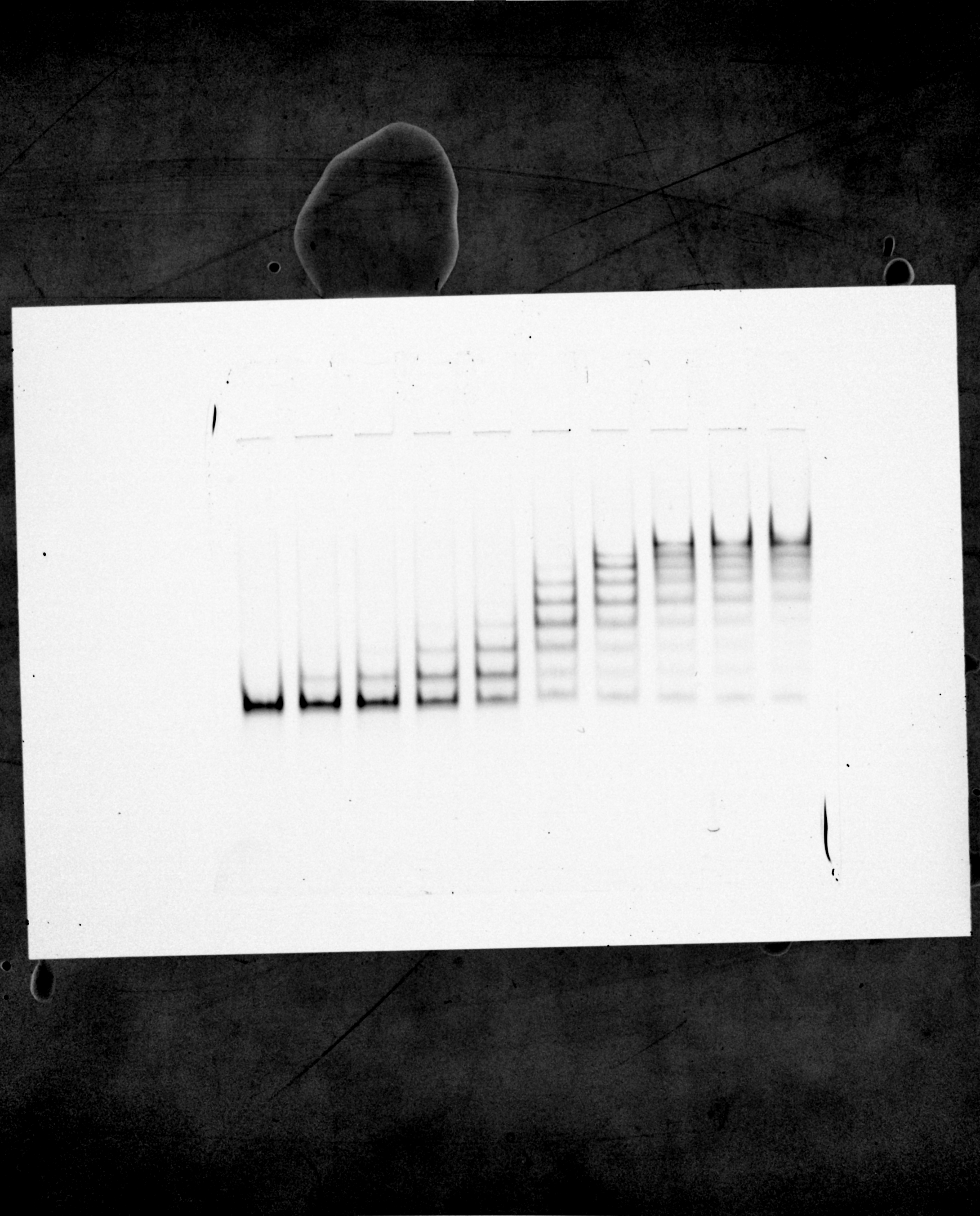

Supplement: Figure 1—source data 2. — Electrophoretic mobility shift assay (EMSA) images (panels f–i and m), data analyses (panels k and l), and flow cytometry data (panel o). [file elife-83538-fig1-data2.zip › Figure 1 - Source data 2/i/220324 Cy5 80bp EMSA with yKER 136to225Y_n2_PUB_600.tif]

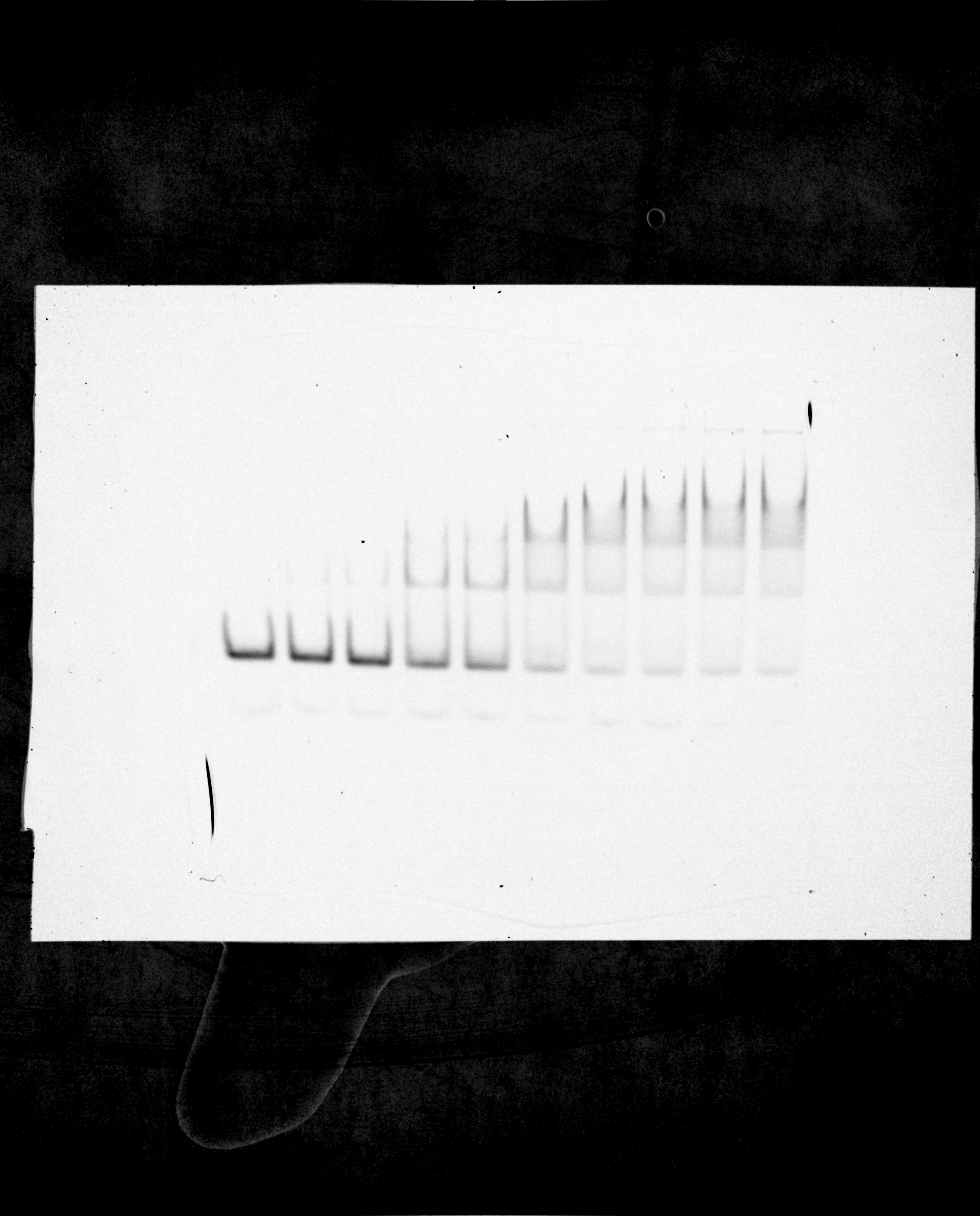

Supplement: Figure 1—source data 2. — Electrophoretic mobility shift assay (EMSA) images (panels f–i and m), data analyses (panels k and l), and flow cytometry data (panel o). [file elife-83538-fig1-data2.zip › Figure 1 - Source data 2/g/211122 Cy5 30 bp EMSA with yKER_PUB_600.tif]

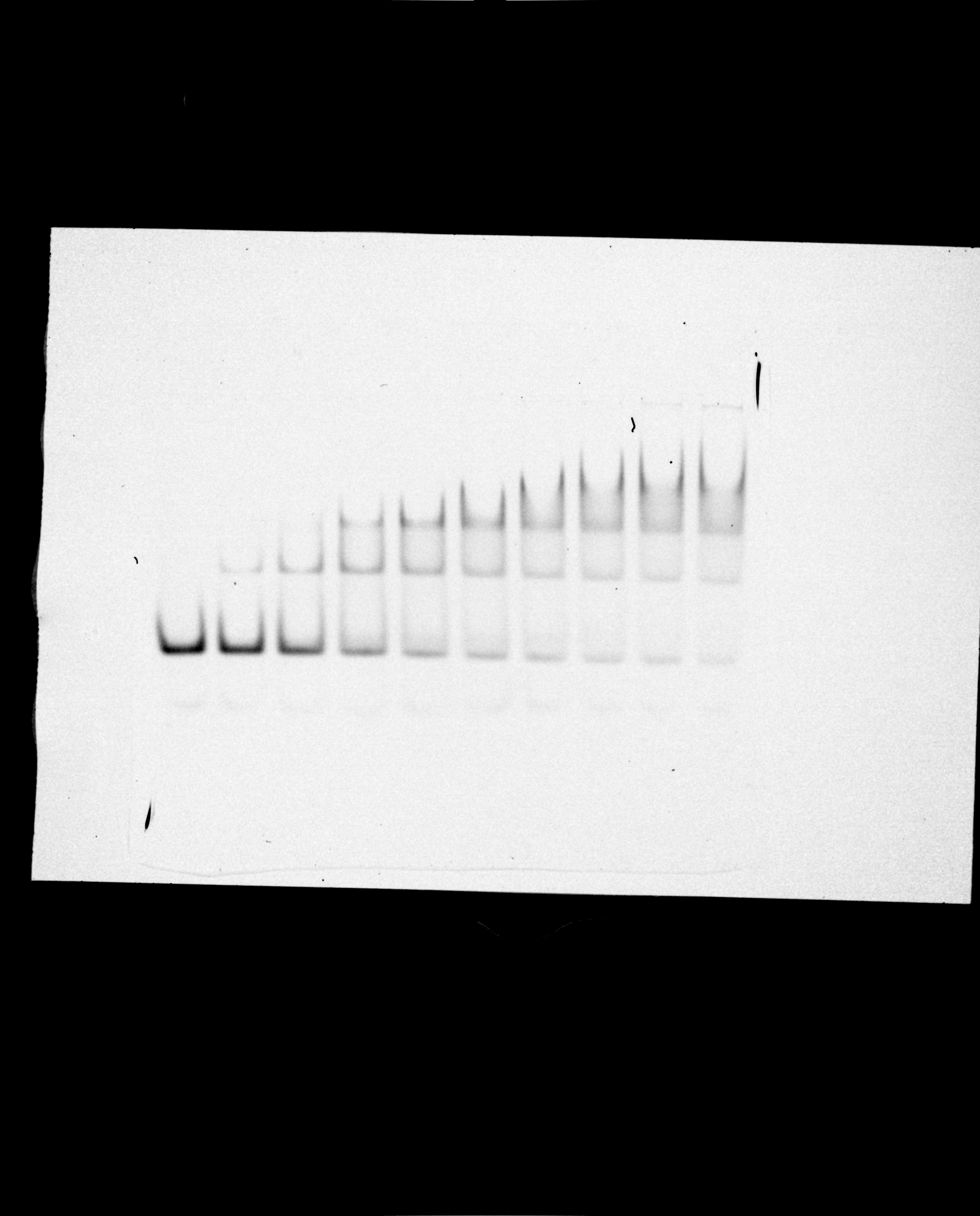

Supplement: Figure 1—source data 2. — Electrophoretic mobility shift assay (EMSA) images (panels f–i and m), data analyses (panels k and l), and flow cytometry data (panel o). [file elife-83538-fig1-data2.zip › Figure 1 - Source data 2/g/211119 Cy5 30 bp EMSA with yKER_PUB_600.tif]

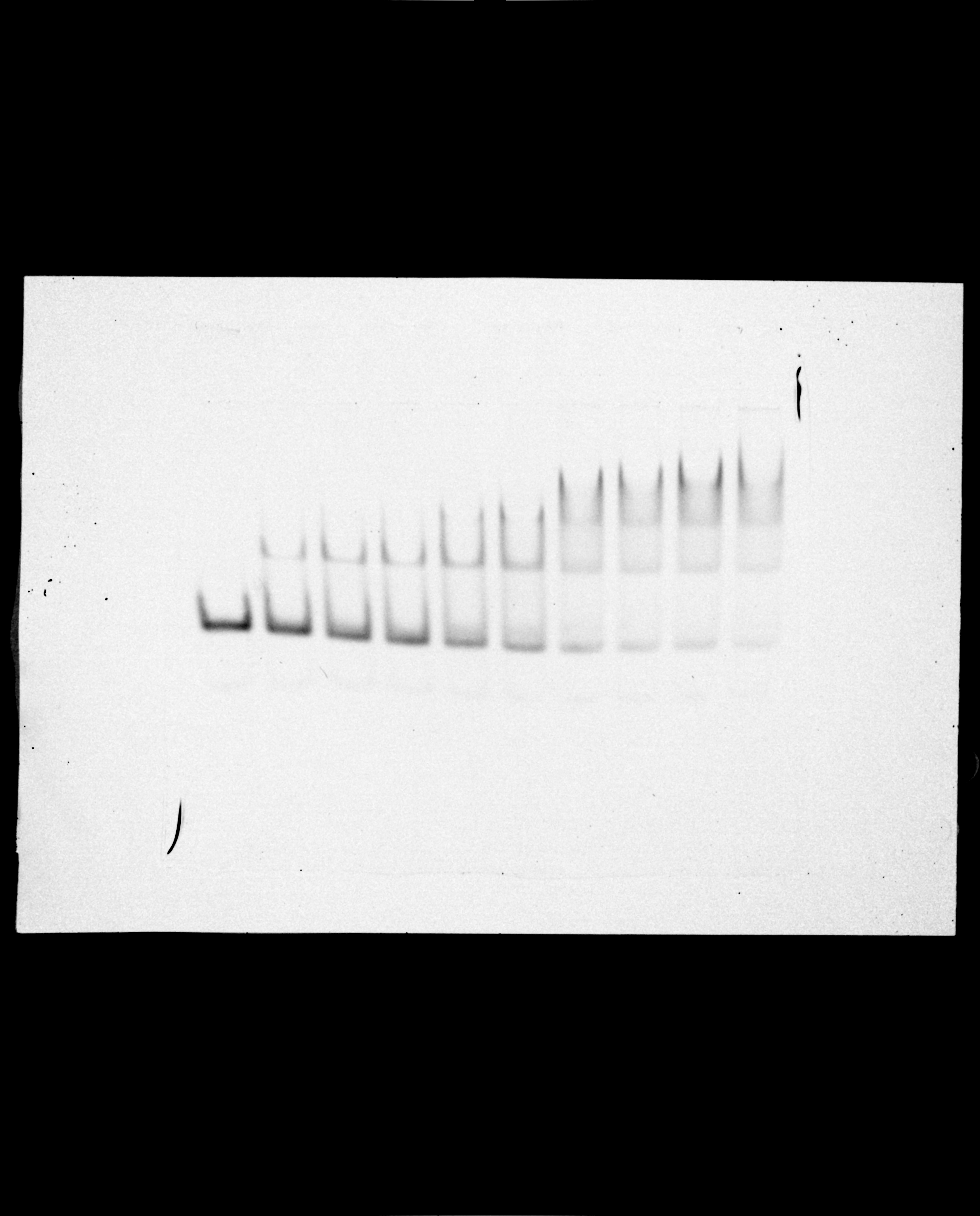

Supplement: Figure 1—source data 2. — Electrophoretic mobility shift assay (EMSA) images (panels f–i and m), data analyses (panels k and l), and flow cytometry data (panel o). [file elife-83538-fig1-data2.zip › Figure 1 - Source data 2/g/211118 Cy5 30 bp EMSA with yKER_n2_PUB_600.tif]

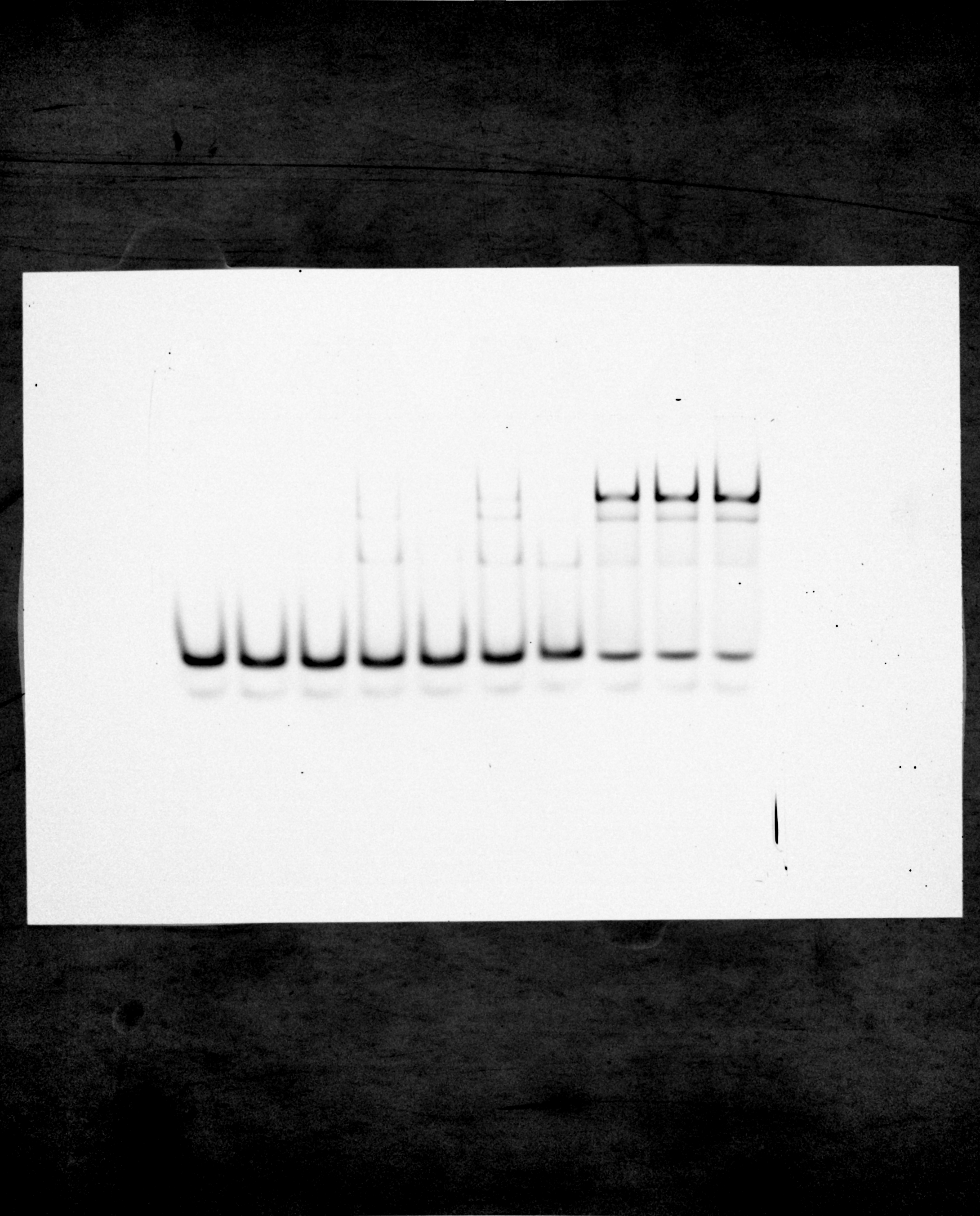

Supplement: Figure 1—source data 2. — Electrophoretic mobility shift assay (EMSA) images (panels f–i and m), data analyses (panels k and l), and flow cytometry data (panel o). [file elife-83538-fig1-data2.zip › Figure 1 - Source data 2/f/211216 Cy5 20bp EMSA with yKER_n2_PUB_600.tif]

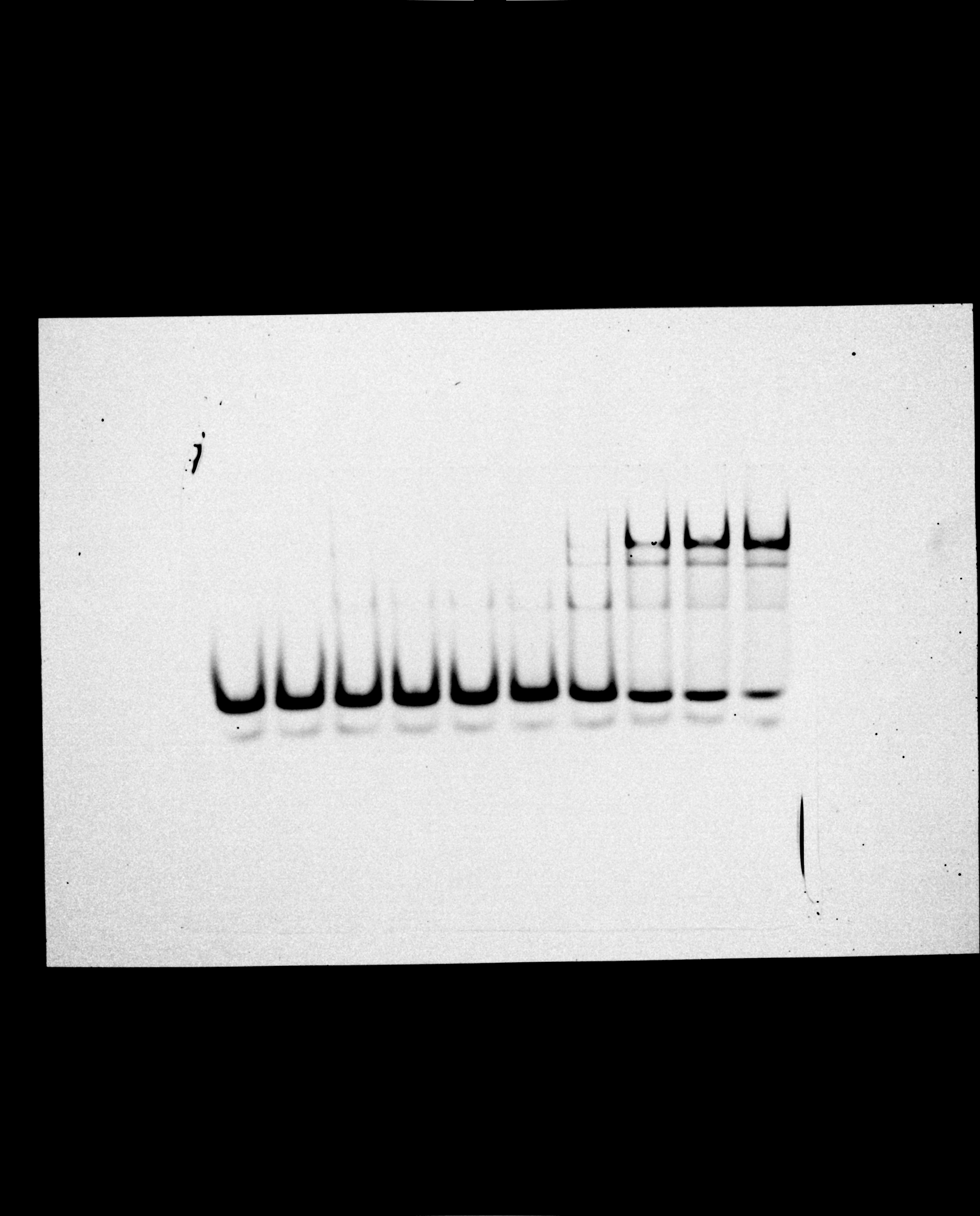

Supplement: Figure 1—source data 2. — Electrophoretic mobility shift assay (EMSA) images (panels f–i and m), data analyses (panels k and l), and flow cytometry data (panel o). [file elife-83538-fig1-data2.zip › Figure 1 - Source data 2/f/211216 Cy5 20bp EMSA with yKER_n1_PUB_600.tif]

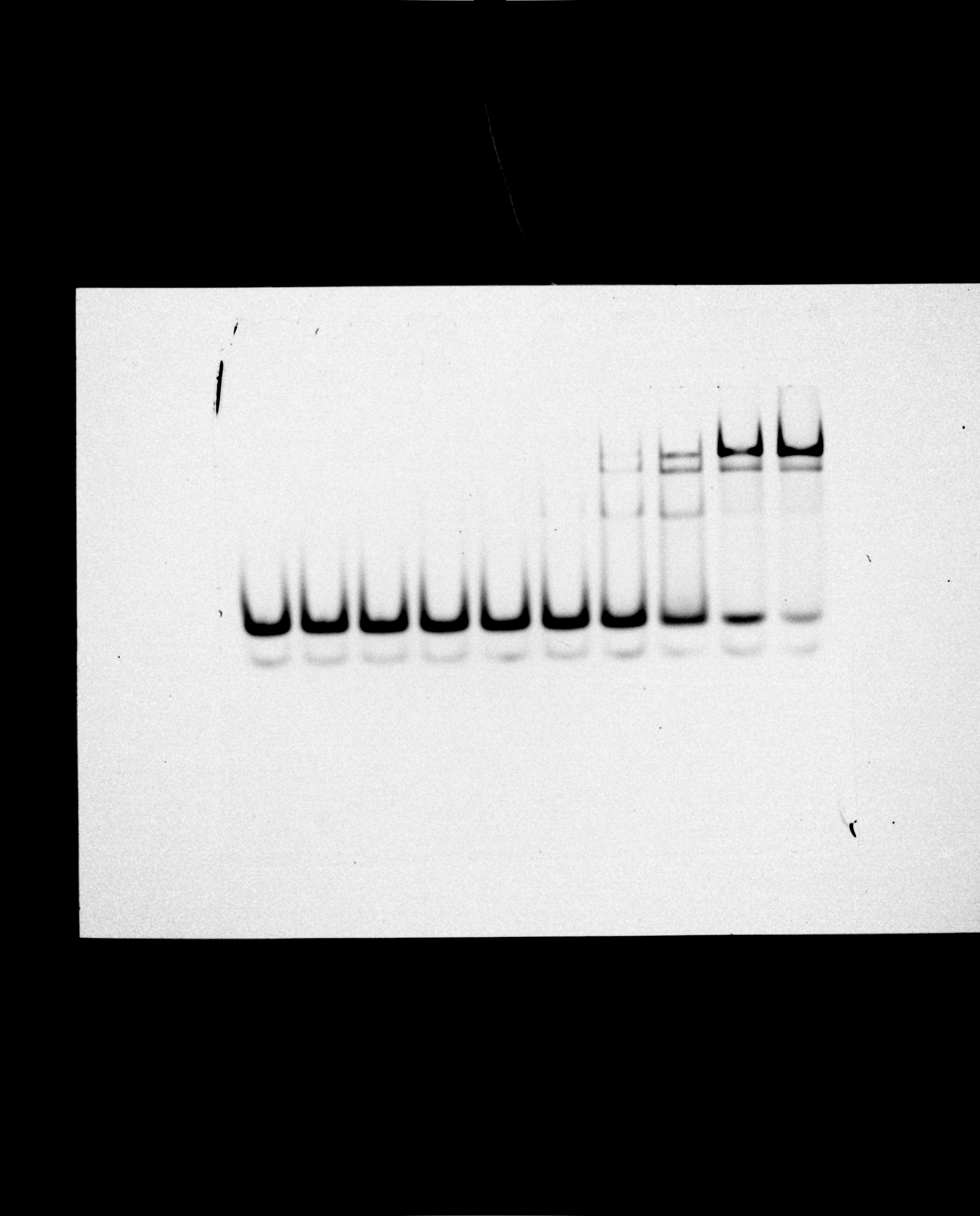

Supplement: Figure 1—source data 2. — Electrophoretic mobility shift assay (EMSA) images (panels f–i and m), data analyses (panels k and l), and flow cytometry data (panel o). [file elife-83538-fig1-data2.zip › Figure 1 - Source data 2/f/211220 Cy5 20 bp EMSA with yKER_PUB_600.tif]

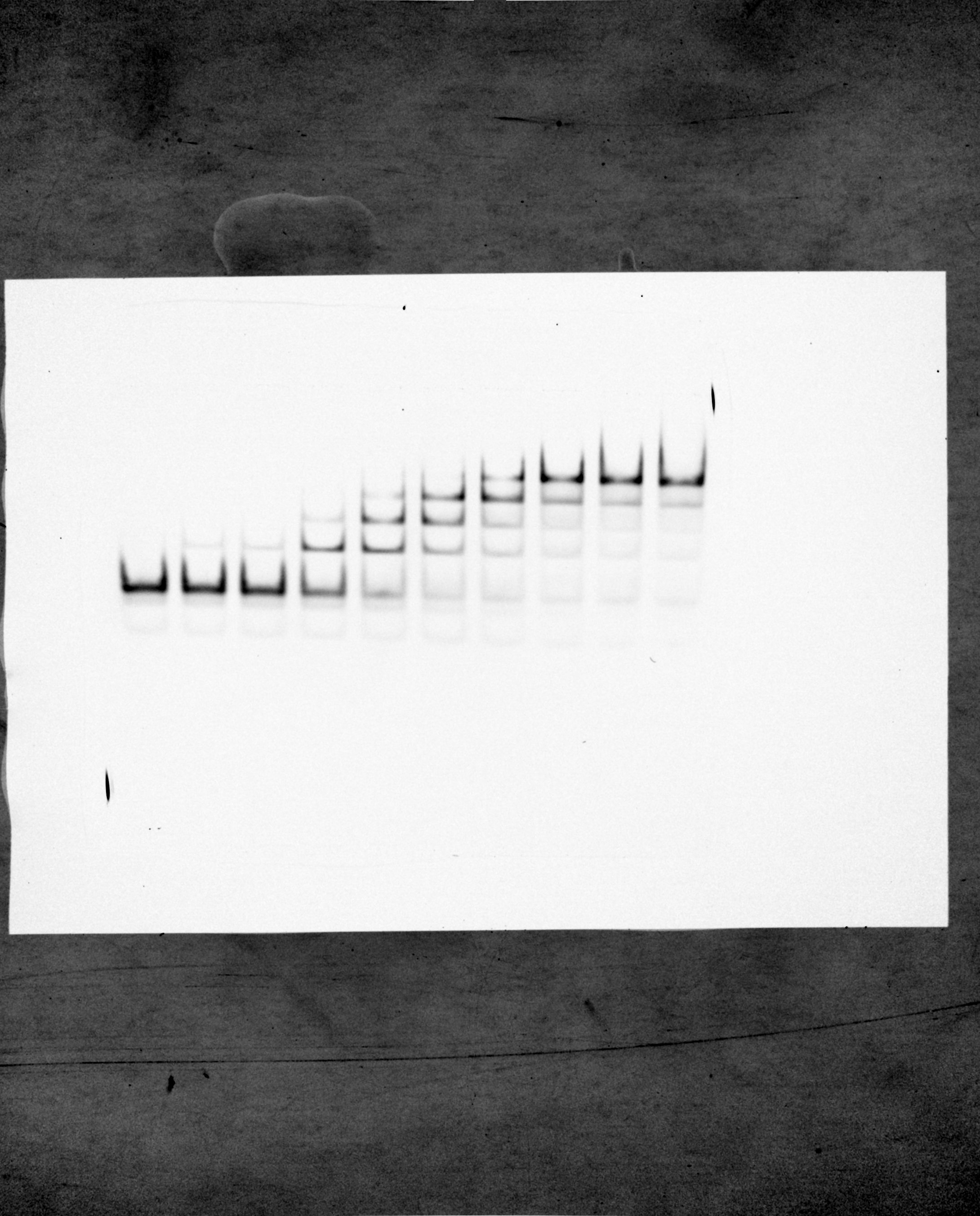

Supplement: Figure 1—source data 2. — Electrophoretic mobility shift assay (EMSA) images (panels f–i and m), data analyses (panels k and l), and flow cytometry data (panel o). [file elife-83538-fig1-data2.zip › Figure 1 - Source data 2/h/211119 Cy5 40 bp EMSA with yKER_PUB_600.tif]

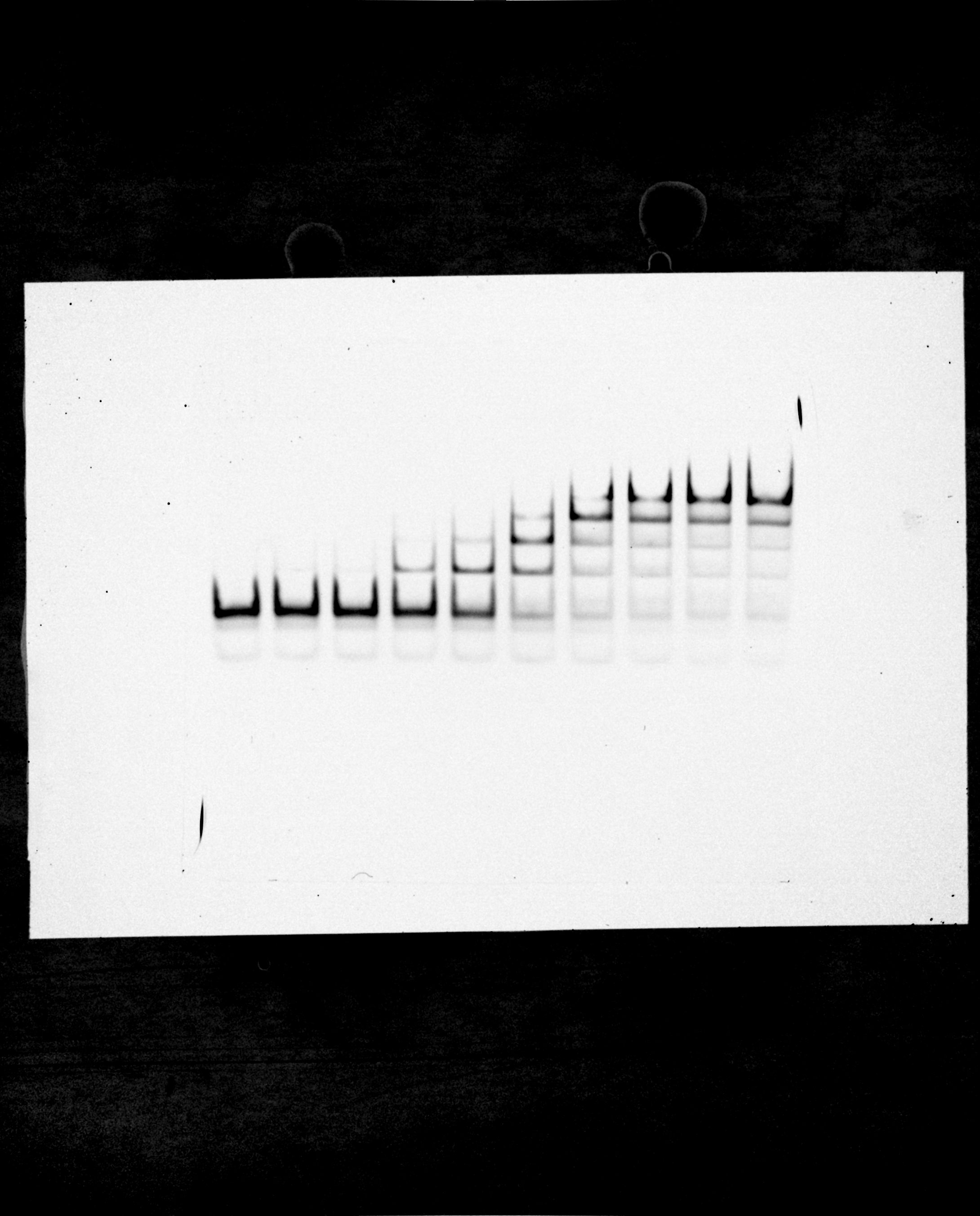

Supplement: Figure 1—source data 2. — Electrophoretic mobility shift assay (EMSA) images (panels f–i and m), data analyses (panels k and l), and flow cytometry data (panel o). [file elife-83538-fig1-data2.zip › Figure 1 - Source data 2/h/211122 Cy5 40 bp EMSA with yKER_PUB_600.tif]

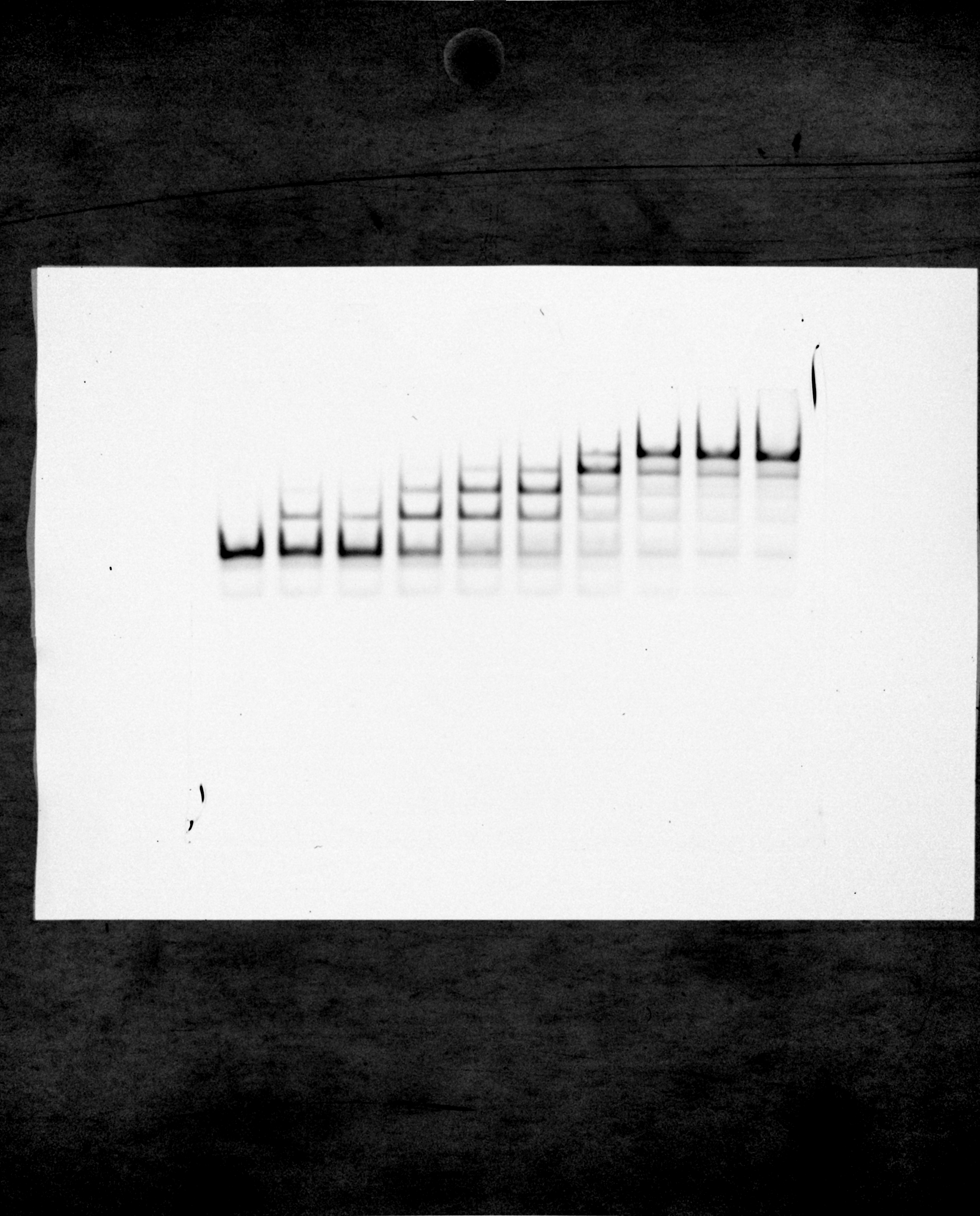

Supplement: Figure 1—source data 2. — Electrophoretic mobility shift assay (EMSA) images (panels f–i and m), data analyses (panels k and l), and flow cytometry data (panel o). [file elife-83538-fig1-data2.zip › Figure 1 - Source data 2/h/211118 Cy5 40 bp EMSA with yKER_n1_PUB_600.tif]

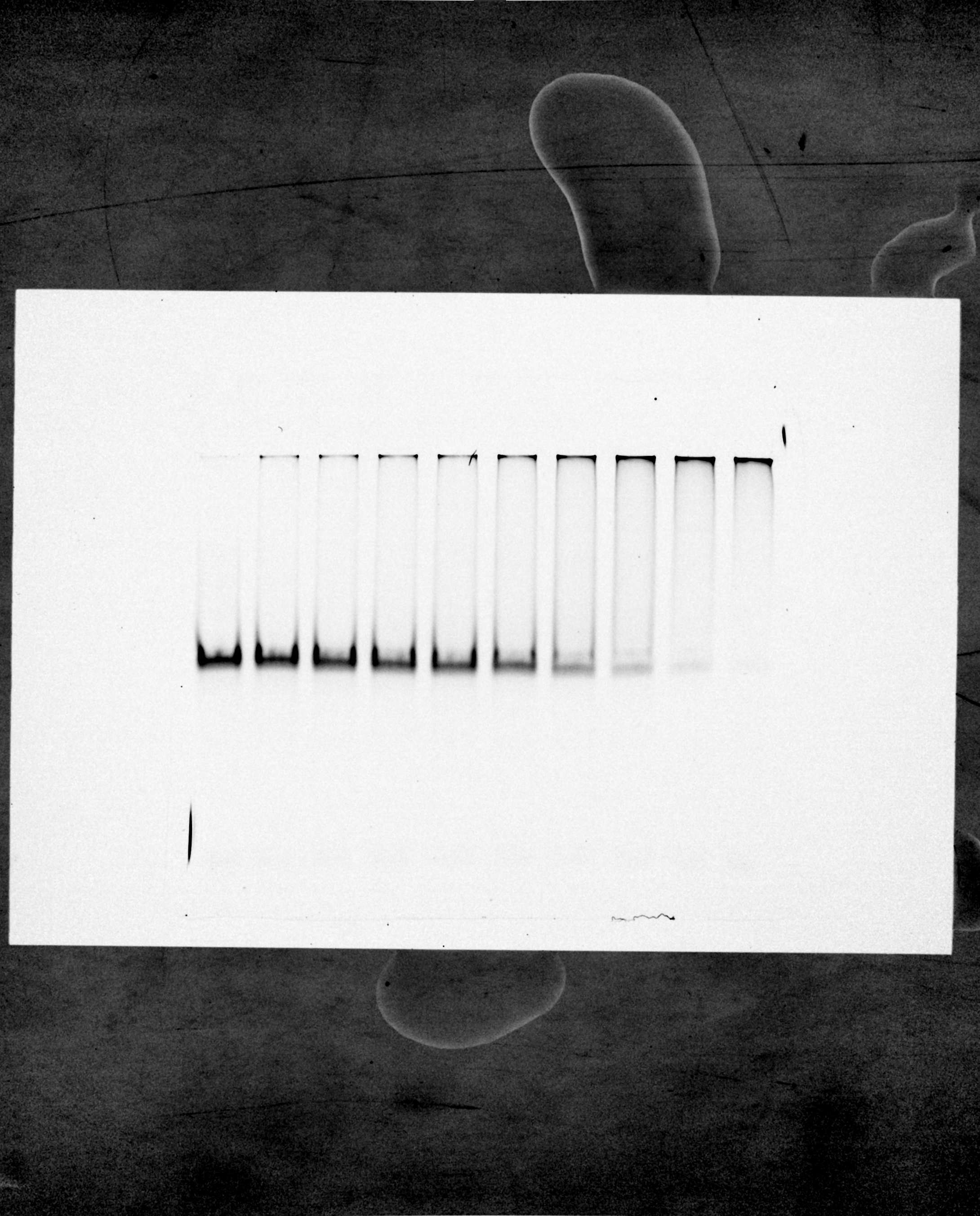

Supplement: Figure 1—source data 2. — Electrophoretic mobility shift assay (EMSA) images (panels f–i and m), data analyses (panels k and l), and flow cytometry data (panel o). [file elife-83538-fig1-data2.zip › Figure 1 - Source data 2/m/220325 Cy5 80bp EMSA with yCAF1 deltaKER_n3_PUB_600.tif]

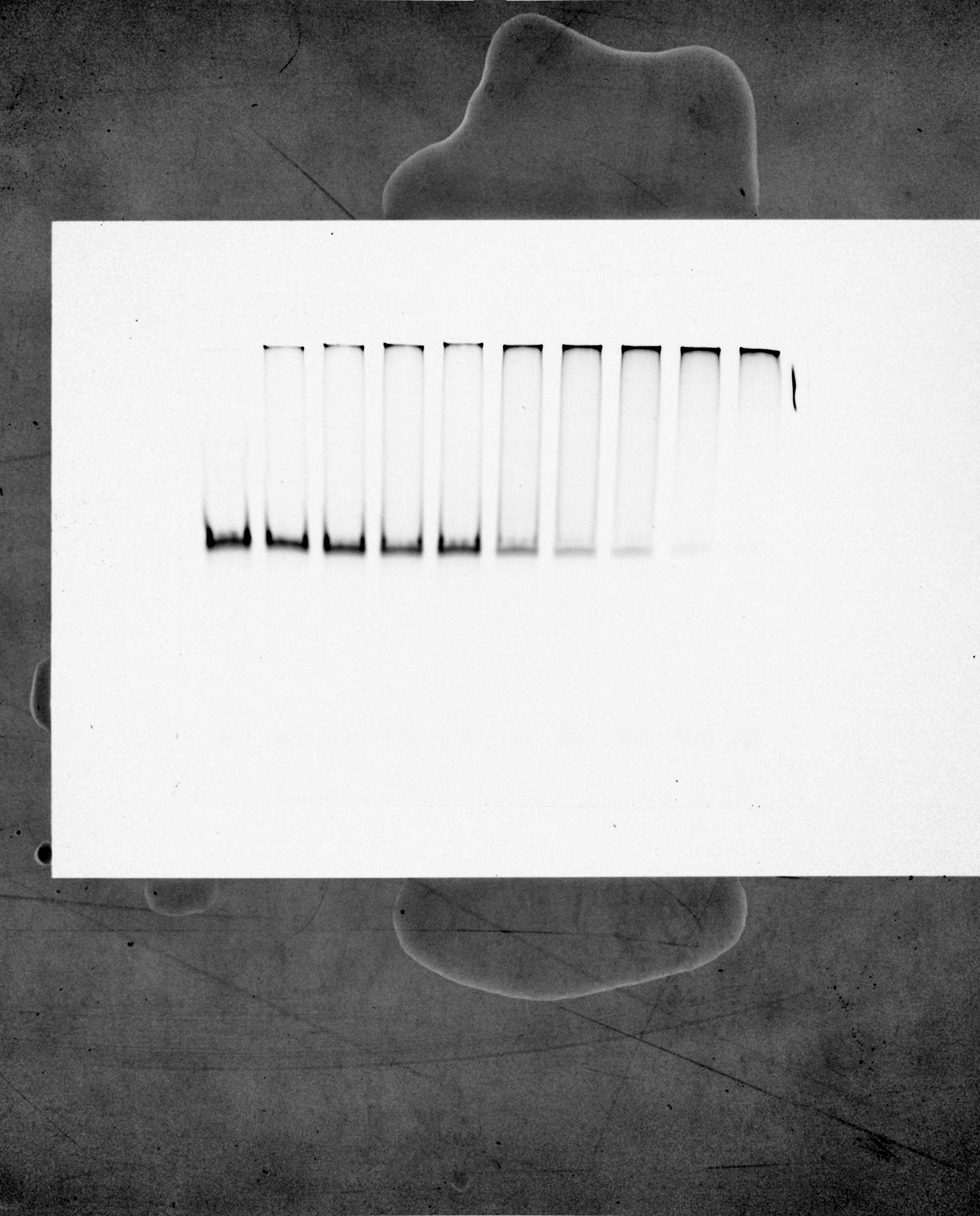

Supplement: Figure 1—source data 2. — Electrophoretic mobility shift assay (EMSA) images (panels f–i and m), data analyses (panels k and l), and flow cytometry data (panel o). [file elife-83538-fig1-data2.zip › Figure 1 - Source data 2/m/220325 Cy5 80bp EMSA with yCAF1 deltaKER_n1_PUB_600.tif]

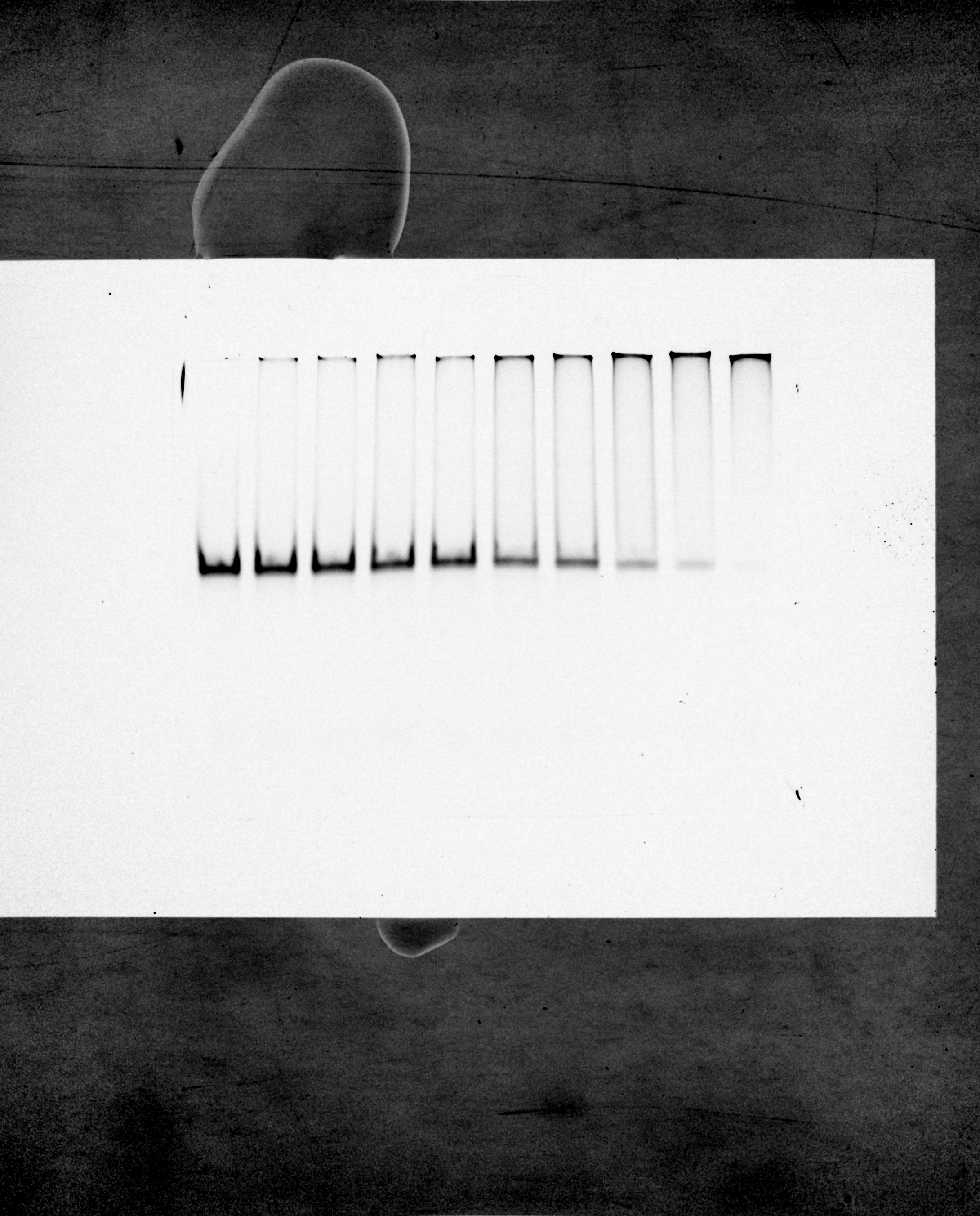

Supplement: Figure 1—source data 2. — Electrophoretic mobility shift assay (EMSA) images (panels f–i and m), data analyses (panels k and l), and flow cytometry data (panel o). [file elife-83538-fig1-data2.zip › Figure 1 - Source data 2/m/220325 Cy5 80bp EMSA with yCAF1 deltaKER_n2_PUB_600.tif]

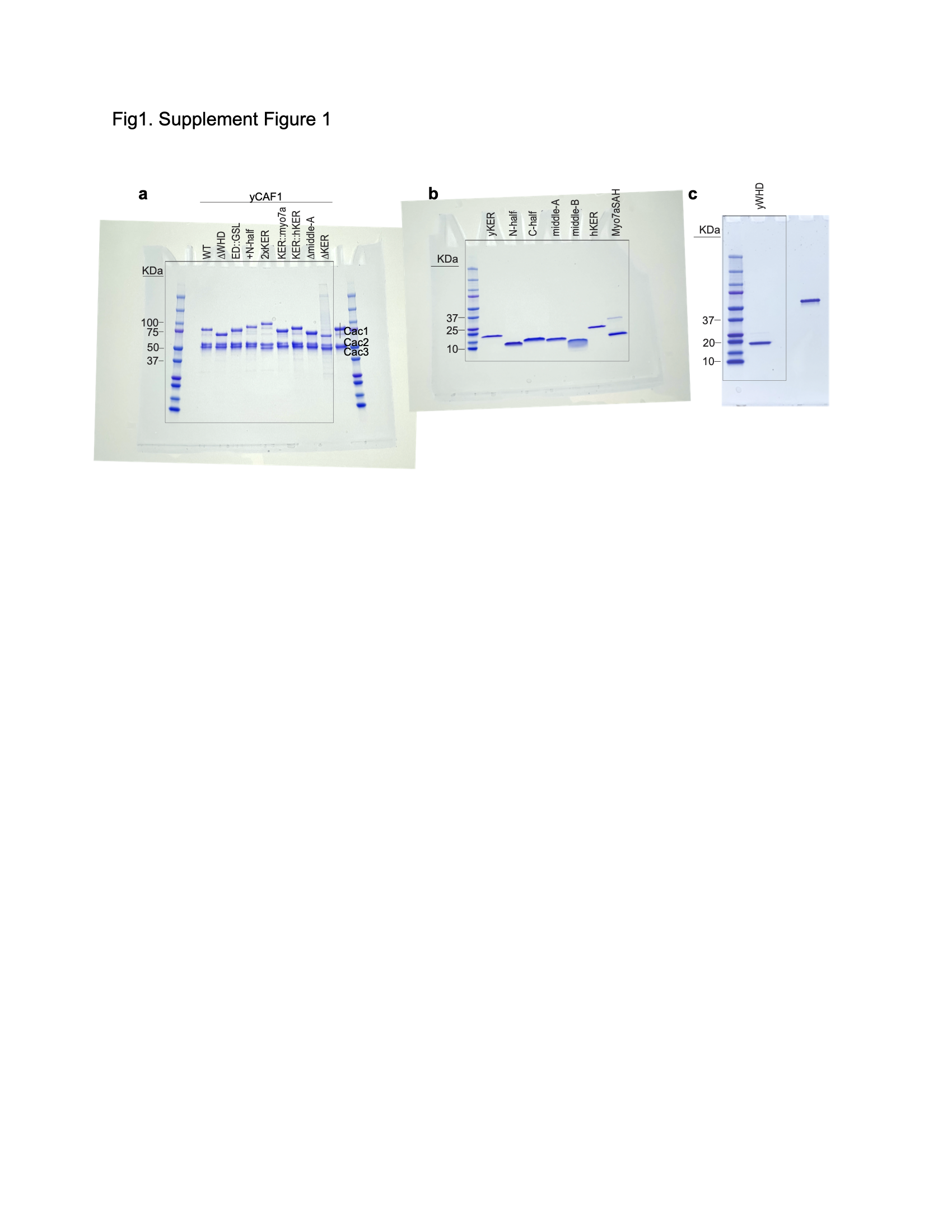

Supplement: Figure 1—figure supplement 2—source data 1. — Sodium dodecyl sulfate–polyacrylamide gel electrophoresis (SDS–PAGE) (panels a–c) and western blots (panel d). [file elife-83538-fig1-figsupp2-data1.zip › Figure 1 - figure supplement 2 - Source data 1/Figure 1 -figure supplement 2 Source data 1_Gels Labeled_a-c.png]

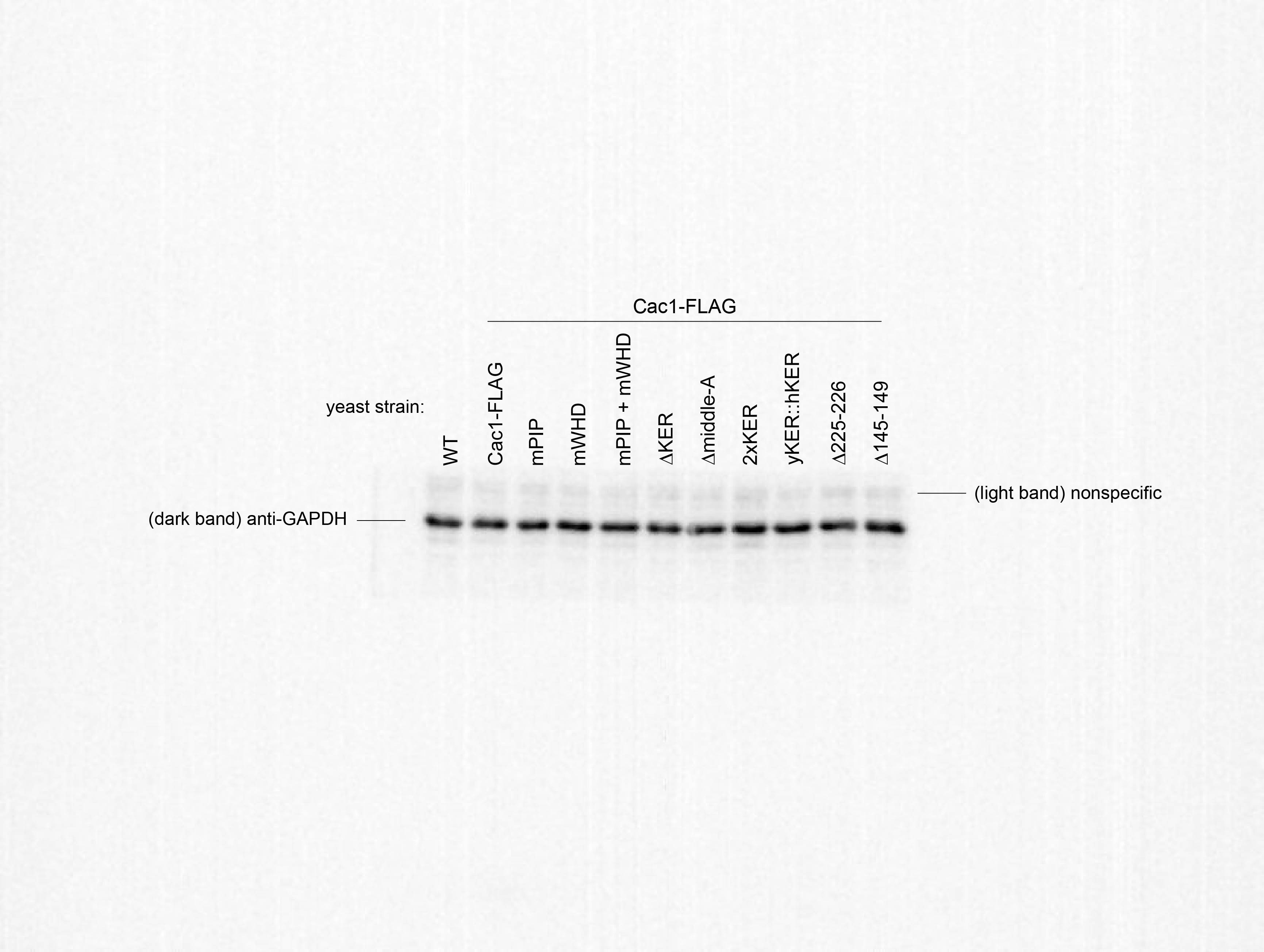

Supplement: Figure 1—figure supplement 2—source data 1. — Sodium dodecyl sulfate–polyacrylamide gel electrophoresis (SDS–PAGE) (panels a–c) and western blots (panel d). [file elife-83538-fig1-figsupp2-data1.zip › Figure 1 - figure supplement 2 - Source data 1/Figure S1d GAPDH Gel 1 labeled.jpg]

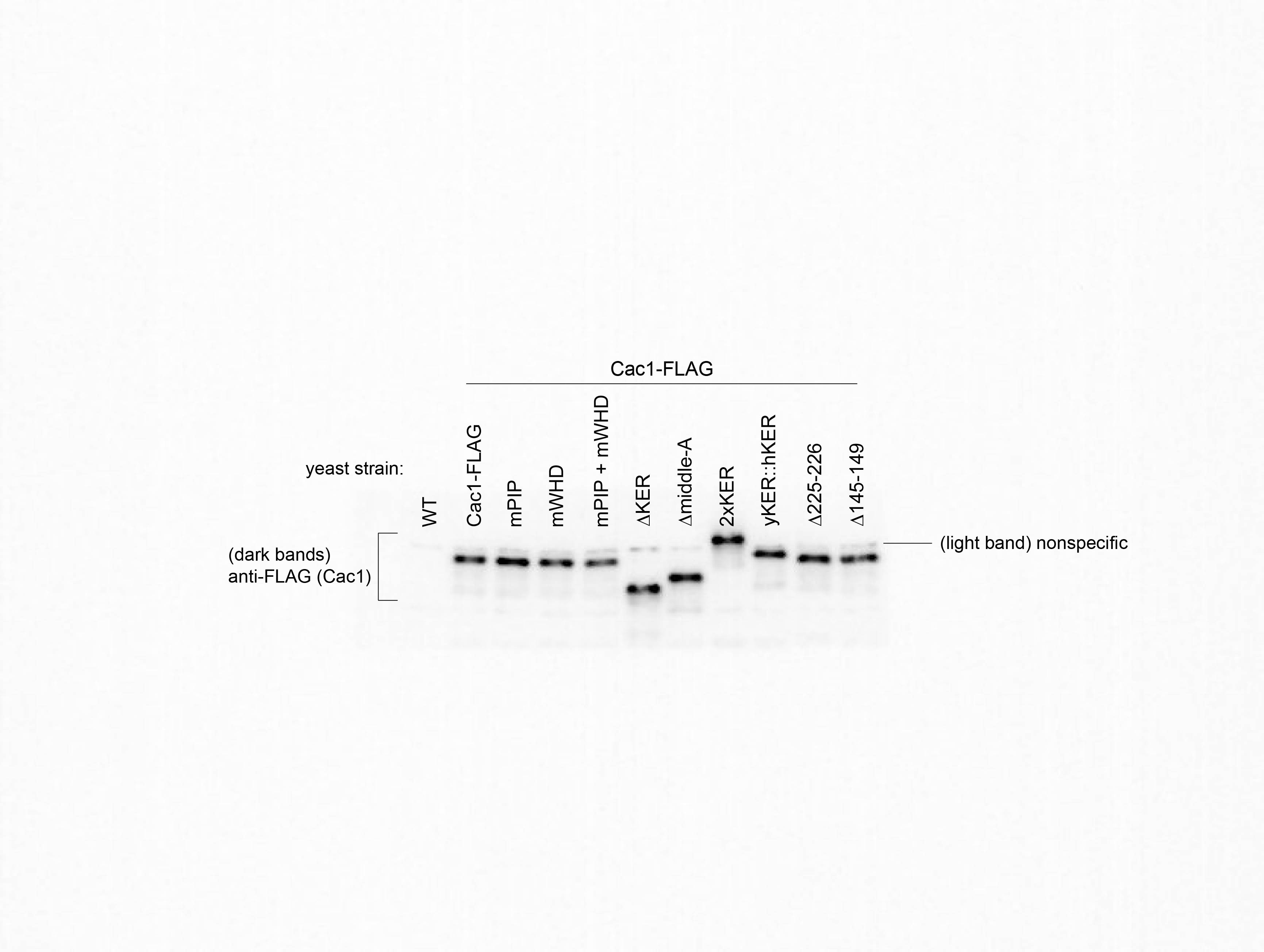

Supplement: Figure 1—figure supplement 2—source data 1. — Sodium dodecyl sulfate–polyacrylamide gel electrophoresis (SDS–PAGE) (panels a–c) and western blots (panel d). [file elife-83538-fig1-figsupp2-data1.zip › Figure 1 - figure supplement 2 - Source data 1/Figure S1d Cac1-FLAG Gel 1 labeled.jpg]

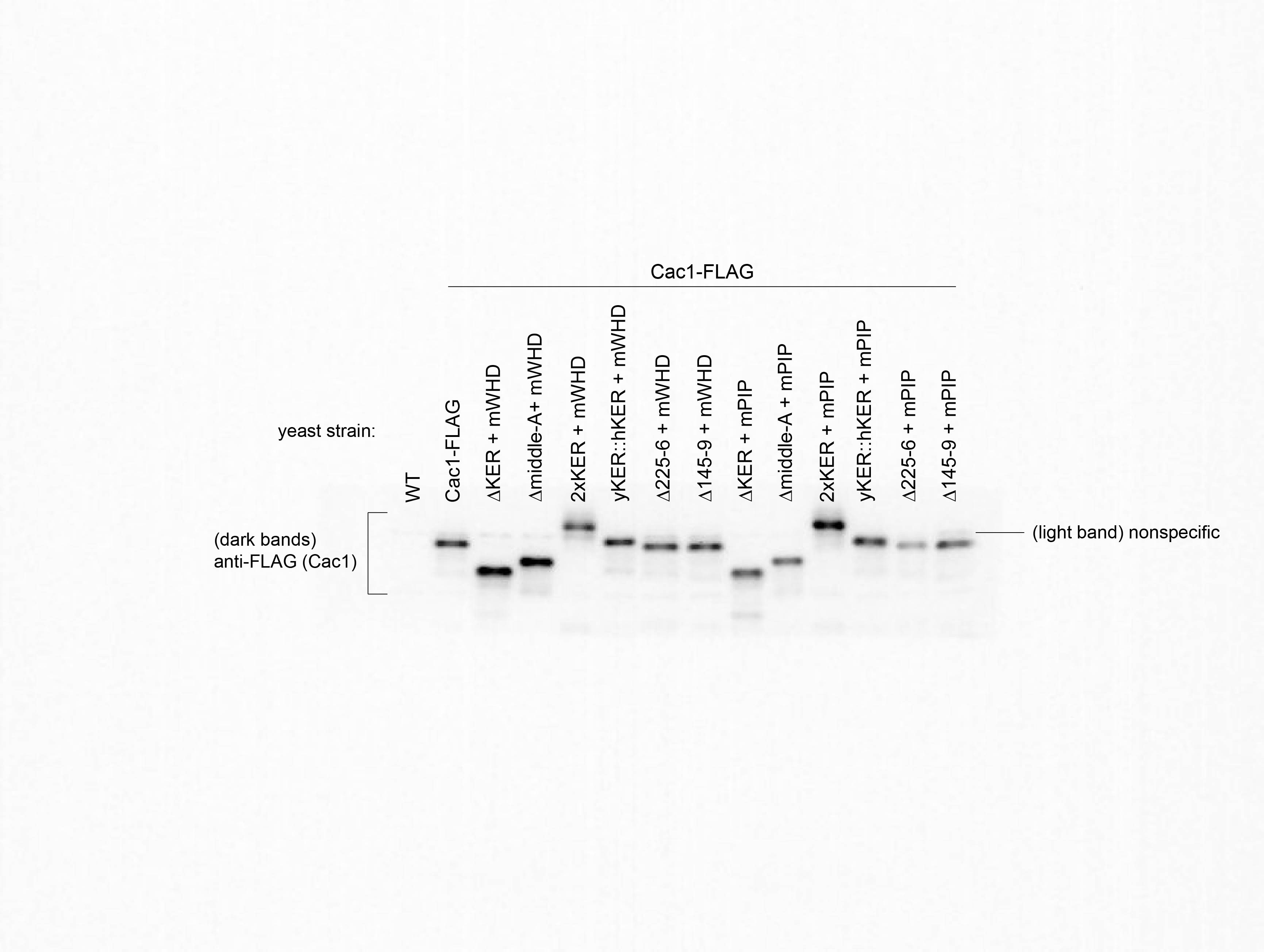

Supplement: Figure 1—figure supplement 2—source data 1. — Sodium dodecyl sulfate–polyacrylamide gel electrophoresis (SDS–PAGE) (panels a–c) and western blots (panel d). [file elife-83538-fig1-figsupp2-data1.zip › Figure 1 - figure supplement 2 - Source data 1/Figure S1d Cac1-FLAG Gel 2 labeled.jpg]

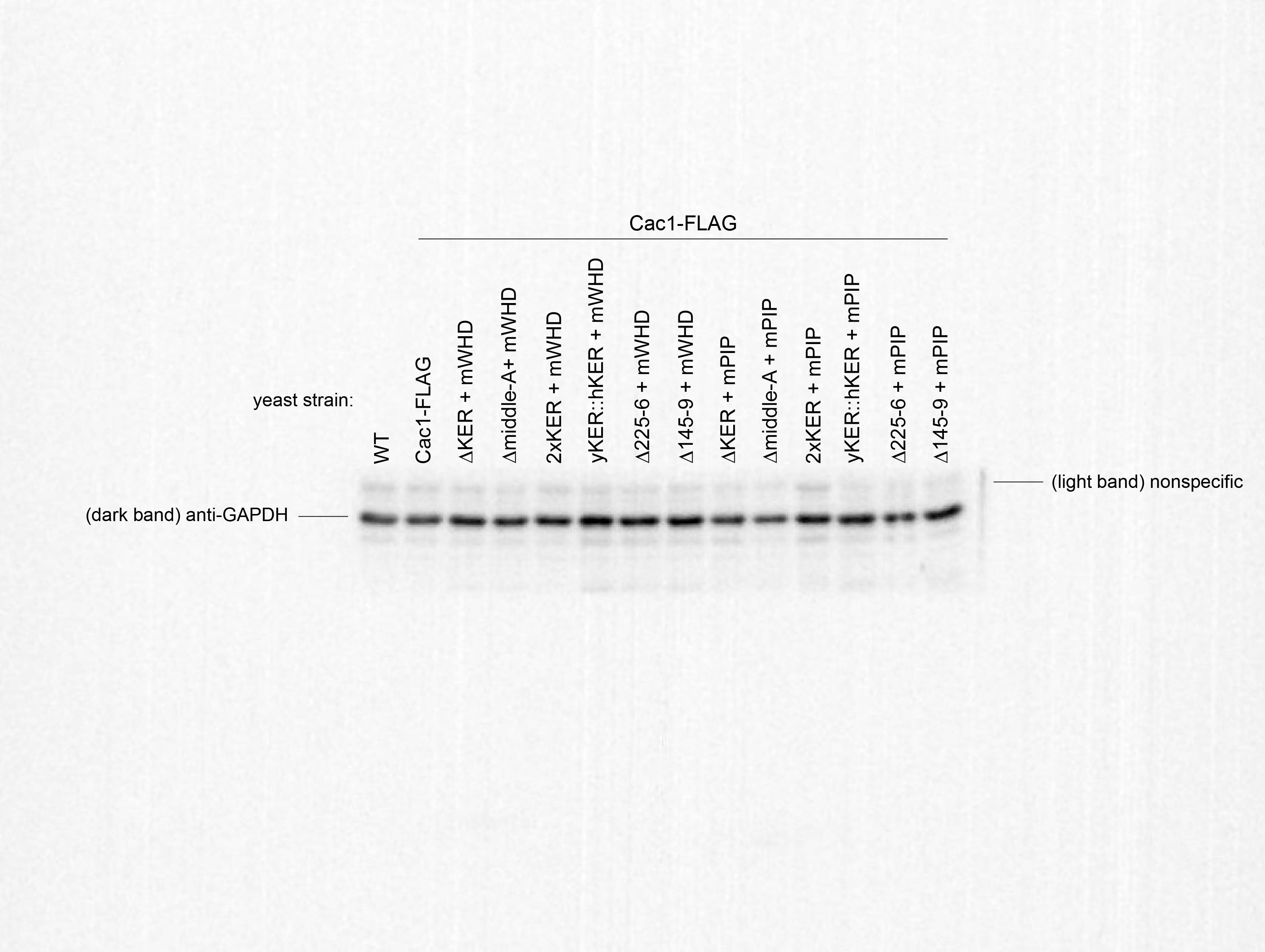

Supplement: Figure 1—figure supplement 2—source data 1. — Sodium dodecyl sulfate–polyacrylamide gel electrophoresis (SDS–PAGE) (panels a–c) and western blots (panel d). [file elife-83538-fig1-figsupp2-data1.zip › Figure 1 - figure supplement 2 - Source data 1/Figure S1d GAPDH Gel 2 labeled.jpg]

Other yCAF1 mutatin not used in this study

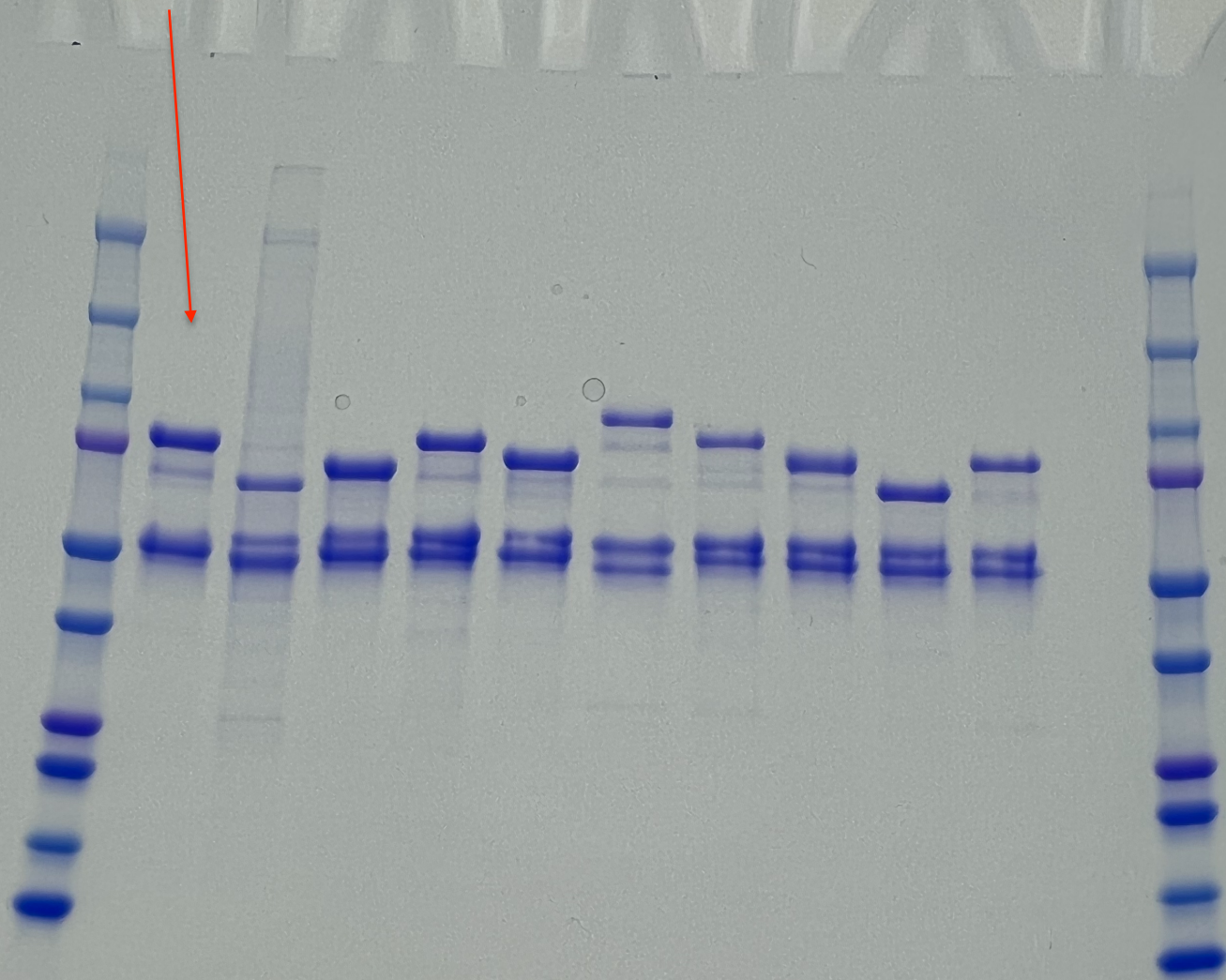

Supplement: Figure 1—figure supplement 2—source data 1. — Sodium dodecyl sulfate–polyacrylamide gel electrophoresis (SDS–PAGE) (panels a–c) and western blots (panel d). [file elife-83538-fig1-figsupp2-data1.zip › Figure 1 - figure supplement 2 - Source data 1/a/220329 SDS PAGE of yCAF1 Proteins.pdf]

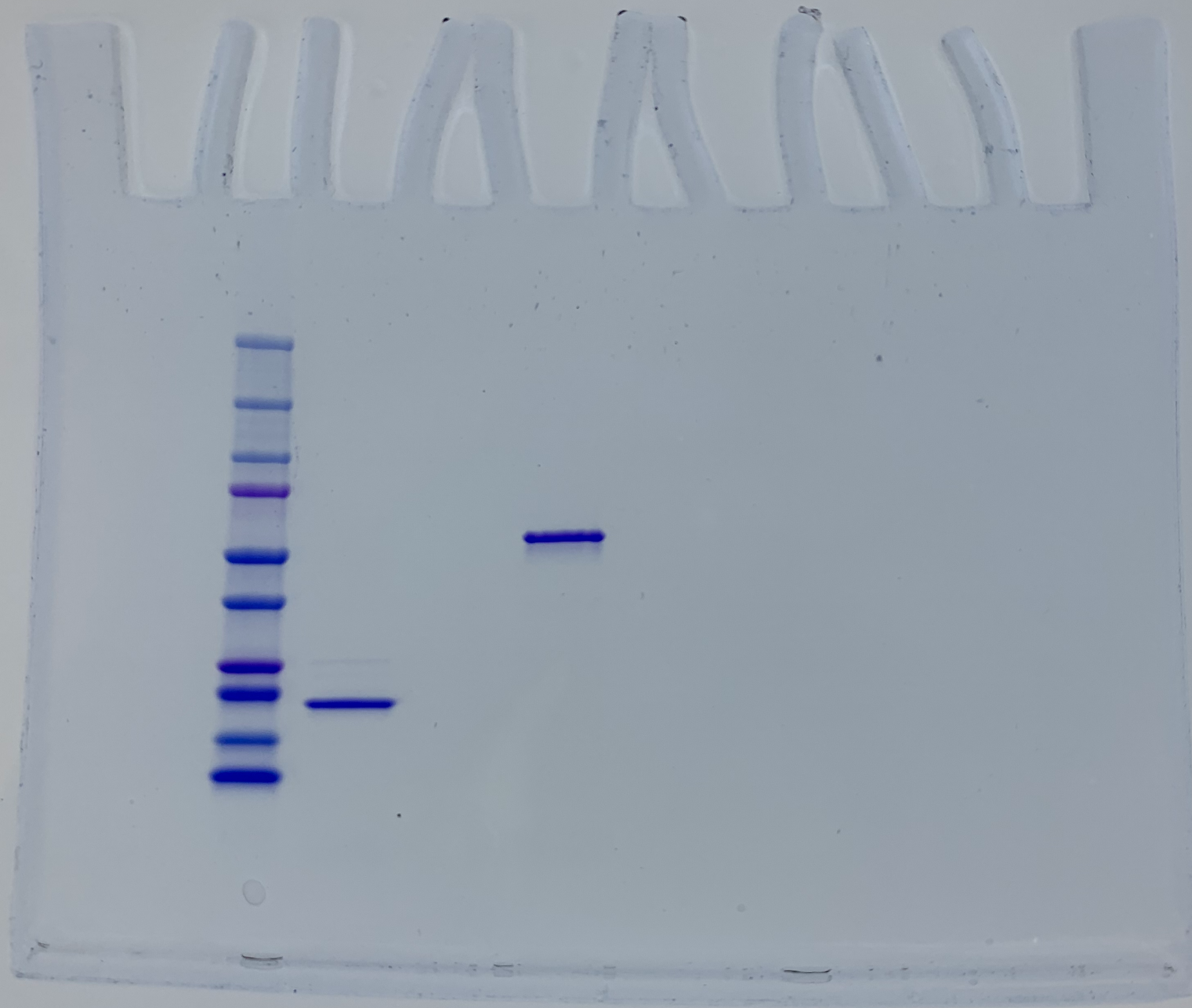

Supplement: Figure 1—figure supplement 2—source data 1. — Sodium dodecyl sulfate–polyacrylamide gel electrophoresis (SDS–PAGE) (panels a–c) and western blots (panel d). [file elife-83538-fig1-figsupp2-data1.zip › Figure 1 - figure supplement 2 - Source data 1/c/SDS PAGE of yWHD.pdf]

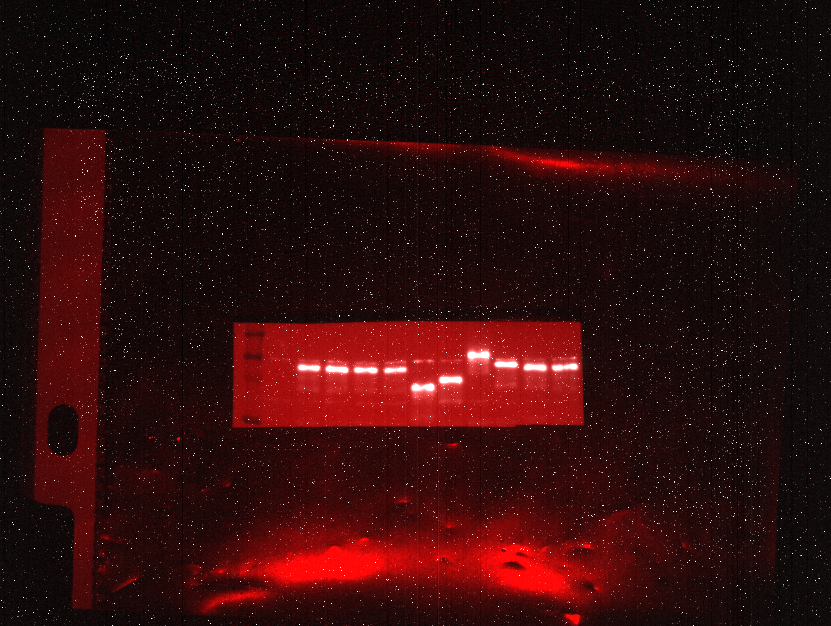

Supplement: Figure 1—figure supplement 2—source data 1. — Sodium dodecyl sulfate–polyacrylamide gel electrophoresis (SDS–PAGE) (panels a–c) and western blots (panel d). [file elife-83538-fig1-figsupp2-data1.zip › Figure 1 - figure supplement 2 - Source data 1/d/2022-07-13_13-52-06_8bit.png]

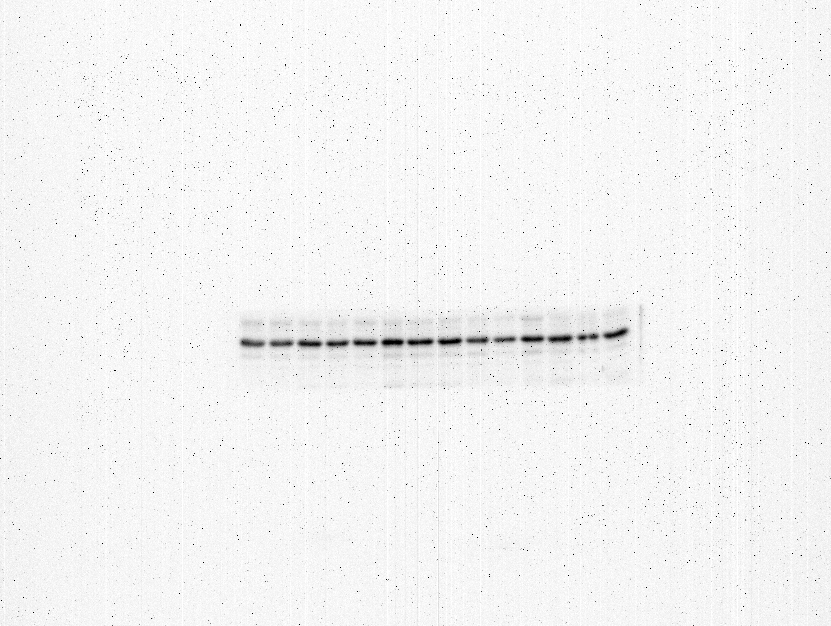

Supplement: Figure 1—figure supplement 2—source data 1. — Sodium dodecyl sulfate–polyacrylamide gel electrophoresis (SDS–PAGE) (panels a–c) and western blots (panel d). [file elife-83538-fig1-figsupp2-data1.zip › Figure 1 - figure supplement 2 - Source data 1/d/Figure S1d GAPDH Gel 2.png]

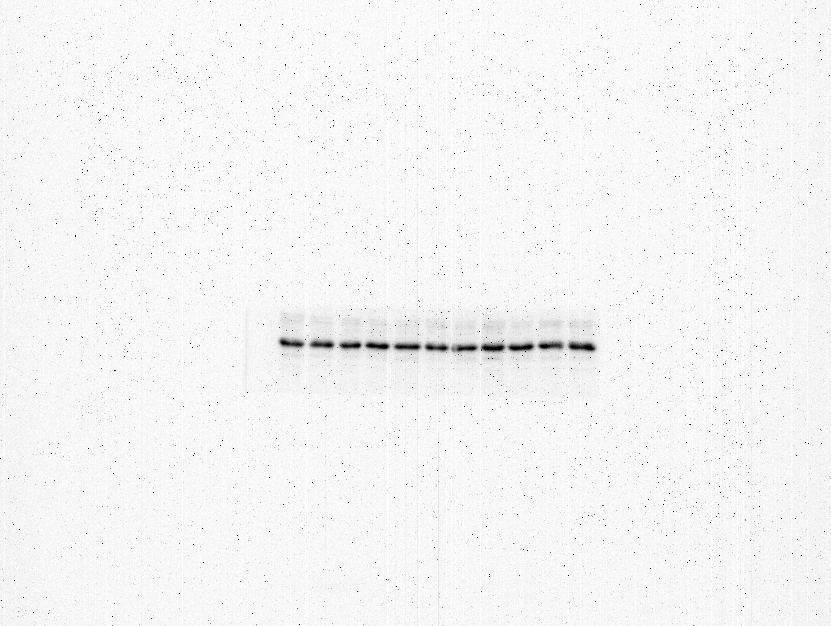

Supplement: Figure 1—figure supplement 2—source data 1. — Sodium dodecyl sulfate–polyacrylamide gel electrophoresis (SDS–PAGE) (panels a–c) and western blots (panel d). [file elife-83538-fig1-figsupp2-data1.zip › Figure 1 - figure supplement 2 - Source data 1/d/Figure S1D GAPDH Gel 1.png]

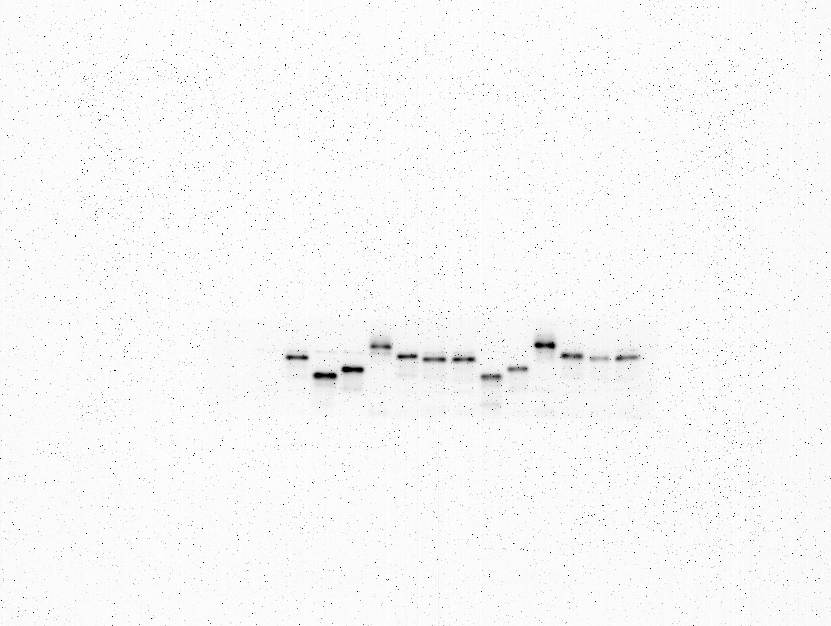

Supplement: Figure 1—figure supplement 2—source data 1. — Sodium dodecyl sulfate–polyacrylamide gel electrophoresis (SDS–PAGE) (panels a–c) and western blots (panel d). [file elife-83538-fig1-figsupp2-data1.zip › Figure 1 - figure supplement 2 - Source data 1/d/Figure S1d Cac1-FLAG Gel 2.png]

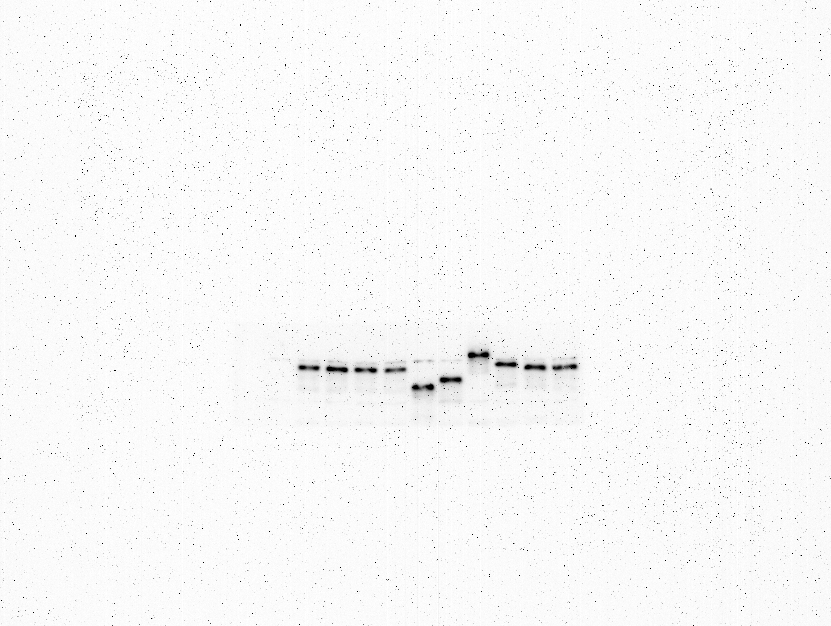

Supplement: Figure 1—figure supplement 2—source data 1. — Sodium dodecyl sulfate–polyacrylamide gel electrophoresis (SDS–PAGE) (panels a–c) and western blots (panel d). [file elife-83538-fig1-figsupp2-data1.zip › Figure 1 - figure supplement 2 - Source data 1/d/Figure S1d Cac1-FLAG Gel 1.png]

AAAAAAAA

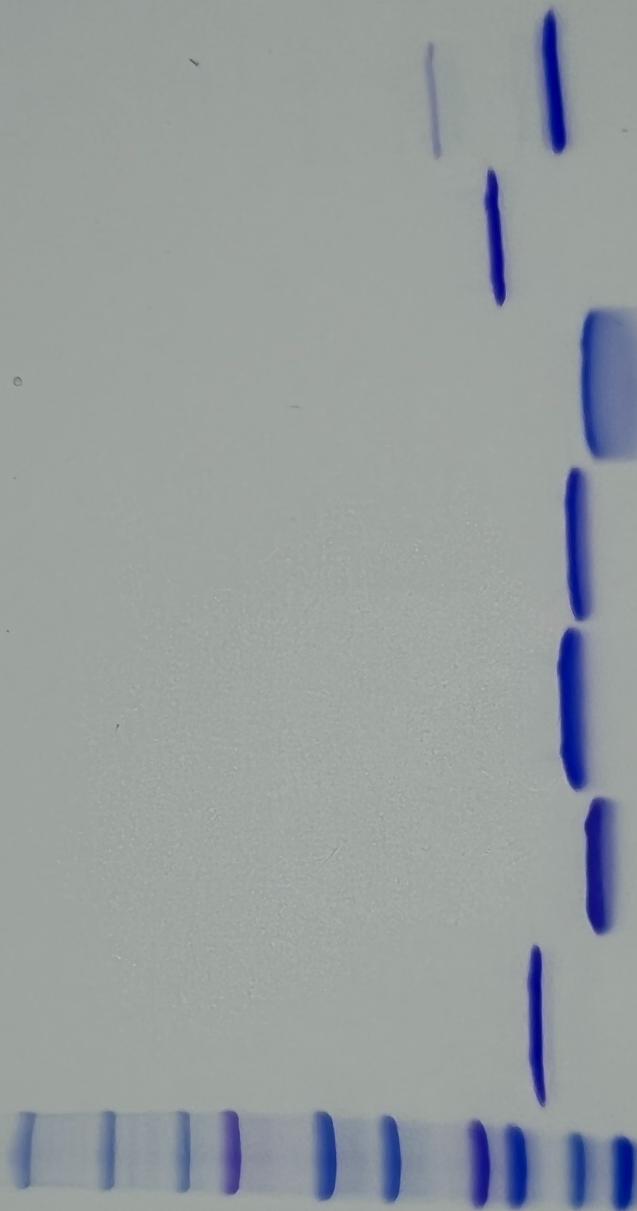

Supplement: Figure 1—figure supplement 2—source data 1. — Sodium dodecyl sulfate–polyacrylamide gel electrophoresis (SDS–PAGE) (panels a–c) and western blots (panel d). [file elife-83538-fig1-figsupp2-data1.zip › Figure 1 - figure supplement 2 - Source data 1/b/220329 SDS PAGE of KER Proteins.pdf]

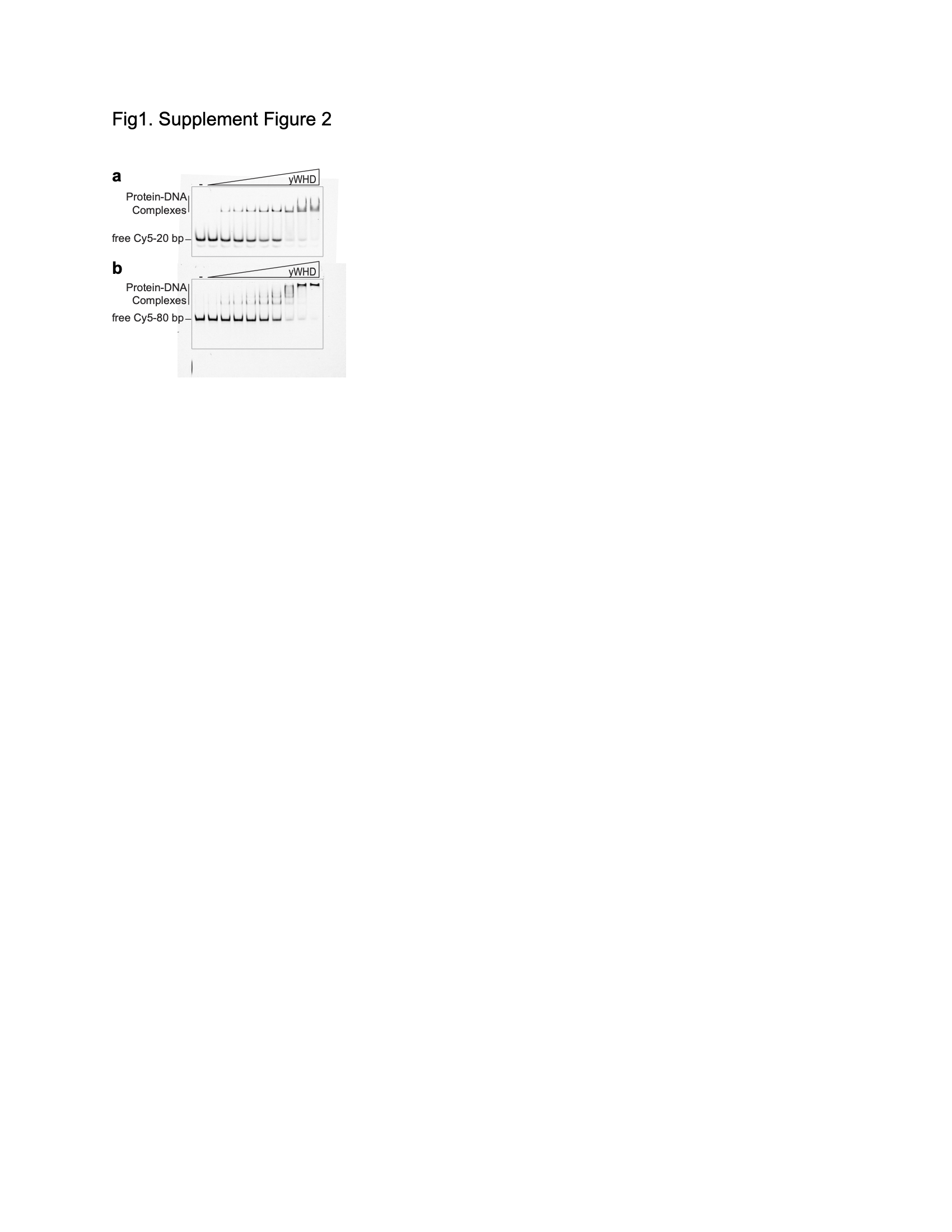

Supplement: Figure 1—figure supplement 3—source data 1. — Electrophoretic mobility shift assay (EMSA) images (panels a and b), data analyses (panel c), flow cytometry data (panel f), and Phosphorimager image (panel g). [file elife-83538-fig1-figsupp3-data1.zip › Figure 1 - figure supplement 3 - Source data 1/Figure 1 - figure supplement 3- Source data 1_Gels Labeled.png]

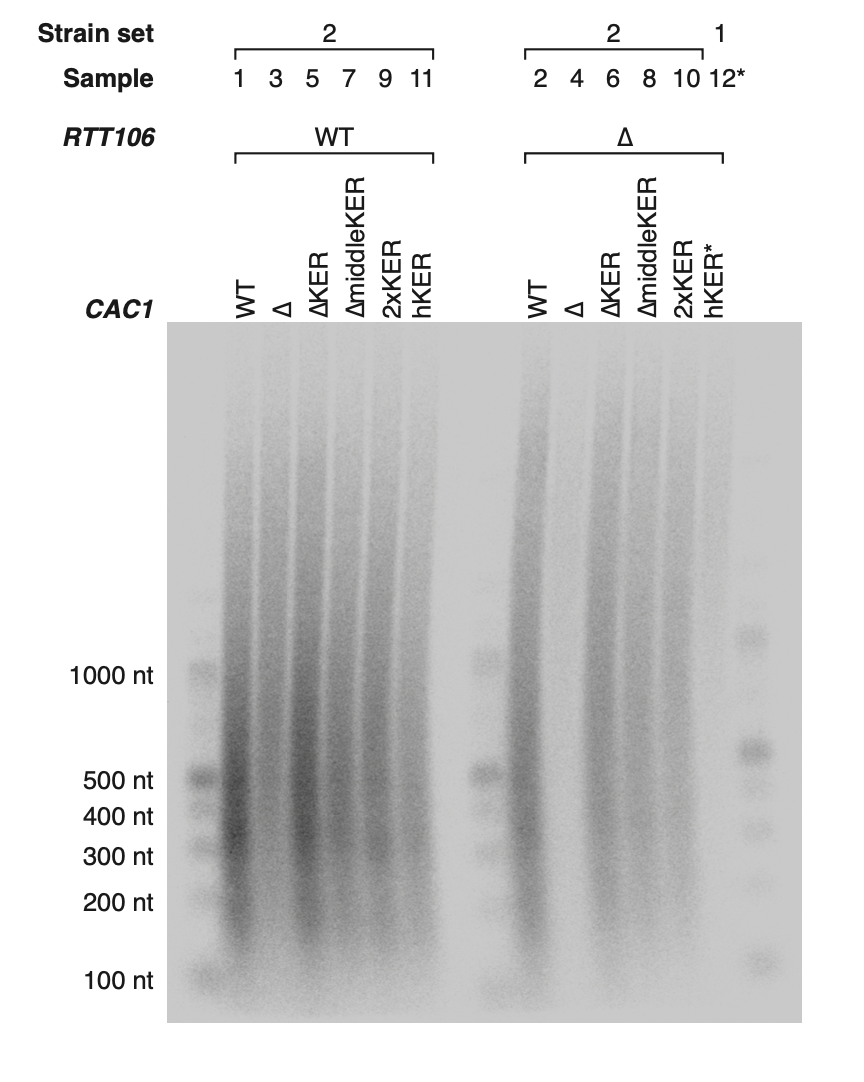

Supplement: Figure 1—figure supplement 3—source data 1. — Electrophoretic mobility shift assay (EMSA) images (panels a and b), data analyses (panel c), flow cytometry data (panel f), and Phosphorimager image (panel g). [file elife-83538-fig1-figsupp3-data1.zip › Figure 1 - figure supplement 3 - Source data 1/g/gel_1.png]

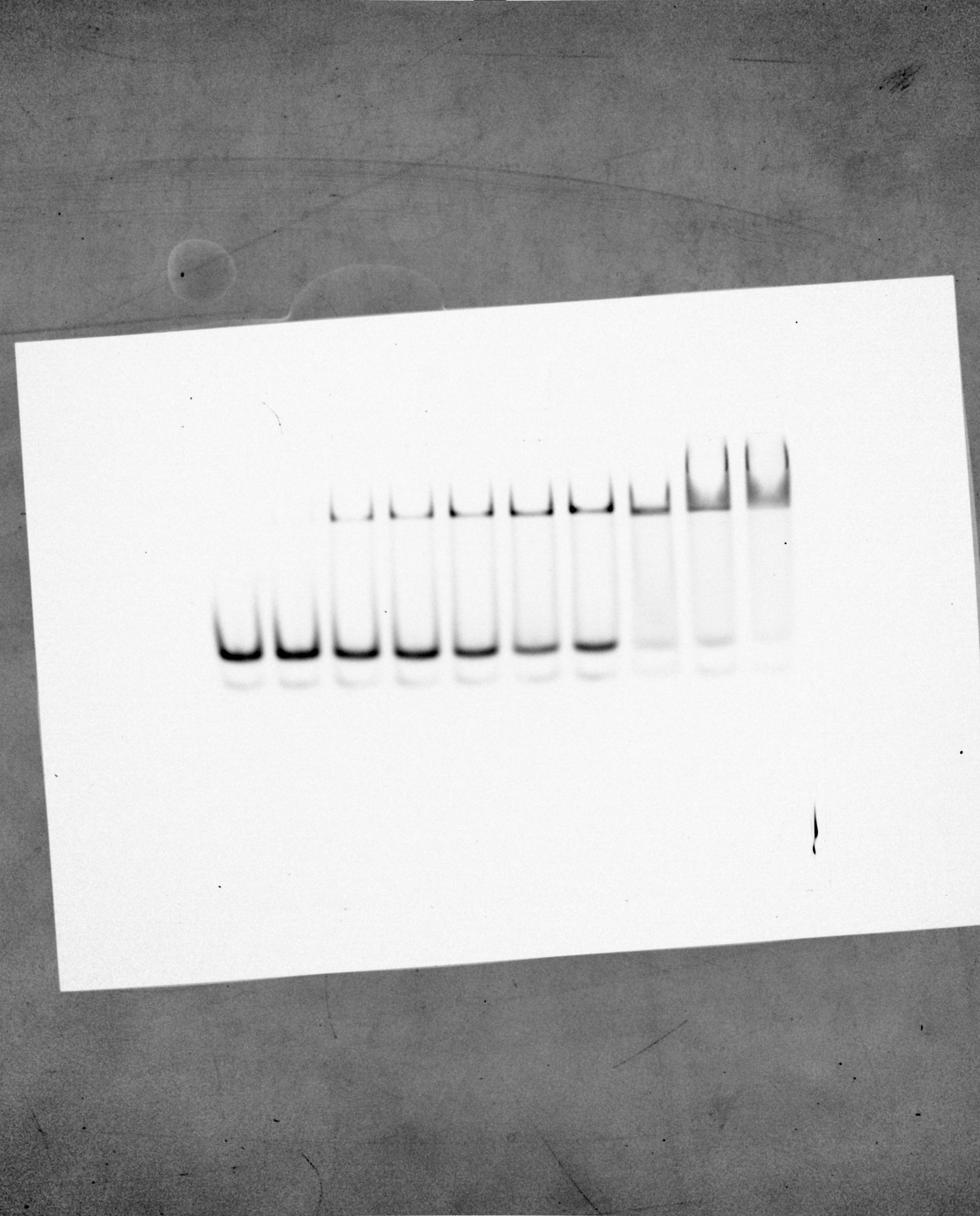

Supplement: Figure 1—figure supplement 3—source data 1. — Electrophoretic mobility shift assay (EMSA) images (panels a and b), data analyses (panel c), flow cytometry data (panel f), and Phosphorimager image (panel g). [file elife-83538-fig1-figsupp3-data1.zip › Figure 1 - figure supplement 3 - Source data 1/a/211220 Cy5 20bp EMSA with yWHD_PUB_600.tif]

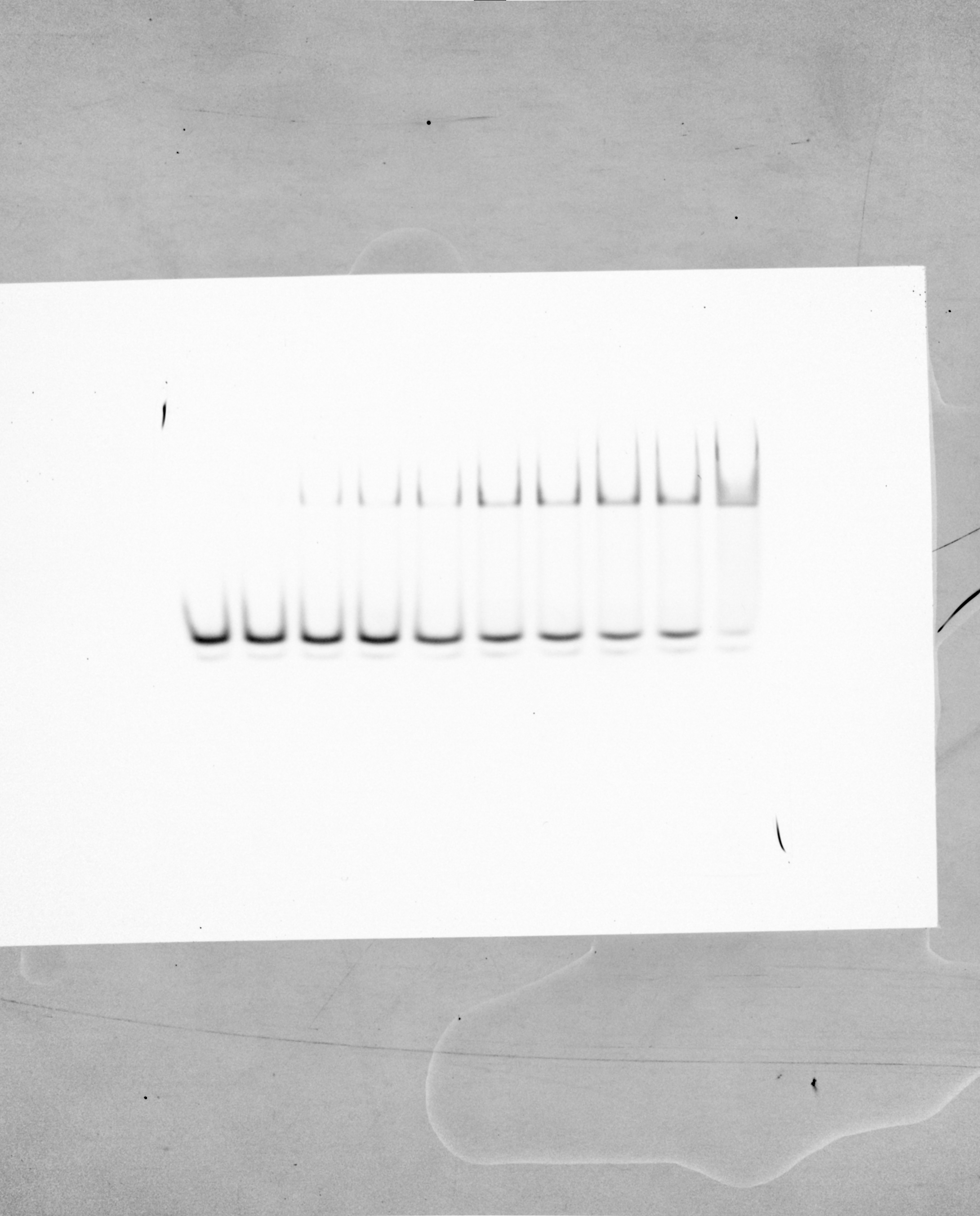

Supplement: Figure 1—figure supplement 3—source data 1. — Electrophoretic mobility shift assay (EMSA) images (panels a and b), data analyses (panel c), flow cytometry data (panel f), and Phosphorimager image (panel g). [file elife-83538-fig1-figsupp3-data1.zip › Figure 1 - figure supplement 3 - Source data 1/a/211206 Cy5 20bp EMSA with yWHD_n2_PUB_600.tif]

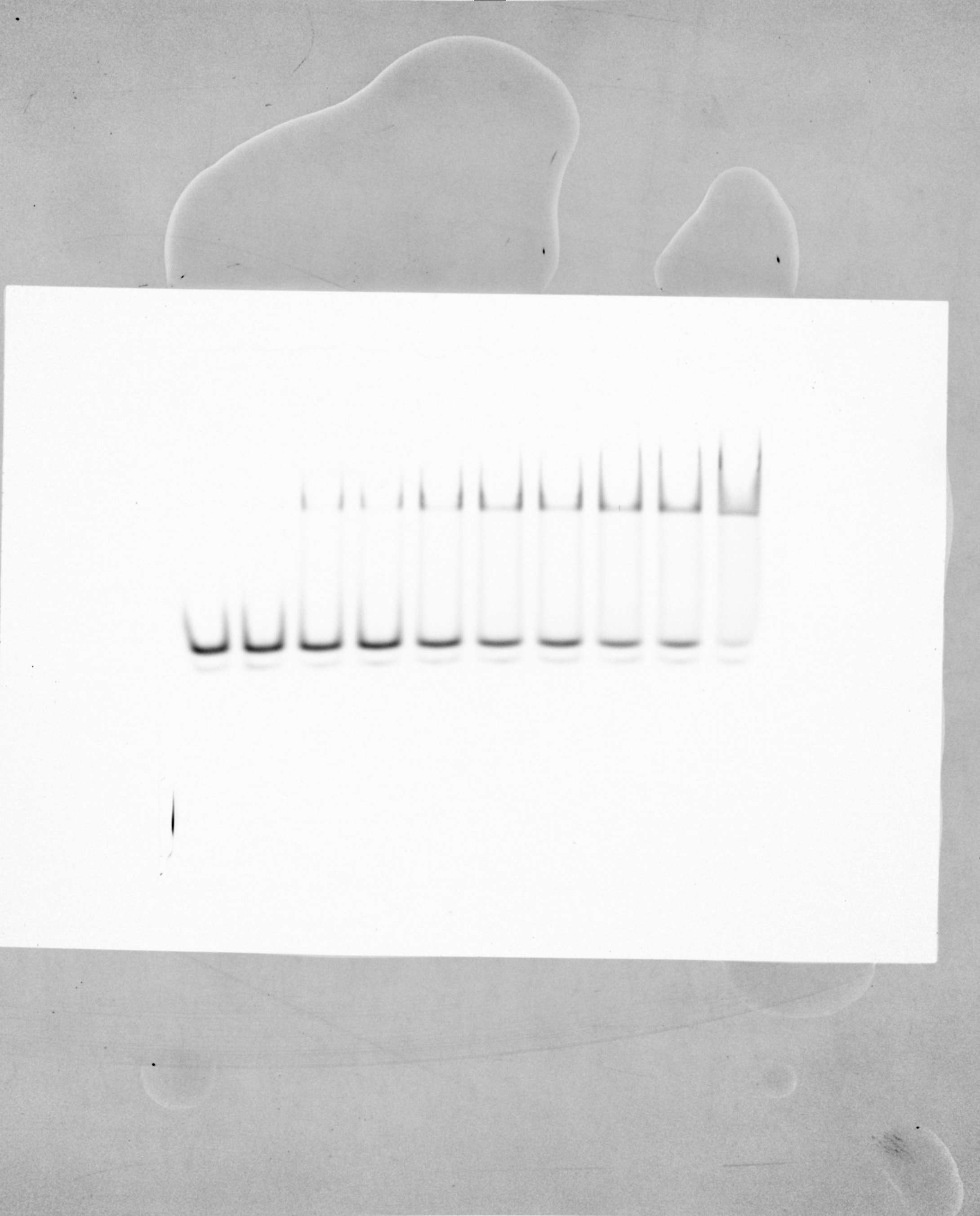

Supplement: Figure 1—figure supplement 3—source data 1. — Electrophoretic mobility shift assay (EMSA) images (panels a and b), data analyses (panel c), flow cytometry data (panel f), and Phosphorimager image (panel g). [file elife-83538-fig1-figsupp3-data1.zip › Figure 1 - figure supplement 3 - Source data 1/a/211206 Cy5 20bp EMSA with yWHD_n1_PUB_600.tif]

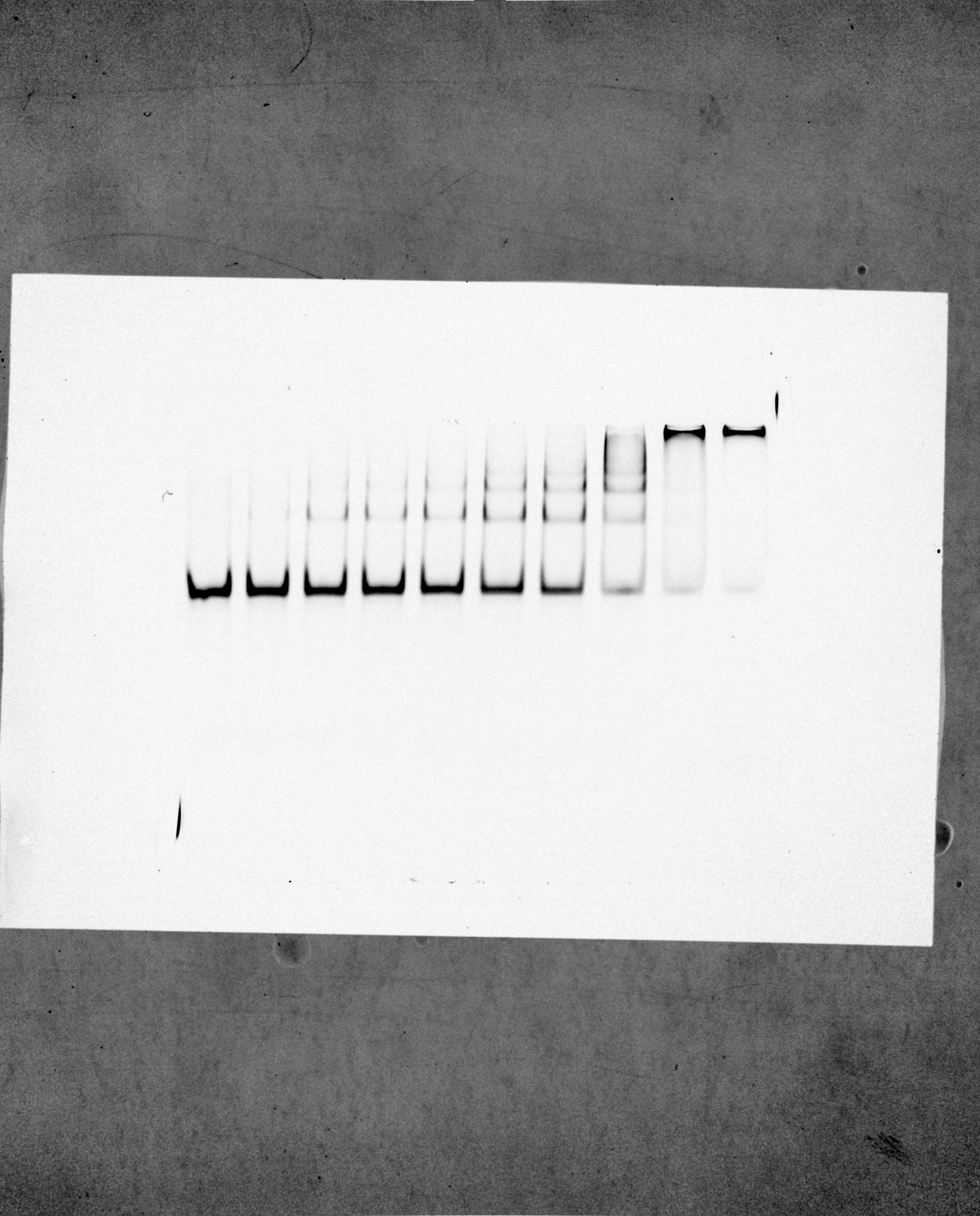

Supplement: Figure 1—figure supplement 3—source data 1. — Electrophoretic mobility shift assay (EMSA) images (panels a and b), data analyses (panel c), flow cytometry data (panel f), and Phosphorimager image (panel g). [file elife-83538-fig1-figsupp3-data1.zip › Figure 1 - figure supplement 3 - Source data 1/b/211026 Cy5 80bp EMSA with yWHD 457to606_n3_PUB_600.tif]

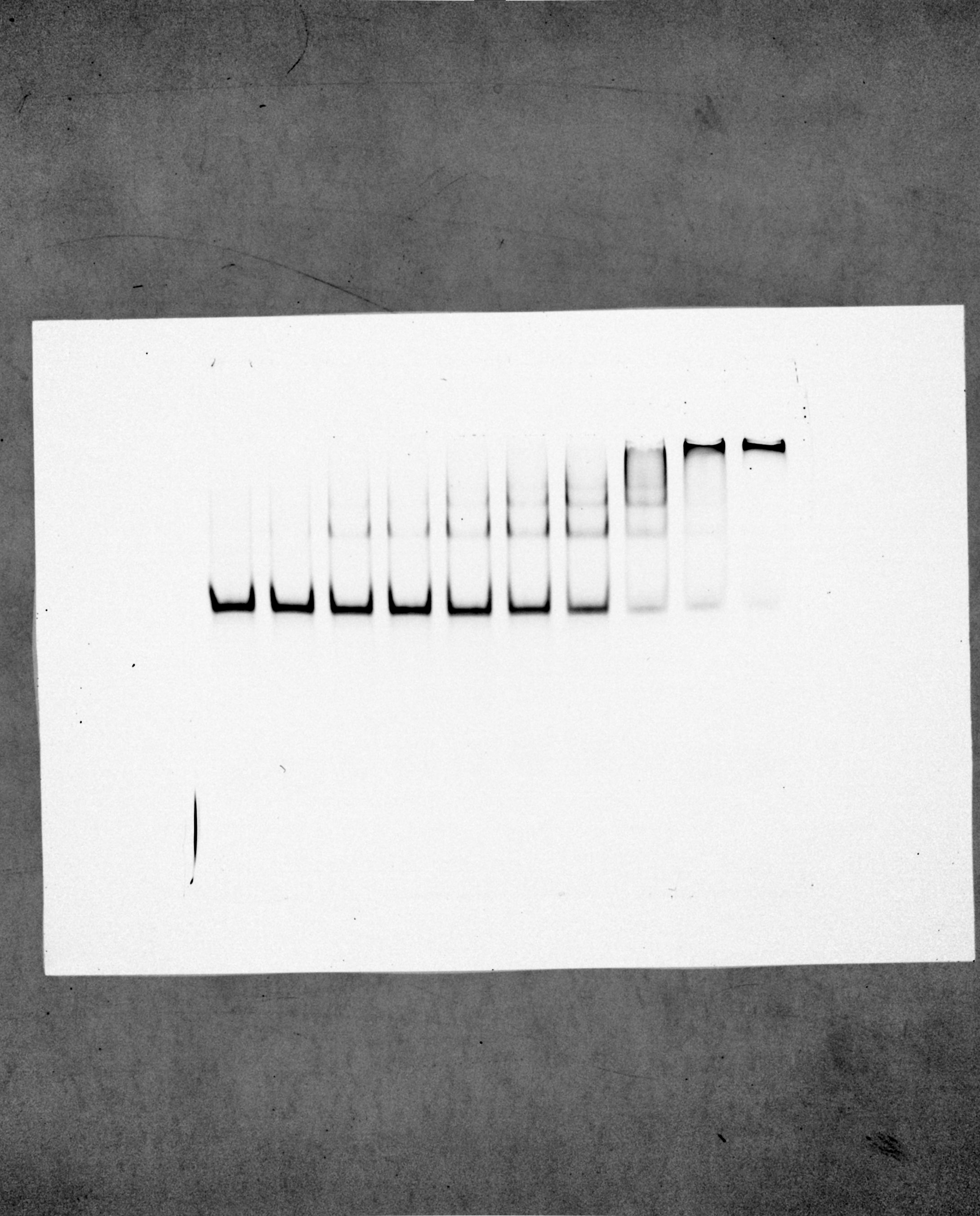

Supplement: Figure 1—figure supplement 3—source data 1. — Electrophoretic mobility shift assay (EMSA) images (panels a and b), data analyses (panel c), flow cytometry data (panel f), and Phosphorimager image (panel g). [file elife-83538-fig1-figsupp3-data1.zip › Figure 1 - figure supplement 3 - Source data 1/b/211026 Cy5 80bp EMSA with yWHD 457to606_n1_PUB_600.tif]

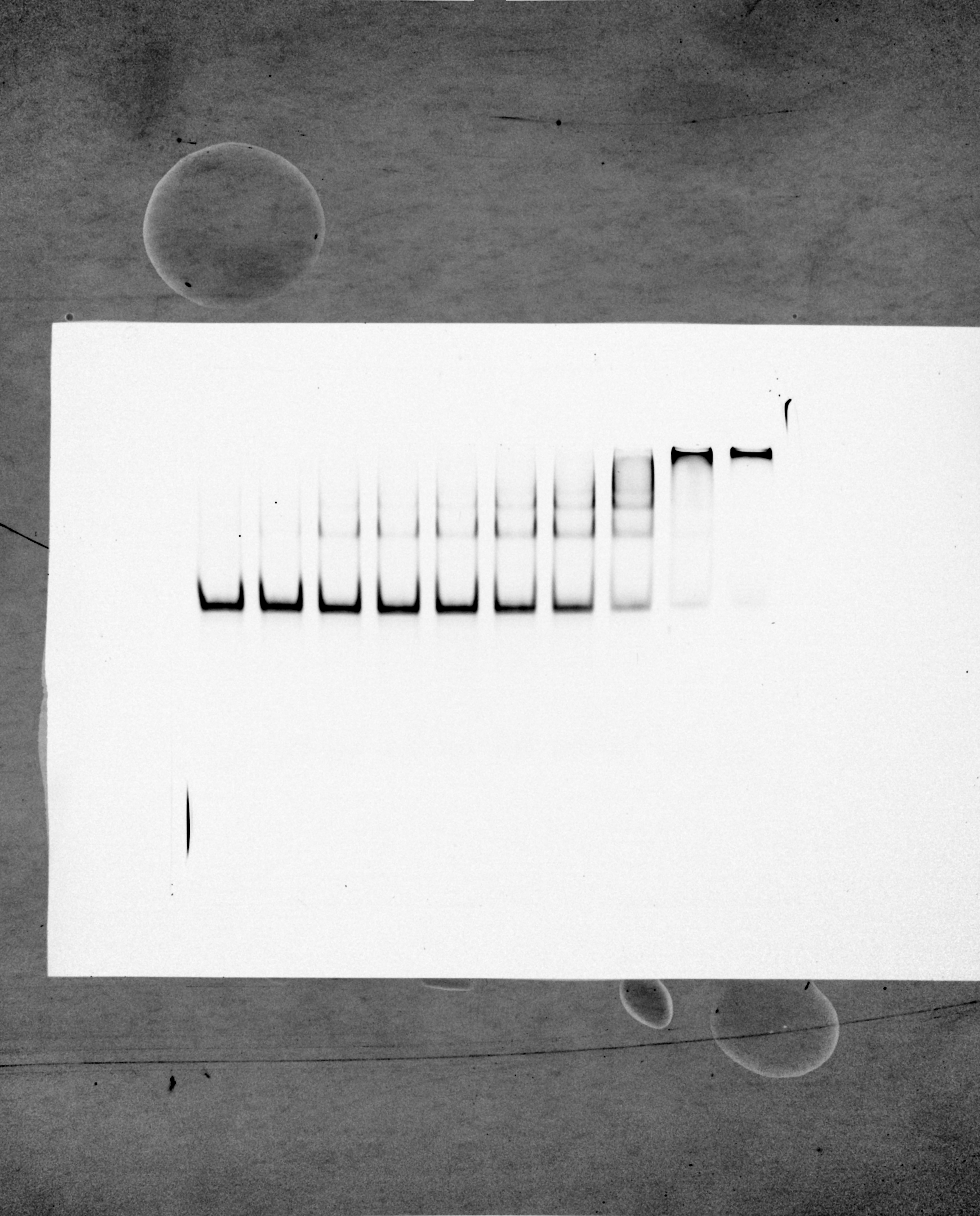

Supplement: Figure 1—figure supplement 3—source data 1. — Electrophoretic mobility shift assay (EMSA) images (panels a and b), data analyses (panel c), flow cytometry data (panel f), and Phosphorimager image (panel g). [file elife-83538-fig1-figsupp3-data1.zip › Figure 1 - figure supplement 3 - Source data 1/b/211026 Cy5 80bp EMSA with yWHD 457to606_n2_PUB_600.tif]

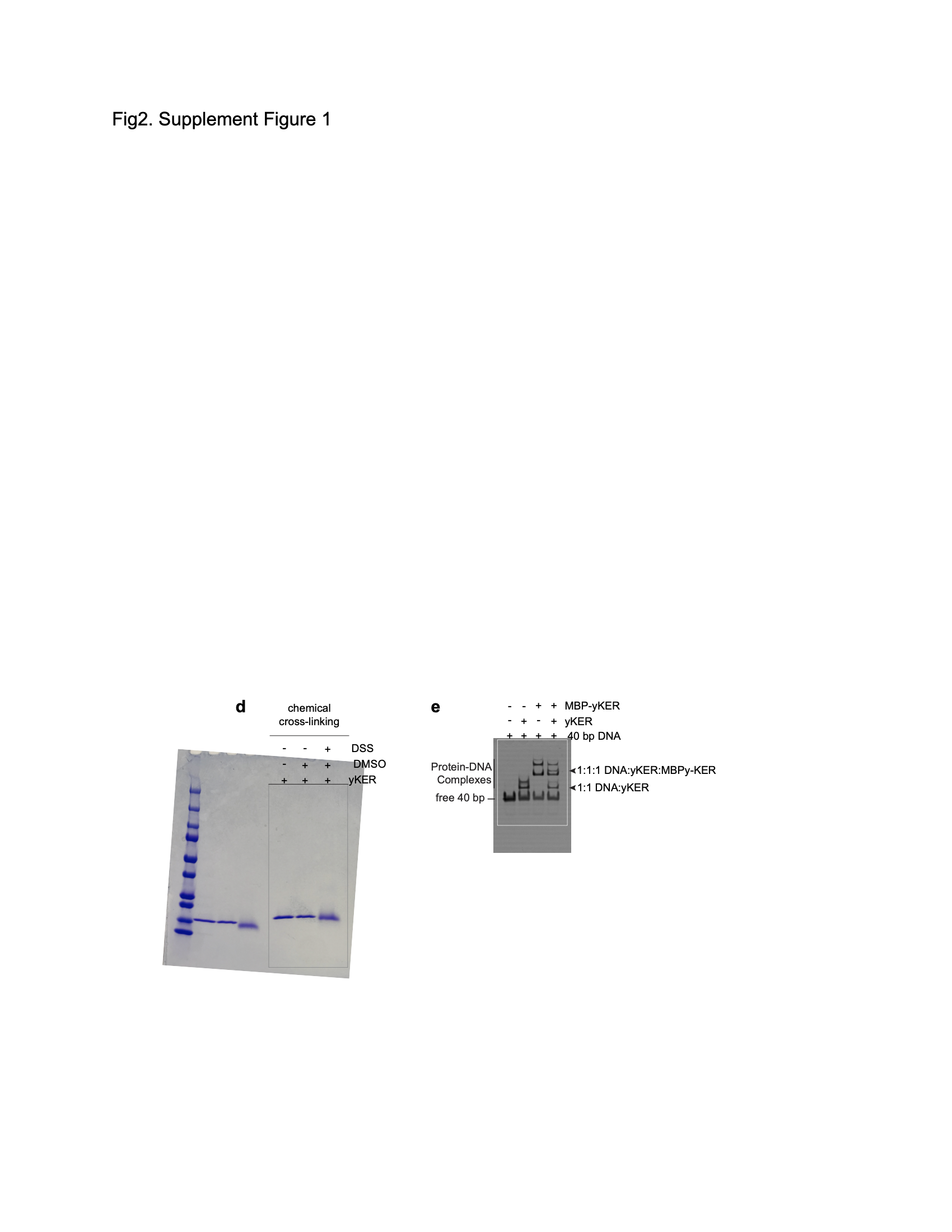

Supplement: Figure 2—figure supplement 1—source data 1. — Electrophoretic mobility shift assay (EMSA) images and data analyses (panels d and e). [file elife-83538-fig2-figsupp1-data1.zip › Figure 2 - figure supplement 1 - Source data 1/Figure 2 - figure supplement 1 - Source data 1_Gels Labeled.png]

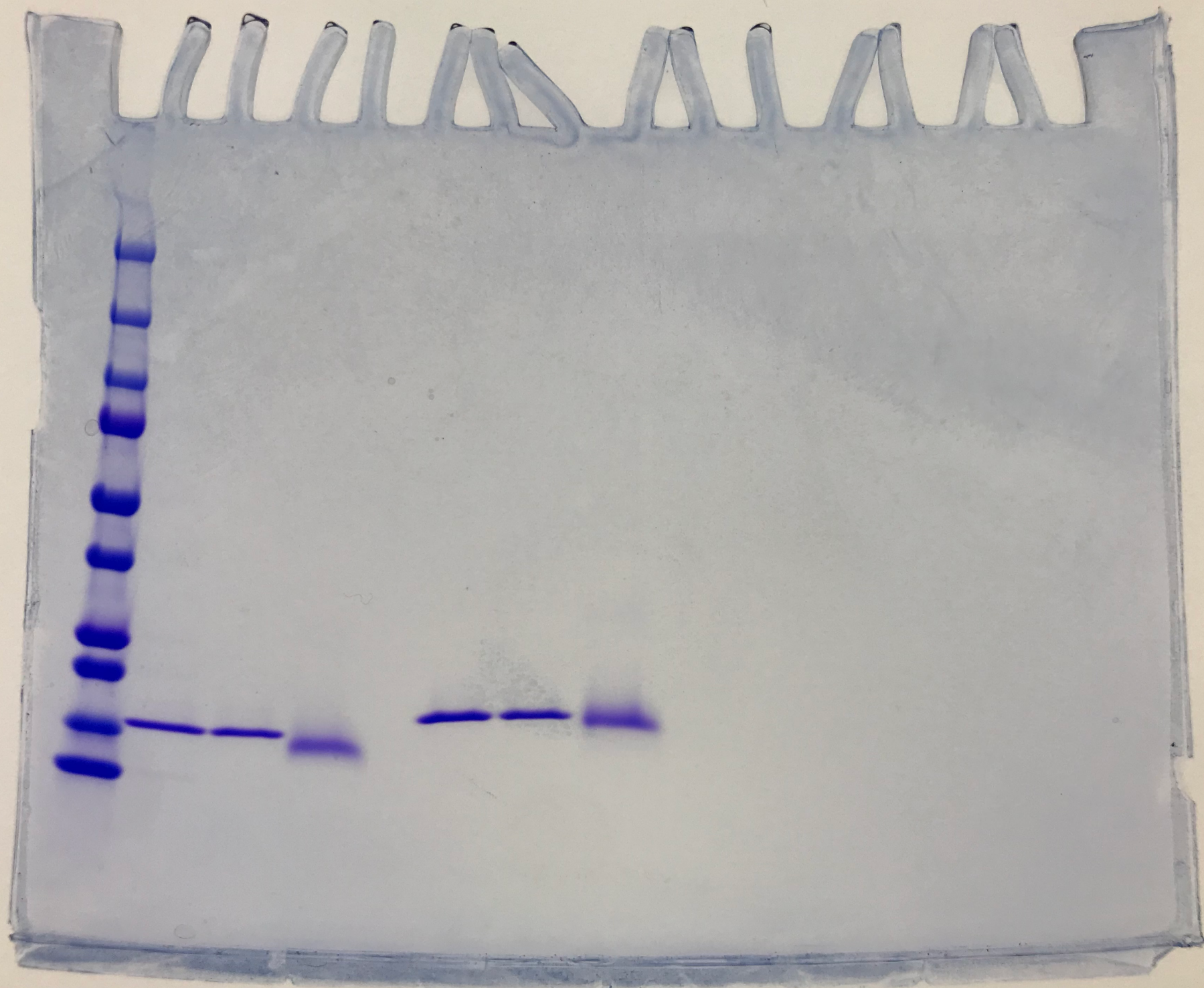

Supplement: Figure 2—figure supplement 1—source data 1. — Electrophoretic mobility shift assay (EMSA) images and data analyses (panels d and e). [file elife-83538-fig2-figsupp1-data1.zip › Figure 2 - figure supplement 1 - Source data 1/d/190226 KER DSS xlinking including Christals construct 118 to 232.pdf]

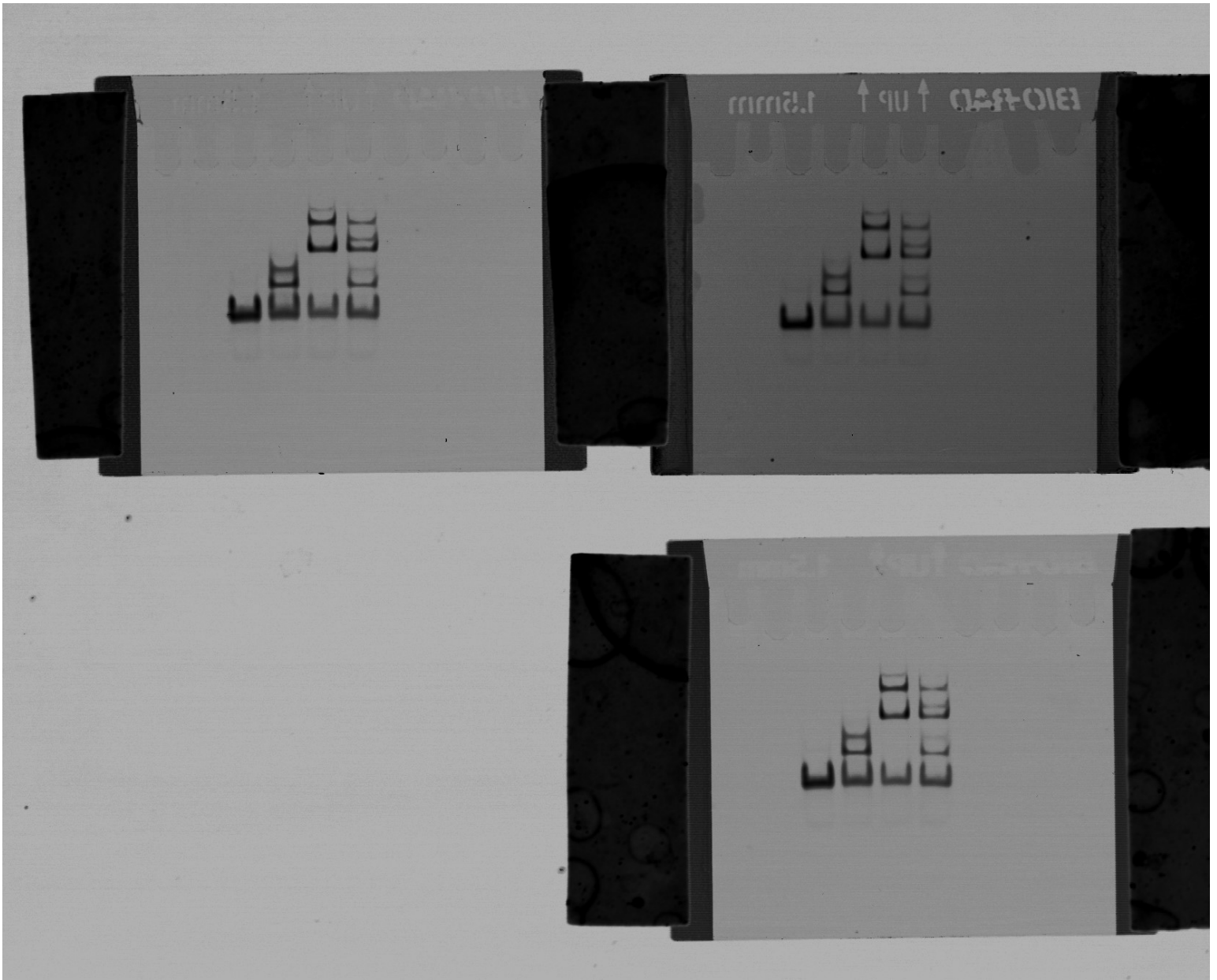

Supplement: Figure 2—figure supplement 1—source data 1. — Electrophoretic mobility shift assay (EMSA) images and data analyses (panels d and e). [file elife-83538-fig2-figsupp1-data1.zip › Figure 2 - figure supplement 1 - Source data 1/e/yKER mix and match EMSA.pdf]

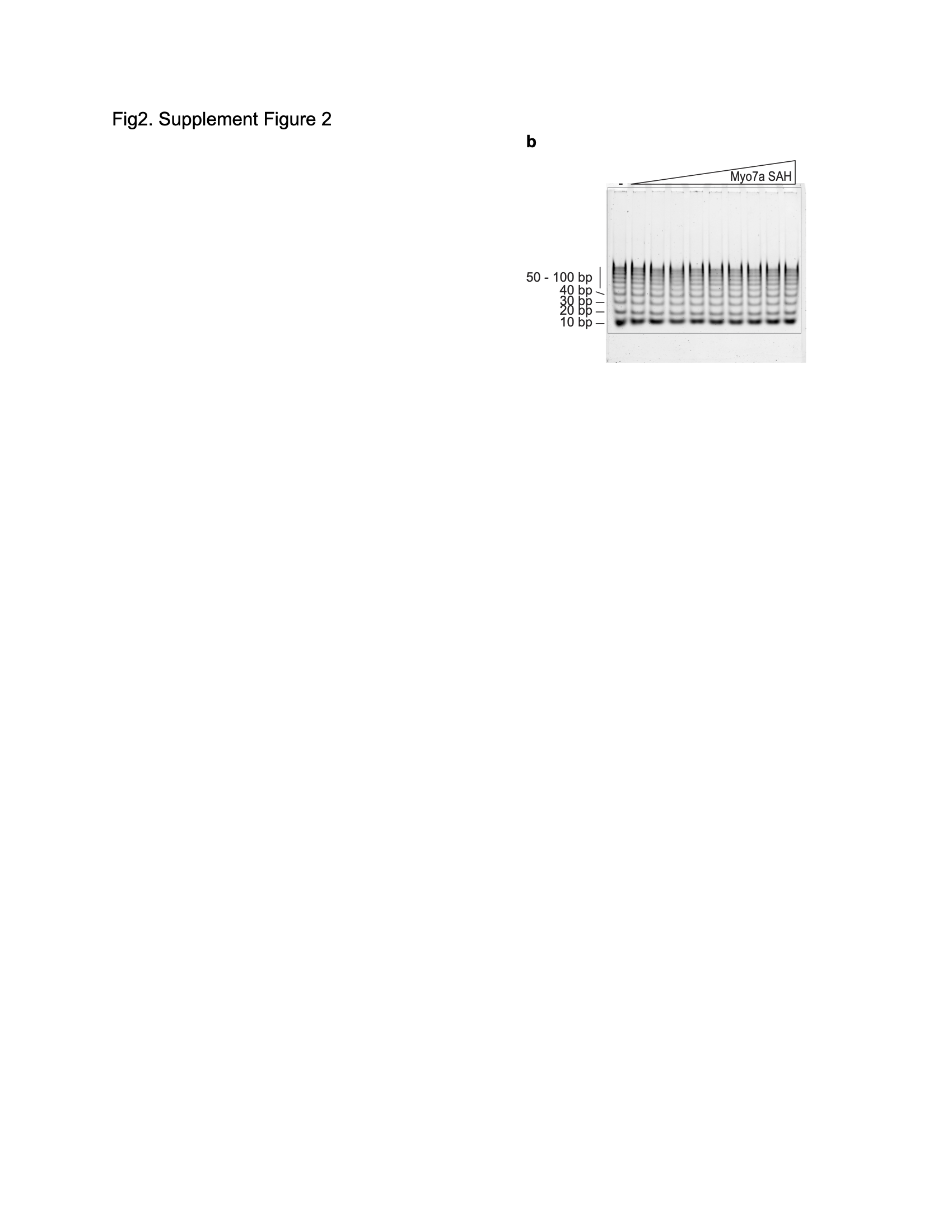

Supplement: Figure 2—figure supplement 2—source data 1. — Circular dichroism data (panel a) and electrophoretic mobility shift assay (EMSA) images (panel b). [file elife-83538-fig2-figsupp2-data1.zip › Figure 2 - figure supplement 2 - Source data 1/Figure 2 - figure supplement 2 - Source data 1_Gels Labeled.png]

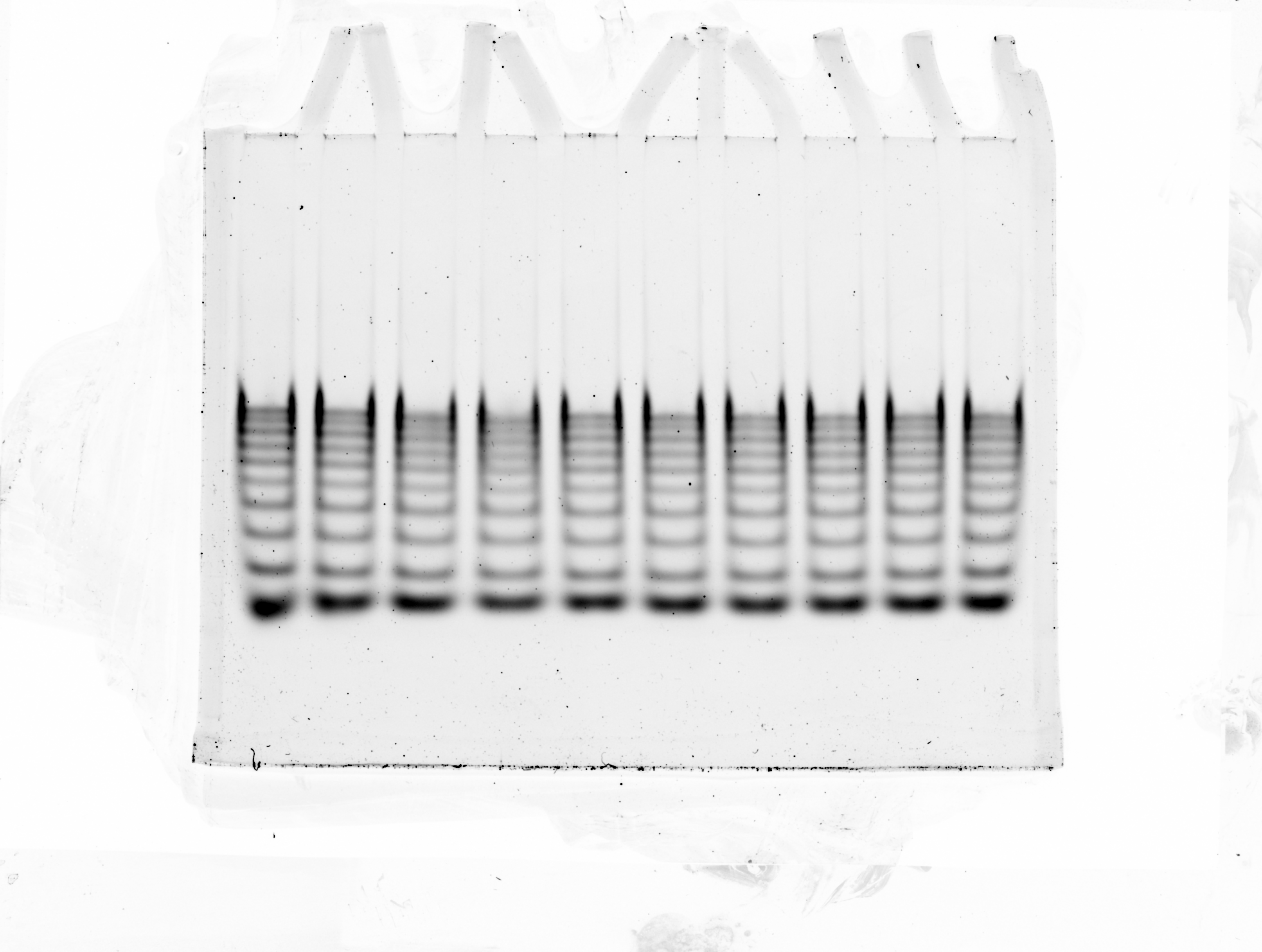

Supplement: Figure 2—figure supplement 2—source data 1. — Circular dichroism data (panel a) and electrophoretic mobility shift assay (EMSA) images (panel b). [file elife-83538-fig2-figsupp2-data1.zip › Figure 2 - figure supplement 2 - Source data 1/b/210119 10 bp ladder EMSA with Myosin7 SAH 4s with Quenching sheet SYBR Green_2_PUB_600.tif]

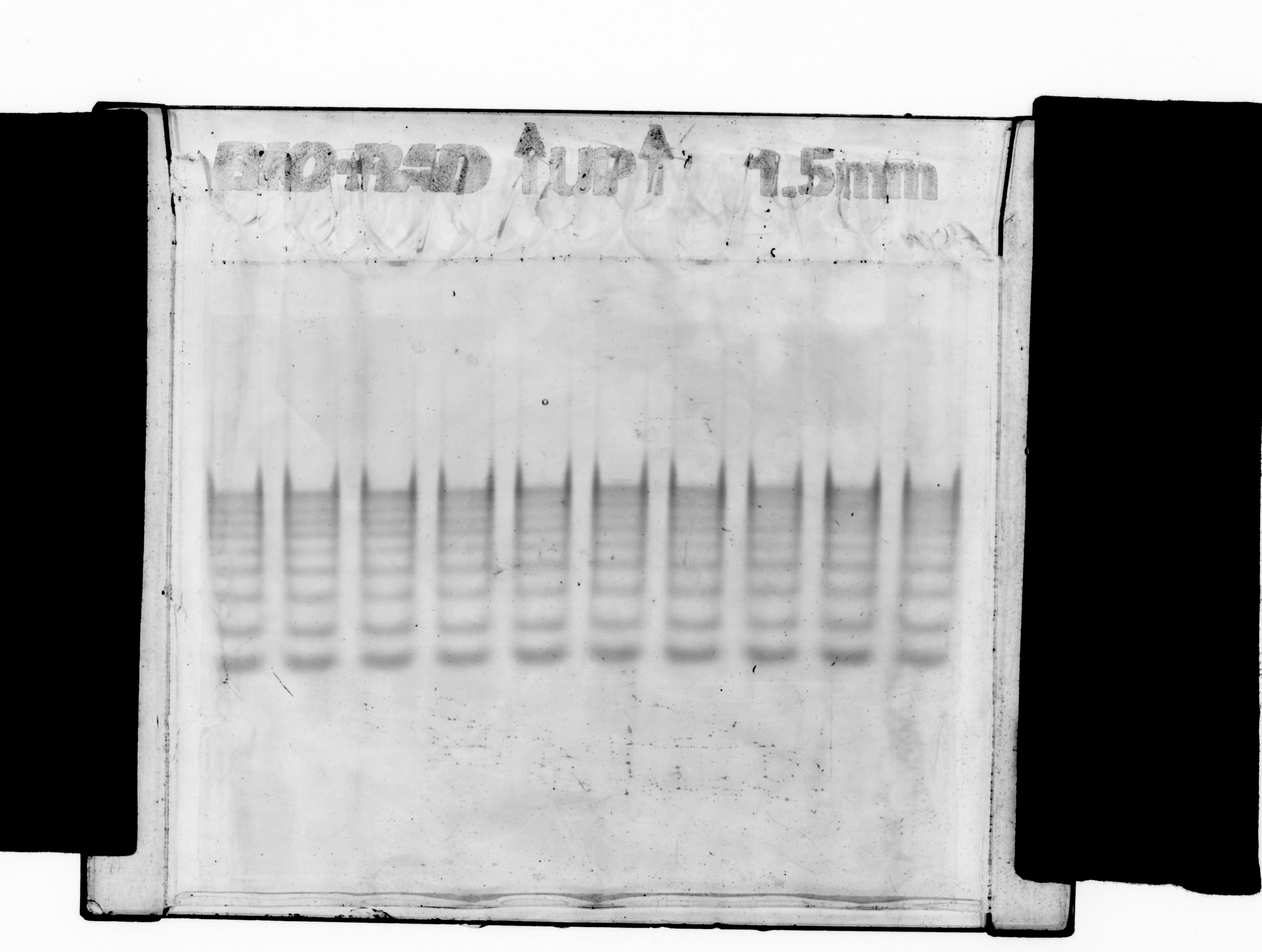

Supplement: Figure 2—figure supplement 2—source data 1. — Circular dichroism data (panel a) and electrophoretic mobility shift assay (EMSA) images (panel b). [file elife-83538-fig2-figsupp2-data1.zip › Figure 2 - figure supplement 2 - Source data 1/b/210112 10bp ladder with Myosin7a SAH SYBR Green_PUB_600.tif]

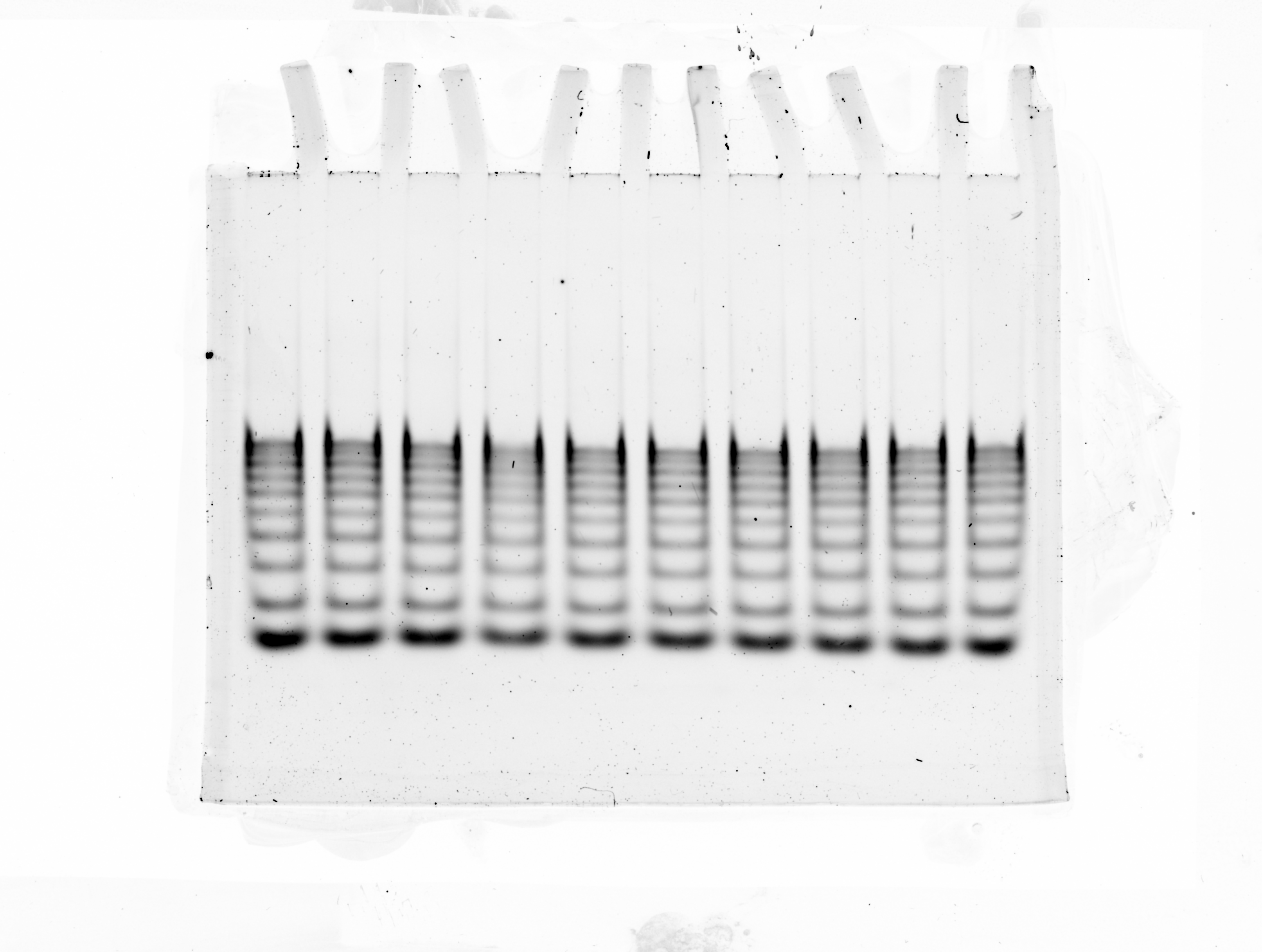

Supplement: Figure 2—figure supplement 2—source data 1. — Circular dichroism data (panel a) and electrophoretic mobility shift assay (EMSA) images (panel b). [file elife-83538-fig2-figsupp2-data1.zip › Figure 2 - figure supplement 2 - Source data 1/b/210119 10 bp ladder EMSA with Myosin7 SAH 4s with Quenching sheet SYBR Green_1_PUB_600.tif]

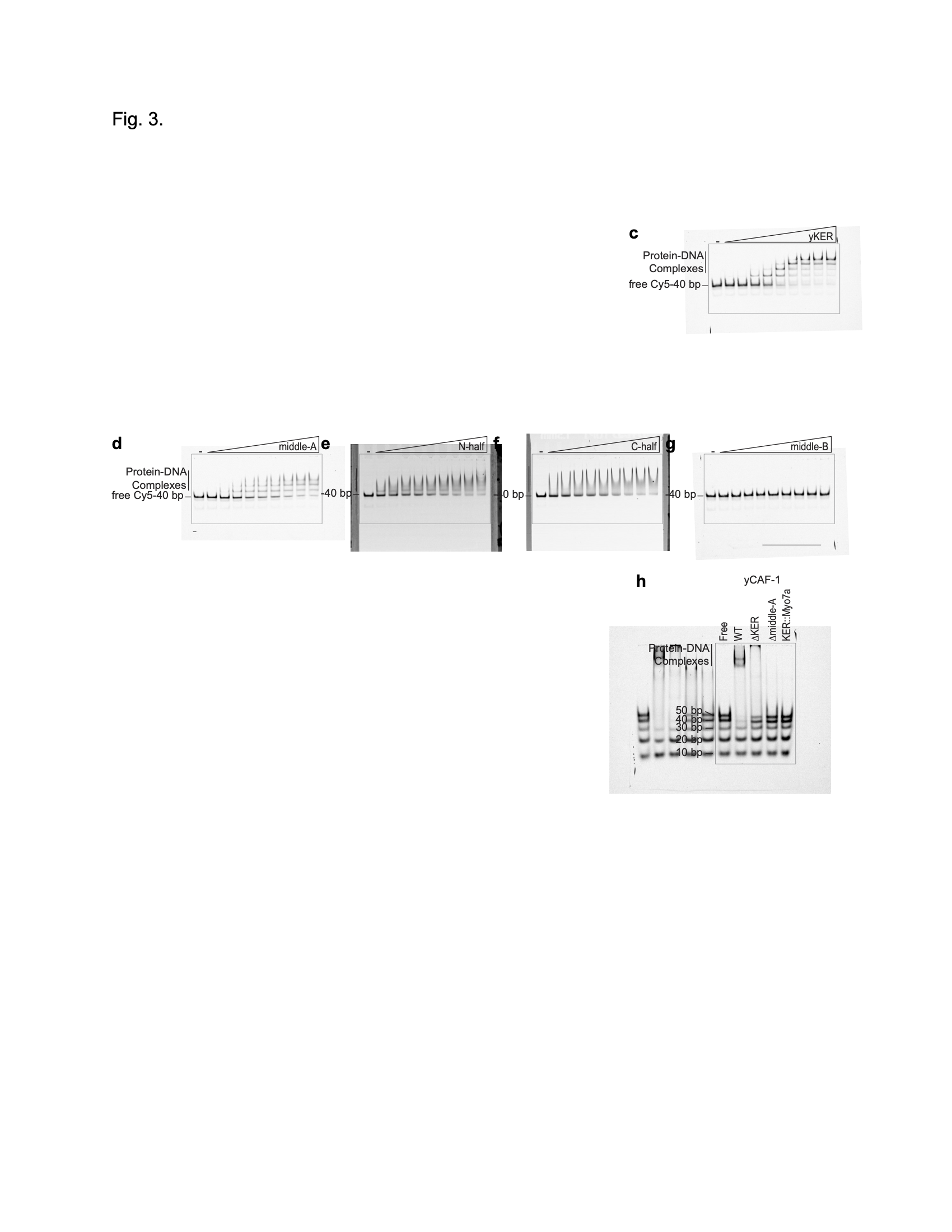

Supplement: Figure 3—source data 1. — Circular dichroism data (panel b), electrophoretic mobility shift assay (EMSA) images (panels c–h), flow cytometry data (panel j), and data analyses (panels c–f, j). [file elife-83538-fig3-data1.zip › Figure 3 - Source data 1/Figure 3 - Source data 1_Gels Labeled.png]

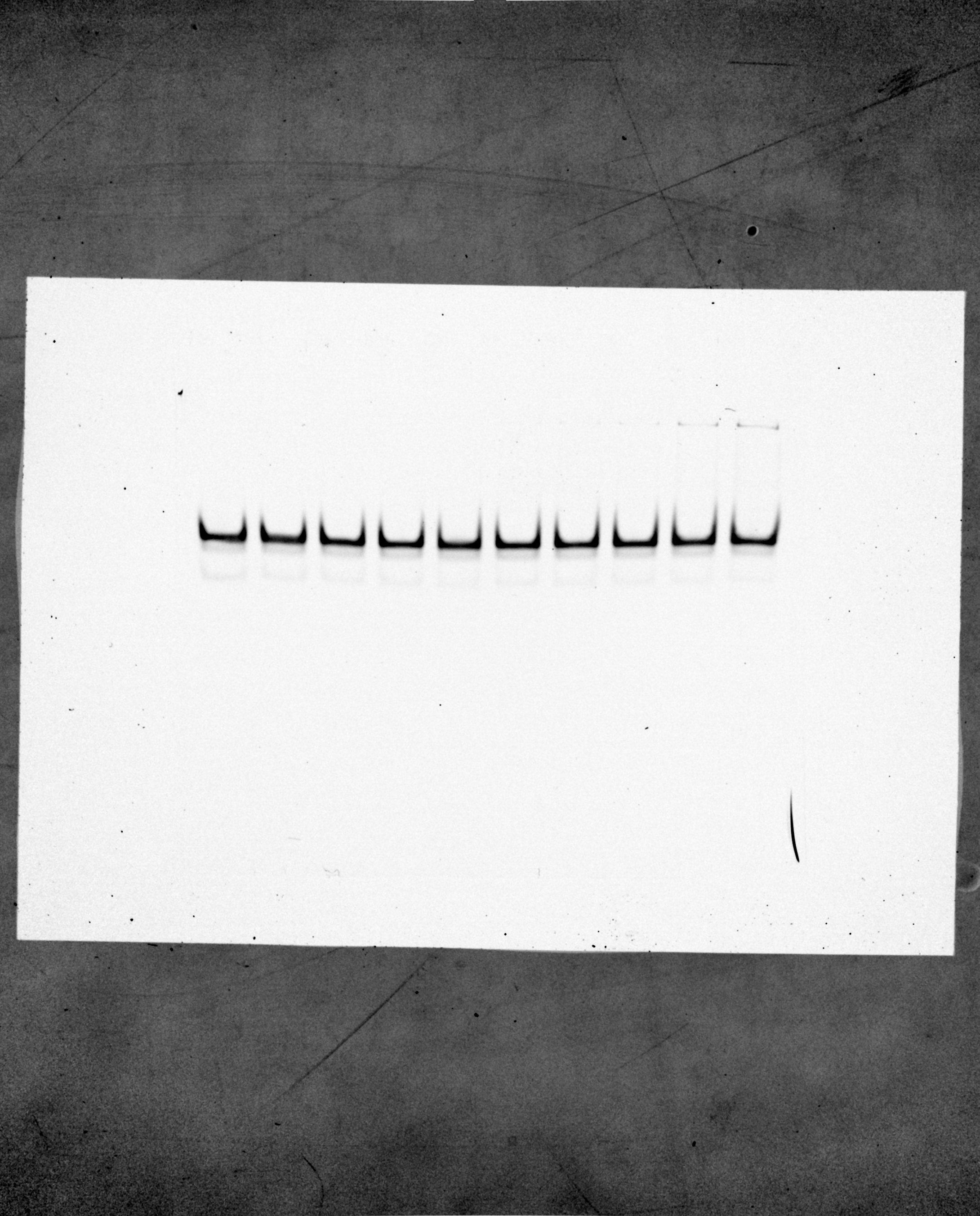

Supplement: Figure 3—source data 1. — Circular dichroism data (panel b), electrophoretic mobility shift assay (EMSA) images (panels c–h), flow cytometry data (panel j), and data analyses (panels c–f, j). [file elife-83538-fig3-data1.zip › Figure 3 - Source data 1/g/220207 Cy5 40bp EMSA with yKER middle_B_PUB_600.tif]

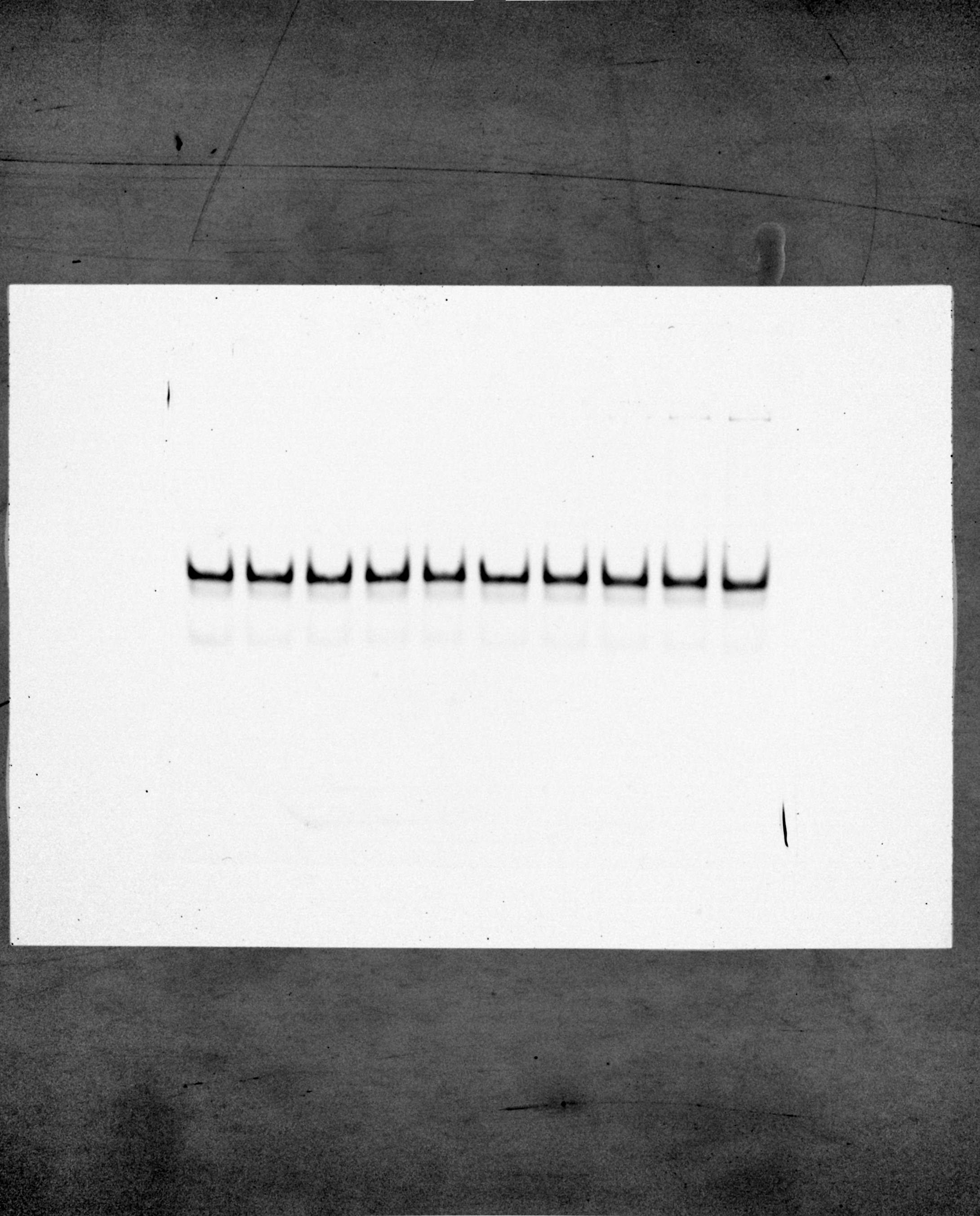

Supplement: Figure 3—source data 1. — Circular dichroism data (panel b), electrophoretic mobility shift assay (EMSA) images (panels c–h), flow cytometry data (panel j), and data analyses (panels c–f, j). [file elife-83538-fig3-data1.zip › Figure 3 - Source data 1/g/220208 Cy5 40bp EMSA with yKER middleB_PUB_600.tif]

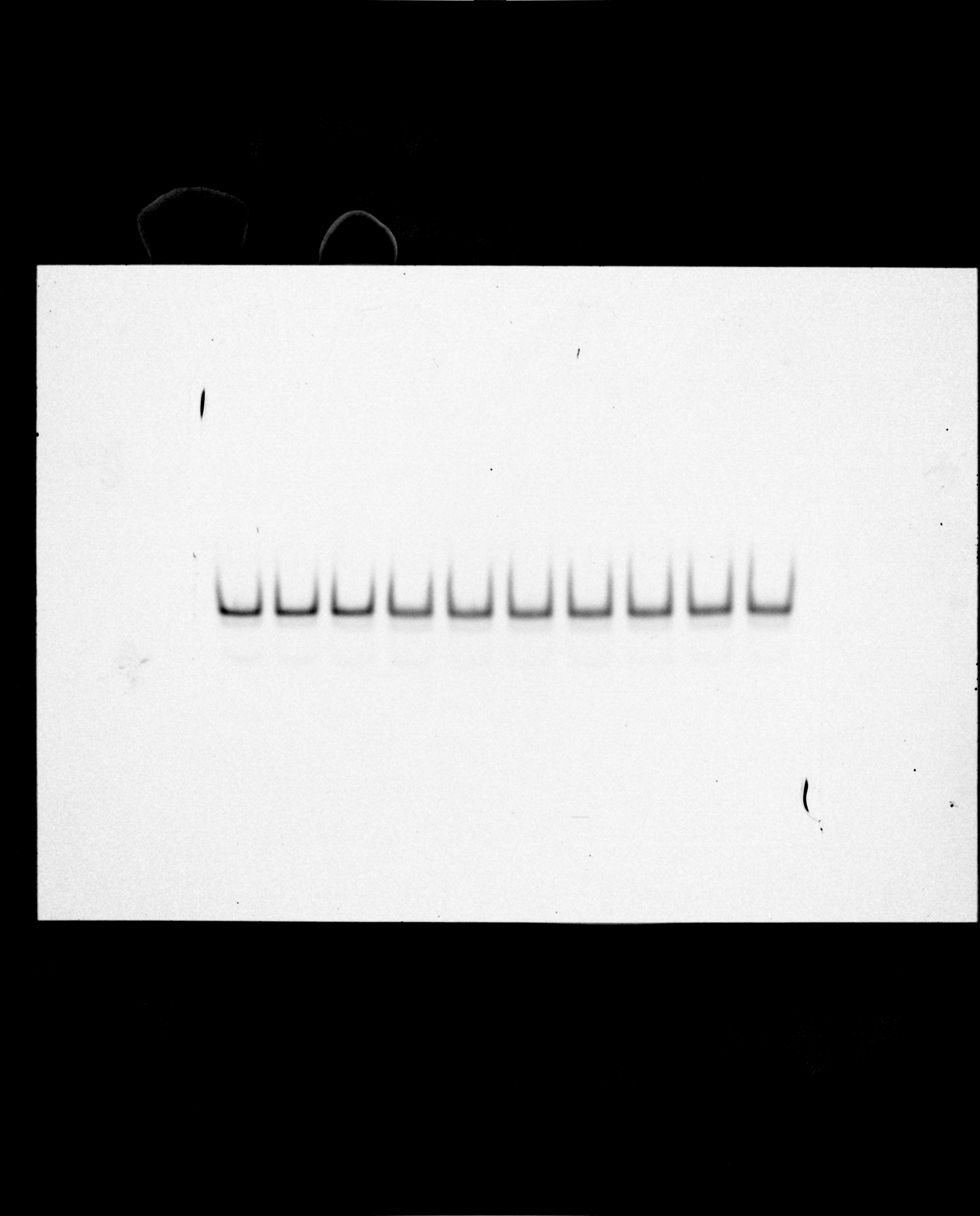

Supplement: Figure 3—source data 1. — Circular dichroism data (panel b), electrophoretic mobility shift assay (EMSA) images (panels c–h), flow cytometry data (panel j), and data analyses (panels c–f, j). [file elife-83538-fig3-data1.zip › Figure 3 - Source data 1/g/220204 Cy5 40bp EMSA with yKER middle_B_PUB_600.tif]

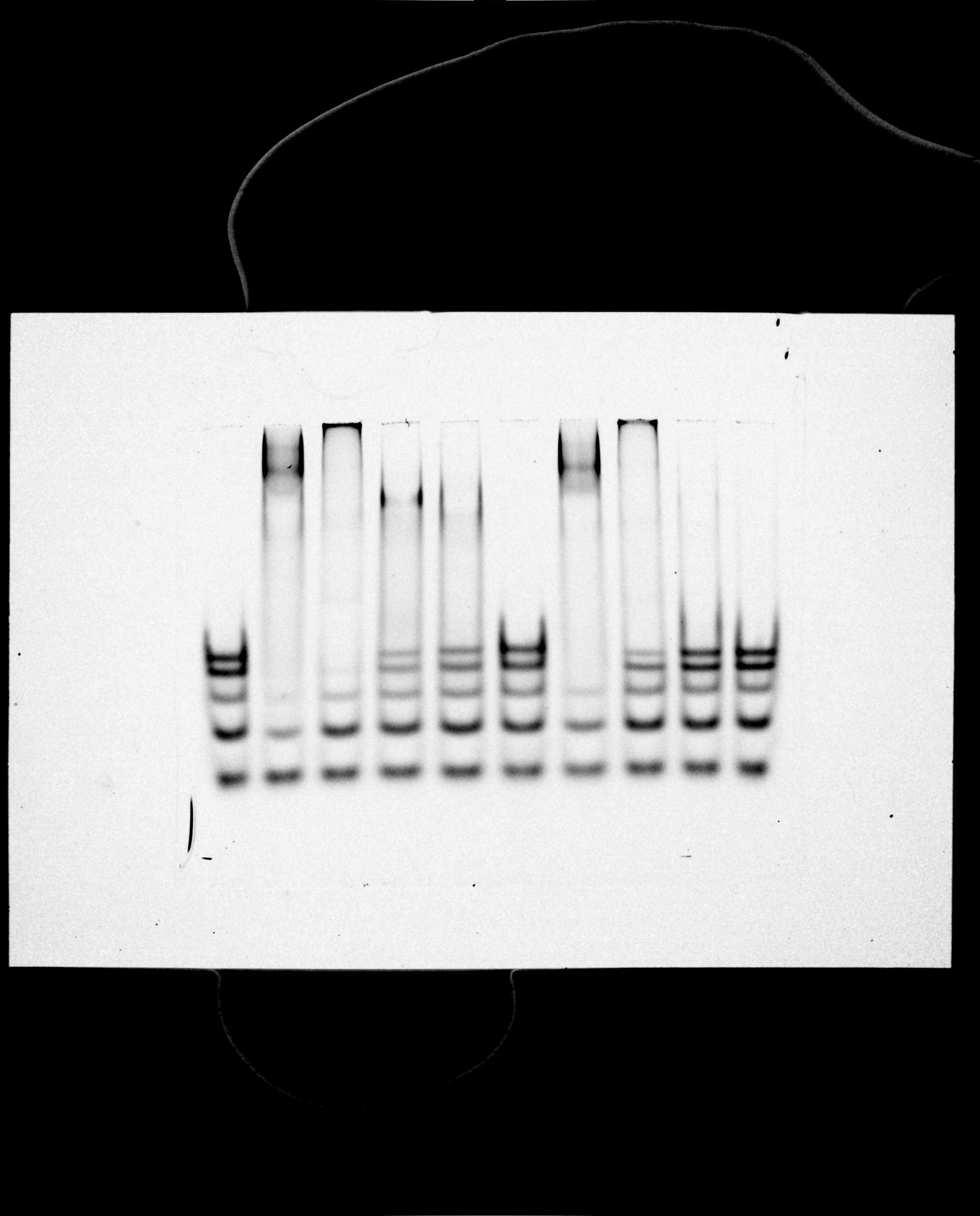

Supplement: Figure 3—source data 1. — Circular dichroism data (panel b), electrophoretic mobility shift assay (EMSA) images (panels c–h), flow cytometry data (panel j), and data analyses (panels c–f, j). [file elife-83538-fig3-data1.zip › Figure 3 - Source data 1/h/220325 Cy5 ladder EMSA with yCAF1 nobinders_n1_PUB_600.tif]

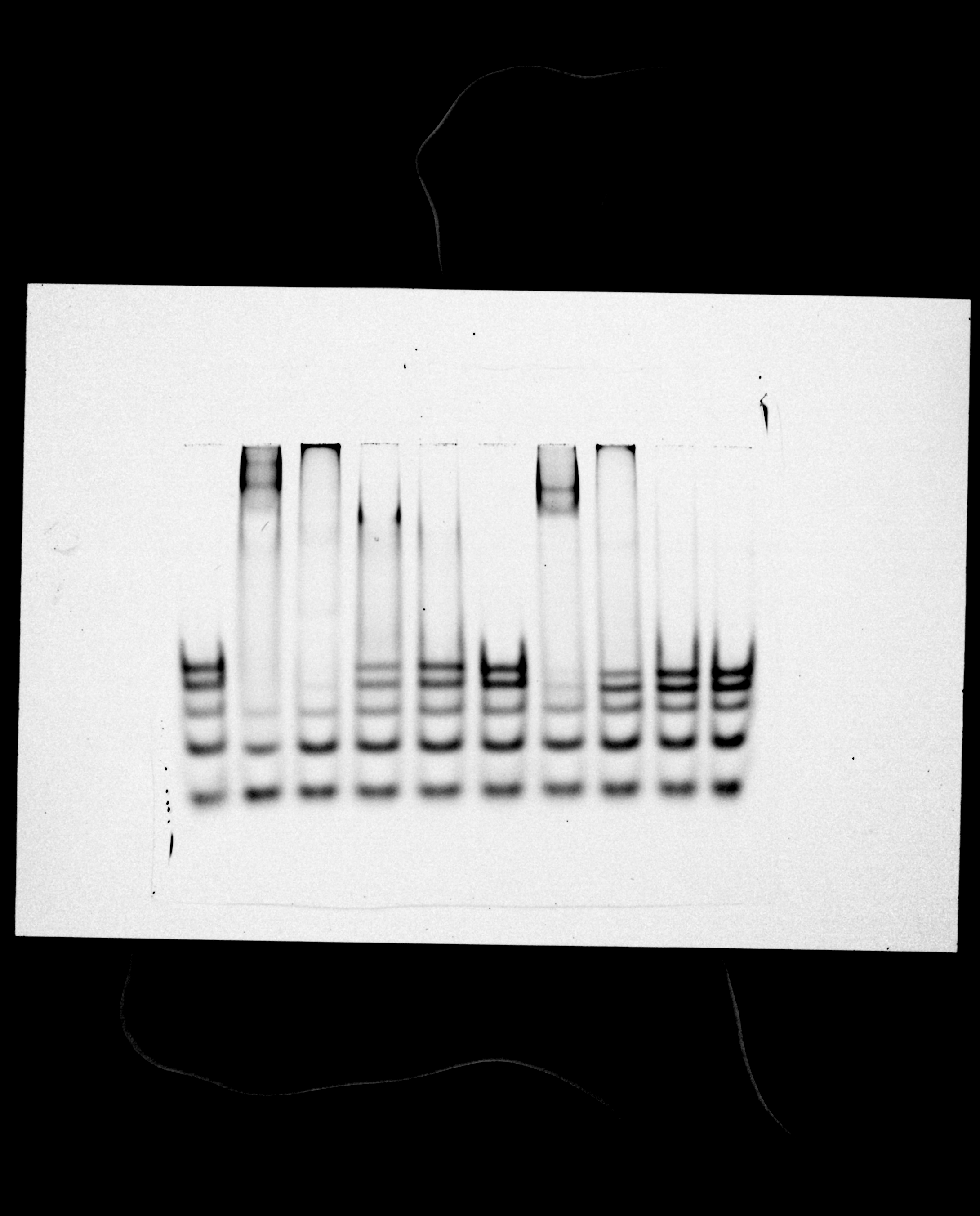

Supplement: Figure 3—source data 1. — Circular dichroism data (panel b), electrophoretic mobility shift assay (EMSA) images (panels c–h), flow cytometry data (panel j), and data analyses (panels c–f, j). [file elife-83538-fig3-data1.zip › Figure 3 - Source data 1/h/220325 Cy5 ladder EMSA with yCAF1 nobinders_n2_PUB_600.tif]

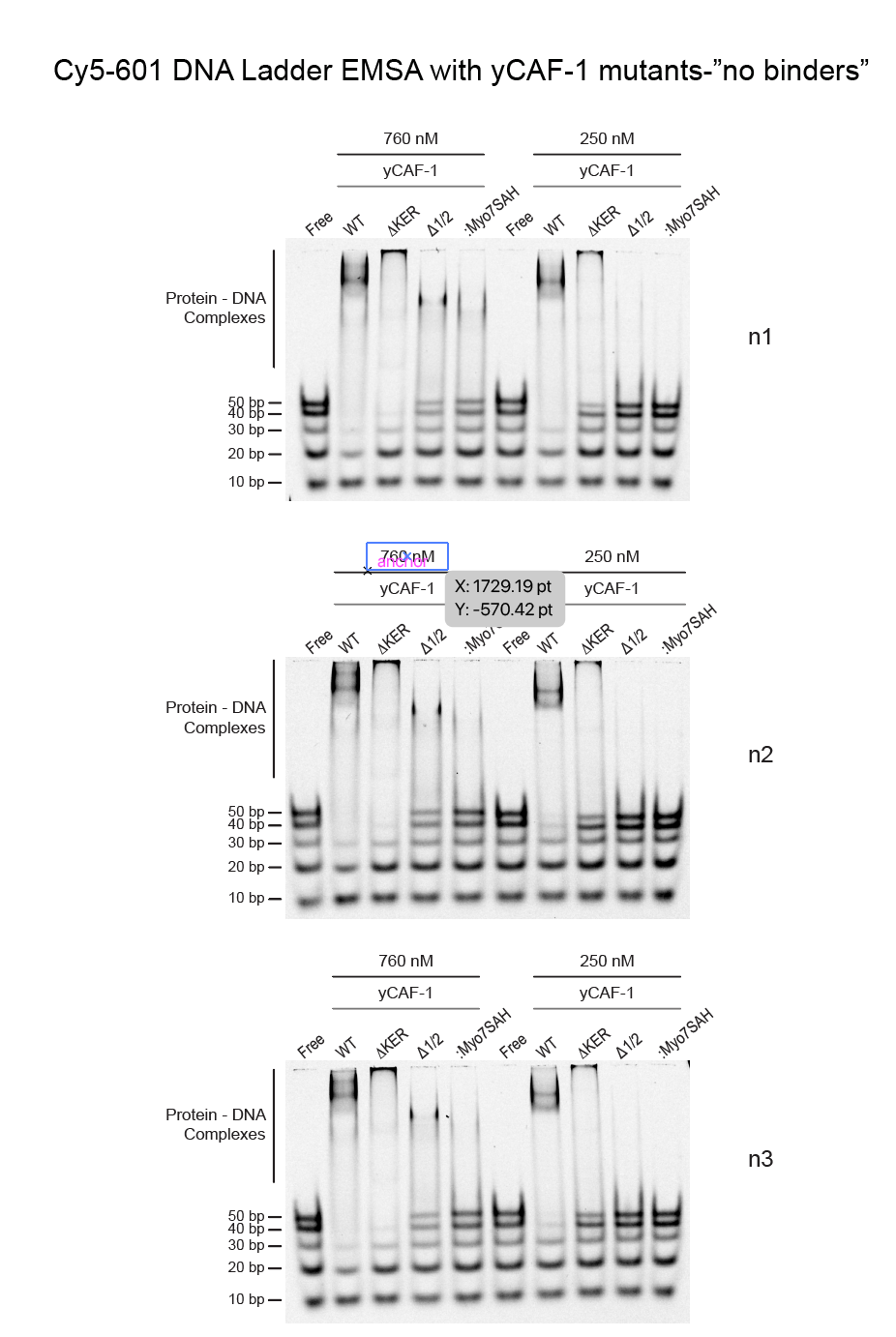

Supplement: Figure 3—source data 1. — Circular dichroism data (panel b), electrophoretic mobility shift assay (EMSA) images (panels c–h), flow cytometry data (panel j), and data analyses (panels c–f, j). [file elife-83538-fig3-data1.zip › Figure 3 - Source data 1/h/Annotation.png]

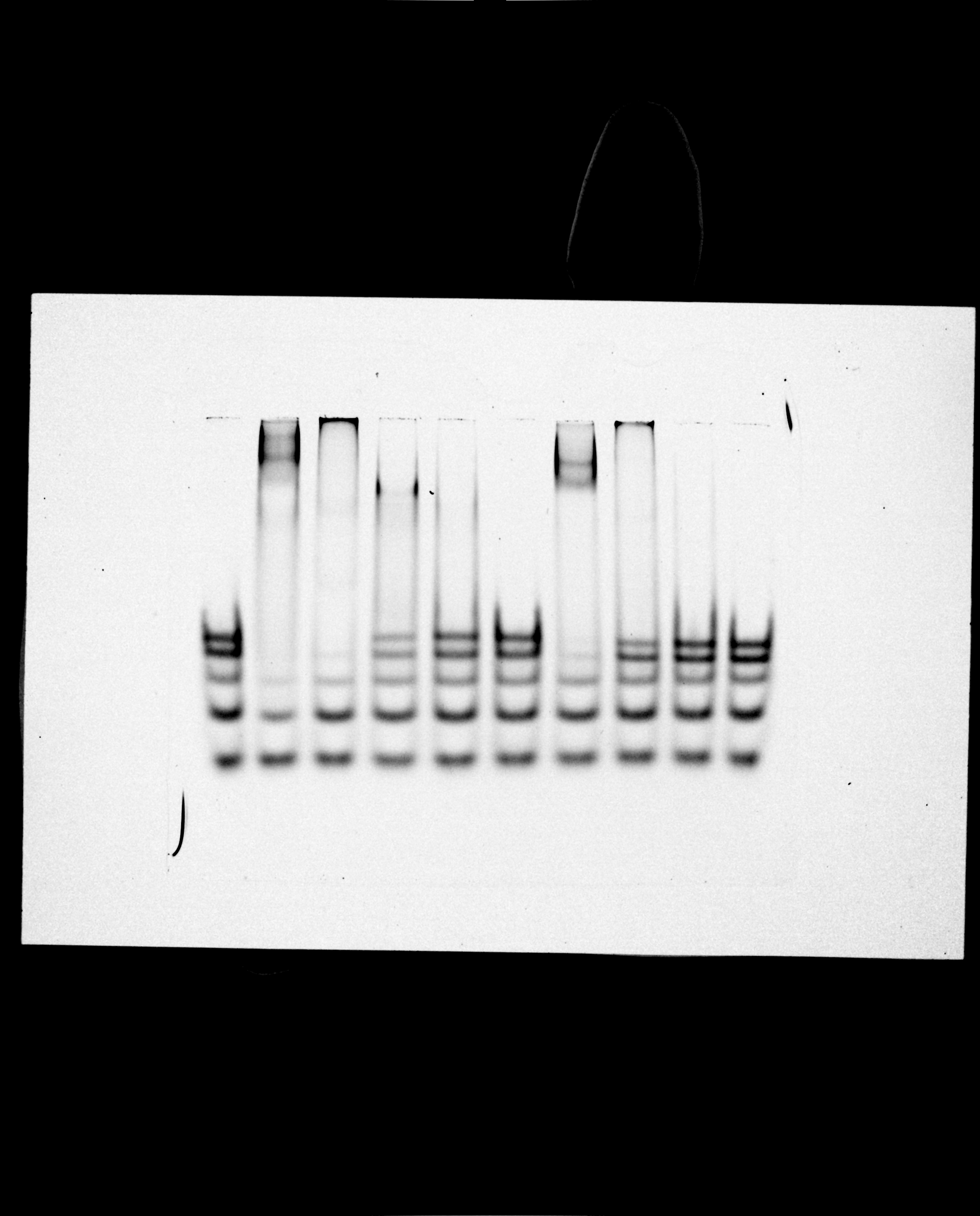

Supplement: Figure 3—source data 1. — Circular dichroism data (panel b), electrophoretic mobility shift assay (EMSA) images (panels c–h), flow cytometry data (panel j), and data analyses (panels c–f, j). [file elife-83538-fig3-data1.zip › Figure 3 - Source data 1/h/220325 Cy5 ladder EMSA with yCAF1 nobinders_n3_PUB_600.tif]

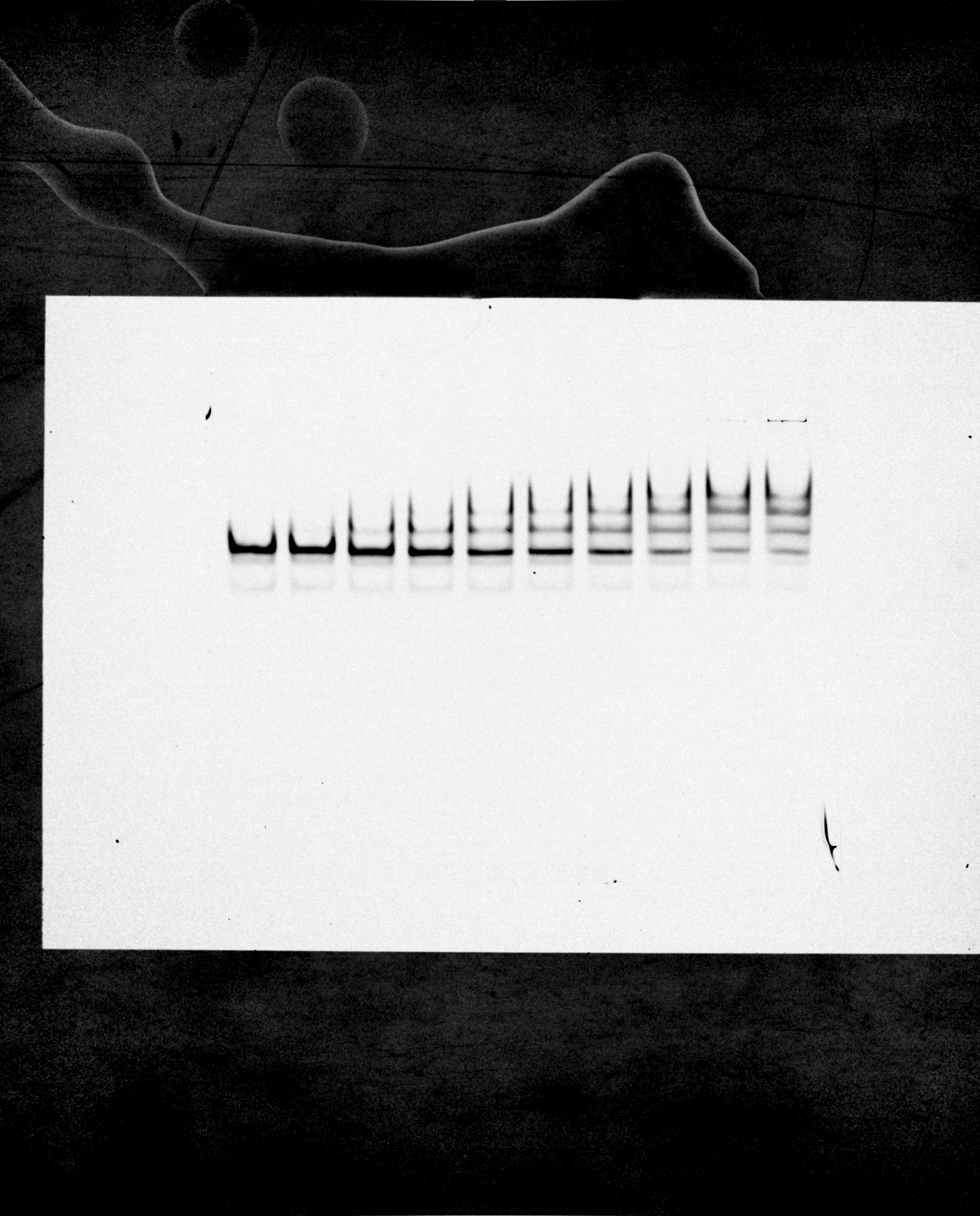

Supplement: Figure 3—source data 1. — Circular dichroism data (panel b), electrophoretic mobility shift assay (EMSA) images (panels c–h), flow cytometry data (panel j), and data analyses (panels c–f, j). [file elife-83538-fig3-data1.zip › Figure 3 - Source data 1/d/220207 Cy5 40bp EMSA with yKER middle_A_PUB_600.tif]

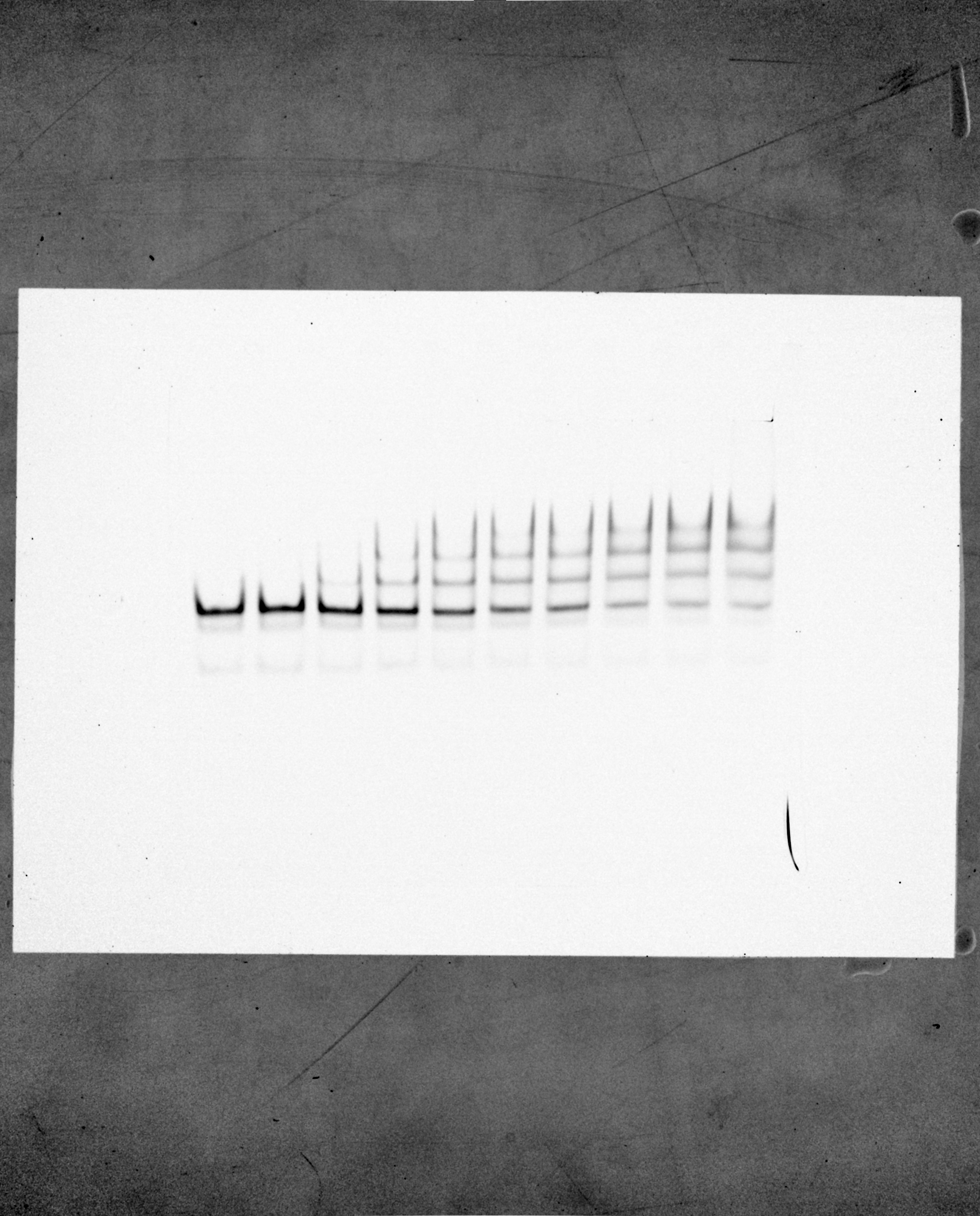

Supplement: Figure 3—source data 1. — Circular dichroism data (panel b), electrophoretic mobility shift assay (EMSA) images (panels c–h), flow cytometry data (panel j), and data analyses (panels c–f, j). [file elife-83538-fig3-data1.zip › Figure 3 - Source data 1/d/220208 Cy5 40bp EMSA with yKER middleA_n2_PUB_600.tif]

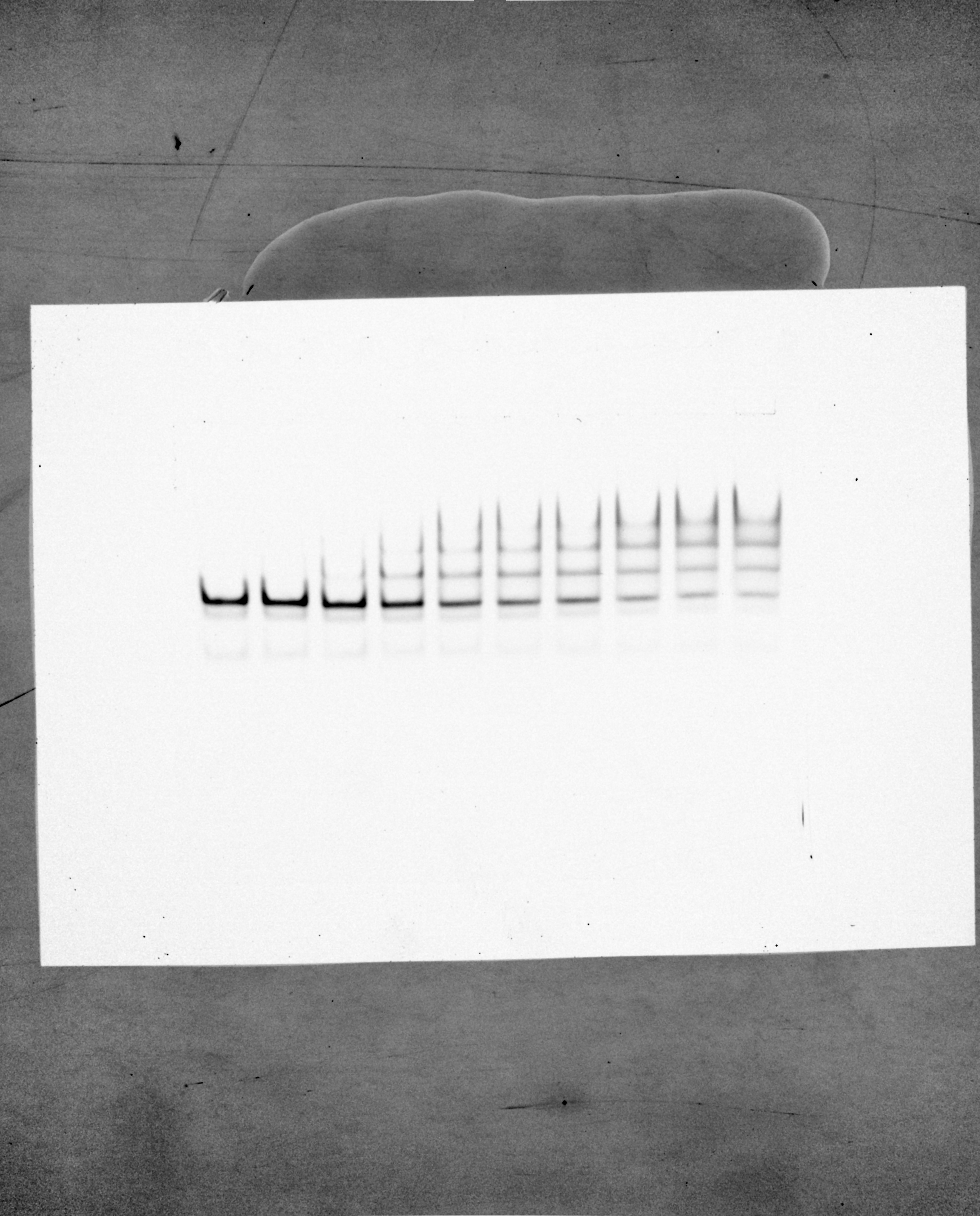

Supplement: Figure 3—source data 1. — Circular dichroism data (panel b), electrophoretic mobility shift assay (EMSA) images (panels c–h), flow cytometry data (panel j), and data analyses (panels c–f, j). [file elife-83538-fig3-data1.zip › Figure 3 - Source data 1/d/220208 Cy5 40bp EMSA with yKER middleA_n1_PUB_600.tif]

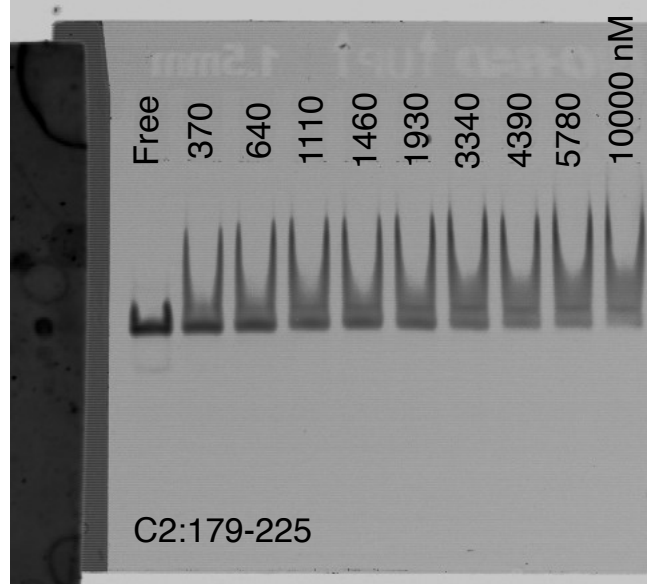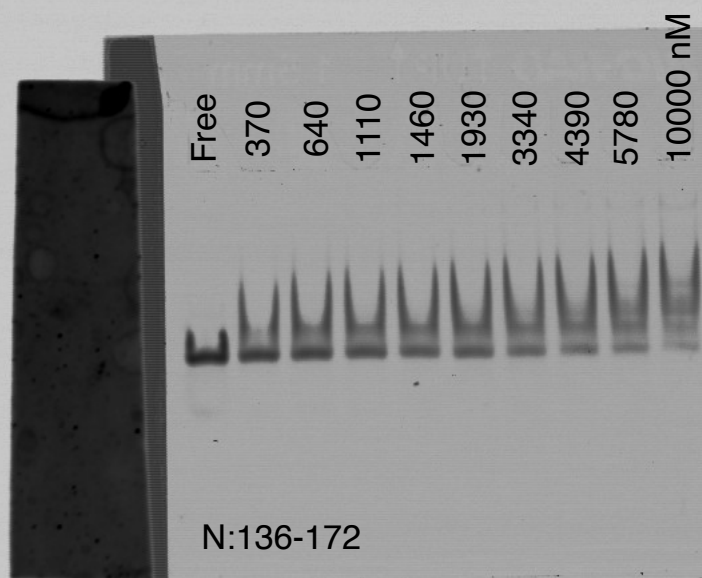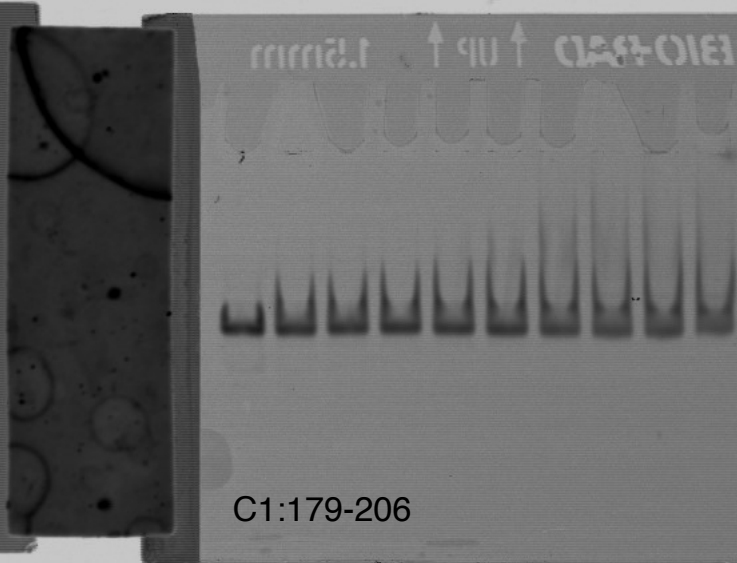

Supplement: Figure 3—source data 1. — Circular dichroism data (panel b), electrophoretic mobility shift assay (EMSA) images (panels c–h), flow cytometry data (panel j), and data analyses (panels c–f, j). [file elife-83538-fig3-data1.zip › Figure 3 - Source data 1/e and f/Gel2_Annotated.pdf]

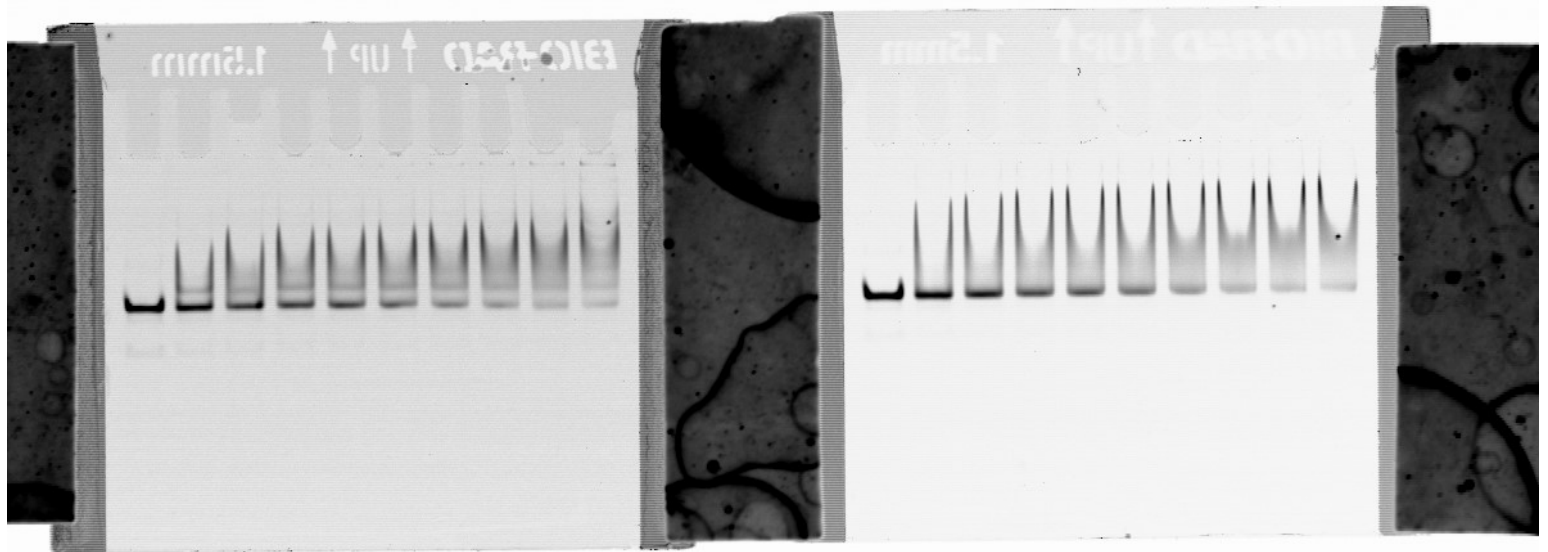

Supplement: Figure 3—source data 1. — Circular dichroism data (panel b), electrophoretic mobility shift assay (EMSA) images (panels c–h), flow cytometry data (panel j), and data analyses (panels c–f, j). [file elife-83538-fig3-data1.zip › Figure 3 - Source data 1/e and f/191125 EMSA KER N and C2 with Cy540bp 10per gel.pdf]

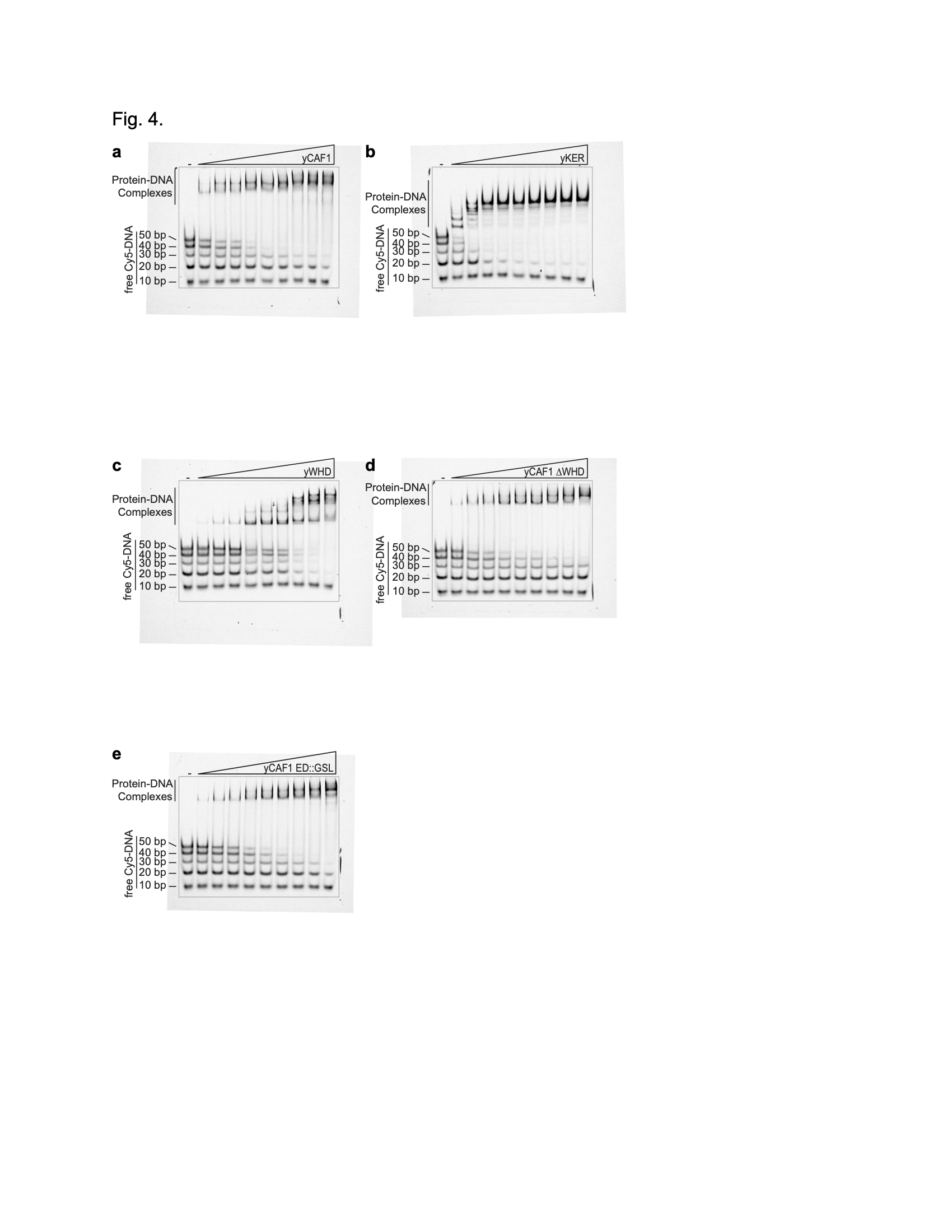

Supplement: Figure 4—source data 1. — Electrophoretic mobility shift assay (EMSA) images and data analyses. [file elife-83538-fig4-data1.zip › Figure 4 - Source data 1/Figure 4 - Source data 1_Gels Labeled.png]

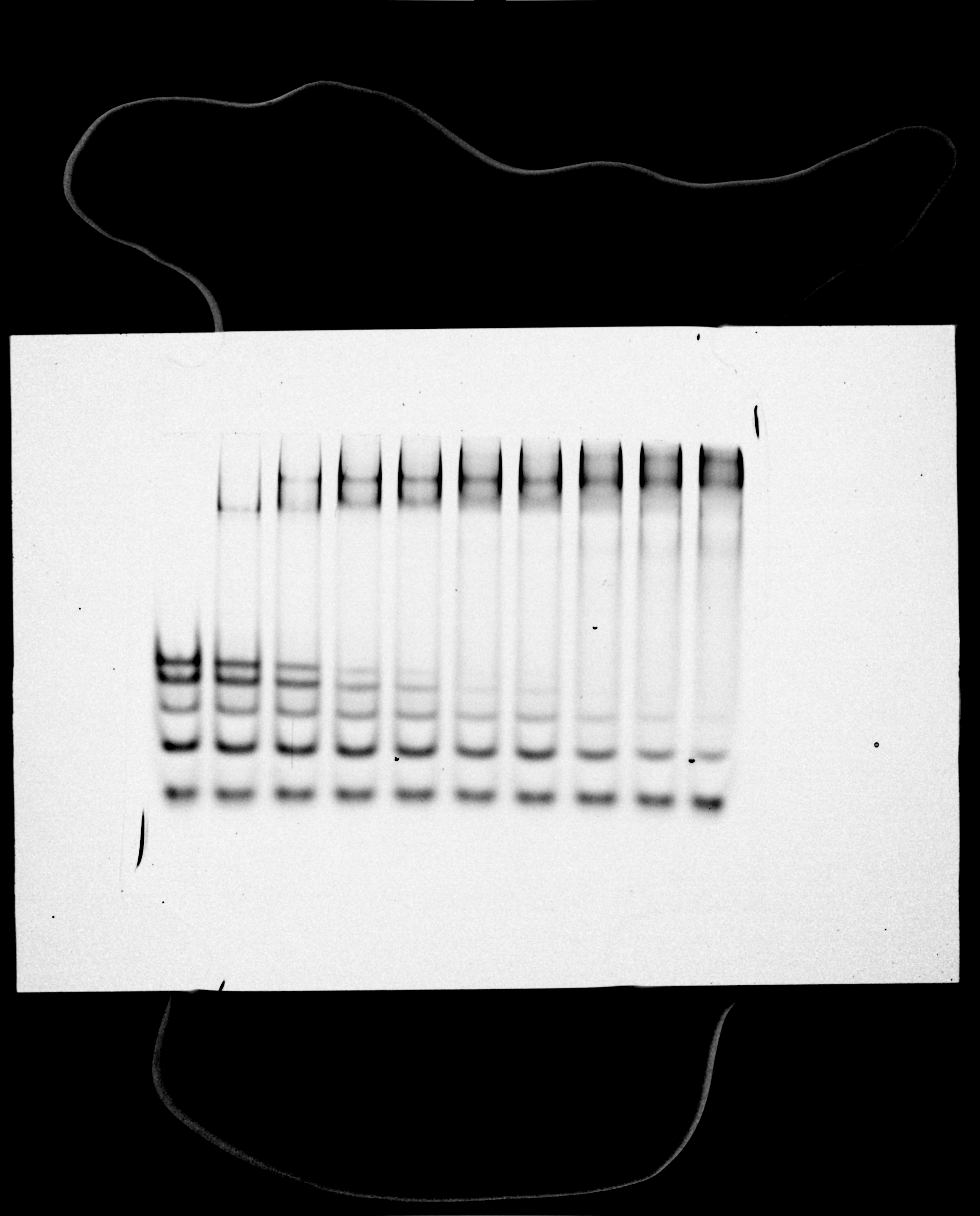

Supplement: Figure 4—source data 1. — Electrophoretic mobility shift assay (EMSA) images and data analyses. [file elife-83538-fig4-data1.zip › Figure 4 - Source data 1/a/220111 Cy5 ladder EMSA with yCAF1 WT_PUB_600.tif]

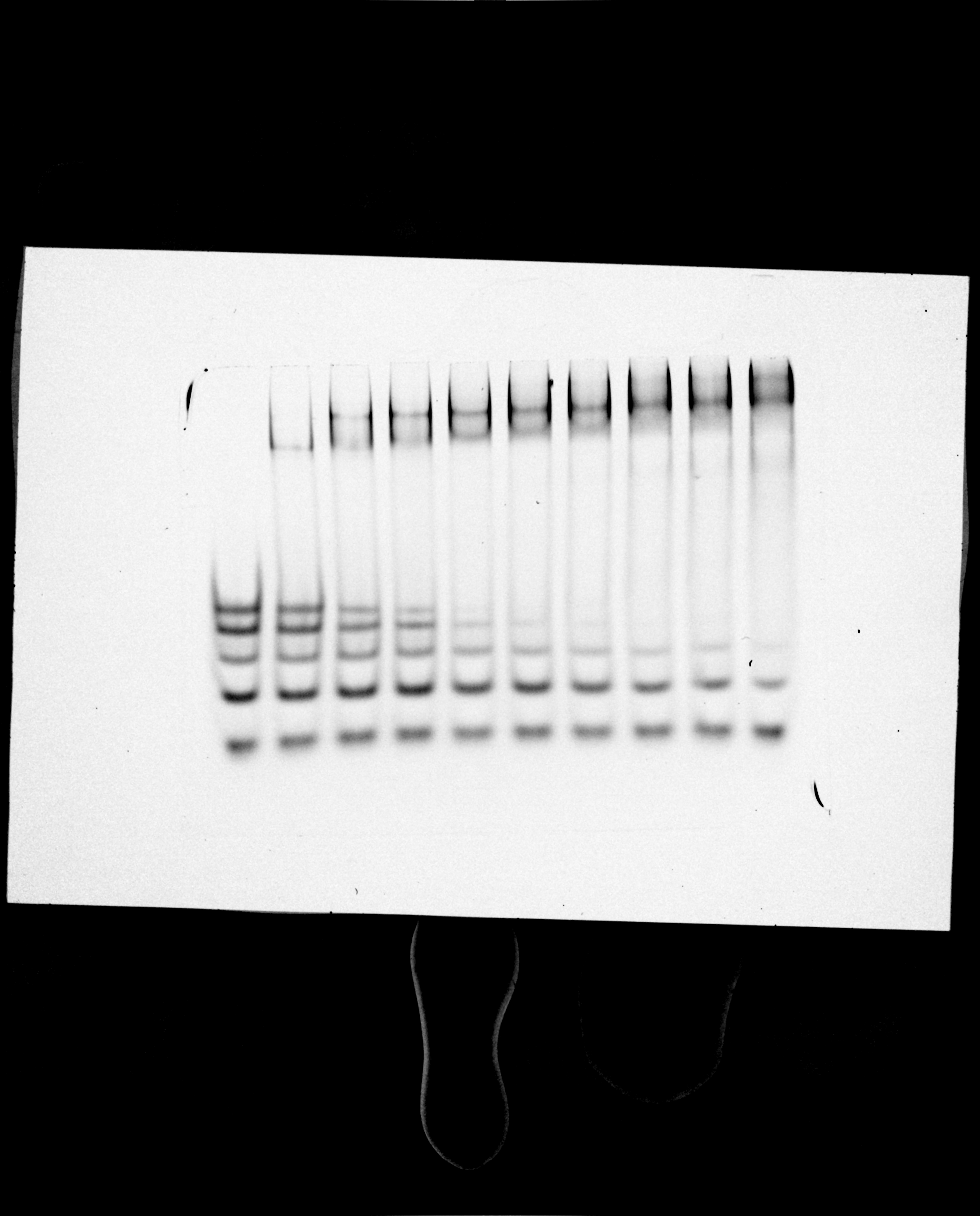

Supplement: Figure 4—source data 1. — Electrophoretic mobility shift assay (EMSA) images and data analyses. [file elife-83538-fig4-data1.zip › Figure 4 - Source data 1/a/220119 Cy5 Ladder EMSA withyCAF1 wild type_n2_PUB_600.tif]

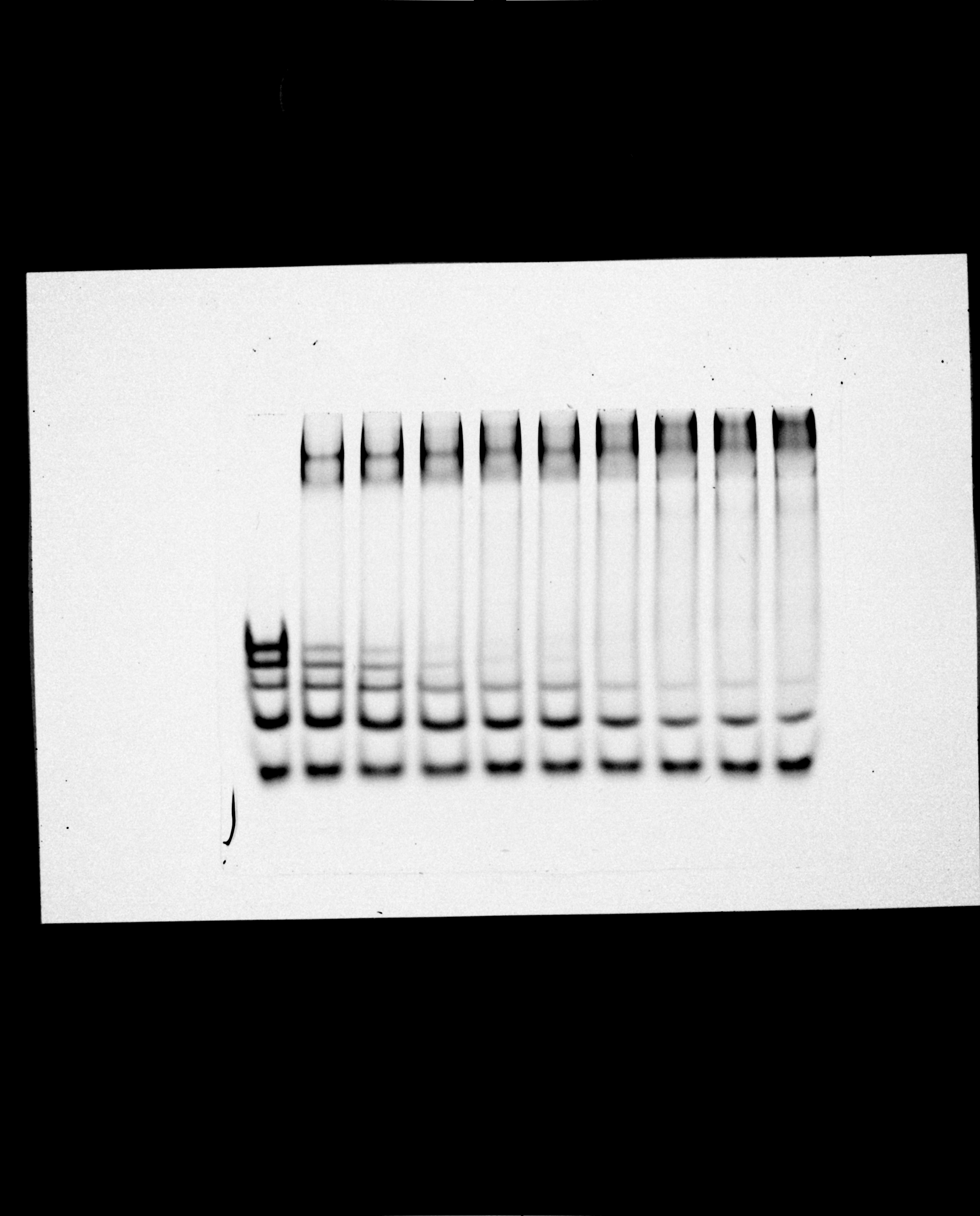

Supplement: Figure 4—source data 1. — Electrophoretic mobility shift assay (EMSA) images and data analyses. [file elife-83538-fig4-data1.zip › Figure 4 - Source data 1/a/211210 Cy5 ladder EMSA with yCAF1 WT_PUB_600.tif]

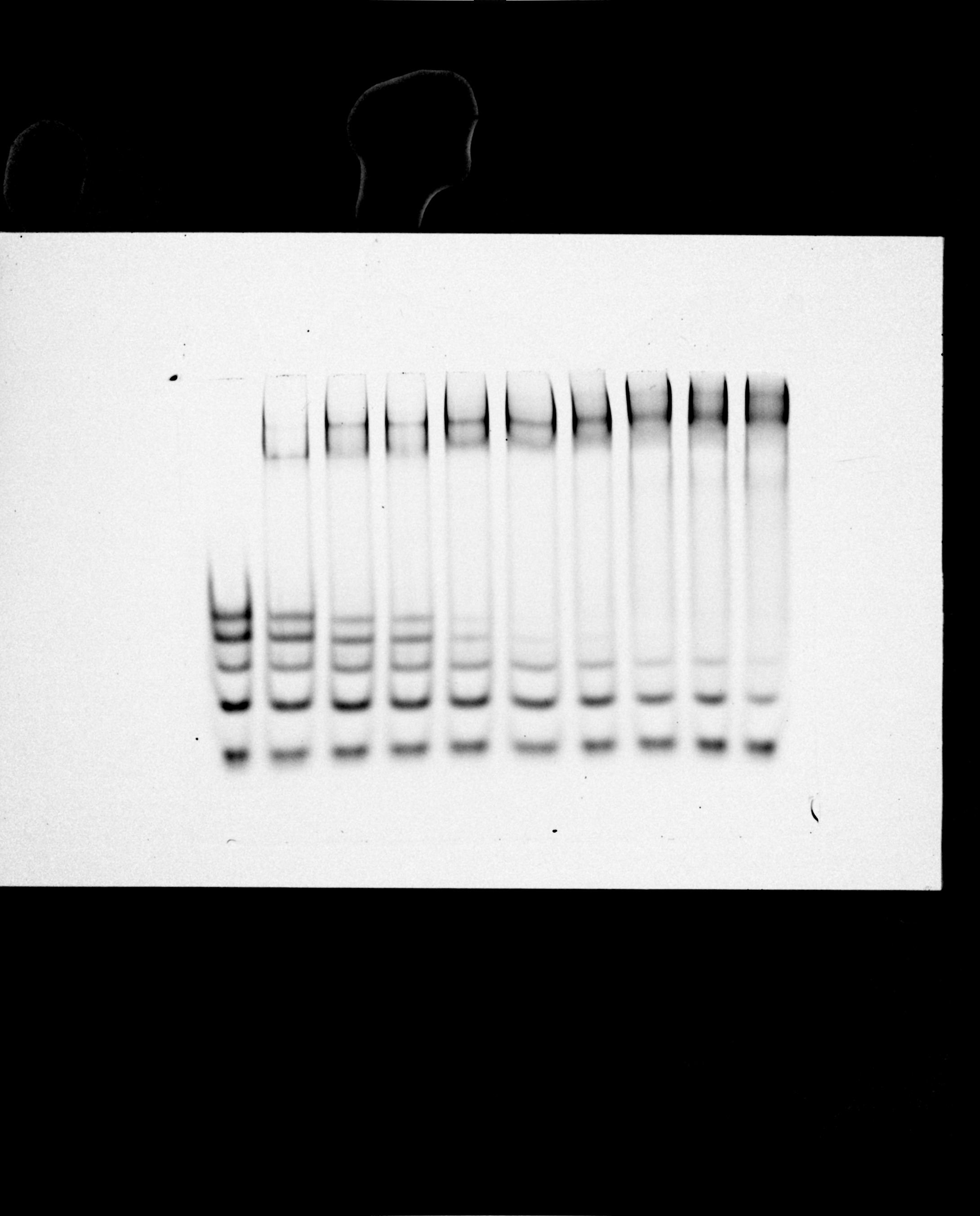

Supplement: Figure 4—source data 1. — Electrophoretic mobility shift assay (EMSA) images and data analyses. [file elife-83538-fig4-data1.zip › Figure 4 - Source data 1/a/220119 Cy5 Ladder EMSA withyCAF1 wild type_n1_PUB_600.tif]

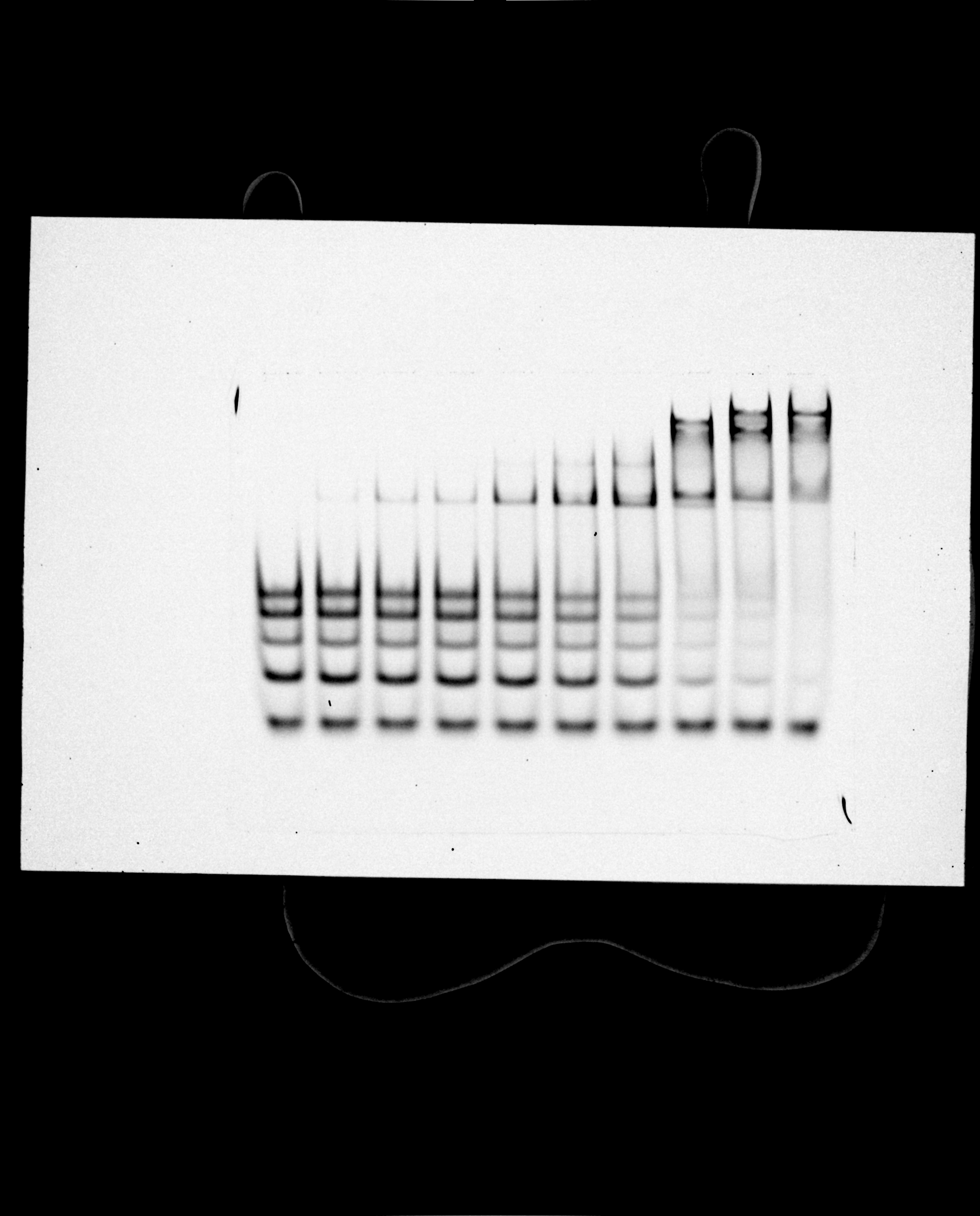

Supplement: Figure 4—source data 1. — Electrophoretic mobility shift assay (EMSA) images and data analyses. [file elife-83538-fig4-data1.zip › Figure 4 - Source data 1/c/220124 Cy5 ladder EMSA with yWHD_n2_PUB_600.tif]

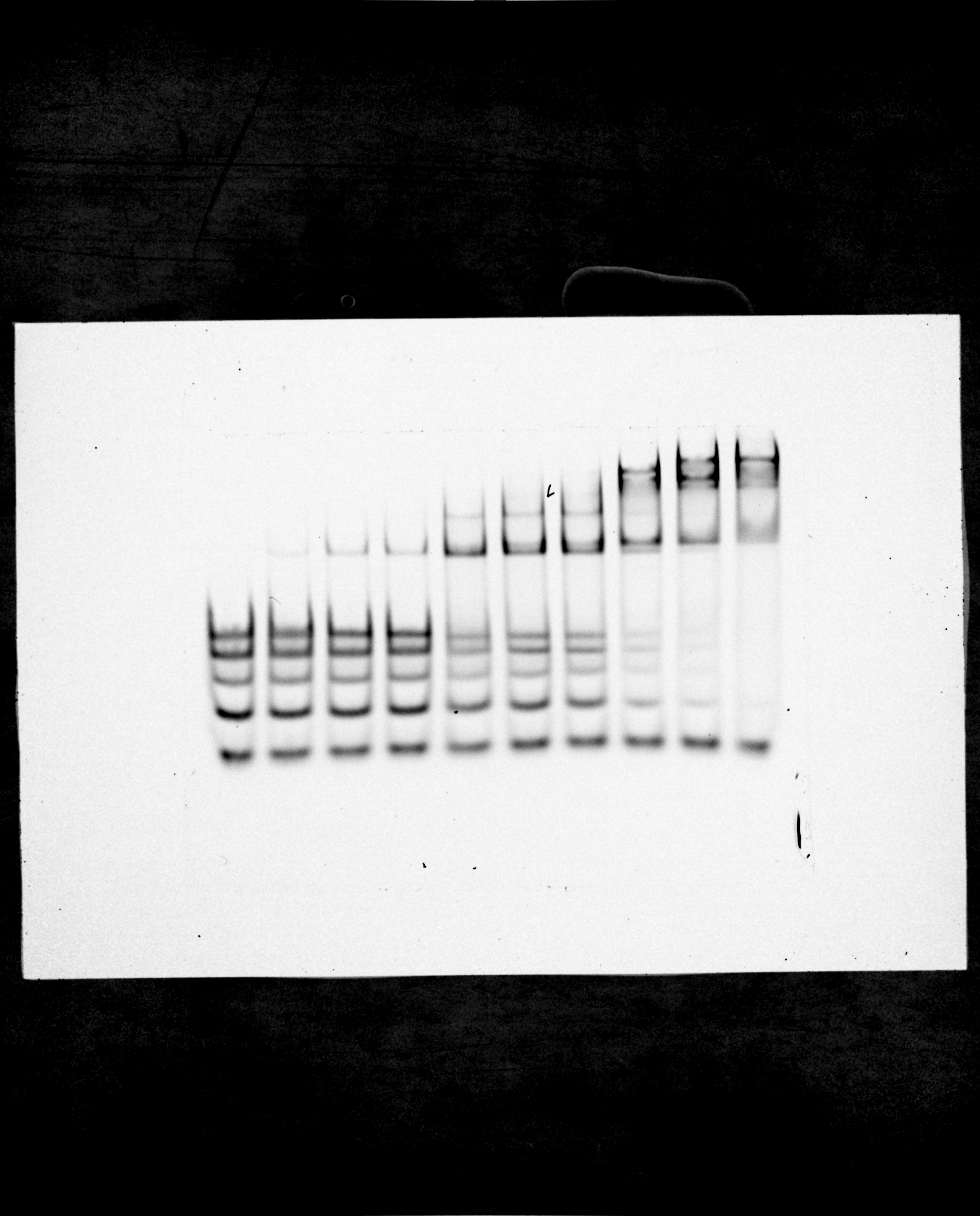

Supplement: Figure 4—source data 1. — Electrophoretic mobility shift assay (EMSA) images and data analyses. [file elife-83538-fig4-data1.zip › Figure 4 - Source data 1/c/220124 Cy5 ladder EMSA with yWHD_n1_PUB_600.tif]

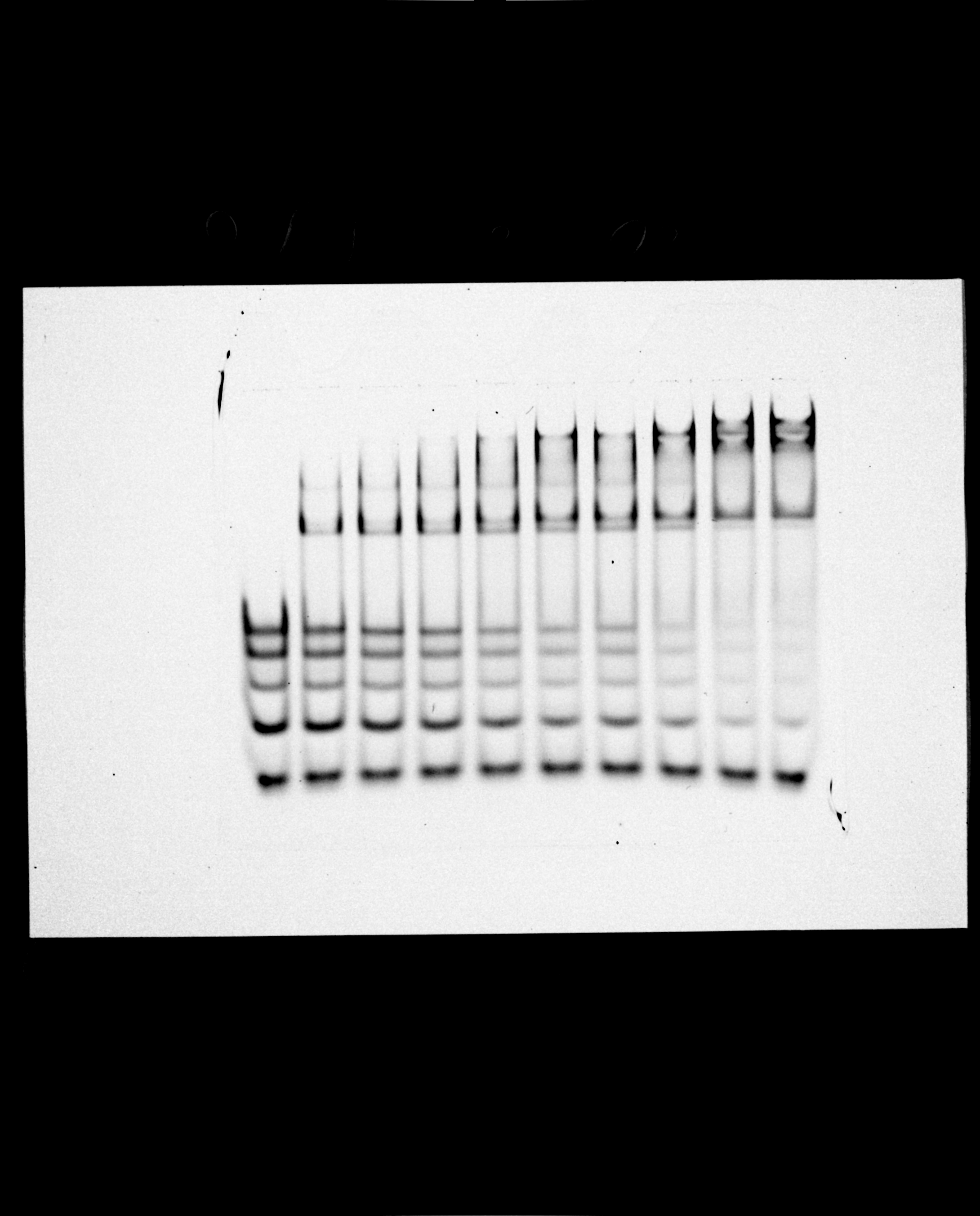

Supplement: Figure 4—source data 1. — Electrophoretic mobility shift assay (EMSA) images and data analyses. [file elife-83538-fig4-data1.zip › Figure 4 - Source data 1/c/211214 Cy5 ladder with yWHD_PUB_600.tif]

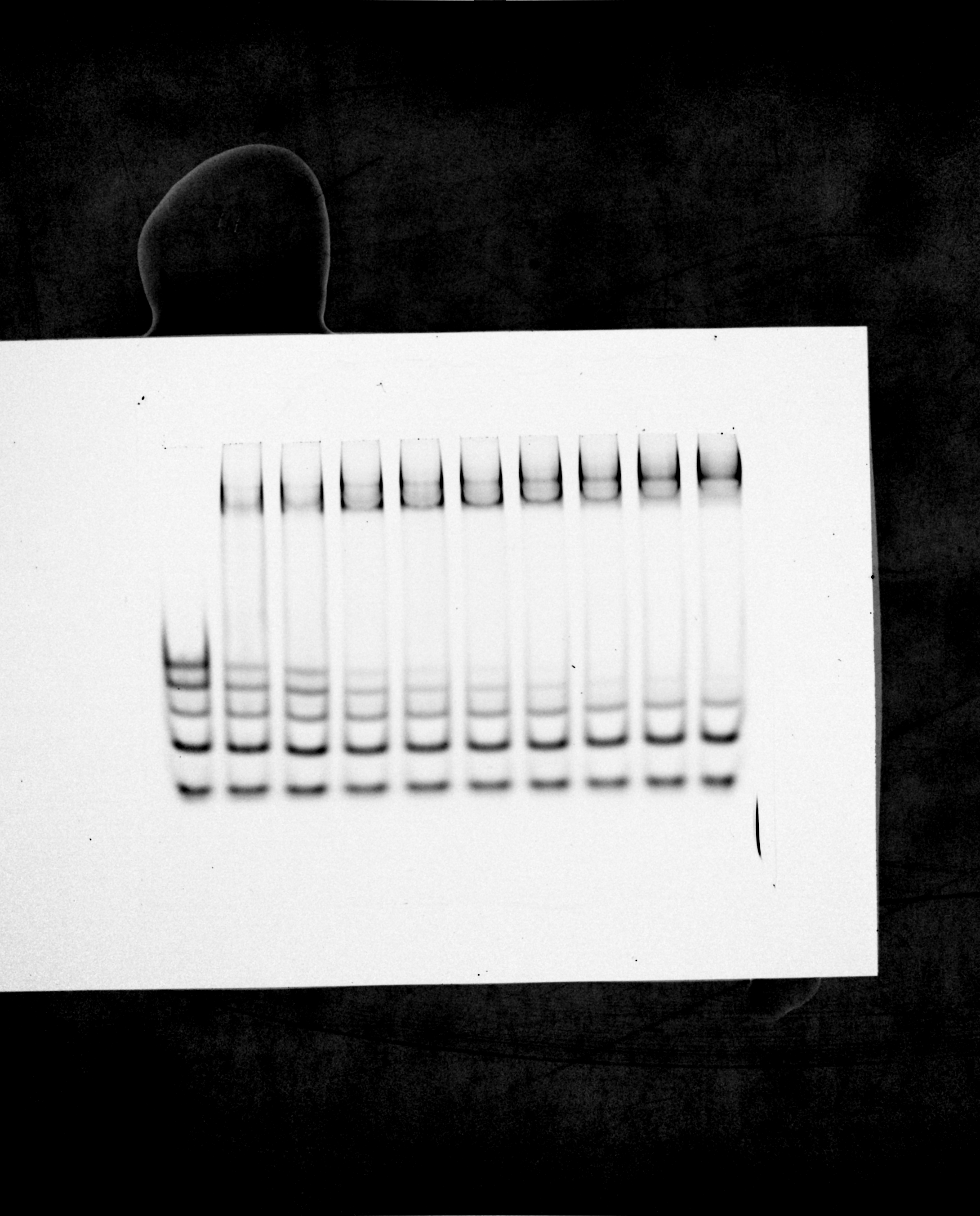

Supplement: Figure 4—source data 1. — Electrophoretic mobility shift assay (EMSA) images and data analyses. [file elife-83538-fig4-data1.zip › Figure 4 - Source data 1/d/211222 Cy5 ladder EMSA with yCAF1 deltaWHD_PUB_600.tif]

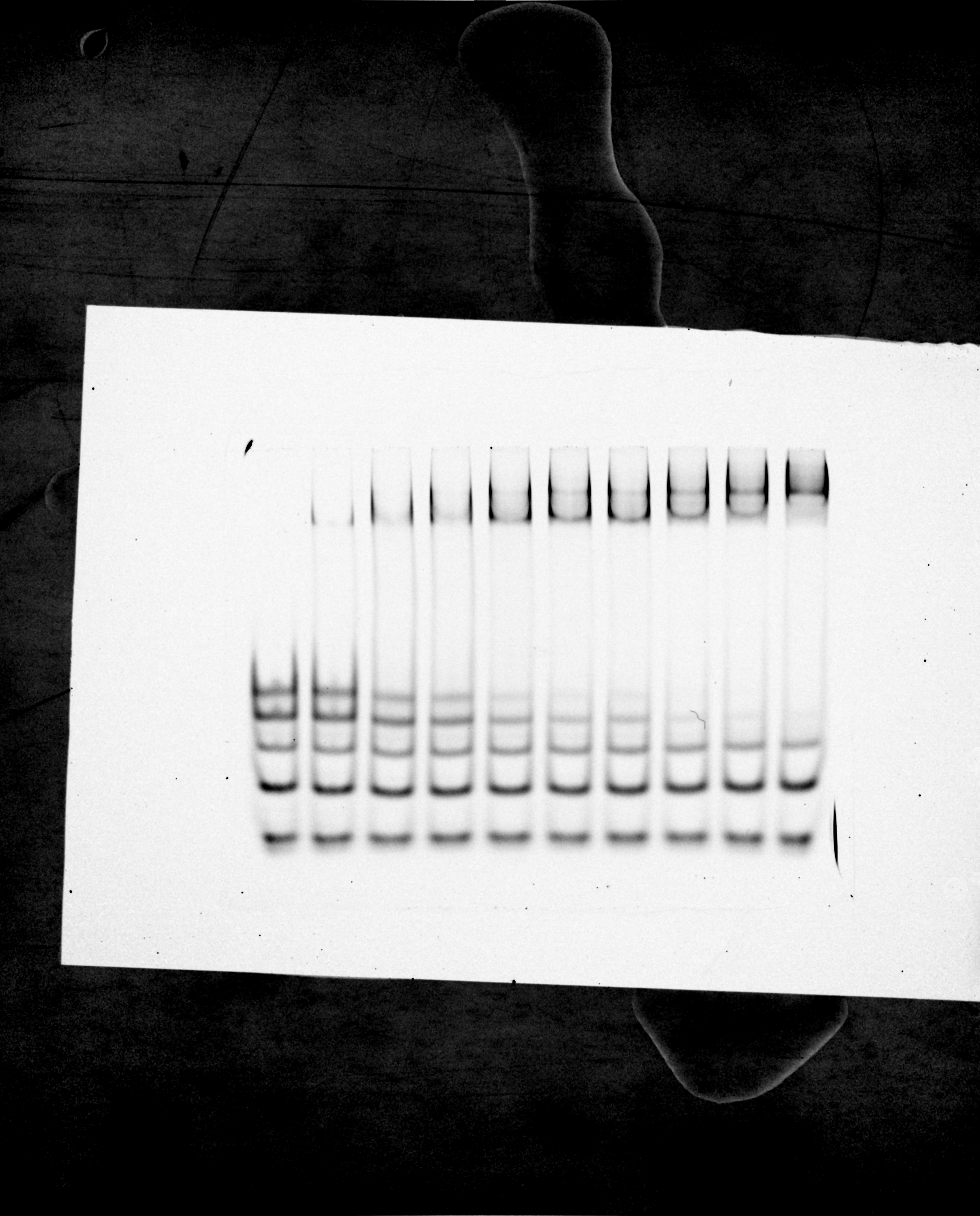

Supplement: Figure 4—source data 1. — Electrophoretic mobility shift assay (EMSA) images and data analyses. [file elife-83538-fig4-data1.zip › Figure 4 - Source data 1/d/220124 Cy5 Ladder EMSA with yCAF1 delta WHD_n1_PUB_600.tif]

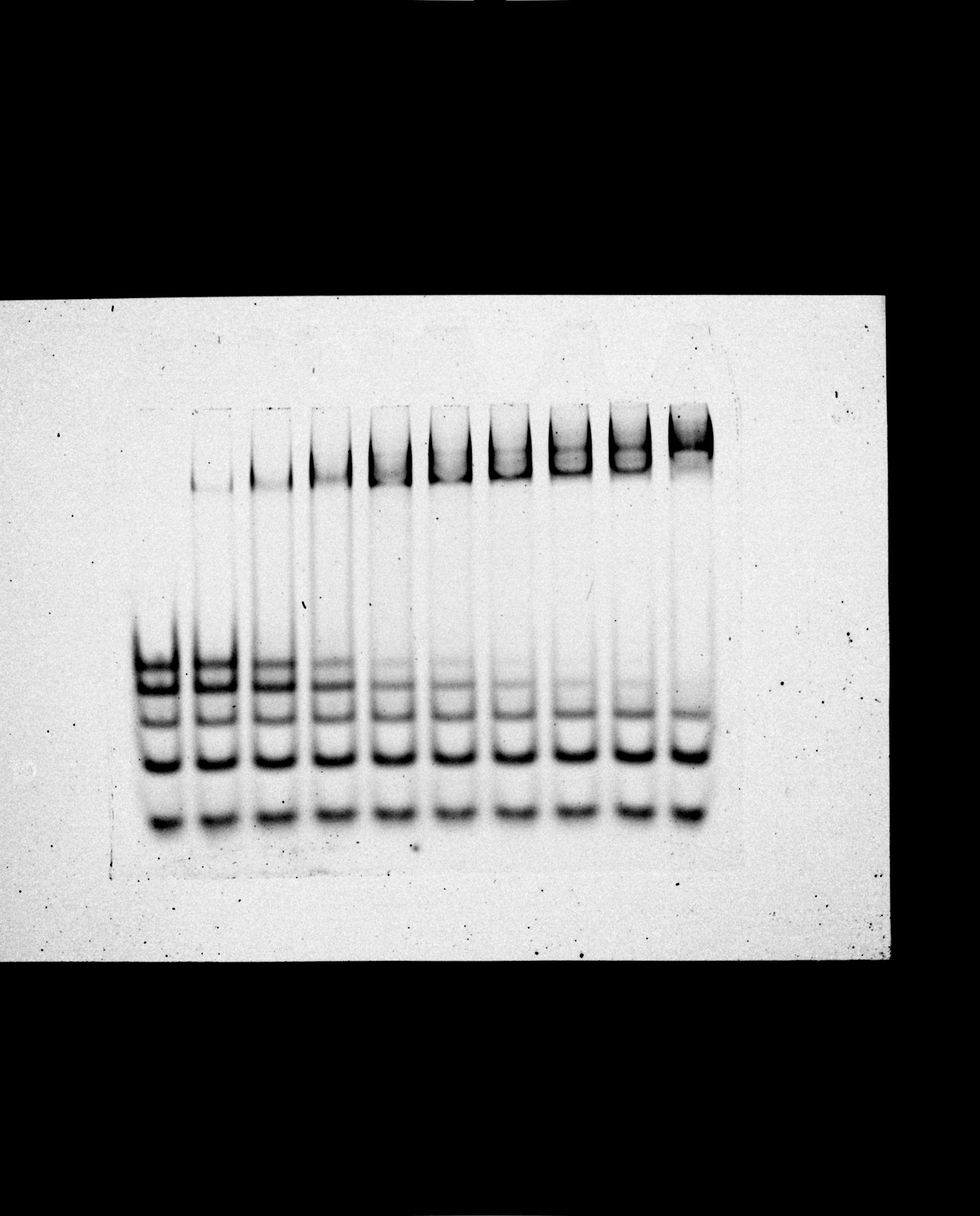

Supplement: Figure 4—source data 1. — Electrophoretic mobility shift assay (EMSA) images and data analyses. [file elife-83538-fig4-data1.zip › Figure 4 - Source data 1/d/220124 Cy5 Ladder EMSA with yCAF1 delta WHD_n2_PUB_600.tif]

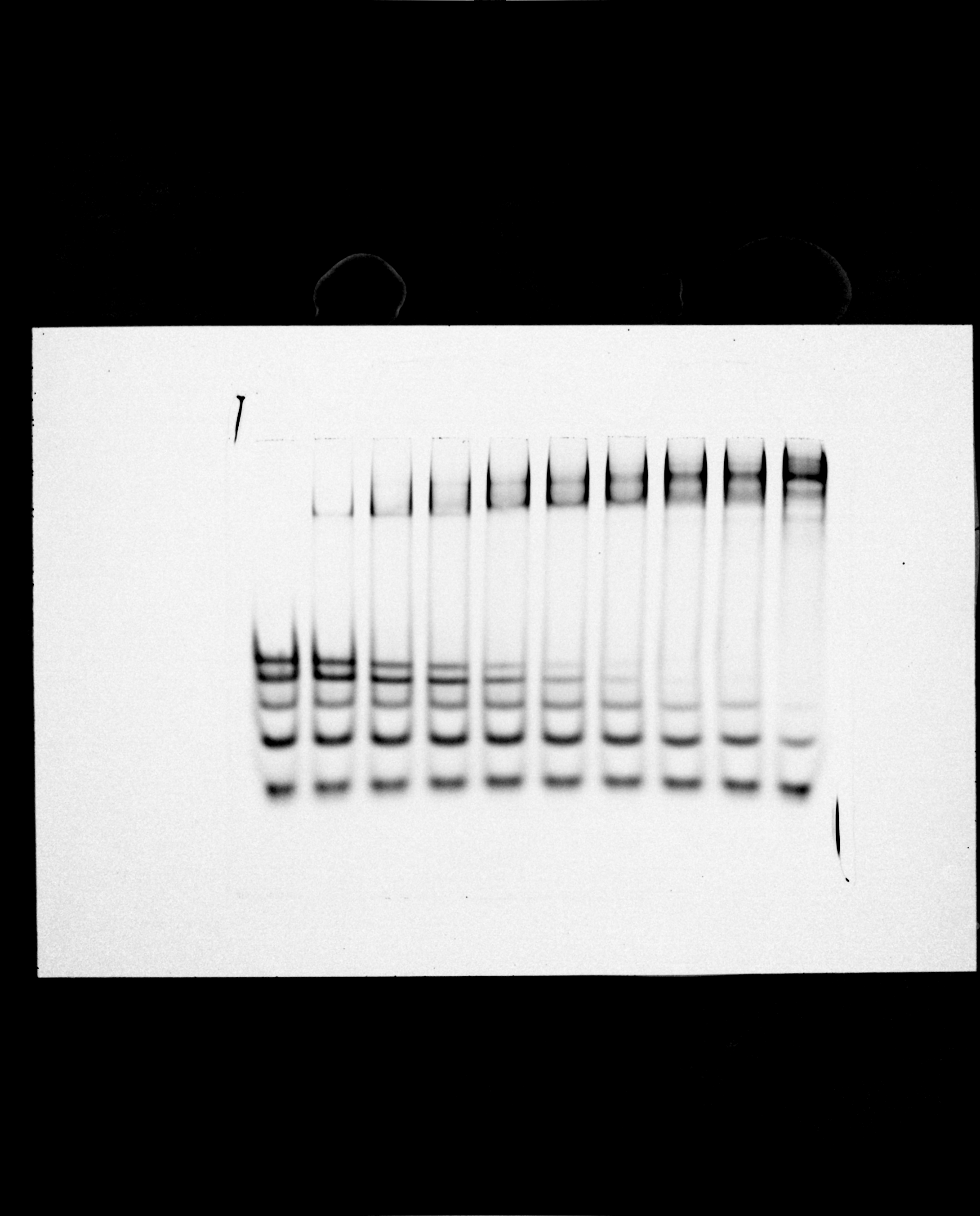

Supplement: Figure 4—source data 1. — Electrophoretic mobility shift assay (EMSA) images and data analyses. [file elife-83538-fig4-data1.zip › Figure 4 - Source data 1/e/220128 Cy5 ladder EMSA with yCAF1 EDtoGSL_n1_PUB_600.tif]

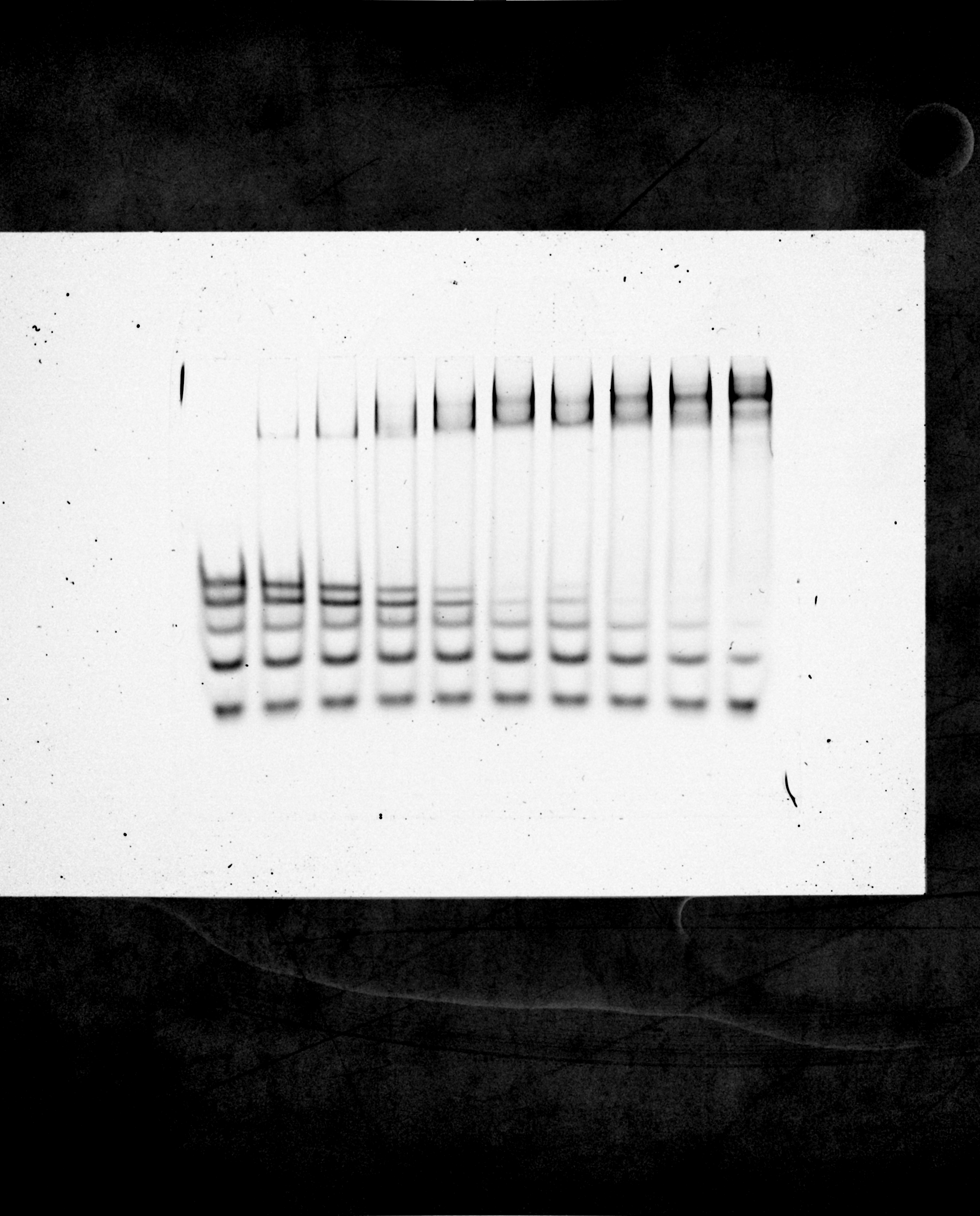

Supplement: Figure 4—source data 1. — Electrophoretic mobility shift assay (EMSA) images and data analyses. [file elife-83538-fig4-data1.zip › Figure 4 - Source data 1/e/220127 Cy5 ladder EMSA with yCAF1 EDtoGSL_n1_PUB_600.tif]

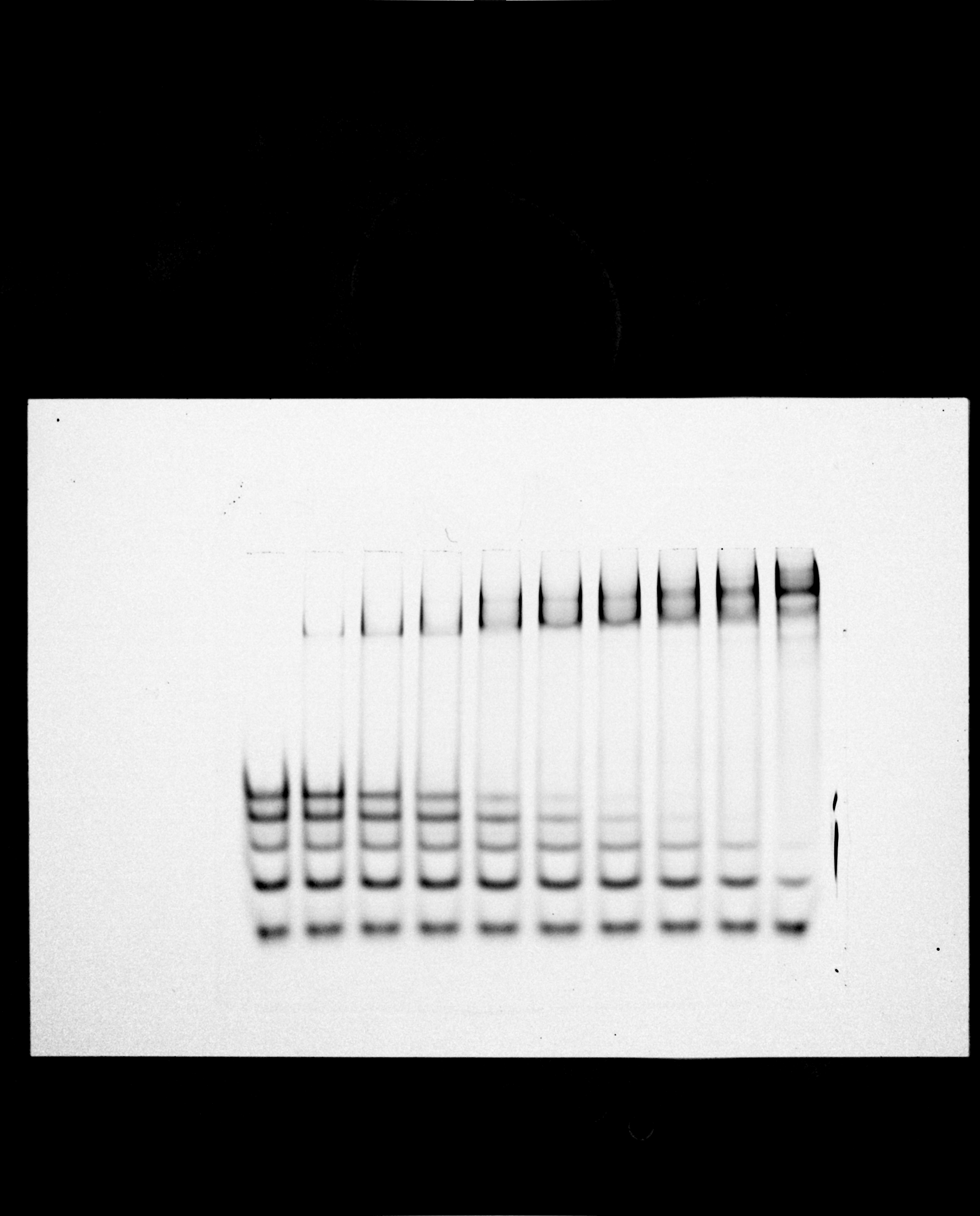

Supplement: Figure 4—source data 1. — Electrophoretic mobility shift assay (EMSA) images and data analyses. [file elife-83538-fig4-data1.zip › Figure 4 - Source data 1/e/220128 Cy5 ladder EMSA with yCAF1 EDtoGSL_n2_PUB_600.tif]

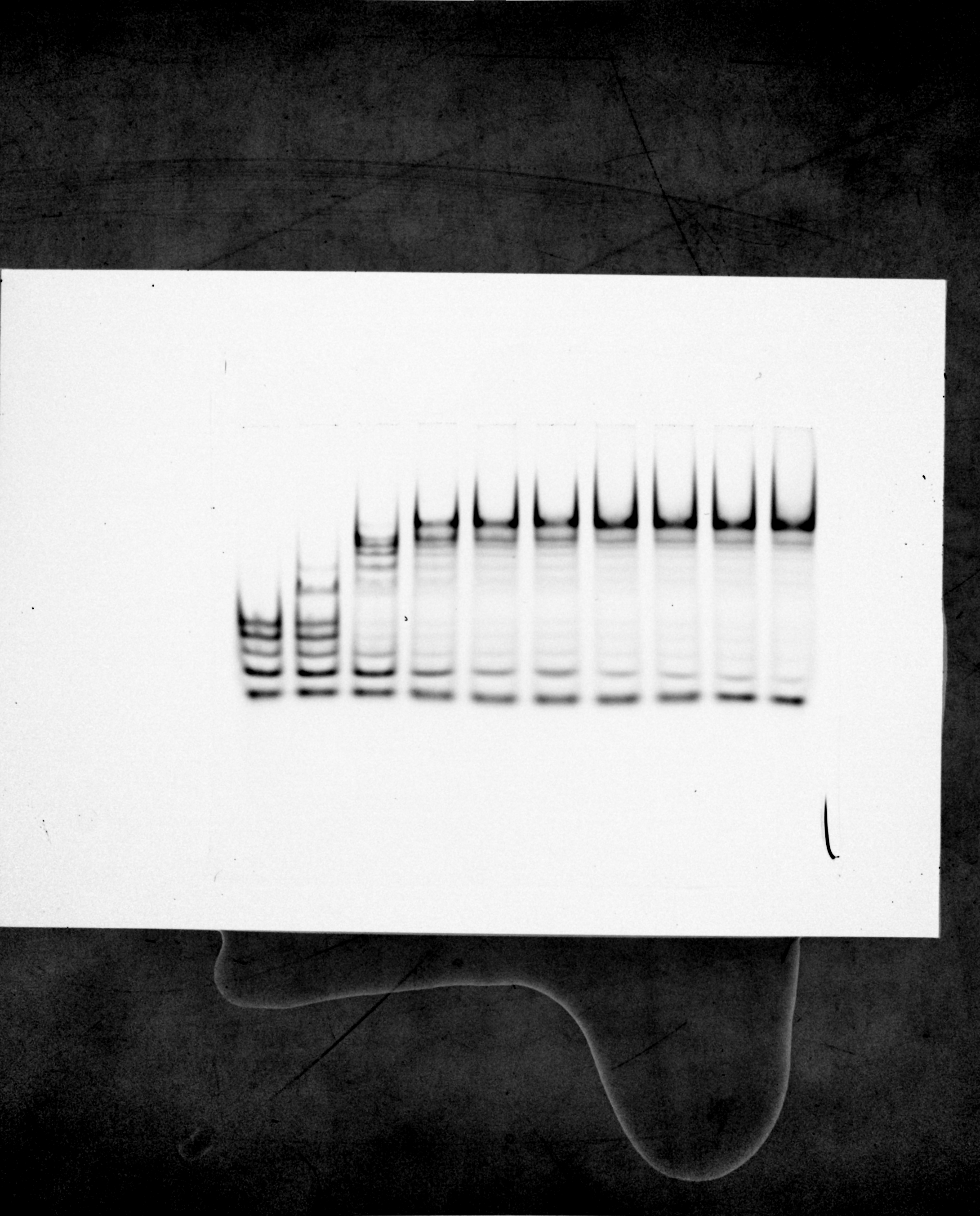

Supplement: Figure 4—source data 1. — Electrophoretic mobility shift assay (EMSA) images and data analyses. [file elife-83538-fig4-data1.zip › Figure 4 - Source data 1/b/220120 Cy5 ladder EMSA with yKER_n2_PUB_600.tif]

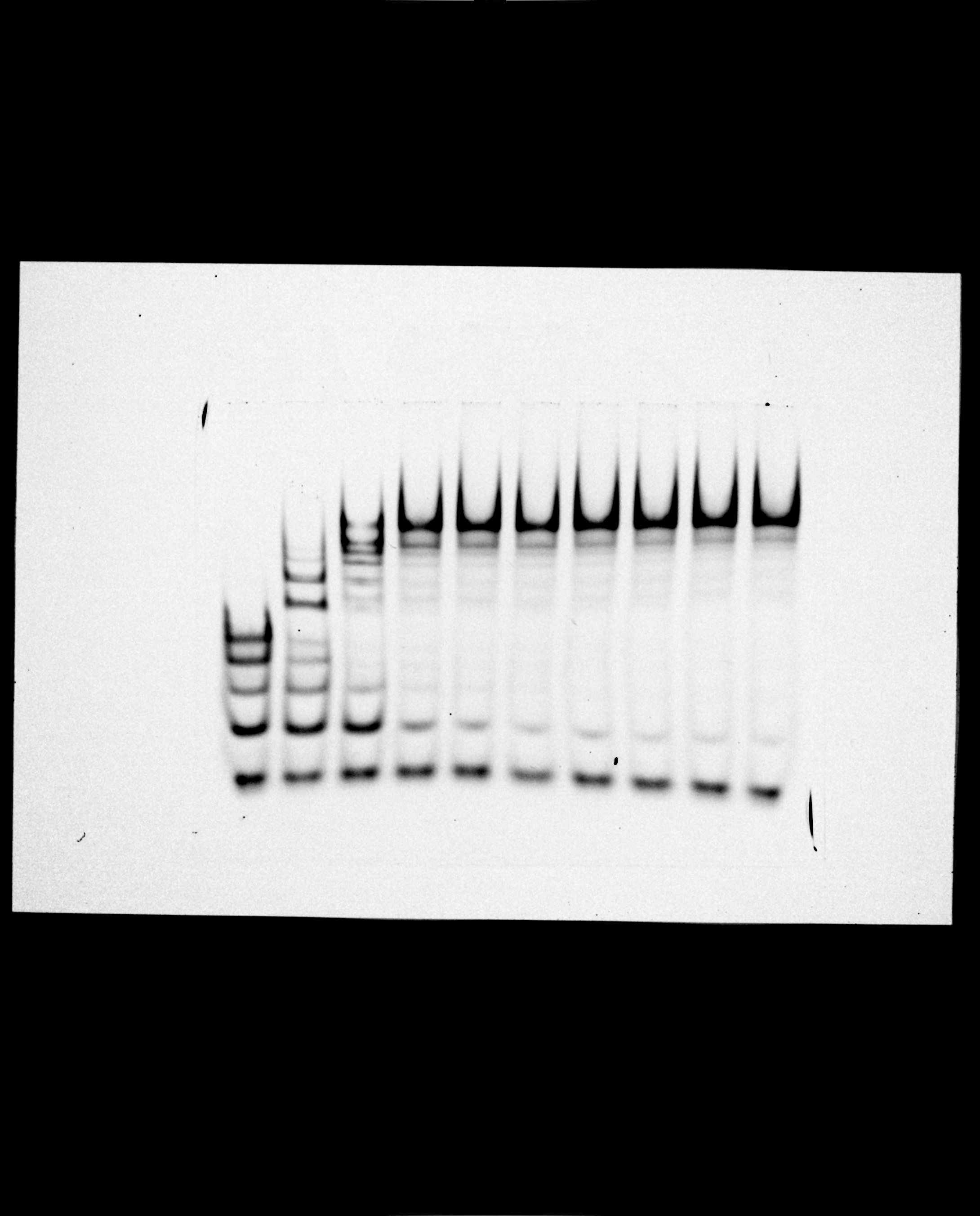

Supplement: Figure 4—source data 1. — Electrophoretic mobility shift assay (EMSA) images and data analyses. [file elife-83538-fig4-data1.zip › Figure 4 - Source data 1/b/211214 Cy5 ladder with yKER_PUB_600.tif]

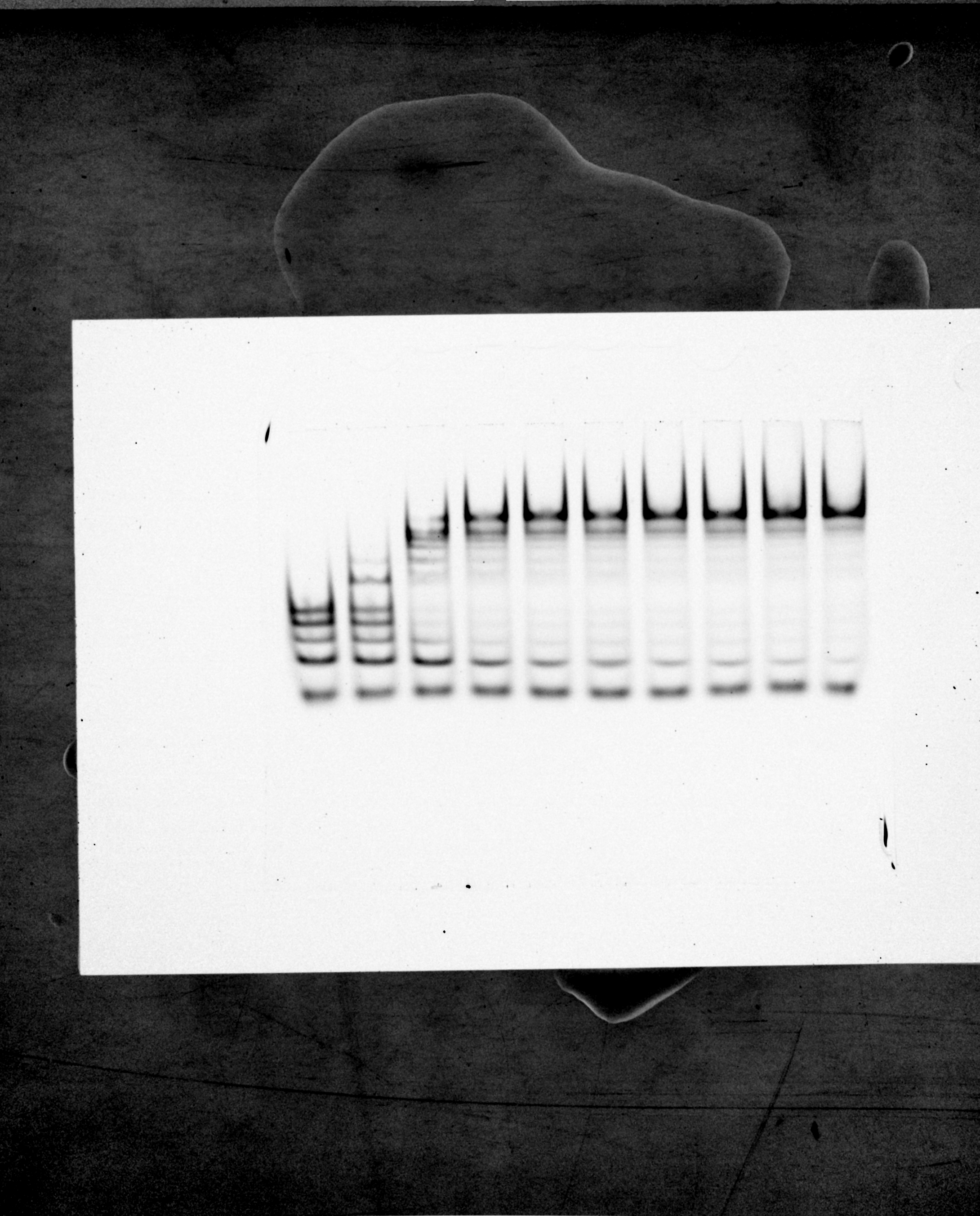

Supplement: Figure 4—source data 1. — Electrophoretic mobility shift assay (EMSA) images and data analyses. [file elife-83538-fig4-data1.zip › Figure 4 - Source data 1/b/220120 Cy5 ladder EMSA with yKER_n1_PUB_600.tif]

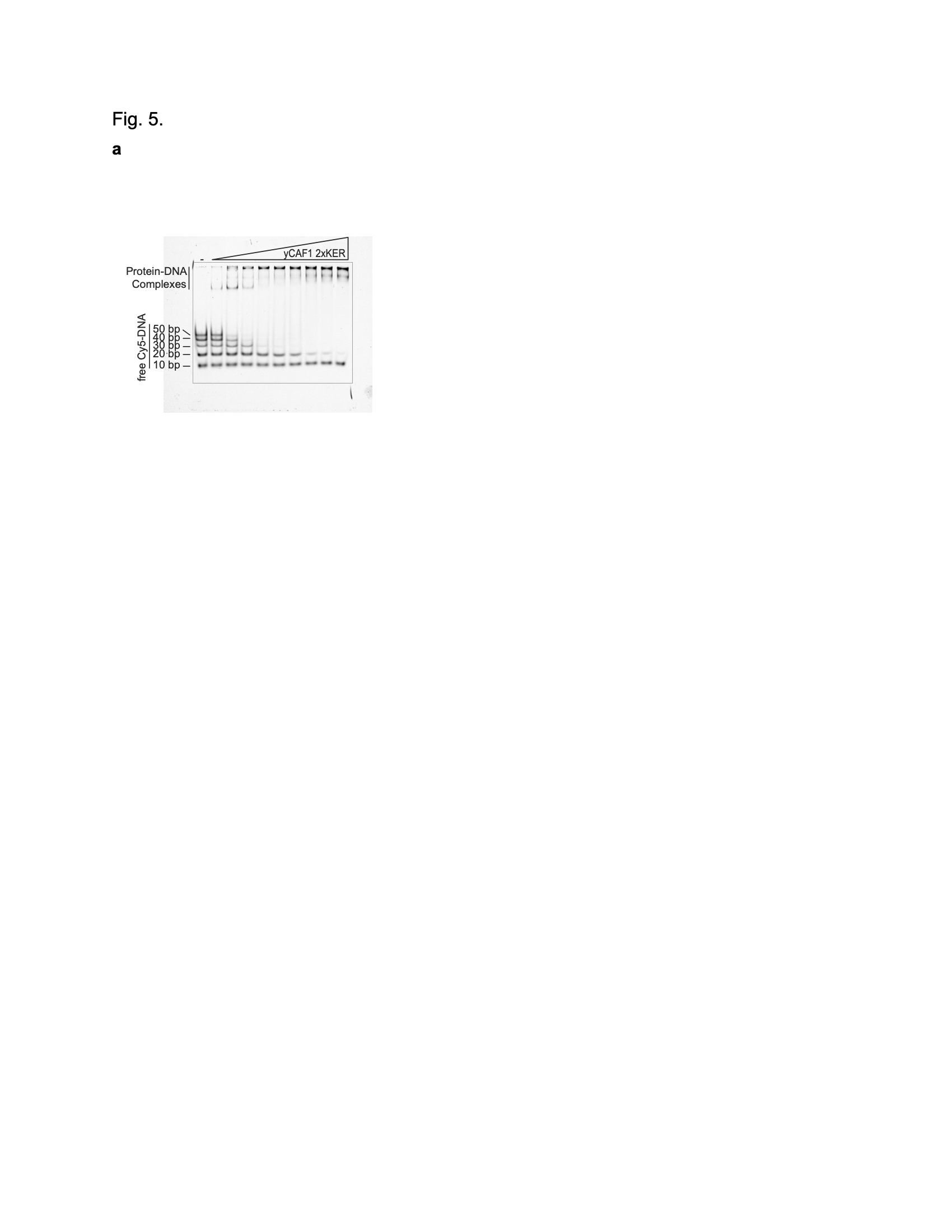

Supplement: Figure 5—source data 1. — Electrophoretic mobility shift assay (EMSA) images (panel a) and flow cytometry data (panel c). [file elife-83538-fig5-data1.zip › Figure 5 - Source data 1/Figure 5 - Source data 1_Gels Labeled.png]

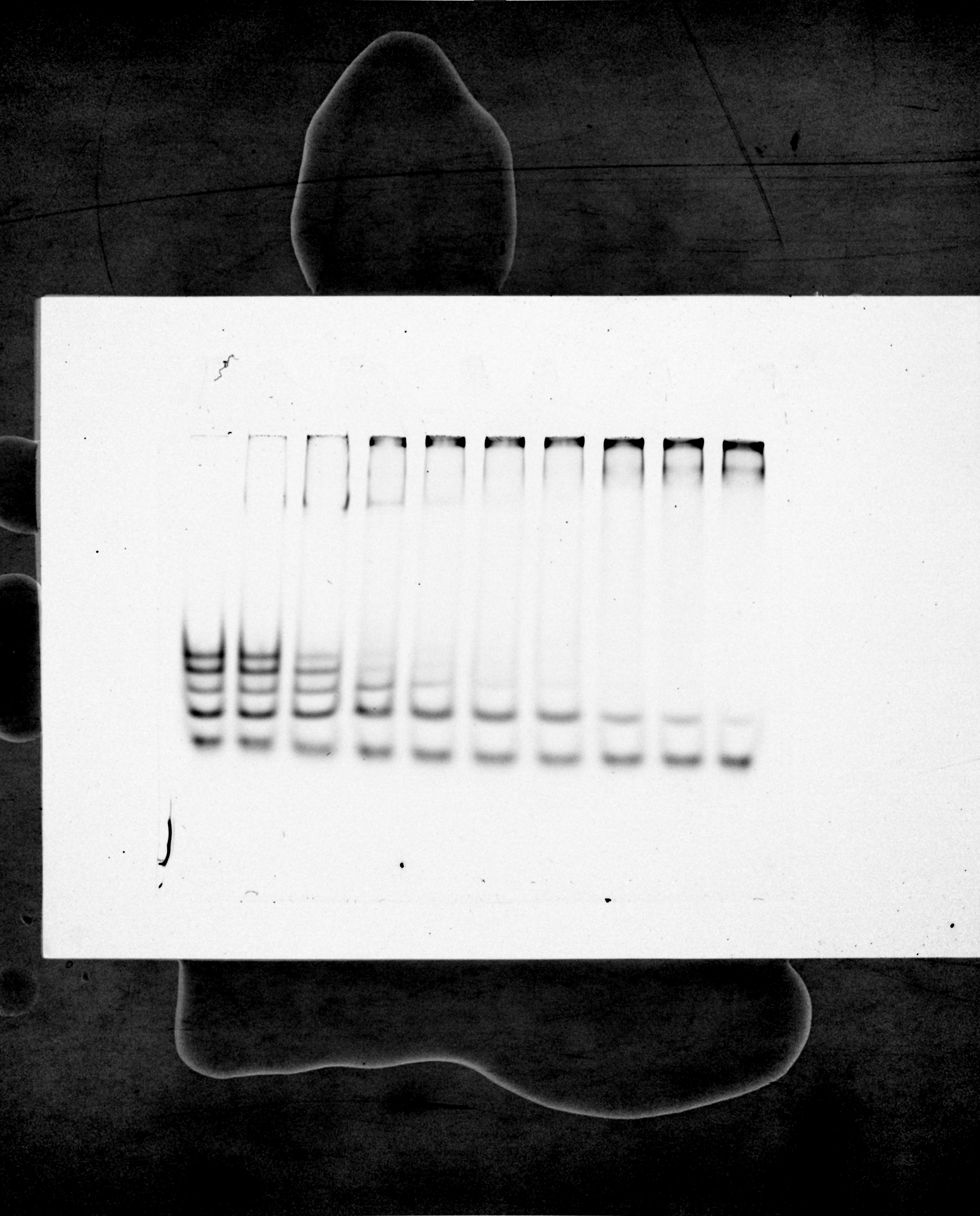

Supplement: Figure 5—source data 1. — Electrophoretic mobility shift assay (EMSA) images (panel a) and flow cytometry data (panel c). [file elife-83538-fig5-data1.zip › Figure 5 - Source data 1/a/220324 Cy5 ladder EMSA with yCAF1 2xKER_n3_PUB_600.tif]

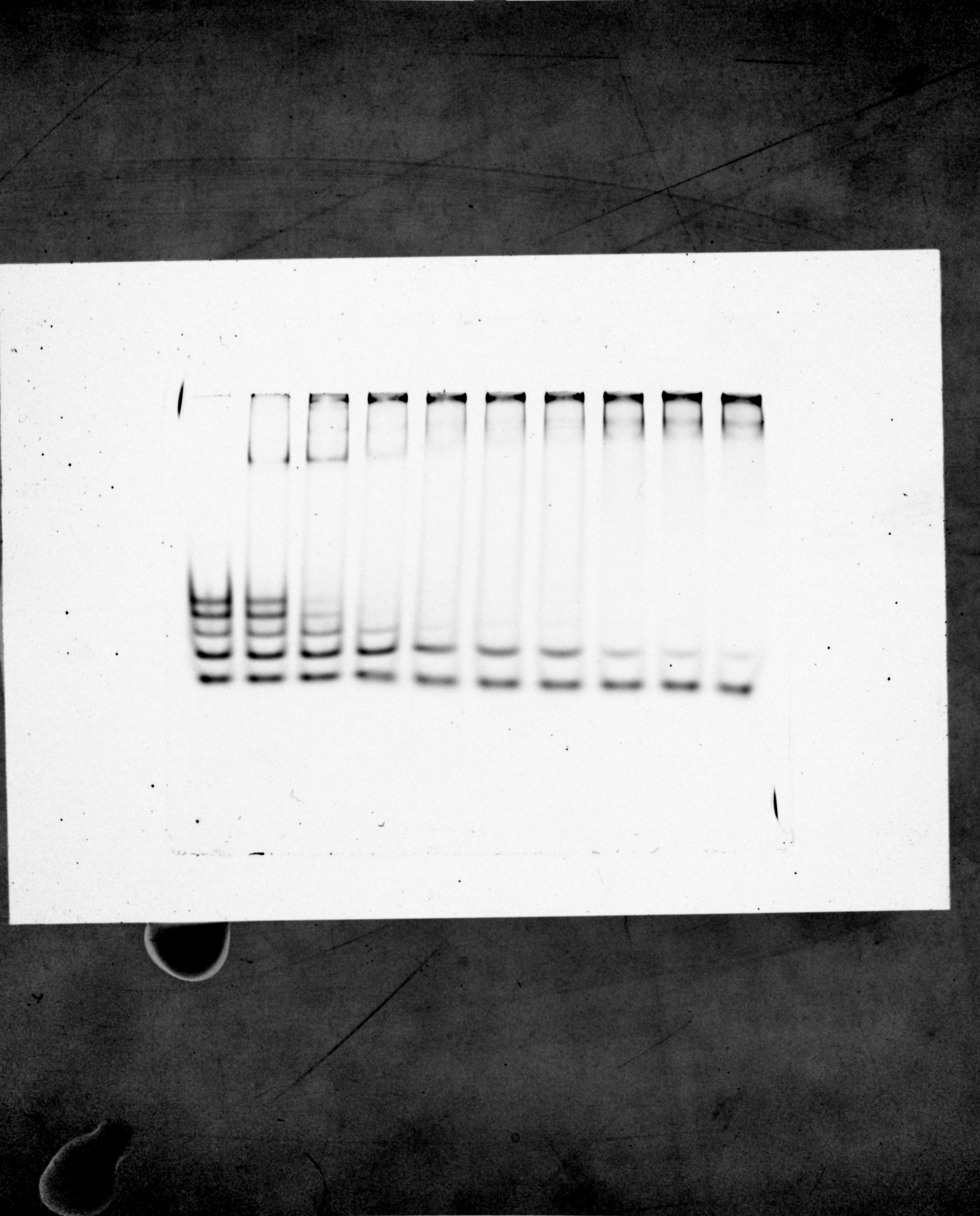

Supplement: Figure 5—source data 1. — Electrophoretic mobility shift assay (EMSA) images (panel a) and flow cytometry data (panel c). [file elife-83538-fig5-data1.zip › Figure 5 - Source data 1/a/220324 Cy5 ladder EMSA with yCAF1 2xKER_n1_PUB_600.tif]

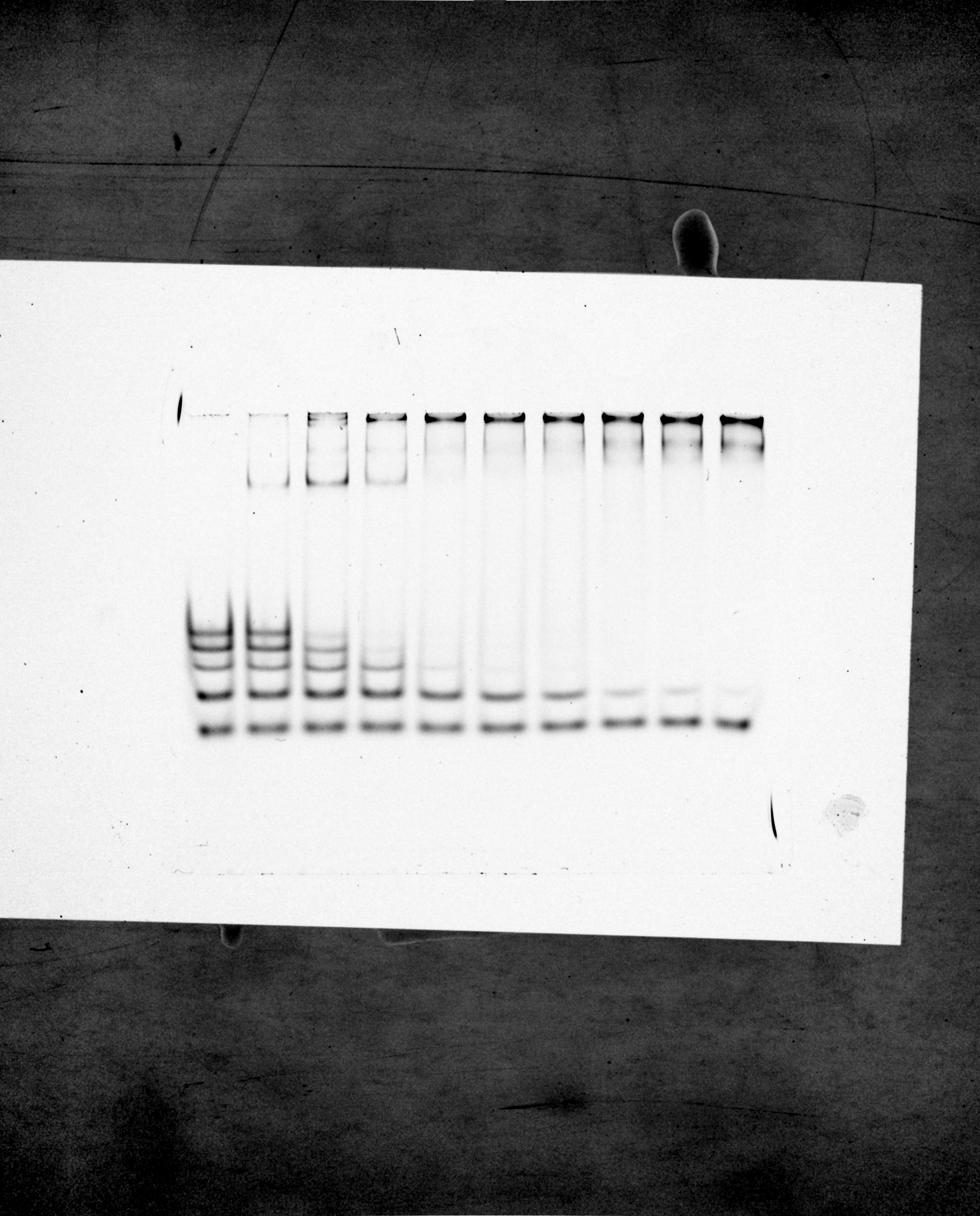

Supplement: Figure 5—source data 1. — Electrophoretic mobility shift assay (EMSA) images (panel a) and flow cytometry data (panel c). [file elife-83538-fig5-data1.zip › Figure 5 - Source data 1/a/220324 Cy5 ladder EMSA with yCAF1 2xKER_n2_PUB_600.tif]

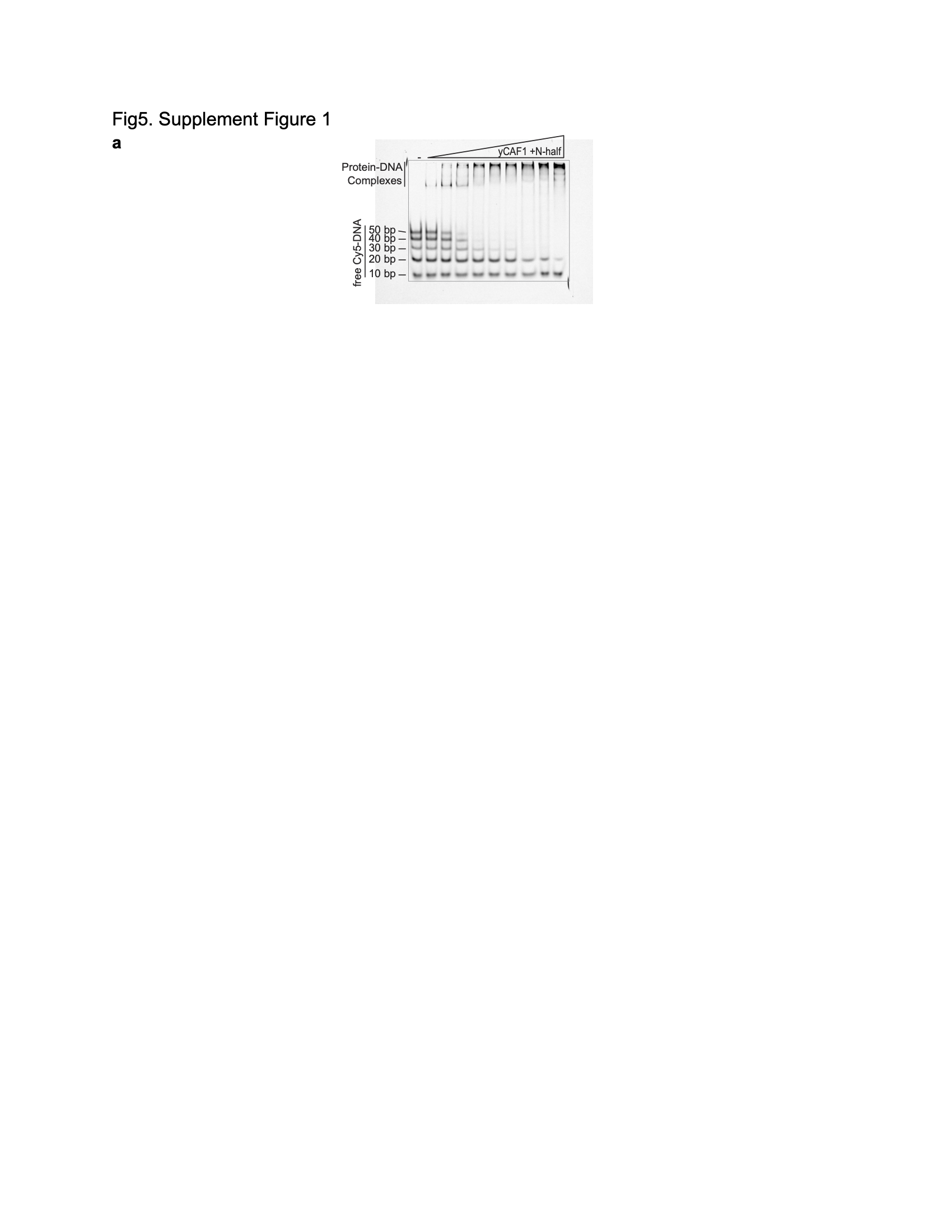

Supplement: Figure 5—figure supplement 1—source data 1. — Electrophoretic mobility shift assay (EMSA) images and data analyses (panel a). [file elife-83538-fig5-figsupp1-data1.zip › Figure 5 - figure supplement 1 - Source data 1/Figure 5 - figure supplement 1- Source data 1_Gels Labeled.png]

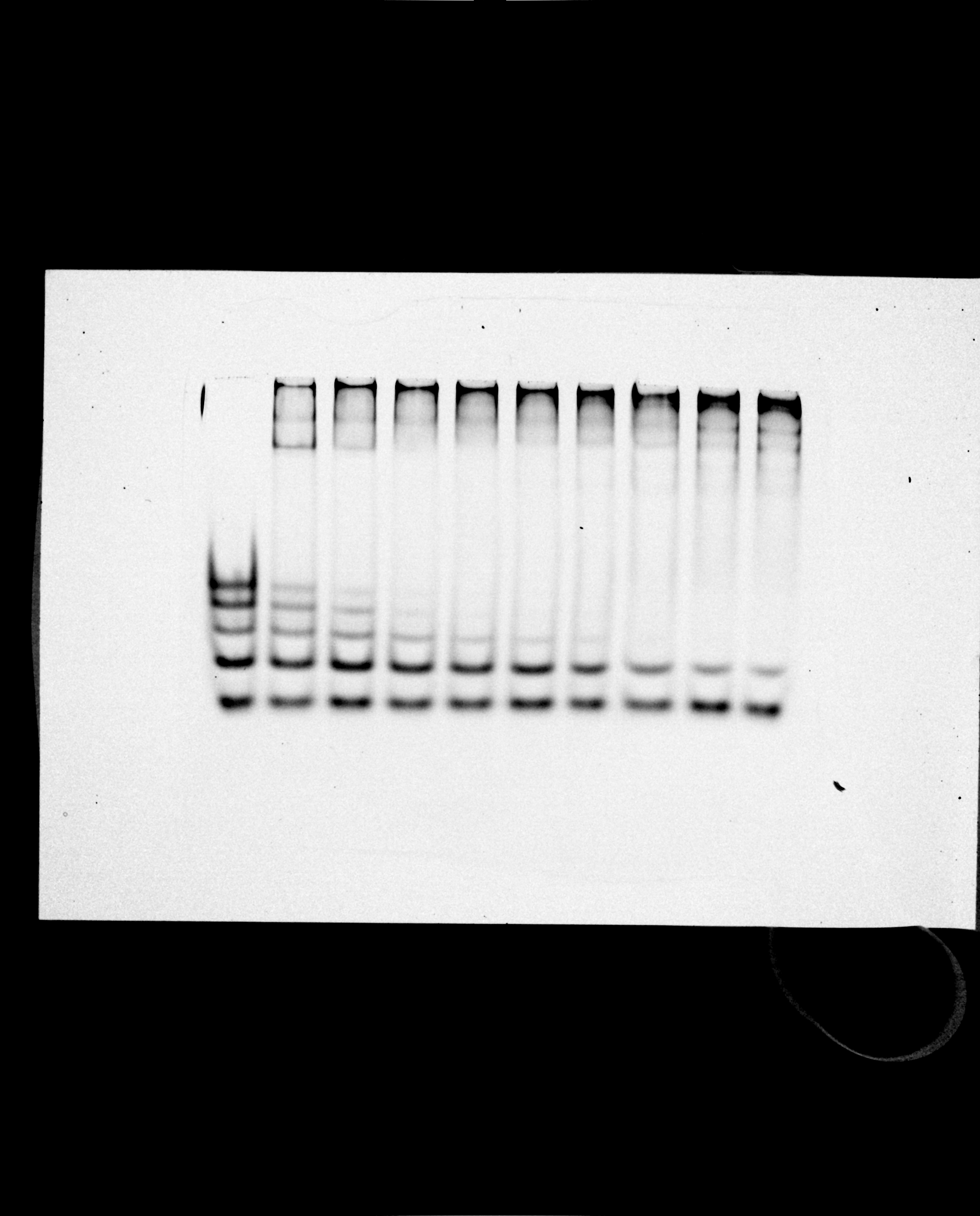

Supplement: Figure 5—figure supplement 1—source data 1. — Electrophoretic mobility shift assay (EMSA) images and data analyses (panel a). [file elife-83538-fig5-figsupp1-data1.zip › Figure 5 - figure supplement 1 - Source data 1/a/211222 Cy5 ladder EMSA with yCAF1 plusNHalf_PUB_600.tif]

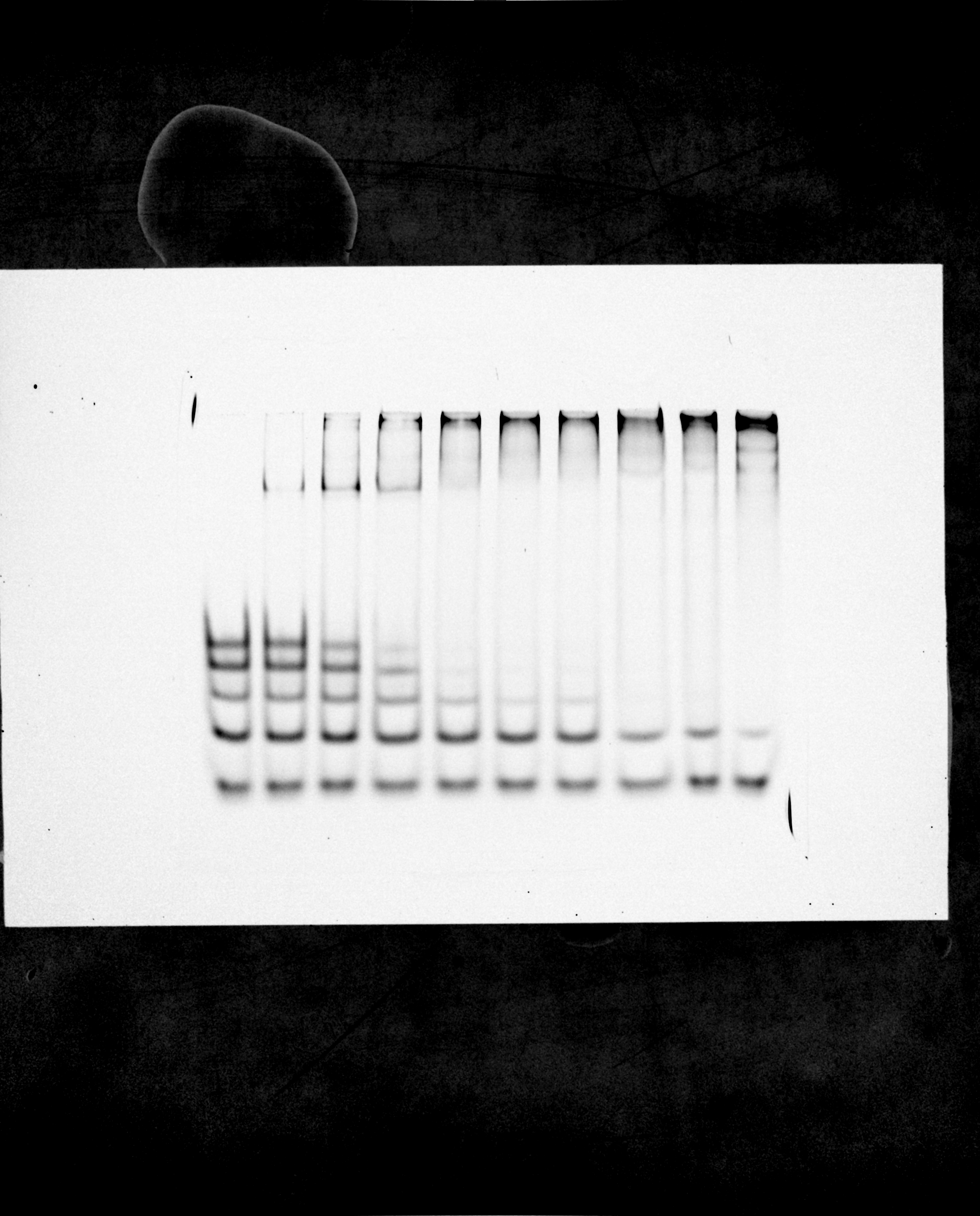

Supplement: Figure 5—figure supplement 1—source data 1. — Electrophoretic mobility shift assay (EMSA) images and data analyses (panel a). [file elife-83538-fig5-figsupp1-data1.zip › Figure 5 - figure supplement 1 - Source data 1/a/220121 Cy5 ladder EMSA with yCAF1 plusNHalf_n1_PUB_600.tif]

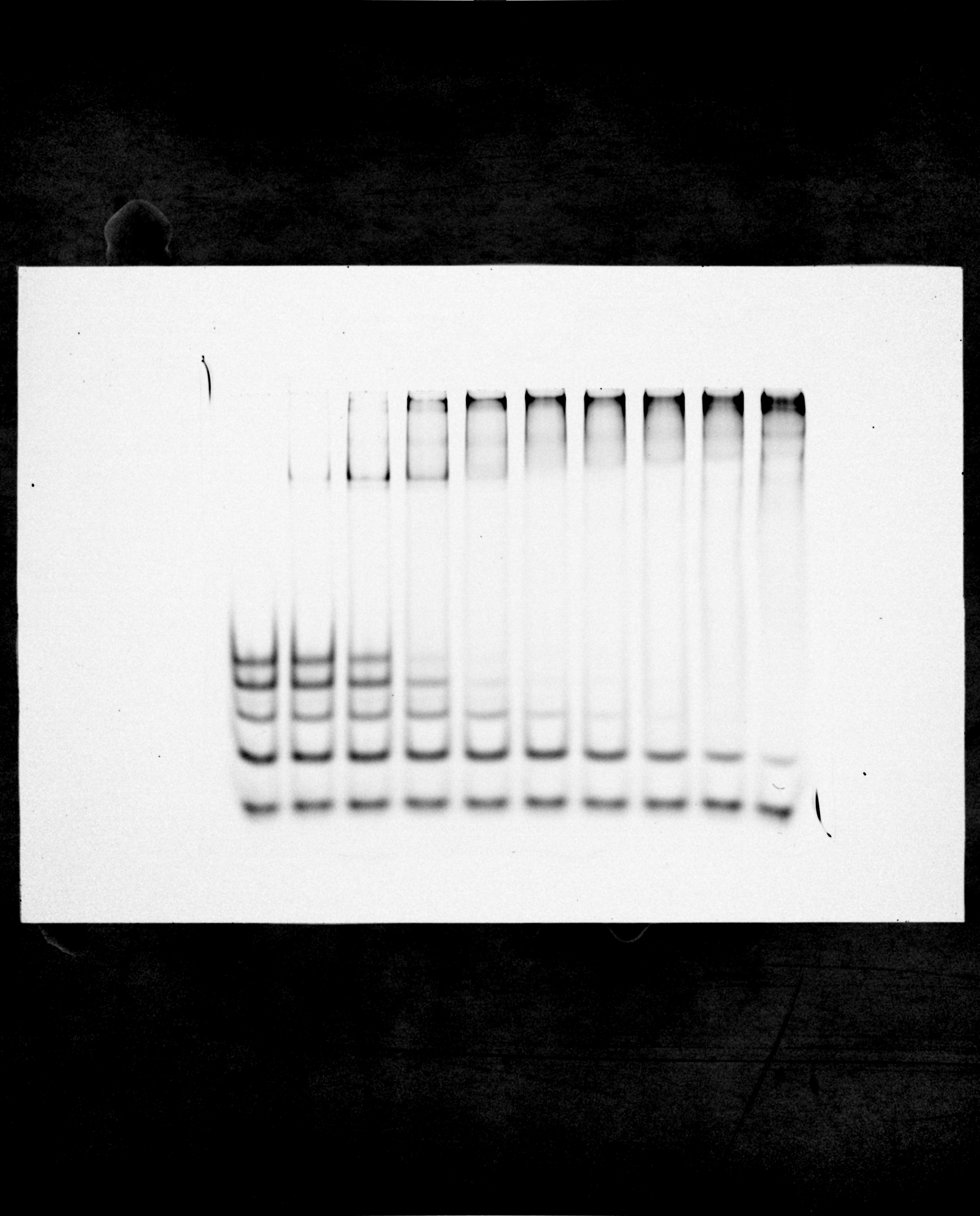

Supplement: Figure 5—figure supplement 1—source data 1. — Electrophoretic mobility shift assay (EMSA) images and data analyses (panel a). [file elife-83538-fig5-figsupp1-data1.zip › Figure 5 - figure supplement 1 - Source data 1/a/220121 Cy5 ladder EMSA with yCAF1 plusNHalf_n2_PUB_600.tif]

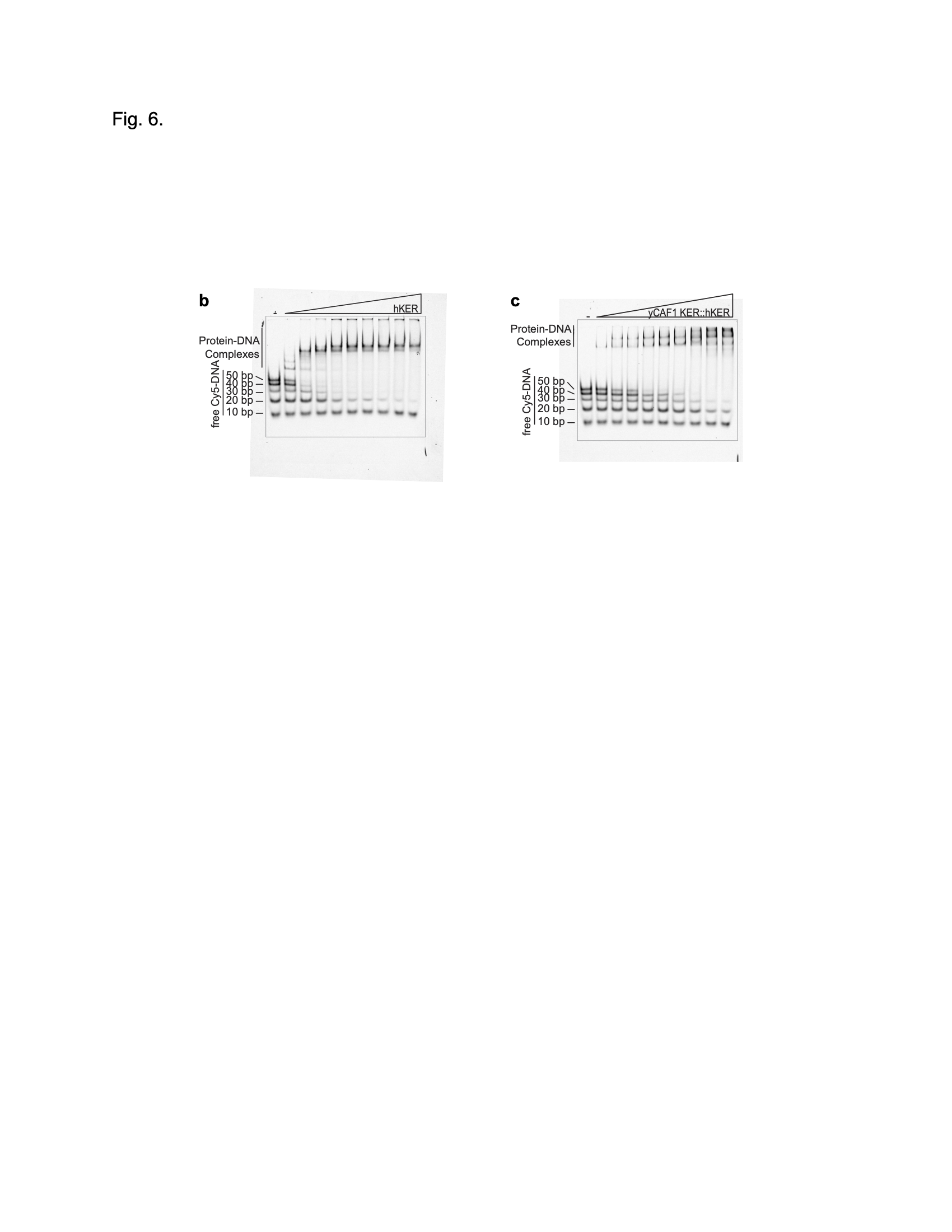

Supplement: Figure 6—source data 1. — Electrophoretic mobility shift assay (EMSA) images and data analyses (panels b and c) and flow cytometry data and analyses (panel e). [file elife-83538-fig6-data1.zip › Figure 6 - Source data 1/Figure 6 - Source data 1_Gels Labeled.png]

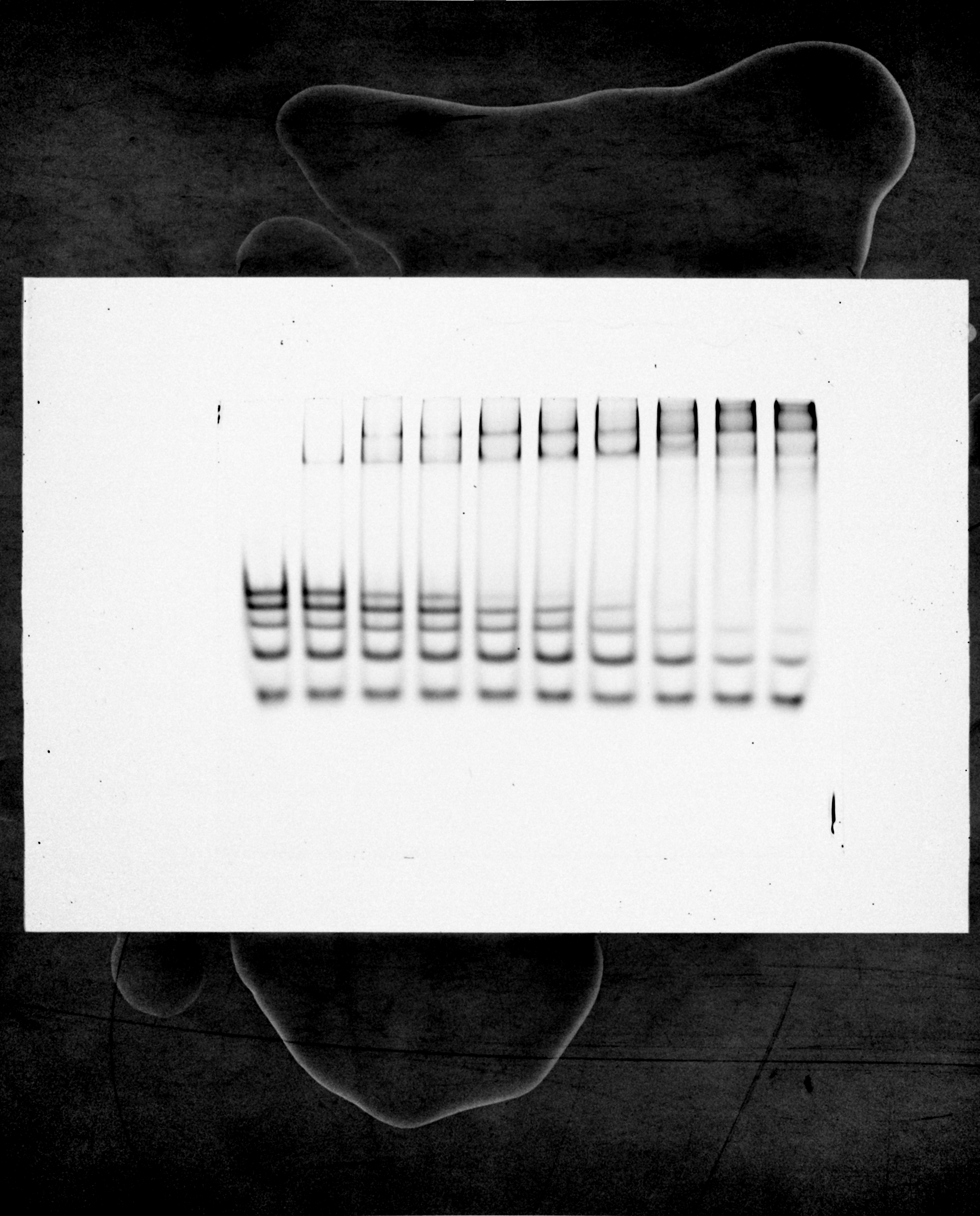

Supplement: Figure 6—source data 1. — Electrophoretic mobility shift assay (EMSA) images and data analyses (panels b and c) and flow cytometry data and analyses (panel e). [file elife-83538-fig6-data1.zip › Figure 6 - Source data 1/c/220121 Cy5 ladder EMSA with yCAF1 yKERtohKER_n1_PUB_600.tif]

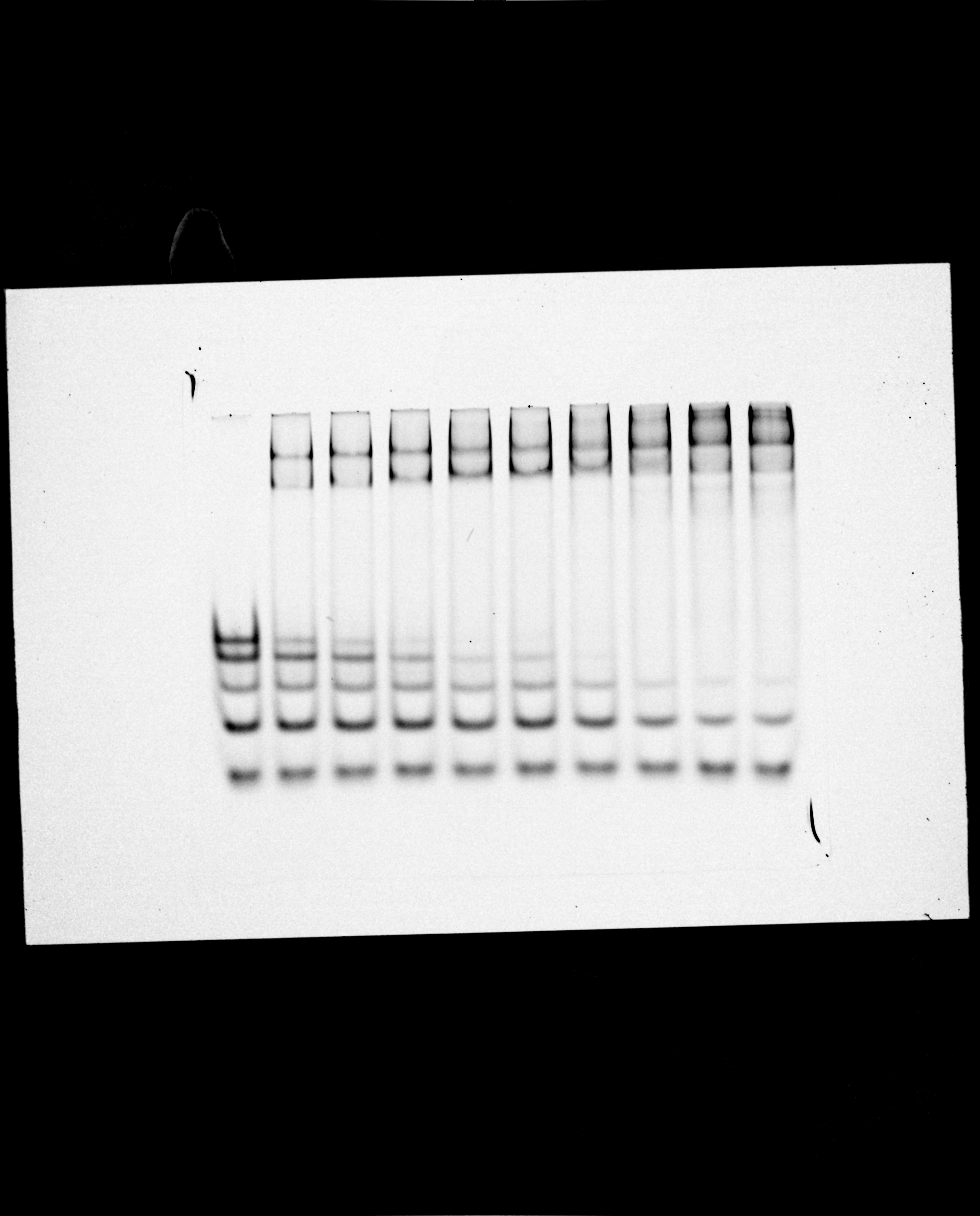

Supplement: Figure 6—source data 1. — Electrophoretic mobility shift assay (EMSA) images and data analyses (panels b and c) and flow cytometry data and analyses (panel e). [file elife-83538-fig6-data1.zip › Figure 6 - Source data 1/c/211227 Cy5 ladder EMSA with yCAF1 yKERtohKER_PUB_600.tif]

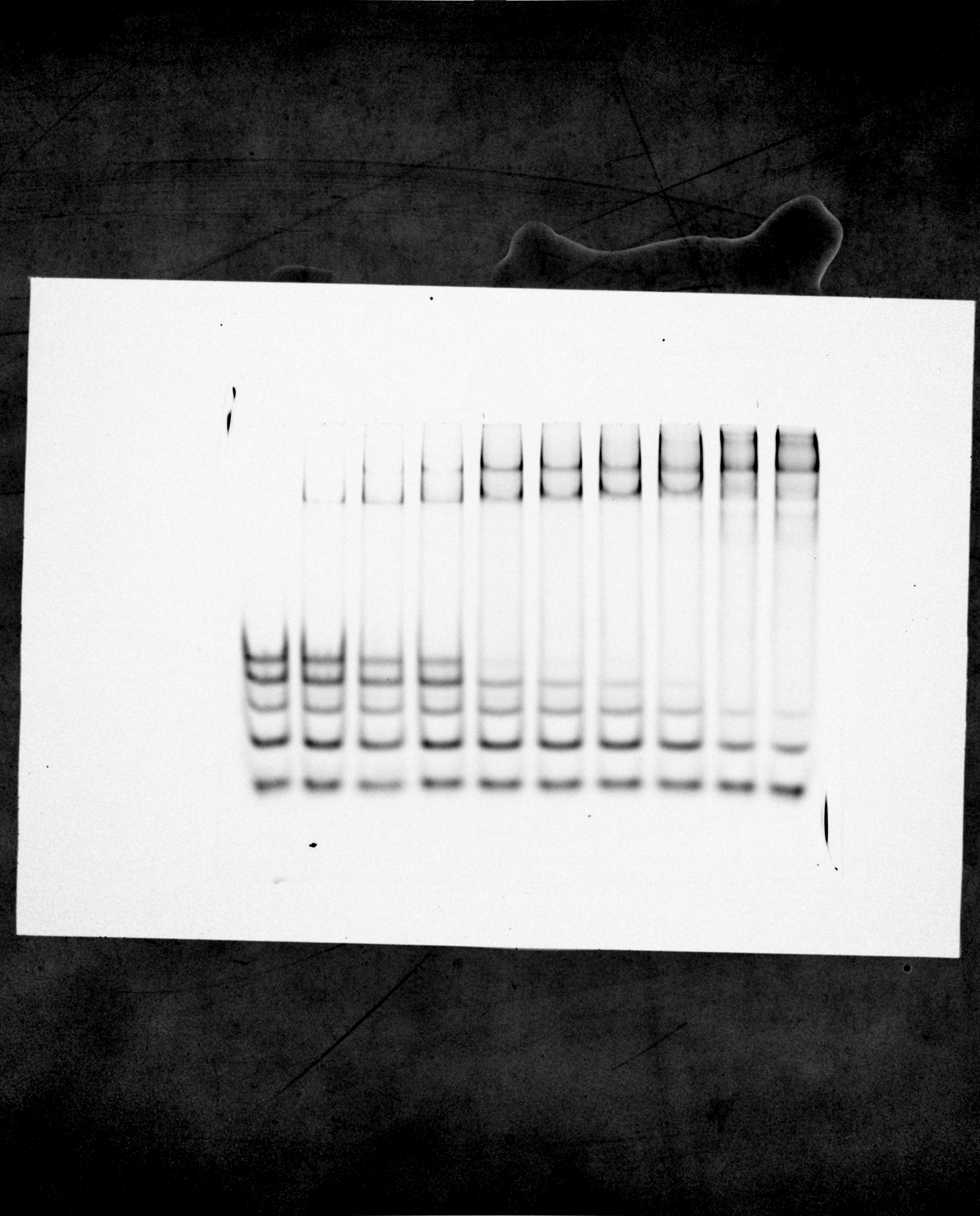

Supplement: Figure 6—source data 1. — Electrophoretic mobility shift assay (EMSA) images and data analyses (panels b and c) and flow cytometry data and analyses (panel e). [file elife-83538-fig6-data1.zip › Figure 6 - Source data 1/c/220121 Cy5 ladder EMSA with yCAF1 yKERtohKER_n2_PUB_600.tif]

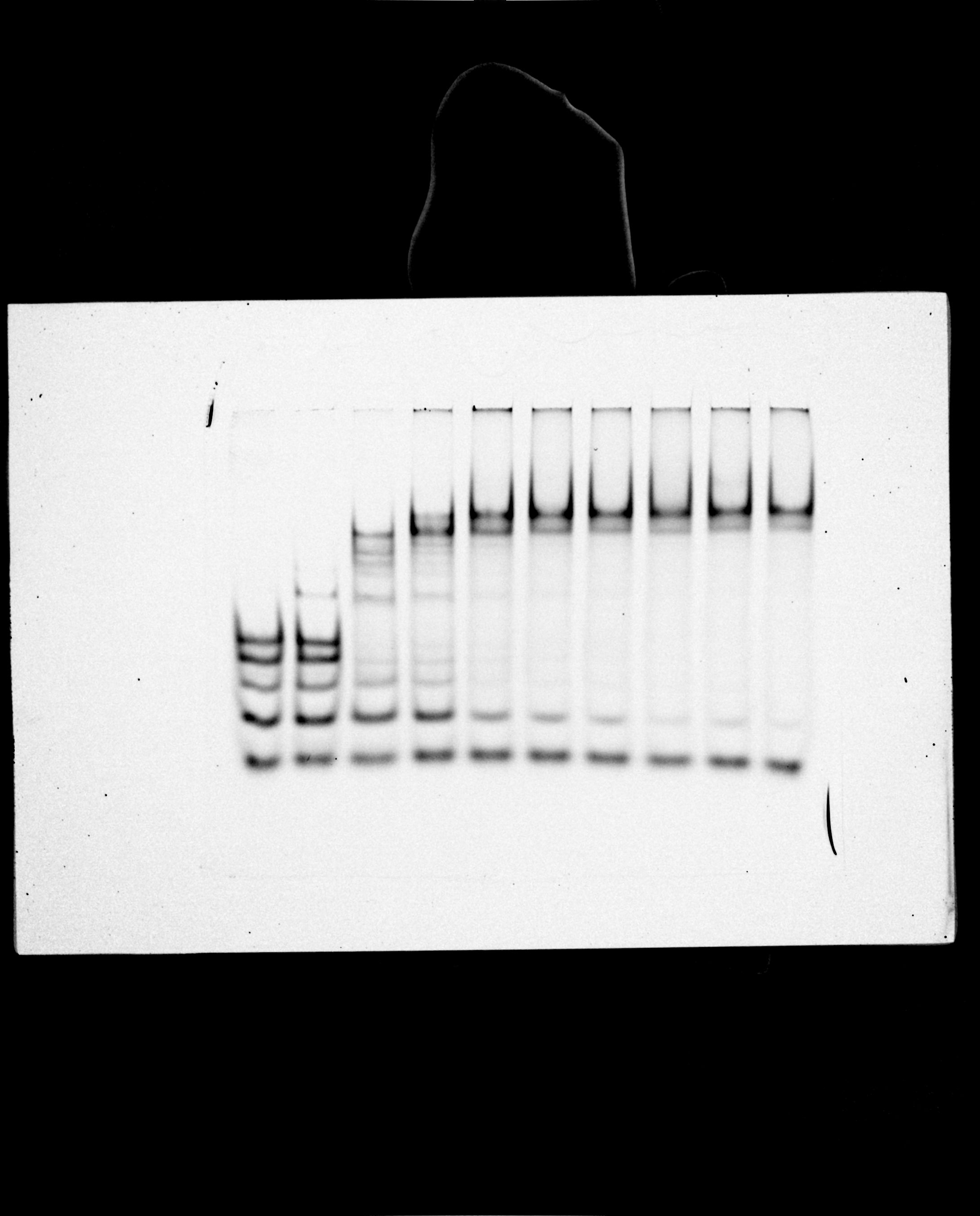

Supplement: Figure 6—source data 1. — Electrophoretic mobility shift assay (EMSA) images and data analyses (panels b and c) and flow cytometry data and analyses (panel e). [file elife-83538-fig6-data1.zip › Figure 6 - Source data 1/b/220120 Cy5 ladder EMSA with hKER_n2_PUB_600.tif]
