# Supplementary figures and images for: A novel single alpha-helix DNA-binding domain in CAF-1 promotes gene silencing and DNA damage survival through tetrasome-length DNA selectivity and spacer function (part 2 of 2)
Source: eLife. 2023 Jul 11;12:e83538. doi: 10.7554/eLife.83538 (PMC10335832; doi:10.7554/eLife.83538)

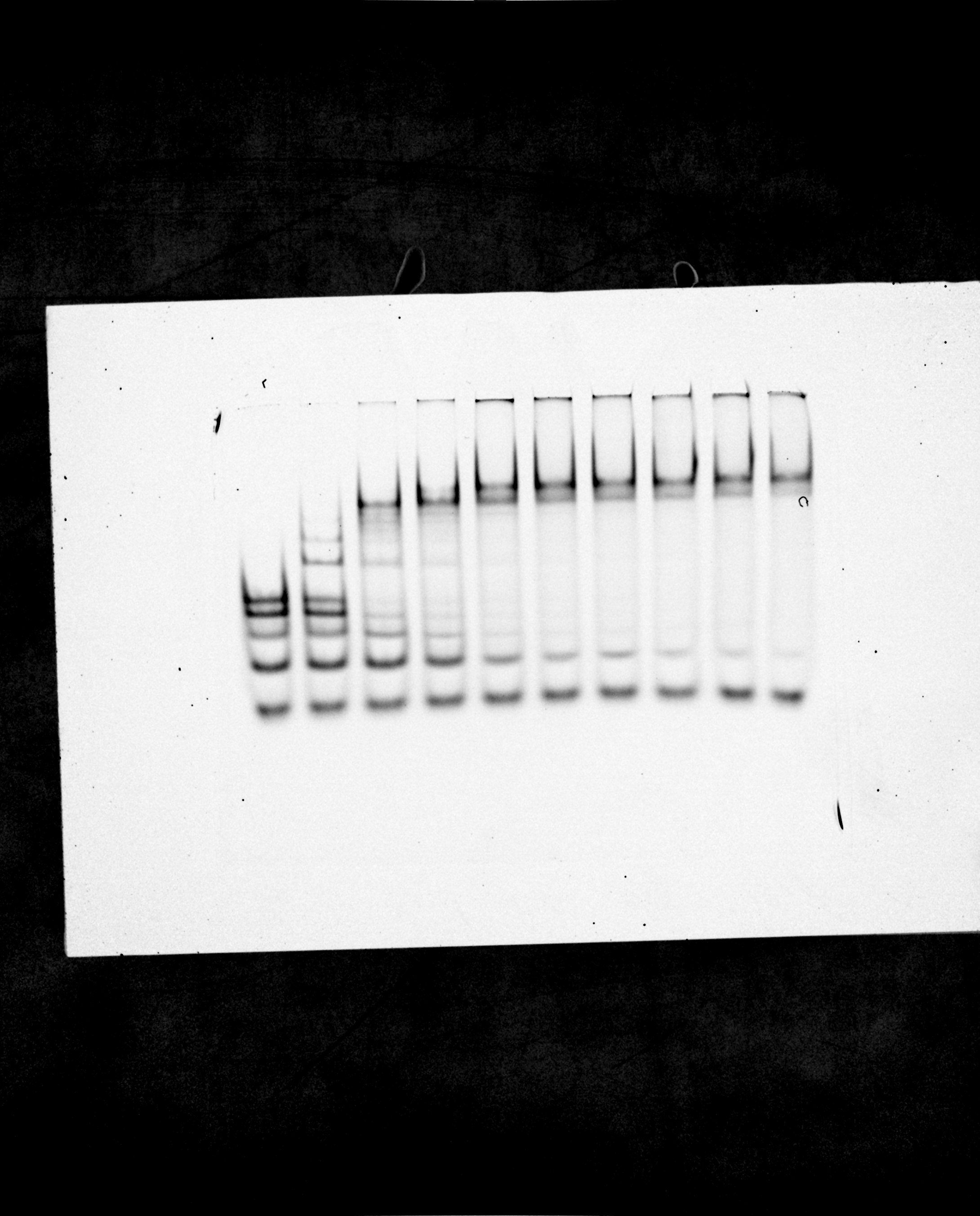

Supplement: Figure 6—source data 1. — Electrophoretic mobility shift assay (EMSA) images and data analyses (panels b and c) and flow cytometry data and analyses (panel e). [file elife-83538-fig6-data1.zip › Figure 6 - Source data 1/b/220120 Cy5 ladder EMSA with hKER_n1_PUB_600.tif]

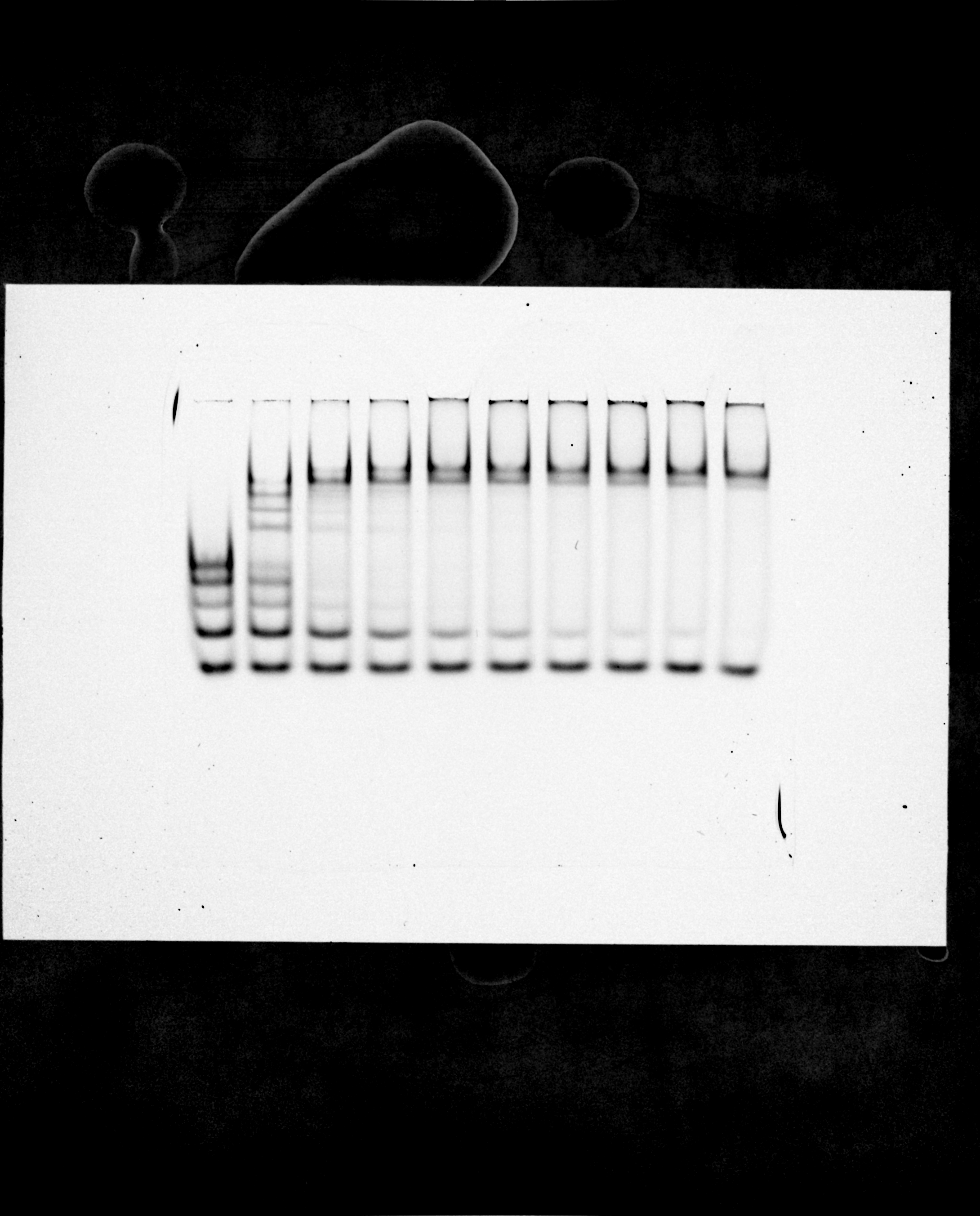

Supplement: Figure 6—source data 1. — Electrophoretic mobility shift assay (EMSA) images and data analyses (panels b and c) and flow cytometry data and analyses (panel e). [file elife-83538-fig6-data1.zip › Figure 6 - Source data 1/b/220103 Cy5 ladder EMSA with hKER_PUB_600.tif]
