# Supplementary material for: A novel single alpha-helix DNA-binding domain in CAF-1 promotes gene silencing and DNA damage survival through tetrasome-length DNA selectivity and spacer function
Source: eLife. 2023 Jul 11;12:e83538. doi: 10.7554/eLife.83538 (PMC10335832; doi:10.7554/eLife.83538)
Supplement: Supplementary file 1. [file elife-83538-supp1.docx]

**Supplementary Table 1.**  List of plasmids.

| **Plasmid** | **Description** | **Reference** |
| --- | --- | --- |
| Cac1, pDONR/Zeo | Cac1 with C-terminal StrepII tag | ([Liu et al., 2012](#_ENREF_32)) |
| Cac1 ∆KER, pDONR/Zeo | Cac1 without residues 136–225 and with C-terminal StrepII tag | This study |
| Cac1 ∆WHD, pDONR/Zeo | Cac1 without residues 520–606 and with C-terminal StrepII tag | This study |
| Cac1 ∆middle-A, pDONR/Zeo | Cac1 without residues 155–204 and with C-terminal StrepII tag | This study |
| Cac1 ED::GSL, pDONR/Zeo | Cac1 residues 397–431 were replaced with glycine/serine/leucine linker. Contains C-terminal StrepII tag | This study |
| Cac1 +N-half, pDONR/Zeo | Cac1 residues 136–172 were introduced after the endogenous 225 residue of Cac1. Contains C-terminal StrepII tag | This study |
| Cac1 2xKER, pDONR/Zeo | Cac1 residues 136–225 were introduced after the endogenous 225 residue in Cac1. Contains C-terminal StrepII tag | This study |
| Cac1 KER::myosin7aSAH, pDONR/Zeo | Cac1 residues 136–225 were replaced with mouse Myosin 7a residues 866–932. Contains a C-terminal StrepII tag | This study |
| Cac1 KER::hKER, pDONR/Zeo | Cac1 residues 136–225 were replaced with CHAF1A residues 331–441. Contains a C-terminal StrepII tag | This study |
| Cac1, pDEST8 | Cac1 with C-terminal StrepII tag | This study |
| Cac1 ∆KER, pDEST8 | Cac1 without residues 136–225 and with C-terminal StrepII tag | This study |
| Cac1 ∆WHD, pDEST8 | Cac1 without residues 520–606 and with C-terminal StrepII tag | This study |
| Cac1 ∆middle-A, pDEST8 | Cac1 without residues 155–204 and with C-terminal StrepII tag | This study |
| Cac1 ED::GSL, pDEST8 | Cac1 residues 397–431 were replaced with glycine/serine/leucine linker. Contains C-terminal StrepII tag | This study |
| Cac1 +N-half, pDEST8 | Cac1 residues 136–172 were introduced after the endogenous 225 residue of Cac1. Contains C-terminal StrepII tag | This study |
| Cac1 2xKER, pDONR/Zeo | Cac1 residues 136–225 were introduced after the endogenous 225 residue in Cac1. Contains C-terminal StrepII tag | This study |
| Cac1 KER::myosin7aSAH, pDEST8 | Cac1 residues 136–225 were replaced with mouse Myosin 7a residues 866–932. Contains a C-terminal StrepII tag | This study |
| Cac1 KER::hKER, pDEST8 | Cac1 residues 136–225 were replaced with hCHAF1A residues 331–441. Contains a C-terminal StrepII tag | This study |
| Cac2, pDONR/Zeo | Cac2 with C-terminal StrepII tag | This study |
| Cac2, pDEST8 | Cac2 with C-terminal StrepII tag | This study |
| Cac3, pDONR/Zeo | Cac3 with C-terminal 3xFLAG tag | This study |
| Cac3, pDEST8 | Cac3 with C-terminal 3xFLAG tag | This study |
| Cac1 KER, pDONR/Zeo | Cac1 residues 136–225 | This study |
| Cac1 KER, pDEST566 | Cac1 residues 136–225 | This study |
| Cac1 KER, pDONR/Zeo | Cac1 residues 136–225 with C-terminal tyrosine | This study |
| Cac1 KER, pDEST566 | Cac1 residues 136–225 with C-terminal tyrosine | This study |
| Cac1 N-half, pDONR/Zeo | Cac1 residues 136–172 with C-terminal tyrosine | This study |
| Cac1 N-half, pDEST566 | Cac1 residues 136–172 with C-terminal tyrosine | This study |
| Cac1 C-half, pDONR/Zeo | Cac1 residues 179–225 with C-terminal tyrosine | This study |
| Cac1 C-half, pDEST566 | Cac1 residues 179–225 with C-terminal tyrosine | This study |
| Cac1 middle-A, pDONR/Zeo | Cac1 residues 155–204 with C-terminal tyrosine | This study |
| Cac1 middle-A, pDEST566 | Cac1 residues 155–204 with C-terminal tyrosine | This study |
| Cac1 middle-B, pDONR/Zeo | Cac1 residues 173–204 with C-terminal tyrosine | This study |
| Cac1 middle-B, pDEST566 | Cac1 residues 173–204 with C-terminal tyrosine | This study |
| CHAF1A KER (hKER), pDONR/Zeo | CHAF1A residues 331–441 with C-terminal tyrosine | This study |
| CHAF1A KER (hKER), pDEST566 | CHAF1A residues 331–441 with C-terminal tyrosine | This study |
| Myosin 7a SAH, pDONR/Zeo | Myosin 7a residues 866–932 with C-terminal tyrosine | This study |
| Myosin 7a SAH, pDEST566 | Myosin 7a residues 866–932 with C-terminal tyrosine | This study |
| Cac1 WHD, pGEX-6P-1 | Cac1 residues 457–606 | ([Liu et al., 2016](#_ENREF_33)) |
| CHAF1A, pGEX-6P-1 | Human CHAF1A | This study |
