# Supplementary material for: A novel single alpha-helix DNA-binding domain in CAF-1 promotes gene silencing and DNA damage survival through tetrasome-length DNA selectivity and spacer function
Source: eLife. 2023 Jul 11;12:e83538. doi: 10.7554/eLife.83538 (PMC10335832; doi:10.7554/eLife.83538)
Supplement: Supplementary file 2. [file elife-83538-supp2.docx]

**Supplementary Table 2.**  List of synthetic DNA oligonucleotides and primers.

| **Name** | **Sequence (5'-3')** |
| --- | --- |
| Cy5 601 10 nts. Sense | Cy5-CGCGCTGTCC |
| Cy5 601 20 nts. Sense | Cy5-ACGTACGCGCTGTCCCCCGC |
| Cy5 601 30 nts. Sense | Cy5‑AACGCACGTACGCGCTGTCCCCCGCGTTTT |
| Cy5 601 40 nts. Sense | Cy5‑GCTTAAACGCACGTACGCGCTGTCCCCCGCGTTTTAACCG |
| 601 40 nts. Sense | GCTTAAACGCACGTACGCGCTGTCCCCCGCGTTTTAACCG |
| Cy5 601 50 nts. Sense | Cy5‑GCACCGCTTAAACGCACGTACGCGCTGTCCCCCGCGTTTTAACCGCCAAG |
| Cy5 601 80 nts. Sense | Cy5‑GTCGTAGACAGCTCTAGCACCGCTTAAACGCACGTACGCGCTGTCCCCCGCGTTTTAACCGCCAAGGGGATTACTCCCTA |
| 601 10 nts. Antisense | GGACAGCGCG |
| 601 20 nts. Antisense | GCGGGGGACAGCGCGTACGT |
| 601 30 nts. Antisense | AAAACGCGGGGGACAGCGCGTACGTGCGTT |
| 601 40 nts. Antisense | CGGTTAAAACGCGGGGGACAGCGCGTACGTGCGTTTAAGC |
| 601 50 nts. Antisense | CTTGGCGGTTAAAACGCGGGGGACAGCGCGTACGTGCGTTTAAGCGGTGC |
| 601 80 nts. Antisense | TAGGGAGTAATCCCCTTGGCGGTTAAAACGCGGGGGACAGCGCGTACGTGCGTTTAAGCGGTGCTAGAGCTGTCTACGAC |
| Cac1 ∆KER  Forward | GCACAATCCCGTATTGGTAACTTCTTTAAAAAACTAAGCG |
| Cac1 ∆KER  Reverse | TTACCAATACGGGATTGTGCCGATGAGGAAAGTTCTCTCTTAGAGCATGG |
| Cac1 ∆middle-A Forward | AGAAAAGAGGAGGAAAGATTGAAAAAGGAGGAGGAAATACG |
| Cac1 ∆middle-A Reverse | AATCTTTCCTCCTCTTTTCTTTTCTCTTCTGCACGTTGCTGCTTTTTTAGTTCC |
| Cac1 ∆WHD Forward | ATGCCAACCCCGTCTTTGTCAGGATGGAGCCACCCGCAGTTCGAAAAGTAG |
| Cac1 ∆WHD Reverse | GACAAAGACGGGGTTGGCATTTTCTTTTCGGGACTTTGAGATTGGCTAGCGG |
| Cac1 KER::Myosin7a SAH Forward | GCACAATCCCGTATTGGTAA |
| Cac1 KER::Myosin7a SAH Reverse | CGATGAGGAAAGTTCTCTCT |
| Cac1 KER::Myosin7a SAH dsDNA | AGAGAGAACTTTCCTCATCGCGCCTGGAAGCGGAACGCATGCGCCTGGCGGAAGAAGAAAAACTGCGCAAAGAAATGAGCGCGAAAAAAGCGAAAGAAGAAGCGGAACGCAAACATCAGGAACGCCTGGCGCAGCTGGCGCGCGAAGATGCGGAACGCGAACTGAAAGAAAAAGAAGAAGCGCGCCGCAAAAAAGAACTGCTGGAACAGATGGAAAAAGCGGCACAATCCCGTATTGGTAA |
| Cac1 ED::GSL  Forward | AACAGTGATTTGGATGGCCTACCCTGC |
| Cac1 ED::GSL  Reverse | TTCTTCTTCTTCATTAACCCATTCAACG |
| Cac1 ED::GSL  dsDNA | GGGTTAATGAAGAAGAAGAAGGCTCACTGGGGTCCCTTGGAAGCTTAGGGTCTCTTGGGTCCCTGGGCTCTTTAGGAAGCCTTGGTTCACTTGGTTCATTAGGATCCCTAGGATCTTTGGGATCCAACAGTGATTTGGATGGCCT |
| Cac1 2xKER Forward | GCACAATCCCGTATTGGTAA |
| Cac1 2xKER Reverse | TCTTTCCTTGGCTTCTTCTTTCAAACG |
| Cac1 2xKER dsDNA -Fwd | AAGAAGAAGCCAAGGAAAGAAAAAAGGAAGAAGCTAAAAGAGAAAAGG |
| Cac1 2xKER dsDNA -Rev | TTACCAATACGGGATTGTGCTCTTTCCTTGGCTTCTTCTTTCAAACGTATTTCCTCC |
| Cac1 +N-half Forward | GCACAATCCCGTATTGGTAA |
| Cac1 +N-half Reverse | TCTTTCCTTGGCTTCTTCTTTCAAACG |
| Cac1 +N-half dsDNA | AAGAAGAAGCCAAGGAAAGAAAAAAGGAAGAAGCTAAAAGAGAAAAGGAACTAAAAAAGCAGCAACGTGCAGAAGAGAAACACAGAAAAGAGTTATTACGACAAGAAGAGAAAAAGAAAAAAGAGCTAAAGGCACAATCCCGTATTGGTAA |
| Cac1 KER::hKER Forward | GCACAATCCCGTATTGGTAA |
| Cac1 KER::hKER Reverse | CGATGAGGAAAGTTCTCTCT |
| Cac1 KER::hKER dsDNA -Fwd | AGAGAGAACTTTCCTCATCGGAAAAGAACAAACTGCGCCTGCAACGCGACC |
| Cac1 KER::hKER dsDNA -Rev | TTACCAATACGGGATTGTGCTTCCGCTTTGATACGCTTTTCTTC |
| Cac1 KER Forward | CTGTTCCAGGGGCCCCTGAAAAAGGAAGAAGCTAAAAGAGAAAAGG |
| Cac1 KER Reverse | GGGGACCACTTTGTACAAGAAAGCTGGGTCCTACTATCTTTCCTTGGCTTCTTCTTTC |
| Cac1 KER Reverse +Y | GGGGACCACTTTGTACAAGAAAGCTGGGTCCTACTAATATCTTTCCTTGGCTTCTTCTTT |
| Cac1 N-half Forward | CTGTTCCAGGGGCCCCTGAAAAAGGAAGAAGCTAAAAGAGAAAAGG |
| Cac1 N-half Reverse | GGGGACCACTTTGTACAAGAAAGCTGGGTCCTACTAATACTTTAGCTCTTTTTTCTTTTT |
| Cac1 middle-A dsDNA | GGGGACAAGTTTGTACAAAAAAGCAGGCTTCCTGGAAGTTCTGTTCCAGGGGCCCCTGAAACATCGTAAAGAATTACTTCGTCAAGAAGAAAAGAAAAAGAAGGAACTTAAAGTAGAGGAAGAACGGCAGCGGCGGGCTGAACTGAAAAAGCAGAAGGAAGAGGAAAAACGGCGTAAAGAGGAGGCGCGTTTGGAGGCCAAACGGCGCTAT  TAGTAGGACCCAGCTTTCTTGTACAAAGTGGTCCCC |
| Cac1 middle-B dsDNA | GGGGACAAGTTTGTACAAAAAAGCAGGCTTCCTGGAAGTTCTGTTCCAGGGGCCCCTGGTAGAGGAAGAACGGCAGCGGCGGGCTGAACTGAAAAAGCAGAAGGAAGAGGAAAAACGGCGTAAAGAGGAGGCGCGTTTGGAGGCCAAACGGCGCTATTAGTAGGACCCAGCTTTCTTGTACAAAGTGGTCCCC |
| Cac1 C-half Forward | CTGTTCCAGGGGCCCCTGAGGCGTGCTGAGCTGAAAAAGC |
|  |  |
| Cac1 C-half Reverse | GGGGACCACTTTGTACAAGAAAGCTGGGTCCTACTAATACTCTTTTCTTCTTTT GGC |
| Myosin 7a SAH dsDNA | GGGGACAAGTTTGTACAAAAAAGCAGGCTTCCTGGAAGTTCTGTTCCAGGGGCCCCTGCGCCTGGAAGCGGAACGCATGCGCCTGGCGGAAGAAGAAAAACTGCGCAAAGAAATGAGCGCGAAAAAAGCGAAAGAAGAAGCGGAACGCAAACATCAGGAACGCCTGGCGCAGCTGGCGCGCGAAGATGCGGAACGCGAACTGAAAGAAAAAGAAGAAGCGCGCCGCAAAAAAGAACTGCTGGAACAGATGGAAAAAGCG  TATTAGTAGGACCCAGCTTTCTTGTACAAAGTGGTCCCC |
| CHAF1A dsDNA for cloning in pGEX-6P-1 | GGGGCCCCTGGGATCCATGGATTGCAAAGATCGCCCGGCGTTTCCGGTGAAAAAACTGATTCAGGCGCGCCTGCCGTTCAAGCGCCTGAACCTGGTGCCGAAAGGCAAAGCGGATGATATGAGCGACGATCAAGGTACGAGCGTTCAGAGCAAATCGCCGGATCTGGAAGCCAGCCTGGATACGCTGGAAAACAACTGTCACGTGGGTAGCGATATTGACTTTCGCCCGAAACTGGTTAATGGTAAAGGCCCGCTGGATAATTTTCTGCGCAACCGCATTGAAACCAGCATCGGCCAGAGCACCGTTATCATTGATCTGACCGAGGATAGCAACGAGCAGCCGGATAGCCTGGTGGATCATAACAAACTGAACAGCGAGGCGAGCCCGTCGCGCGAGGCGATCAATGGCCAGCGCGAAGACACCGGTGATCAACAGGGTTTACTGAAGGCGATTCAAAATGACAAACTGGCCTTCCCGGGTGAAACCCTGAGCGACATTCCGTGTAAGACCGAGGAAGAAGGTGTGGGTTGTGGTGGCGCGGGCCGCCGTGGCGATAGCCAGGAATGCAGCCCGCGCAGCTGTCCGGAACTGACGAGCGGTCCGCGCATGTGTCCGCGCAAGGAACAAGATAGCTGGAGCGAGGCCGGCGGCATTCTGTTCAAGGGAAAAGTTCCGATGGTTGTTCTGCAGGACATTCTGGCGGTGCGCCCTCCGCAGATCAAAAGCCTGCCGGCCACGCCGCAAGGCAAAAACATGACGCCGGAAAGCGAAGTGCTGGAAAGCTTTCCGGAAGAGGACAGCGTTCTGAGCCATTCGAGCCTGTCGAGCCCGAGCAGCACCTCGAGCCCGGAAGGTCCTCCGGCCCCGCCGAAGCAGCATTCGAGCACGAGCCCGTTTCCGACGAGCACCCCGCTGCGTCGCATTACCAAGAAATTTGTGAAAGGTAGCACGGAAAAGAACAAACTGCGCCTGCAACGCGACCAAGAACGCCTGGGTAAGCAACTGAAACTGCGCGCCGAGCGCGAGGAAAAGGAAAAGCTGAAAGAGGAAGCCAAACGCGCCAAGGAGGAGGCGAAGAAAAAAAAGGAGGAAGAAAAGGAACTGAAGGAGAAAGAGCGCCGTGAAAAGCGCGAAAAGGATGAGAAGGAAAAAGCGGAAAAACAACGCCTGAAGGAGGAACGCCGTAAAGAACGCCAGGAAGCCCTGGAAGCCAAACTGGAAGAAAAACGCAAAAAGGAGGAAGAAAAGCGTCTGCGCGAGGAAGAAAAGCGTATCAAAGCGGAAAAGGCGGAAATTACCCGCTTCTTCCAGAAGCCGAAGACGCCTCAGGCCCCGAAGACGCTGGCGGGTAGCTGTGGTAAATTTGCCCCGTTCGAGATTAAGGAACATATGGTGCTGGCCCCGCGTCGCCGCACGGCGTTTCATCCGGACCTGTGCAGCCAGCTGGACCAGCTGCTGCAGCAGCAGAGCGGTGAGTTCTCGTTCCTGAAGGATTTAAAGGGCCGCCAACCGCTGCGCAGCGGTCCGACCCACGTTAGCACGCGCAACGCCGATATCTTCAATAGCGACGTTGTGATCGTGGAGCGCGGCAAAGGCGACGGTGTTCCGGAGCGTCGCAAGTTTGGACGCATGAAGTTACTGCAATTCTGCGAGAACCATCGCCCGGCCTATTGGGGCACGTGGAACAAGAAAACTGCGCTGATTCGCGCGCGTGATCCGTGGGCCCAGGATACGAAGTTACTGGACTACGAAGTTGATAGCGATGAAGAGTGGGAAGAAGAGGAACCGGGTGAGAGCCTGTCGCACAGCGAGGGCGACGATGATGACGACATGGGTGAGGATGAGGACGAAGACGATGGTTTCTTTGTGCCTCATGGTTACCTGAGCGAAGACGAGGGTGTTACCGAAGAGTGTGCGGACCCGGAAAACCATAAGGTGCGCCAGAAGCTGAAGGCCAAAGAGTGGGACGAGTTCCTGGCGAAGGGCAAACGTTTTCGCGTTCTGCAGCCGGTTAAAATTGGCTGTGTTTGGGCCGCGGATCGCGACTGCGCGGGTGATGACCTGAAAGTTCTGCAGCAATTCGCGGCCTGCTTCCTGGAGACCCTGCCGGCGCAGGAGGAACAAACCCCGAAAGCCAGCAAACGCGAACGTCGCGATGAGCAGATTCTGGCGCAGTTACTGCCGTTACTGCATGGCAACGTTAACGGTAGCAAAGTGATCATTCGCGAATTCCAGGAGCACTGCCGTCGCGGTTTACTGAGCAATCATACCGGTAGCCCGCGCACGCCGAGCACCACCTACCTGCATACGCCGACCCCGAGCGAGGATGCCGCGATTCCGAGCAAGTCGCGCCTGAAGCGCCTGATTAGCGAAAATAGCGTTTACGAAAAGCGCCCGGACTTTCGCATGTGTTGGTACGTGCATCCGCAGGTGCTGCAGAGCTTTCAGCAGGAACATCTGCCGGTTCCGTGCCAATGGAGCTACGTTACGAGCGTGCCGAGCGCCCCGAAAGAGGATAGCGGTAGCGTGCCGAGCACGGGTCCGAGCCAAGGTACCCCGATCAGCCTGAAGCGCAAGAGCGCCGGTAGCATGTGCATTACCCAATTTATGAAGAAACGTCGCCATGACGGCCAGATCGGTGCGGAGGACATGGACGGTTTTCAAGCGGATACCGAGGAAGAGGAAGAAGAAGAGGGCGACTGCATGATTGTTGATGTTCCGGACGCCGTGGAAGTTCAGGCCCCGTGTGGTGCCGCGAGCGGGGCCGGTGGCGGCGTTGGCGTGGATACCGGAAAAGCGACCCTGACGGCCAGCCCGCTGGGTGCGAGCTAAGGATCCCCGGAATTCC |
| CHAF1A KER (hKER) Forward | GGGGACAAGTTTGTACAAAAAAGCAGGCTTCCTGGAAGTTCTGTTCCAGGGGCCCCTGGAAAAGAACAAACTGCGCCTGCAACGCGACC |
| CHAF1A KER (hKER) Reverse | GGGGACCACTTTGTACAAGAAAGCTGGGTCCTACTAATATTCCGCTTTGATACGCTTTTCTTC |
| CAC1_FLAG_gRNA_F | CTTTCCGTTCAAGTTACAAAGACG |
| CAC1_FLAG_gRNA_R | AAACCGTCTTTGTAACTTGAACGG |
| mPIP_∆225-226_gRNA_F | CTTTACGTTTGAAAGAAGAAGCCA |
| mPIP_∆225-226_gRNA_R | AAACTGGCTTCTTCTTTCAAACGT |
| mWHD_gRNA_F | CTTTAACAATTAAAAACACCATAA |
| mWHD_gRNA_R | AAACTTATGGTGTTTTTAATTGTT |
| ∆KER_gRNA_F | CTTTAGGTAGAAGAGGAAAGACAA |
| ∆KER_gRNA_F | AAACTTGTCTTTCCTCTTCTACCT |
| ∆145-149_gRNA_R | CTTTGGAAGAAGCTAAAAGAGAAA |
| ∆145-149_gRNA_R | TTTCTCTTTTAGCTTCTTCC |
| CAC1_FLAG_HR_F | CCAATGCAAATATGCCAACCCCGTCTTTGGGATCCGCTGGCTCCGCTGCTGGTTCTGGCG |
| CAC1_FLAG_HR_M | GGTTCTGGCGATTACAAGGATGACGACGATAAGGACTATAAGGACGATGATGACAAGGACTACAAAGATGATGACGATAAATAACTTGAA |
| CAC1_FLAG_HR_R | TACCAATAAATAATCAGTTTATCTGTATGTTTCTATATACTAAAGATCCGTTCAAGTTAT |
| CAC1_mPIP_HR_F | AAATACGTTTGAAAGAAGAAGCCAAAGAAAGAGCACAATCCCGTATTGGTAACGC |
| CAC1_mPIP_HR_M | TTGGTAACGCCGCGAAAAAACTAAGCGATTCTAATACGCCTGTGGTTGAAAAGTCGGATT |
| CAC1_mPIP_HR_R | CTCTAACTCCATCTTTAGCATAGAAAGGTAGAAAAAATTTTTCATAATCCGACTT |
| CAC1_mWHD_HR_F | CAGCACGTTTTCTTTGGGTACTGTGACTGAAATAGCACAGAAAAATTTGCCGCAATACAA |
| CAC1_mWHD_HR_M | CGCAATACAACAAACAAACAATTGAAAACACCATAGAGGAATATGCCATAAGAAGTTCTG |
| CAC1_mWHD_HR_R | CCAGTTTTGTGCGTCTTTGATTACCCATTTGCGGGGCAAATCACCCTTTCCAGAACTTCT |
| CAC1_∆KER_HR_F | TACCCAATGGAAATATAATAGCTATCGAGACAAAAAGCAGAAGCTCTTCTCCATGCTCTA |
| CAC1_∆KER_HR_M | TCTCCATGCTCTAAGAGAGAACTTTCCTCATCGGCACAATCCCGTATTGGTAACTTCTTT |
| CAC1_∆KER_HR_R | TCATAATCCGACTTTTCAACCACAGGCGTATTAGAATCGCTTAGTTTTTTAAAGAAGTTA |
| CAC1_∆KERmPIP_HR_M | TCTCCATGCTCTAAGAGAGAACTTTCCTCATCGGCACAATCCCGTATTGGTAACGCCGCG |
| CAC1_∆KERmPIP_HR_R | TCATAATCCGACTTTTCAACCACAGGCGTATTAGAATCGCTTAGTTTTTTCGCGGCGTTA |
| CAC1_∆1-2KER_HR_F | CTAAGAGAGAACTTTCCTCATCGAAAAAGGAAGAAGCTAAAAGAGAAAAGGAACTAAAAA |
| CAC1_∆1-2KER_HR_M | GAACTAAAAAAGCAGCAACGTGCAGAAGAGAAAGAGGAGGAAAGATTGAAAAAGGAGGAG |
| CAC1_∆1-2KER_HR_R | CCAATACGGGATTGTGCTCTTTCCTTGGCTTCTTCTTTCAAACGTATTTCCTCCTCCTTT |
| CAC1_2xandHumanKER_HR_F | GCTATCGAGACAAAAAGCAGAAGCTCTTCTCCATGCTCTAAGAGAGAACTTTCCTCATCG |
| CAC1_2xandHumanKER_HR_R | AACCACAGGCGTATTAGAATCGCTTAGTTTTTTAAAGAAGTTACCAATACGGGATTGTGC |
| CAC1_∆225-226_HR_F | GATTAGAAGCCAAAAGAAGAAAAGAGGAGGAAAGATTGAAAAAGGAAGAGGAAATACGTT |
| CAC1_∆225-22_HR_M | GAAATACGTTTGAAAGAAGAAGCCAAAGAACAATCCCGTATTGGTAACTTCTTTAAAAAA |
| CAC1_∆225-226_HR_R | AATTTTTCATAATCCGACTTTTCAACCACAGGCGTATTAGAATCGCTTAGTTTTTTAAAG |
| CAC1_∆225-226mPIP_HR_M | GAAATACGTTTGAAAGAAGAAGCCAAAGAACAATCCCGTATTGGTAACGCCGCGAAAAAA |
| CAC1_∆225-226mPIP_HR_R | AATTTTTCATAATCCGACTTTTCAACCACAGGCGTATTAGAATCGCTTAGTTTTTTCGCG |
| CAC1_∆145-149_HR_F | AAAAGCAGAAGCTCTTCTCCATGCTCTAAGAGAGAACTTTCCTCATCGAAAAAGGAAGAA |
| CAC1_∆145-149_HR_M | AAAGGAAGAAGCTAAAAGAGAAAAGCAACGTGCAGAAGAGAAACACAGAA |
| CAC1_∆145-149_HR_R | TTCTACCTTTAGCTCTTTTTTCTTTTTCTCTTCTTGTCGTAATAACTCTTTTCTGTGTTT |
| Yeast::Human KER | AAGAGAGAACTTTCCTCATCGGAAAAAAATAAGCTGAGATTACAACGTGACCAAGAGAGGCTGGGAAAGCAACTTAAACTTAGAGCCGAGCGTGAAGAAAAGGAGAAGCTAAAAGAAGAAGCTAAGAGGGCTAAGGAAGAGGCTAAAAAAAAGAAAGAAGAAGAGAAAGAGTTGAAAGAGAAAGAAAGGAGGGAAAAACGTGAAAAGGATGAAAAGGAGAAGGCAGAAAAGCAGCGTTTAAAAGAAGAGAGAAGAAAGGAACGTCAAGAAGCATTAGAAGCAAAGCTGGAGGAAAAGAGGAAGAAAGAAGAGGAAAAGAGGTTACGTGAAGAAGAAAAAAGAATAAAGGCTGAGGCACAATCCCGTATTGGTAAC |
| 2xKER | AAGAGAGAACTTTCCTCATCGAAAAAAGAAGAAGCAAAGAGGGAAAAGGAGCTTAAAAAGCAACAACGTGCTGAAGAGAAGCATAGGAAAGAATTGTTGAGACAGGAAGAAAAAAAGAAGAAAGAACTGAAGGTGGAGGAAGAGAGACAAAGGAGGGCCGAACTAAAAAAGCAGAAGGAGGAGGAAAAACGTCGTAAAGAAGAGGCGAGACTAGAGGCGAAAAGGAGAAAAGAAGAAGAAAGGTTGAAAAAGGAGGAGGAAATTAGGTTAAAAGAAGAAGCGAAGGAAAGGAAAAAGGAGGAGGCAAAGCGTGAGAAGGAGCTAAAGAAACAGCAACGTGCAGAAGAGAAACACAGAAAGGAGTTGTTGAGGCAGGAAGAAAAAAAAAAAAAGGAACTAAAAGTCGAAGAAGAAAGACAGAGACGTGCCGAACTAAAAAAACAAAAAGAAGAAGAGAAGCGTAGAAAGGAAGAAGCAAGGCTTGAAGCAAAACGTAGGAAGGAAGAGGAACGTCTGAAGAAAGAGGAGGAGATCAGACTAAAGGAAGAGGCTAAAGAGAGAGCACAATCCCGTATTGGTAAC |
| rtt106_HIS_F | TGTAGTAATAACTATGATGTAAAGGTGCTGGAAACGCTGACAGCTGAAGCTTCGTACGC |
| rtt106_HIS_R | TATTCTTCAGGATAAAAAAAGTGGTATTTATGAACTCTTACATAGGCCACTAGTGGATCTG |
| cac1_KAN_F | ACATTCTGTTATTGCTGTTACAGAGAATTATATGTTTTAGCAGCTGAAGCTTCGTACGC |
| cac1_KAN_R | TAGTGTTGTCGCCTTTTTCATGTATACCAATAAATAATCACATAGGCCACTAGTGGATCTG |
| sir2_KAN_F | CCCATCTCAGAGAAAAAACGAGG |
| sir2_KAN_R | AGCTATTTGTGAGAGCCTTGCGTC |
| bar1_LEU2_F | GCCAGCTATTCTGAAACACACCAC |
| bar1_LEU2_R | GCTACTTGTTCAAAATTGTGATGGCTGC |
| bar1_LEU2_R | GCTACTTGTTCAAAATTGTGATGGCTGC |
