# Supplementary material for: A novel single alpha-helix DNA-binding domain in CAF-1 promotes gene silencing and DNA damage survival through tetrasome-length DNA selectivity and spacer function
Source: eLife. 2023 Jul 11;12:e83538. doi: 10.7554/eLife.83538 (PMC10335832; doi:10.7554/eLife.83538)
Supplement: Supplementary file 3. [file elife-83538-supp3.docx]

**Supplementary Table 3.**  List of yeast strains.

| **Strain** | **Mutation** | **Genotype** | **Reference** |
| --- | --- | --- | --- |
| W303-1a |  | *Mat a ade2-1 leu2-2,112 his3-11,15 trp1 ura3-1 can1-100* | ([Thomas and Rothstein, 1989](#_ENREF_54)) |
| RAY160 | rtt106Δ | *Mat a ade2-1 leu2-2,112 his3-11,15 trp1 ura3-1 can1-100 rtt106::HIS3* | This study |
| RAY165 | rtt106Δcac1Δ | *Mat a ade2-1 leu2-2,112 his3-11,15 trp1 ura3-1 can1-100 rtt106::HIS3 cac1::KANMX* | This study |
| RAY187 | mPIP | RAY160 *CAC1 F233A/F234A* | This study |
| RAY180 | mWHD | RAY160 *CAC1 K564E/K568E* | This study |
| RAY192 | mPIP + mWHD | RAY160 *CAC1 F233A/F234A/K564E/K568E* | This study |
| RAY264 | ΔKER) | RAY160 *CAC1 Δ136-225* | This study |
| RAY265 | ΔKER + mPIP | RAY264 *CAC1 F233A/F234A* | This study |
| RAY266 | ΔKER + mWHD | RAY264 *CAC1 K564E/K568E* | This study |
| RAY221 | Δmiddle-AKER | RAY160 *CAC1 Δ155-204* | This study |
| RAY222 | Δmiddle-AKER + mPIP | RAY221 *CAC1 F233A/F234A* | This study |
| RAY223 | Δmiddle-AKER + mWHD | RAY221 *CAC1 K564E/K568E* | This study |
| RAY245 | 2xKER) | RAY160 *CAC1 dup(136-225)* | This study |
| RAY258 | 2xKER + mPIP | RAY245 *CAC1 F233A/F234A* | This study |
| RAY247 | 2xKER + mWHD | RAY245 *CAC1 K564E/K568E* | This study |
| RAY233 | yeast KER::human KER | RAY160 *CAC1 136-225::hCHAF1A 331-441* | This study |
| RAY256 | yeast KER::human KER + mPIP | RAY233 *CAC1 F233A/F234A* | This study |
| RAY243 | yeast KER::human KER + mWHD | RAY233 *CAC1 K564E/K568E* | This study |
| RAY226 | Δ225-226 | RAY160 *CAC1 Δ225-226* | This study |
| RAY239 | Δ225-226 + mPIP | RAY226 *CAC1 F223A/F234A* | This study |
| RAY241 | Δ225-226 + mWHD | RAY226 *CAC1 K564E/K568E* | This study |
| RAY207 | ∆145-149 | RAY160 *CAC1 Δ145-149* | This study |
| RAY216 | ∆145-149 + mPIP | RAY207 *CAC1 F233A/F234A* | This study |
| RAY218 | ∆145-149 + mWHD | RAY207 *CAC1 K564E/K568E* | This study |
| BY4741 |  | *Mat a his3∆1 leu2∆0 met15∆0 ura3∆0* | ([Baker Brachmann et al., 1998](#_ENREF_3)) |
| ^a^RAY156 |  | *Mat a his3∆1 leu2∆0 met15∆0 ura3∆0 hmr::P_URA3_-GFP/URA3 TRP::BrdU-Inc(TRP) bar1::LEU2* | This study |
| ^a^RAY152 | *sir2∆* | *Mat a his3∆1 leu2∆0 met15∆0 ura3∆0 hmr::P_URA3_-GFP/URA3 TRP::BrdU-Inc(TRP) sir2::KANMX* | This study |
| ^a^RAY177 | *rtt106∆* | *Mat a his3∆1 leu2∆0 met15∆0 ura3∆0 hmr::P_URA3_-GFP/URA3 TRP::BrdU-Inc(TRP) bar1::LEU2 rtt106::HIS3* | This study |
| ^a^RAY179 | *cac1∆* | *Mat a his3∆1 leu2∆0 met15∆0 ura3∆0 hmr::P_URA3_-GFP/URA3 TRP::BrdU-Inc(TRP) bar1::LEU2 cac1::KANMX* | This study |
| ^a^RAY189 | *rtt106∆cac1∆* | *Mat a his3∆1 leu2∆0 met15∆0 ura3∆0 hmr::P_URA3_-GFP/URA3 TRP::BrdU-Inc(TRP) bar1::LEU2 rtt106::HIS3 cac1::KANMX* | This study |
| RAY193 | mPIP | RAY177 *CAC1 F233A/F234A* | This study |
| RAY194 | mWHD | RAY177 *CAC1 K564E/K568E* | This study |
| RAY199 | mPIP + mWHD | RAY177 *CAC1 F233A/F234A/K564E/K568E* | This study |
| RAY205 | ∆KER | RAY177 *CAC1 Δ136-225* | This study |
| RAY212 | ∆KER + mPIP | RAY205 *CAC1 F233A/F234A* | This study |
| RAY206 | ∆KER + mWHD | RAY205 *CAC1 K564E/K568E* | This study |
| RAY219 | ∆middle-A | RAY177 *CAC1 Δ155-204* | This study |
| RAY259 | ∆middle-A + mPIP | RAY219 *CAC1 F233A/F234A* | This study |
| RAY230 | ∆middle-A + mWHD | RAY219 *CAC1 K564E/K568E* | This study |
| RAY244 | 2xKER | RAY177 *CAC1 dup(136-225)* | This study |
| RAY263 | 2xKER + mPIP | RAY244 *CAC1 F233A/F234A* | This study |
| RAY246 | 2xKER + mWHD | RAY244 *CAC1 K564E/K568E* | This study |
| RAY232 | yeast KER::human KER | RAY177 *CAC1 136-225::hCHAF1A 331-441* | This study |
| RAY257 | yeast KER::human KER + mPIP | RAY232 *CAC1 F233A/F234A* | This study |
| RAY242 | yeast KER::human KER + mWHD | RAY232 *CAC1 K564E/K568E* | This study |
| RAY225 | ∆225-226 | RAY177 *CAC1 Δ225-226* | This study |
| RAY238 | ∆225-226 + mPIP | RAY225 *CAC1 F223A/F234A* | This study |
| RAY240 | ∆225-226 + mWHD | RAY225 *CAC1 K564E/K568E* | This study |
| RAY208 | ∆145-149 | RAY177 *CAC1 Δ145-149* | This study |
| RAY209 | ∆145-149 + mPIP | RAY208 *CAC1 F233A/F234A* | This study |
| RAY231 | ∆145-149 + mWHD | RAY208 *CAC1 K564E/K568E* | This study |
| RAY191 | CAC1-FLAG | *Mat a ade2-1 leu2-2,112 his3-11,15 trp1 ura3-1 can1-100 rtt106::HIS3 CAC1-3xFLAG* | This study |
| RAY195 | CAC1-FLAG mPIP | RAY191 *CAC1 F233A/F234A* | This study |
| RAY196 | CAC1-FLAG mWHD | RAY191 *CAC1 K564E/K568E* | This study |
| RAY197 | CAC1-FLAG mPIP + mWHD | RAY191 *CAC1 F233A/F234A/K564E/K568E* | This study |
| RAY203 | CAC1-FLAG ∆KER | RAY191 *CAC1 Δ136-225* | This study |
| RAY214 | CAC1-FLAG ∆KER + mPIP | RAY214 *CAC1 F233A/F234A* | This study |
| RAY207 | CAC1-FLAG ∆KER + mWHD | RAY214 *CAC1 K564E/K568E* | This study |
| RAY220 | CAC1-FLAG Δmiddle-A | RAY191 *CAC1 Δ155-204* | This study |
| RAY250 | CAC1-FLAG ∆middle-A + mPIP | RAY250 *CAC1 F233A/F234A* | This study |
| RAY224 | CAC1-FLAG ∆middle-A + mWHD | RAY250 *CAC1 K564E/K568E* | This study |
| RAY254 | CAC1-FLAG 2xKER | RAY191 *CAC1 dup(136-225)* | This study |
| RAY255 | CAC1-FLAG 2xKER + mPIP | RAY254 *CAC1 F233A/F234A* | This study |
| RAY261 | CAC1-FLAG 2xKER + mWHD | RAY254 *CAC1 K564E/K568E* | This study |
| RAY262 | CAC1-FLAG yeast KER::human KER | RAY191 *CAC1 136-225::hCHAF1A 331-441* | This study |
| RAY260 | CAC1-FLAG yeast KER::human KER + mPIP | RAY262 *CAC1 F233A/F234A* | This study |
| RAY253 | CAC1-FLAG yeast KER::human KER + mWHD | RAY262 *CAC1 K564E/K568E* | This study |
| RAY229 | CAC1-FLAG ∆225-226 | RAY191 *CAC1 Δ225-226* | This study |
| RAY248 | CAC1-FLAG ∆225-226 + mPIP | RAY248 *CAC1 F223A/F234A* | This study |
| RAY249 | CAC1-FLAG ∆225-226 + mWHD | RAY229 *CAC1 K564E/K568E* | This study |
| RAY215 | CAC1-FLAG ∆145-149 | RAY191 *CAC1 Δ145-149* | This study |
| RAY251 | CAC1-FLAG ∆145-149 + mPIP | RAY251 *CAC1 F233A/F234A* | This study |
| RAY217 | CAC1-FLAG ∆145-149 + mWHD | RAY251 *CAC1 K564E/K568E* | This study |
| YDS12 | tetO7-CDC9 | *Mat ⍺ ade2-1 trp 1-1 cac1-100 leu2-3,112 his3-11,15 ura3-1 cdc9::tetO7-CDC9 cmv-Laci-NAT* | ([Yeung and Smith, 2020](#_ENREF_64)) |
| NAY001 | tetO7-CDC9 | *Mat a ade2-1 trp 1-1 cac1-100 leu2-3,112 his3-11,15 ura3-1 cdc9::tetO7-CDC9 cmv_Laci-NAT* | This study |
| NAY002 | tetO7-CDC9 + *rtt106Δ* | *Mat a ade2-1 trp 1-1 cac1-100 leu2-3,112 his3-11,15 ura3-1 cdc9::tetO7-CDC9 cmv_Laci-NAT rtt106::HIS* | This study |
| NAY003 | tetO7-CDC9 + *cac1Δ* | *Mat a ade2-1 trp 1-1 cac1-100 leu2-3,112 his3-11,15 ura3-1 cdc9::tetO7-CDC9 cmv_Laci-NAT cac1::KAN* | This study |
| NAY004 | tetO7-CDC9 + *cac1Δ + rtt106Δ* | *Mat a ade2-1 trp 1-1 cac1-100 leu2-3,112 his3-11,15 ura3-1 cdc9::tetO7-CDC9 cmv_Laci-NAT rtt106::HIS cac1::KAN* | This study |
| NAY005 | tetO7-CDC9 + ΔKER | NAY001 *CAC1 Δ136-225* | This study |
| NAY006 | tetO7-CDC9 + *rtt106Δ* + ΔKER | NAY002 *CAC1 Δ136-225* | This study |
| NAY007 | tetO7-CDC9 + Δmiddle-AKER | NAY001 *CAC1 Δ155-204* | This study |
| NAY008 | tetO7-CDC9 + *rtt106Δ* + Δmiddle-AKER | NAY002 *CAC1 Δ155-204* | This study |
| NAY009 | tetO7-CDC9 + 2xKER | NAY001 *CAC1 dup(136-225)* | This study |
| NAY010 | tetO7-CDC9 + *rtt106Δ* + 2xKER | NAY002 *CAC1 dup(136-225)* | This study |

*^a^ BrdU-Inc(*[Viggiani and Aparicio, 2006](#_ENREF_56)*); hmr::PURA3-GFP/URA3(*[Laney and Hochstrasser, 2003](#_ENREF_30)*).*

**Supplementary References**

Laney, Jeffrey D., and Mark Hochstrasser. 2003. 'Ubiquitin-dependent degradation of the yeast Matα2 repressor enables a switch in developmental state', *Genes and Development*, 17: 2259-70.

Viggiani, Christopher J., and Oscar M. Aparicio. 2006. 'New vectors for simplified construction of BrdU-Incorporating strains of Saccharomyces cerevisiae', *Yeast*, 23: 1045-51.
