# Supplementary material for: A novel single alpha-helix DNA-binding domain in CAF-1 promotes gene silencing and DNA damage survival through tetrasome-length DNA selectivity and spacer function
Source: eLife. 2023 Jul 11;12:e83538. doi: 10.7554/eLife.83538 (PMC10335832; doi:10.7554/eLife.83538)
Supplement: Supplementary file 4. [file elife-83538-supp4.docx]

**Supplementary Table 4.** Statistics for crystallographic data collection and refinement.

| Data statistics | MBP-KER (PDB: 8DEI) |
| --- | --- |
| Spacegroup | P2_1_ |
| Cell dimensions (Å) | a=53.586 b=165.249 c=116.149  α=90° β=96.47° γ=90° |
| Resolution range (Å) | 26.68 - 2.81 (2.9? - 2.81)^a^ |
| Total reflections | 188,138 (13,259) ^a^ |
| Unique reflections | 48320 (4400) ^a^ |
| Redundancy | 3.9 (3.0) ^a^ |
| R_sym_^b^ (%) | 17.6 (68.9)^a^ |
| Completeness (%) | 97.76 (90.08) ^a^ |
| Intensity (I/σ) | 7.3 (1.5) ^a^ |
| CC1/2 | .979 (695) |
| Wilson B factor (Å^2^) | 46.75 |
| Refinement statistics |  |
| Resolution Range (Å) | 28.68 - 2.81 (2.91 - 2.81) ^a^ |
| Unique reflections | 47,773 (4364) |
| Rfree^c^ (%) | 28.25 (35.43) ^a^ |
| Rworking (%) | 23.30 (29.20) ^a^ |
| Final Model |  |
| Number of protein atoms | 14,659 |
| Number of heteroatoms | 106 |
| Number of solvent atoms | 11 |
| Average B factor (Å^2^) | 51.56 |
| R.m.s.d. bond lengths (Å) | 0.001 |
| R.m.s.d. bond angles (°) | 0.342 |
| Molprobity score | 1.39 |
| Ramachandran Analysis | 96.44% most favored; 3.56% allowed; 0% outlier |

*^a^ High resolution shell*

*^b^ R_sym_ = ∑|I - <I>|/∑I*

*^c^ R_free_ calculated with an excluded set of 10%*
